# Dynamically and Reversibly Functionalized Self-Assembled Platelets via Noncovalent Interactions

**DOI:** 10.1021/jacs.6c17888

**Published:** 2026-10-29

**Authors:** Tianlai Xia, Peng Sun, Laihui Xiao, Andrew P. Dove, Rachel K. O’Reilly

**Affiliations:** † School of Chemistry, 152859 University of Birmingham, Edgbaston, Birmingham B15 2TT, U.K.; ‡ School of Interdisciplinary Science, 47833 Beijing Institute of Technology, Beijing 100081, China

## Abstract

The creation of polymeric assemblies with reconfigurable surface functionality remains a major challenge for programmable soft materials. Covalent modification strategies can provide stable functional materials, but often require harsh reaction conditions or irreversible bond formation. Here, we integrate noncovalent interactions with crystallization-driven self-assembly (CDSA) to construct polymer platelets with region-selective functionalization and controllable responsiveness under mild conditions. Polycaprolactone-based platelets bearing cyclodextrin, barbituric acid, or related recognition motifs were prepared and subsequently orthogonal modified with complementary fluorescent guests through host–guest interactions or hydrogen bonding. Confocal fluorescence microscopy confirmed platelet-localized dye incorporation, while competitive displacement and relabeling demonstrated reversible molecular loading and release without disrupting platelet structural integrity. This work establishes supramolecular modification of CDSA-based assemblies as a modular route to soft materials with controllable and reconfigurable molecular functionality under mild conditions.

## Graphical abstract

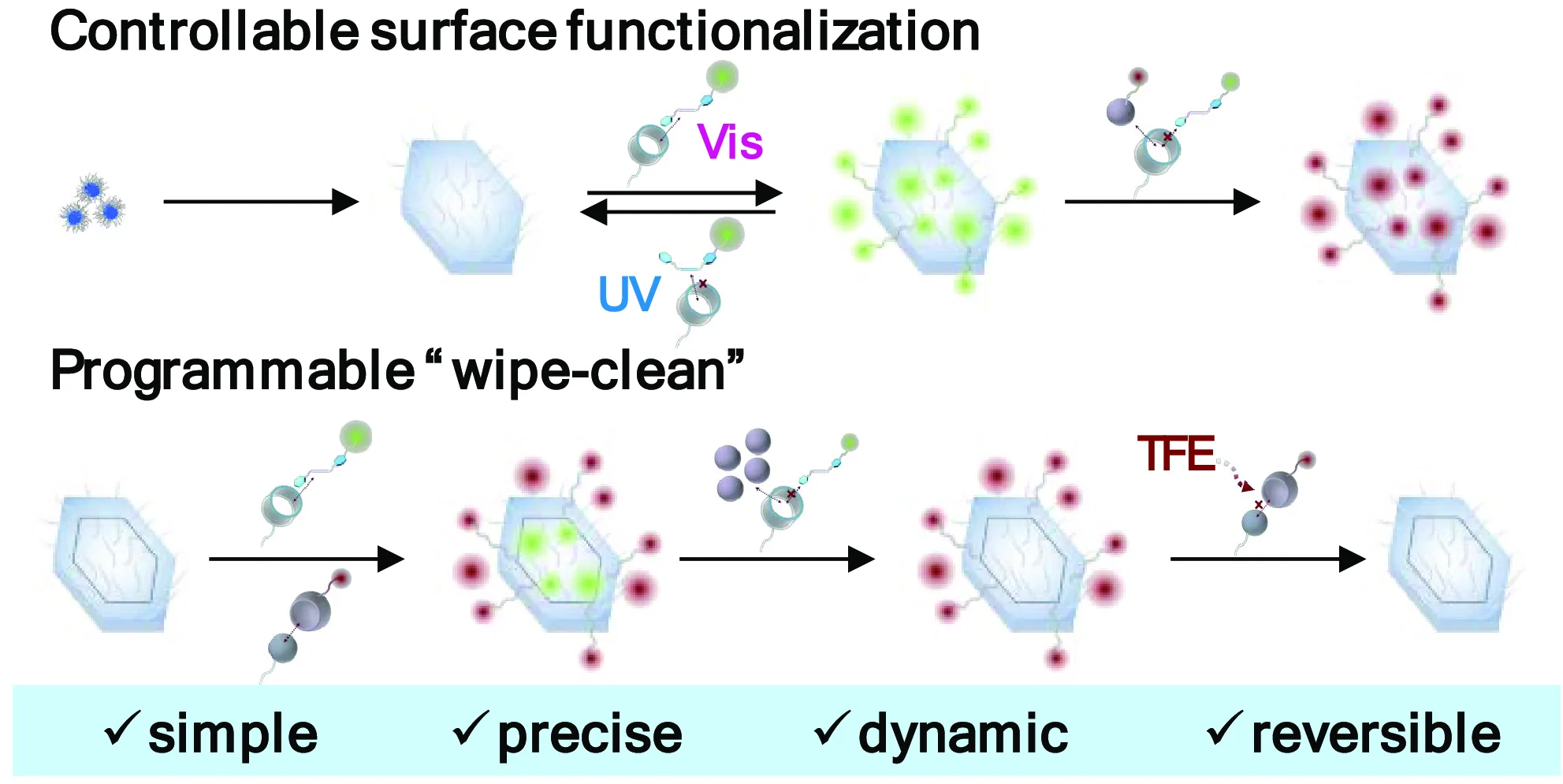

## Introduction

The precise engineering of polymeric nanostructures is central to advancing next-generation technologies in information storage, sensing, and molecular encoding,^1,2^ where the performance is often dictated by surface functionality.^3^ The advent of controllable polymer self-assembly has provided powerful and convenient tools for constructing polymeric nanomaterials with uniform structures and well-controlled morphologies.^4–28^ By integration of controllable self-assembly with covalent chemistry, polymer nanostructures with sophisticated architectures and versatile surface functionalities have been successfully developed.^16,29–32^ However, synthetic nanostructures remain functionally limited compared with the stimulus-regulated and reconfigurable soft matter systems.^33–35^ Covalent surface-functionalization approaches often suffer from harsh reaction conditions, limited region specificity, and poor dynamic adaptability. Harsh conditions can cause irreversible disruption of nanostructural morphology, insufficient precision can lead to compromised material performance, and the lack of dynamic behavior limits the applications of polymer nanomaterials.^36–38^ Developing a mild and efficient surface-functionalization strategy to construct polymer nanomaterials with structural precision, region-selective functionalization, and controllable responsiveness is of significant scientific interest and practical importance.^39–44^

Noncovalent interactions encompass intermolecular and intramolecular forces that do not involve covalent bond formation, and have been widely exploited in the design of supramolecular materials with stimuli-responsive, recyclable, and self-healing properties.^45,46^ Noncovalent interactions are distinguished by spontaneity under ambient conditions, high orthogonality, modest binding strength, and reversible association–dissociation behavior. The modest binding affinities allow supramolecular systems to respond rapidly to subtle environmental changes, conferring excellent stimuli-responsiveness.^47^ Their reversible nature enables assemblies to recover their original state following structural or functional transformations, imparting reconfigurability. Moreover, the spontaneous nature of noncovalent interactions facilitates processing under mild conditions, while orthogonality allows for site-specific modifications. Together, these features endow supramolecular materials with dynamic adaptability and responsiveness.

Here, we combine supramolecular chemistry with crystallization-driven self-assembly (CDSA) to create polymer platelets, where “platelet” refers to two-dimensional lamellar structures, with programmable and dynamic functionality (Scheme 1). By embedding orthogonal motifs such as host–guest pairs and multiple hydrogen-bonding units, surface modification can be achieved under mild conditions, enabling selective incorporation of distinct fluorophores into defined platelet regions. The dynamic nature and reversibility of noncovalent interactions allow competitive guest exchange, light-triggered switching, and programmable “wipe-clean” erasure, imparting reconfigurable properties to otherwise static assemblies. This approach establishes a platform in which molecular recognition governs precise, region-specific, and dynamic functionalization. This advance demonstrates how supramolecular modification of CDSA-derived polymer assemblies can be transformed from a static assembly method into a platform for adaptive and responsive soft materials.

**Scheme 1:**
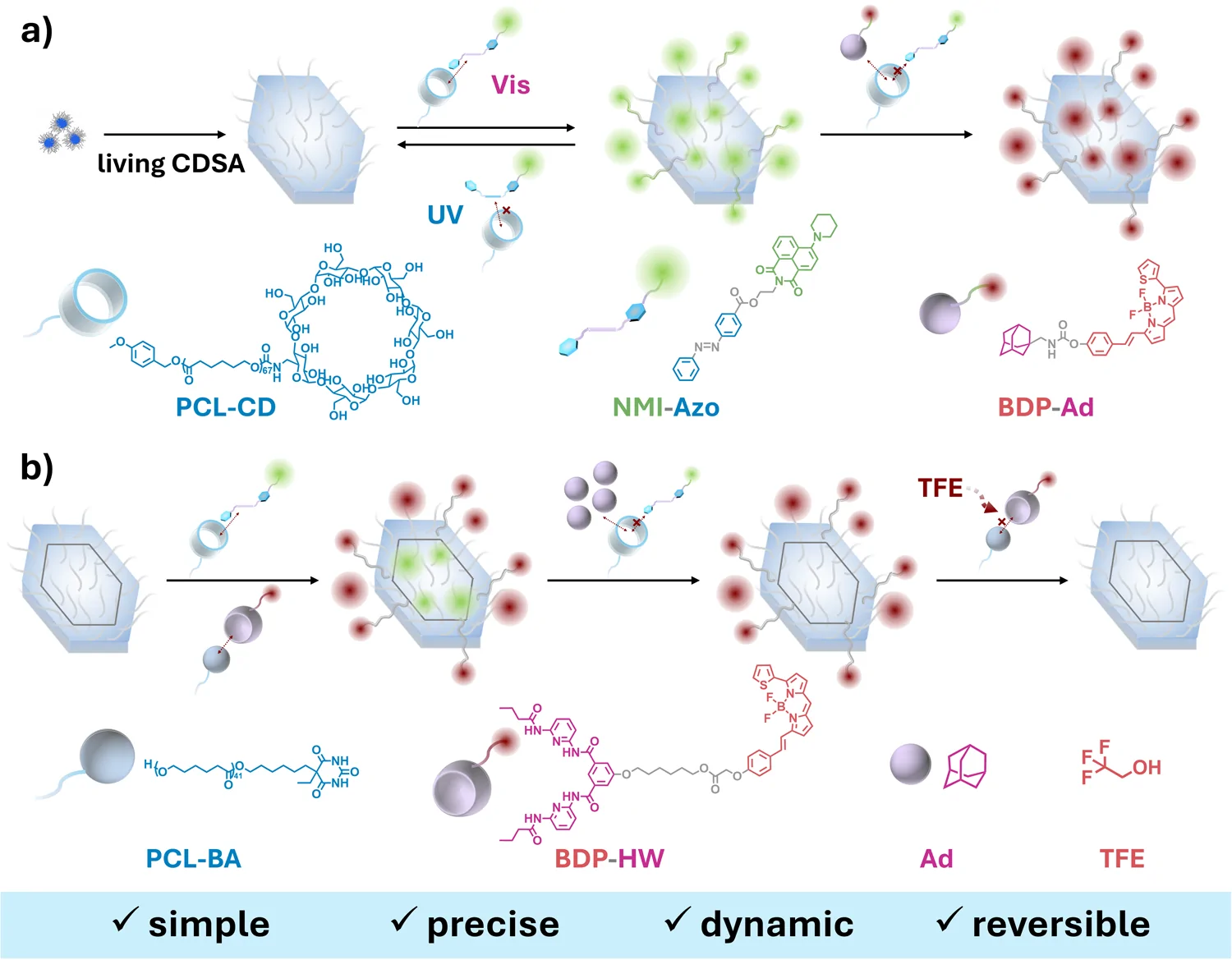
Supramolecularly Functionalized Platelets^a^

a (a) Schematic illustration of noncovalent surface functionalization of CDSA-derived platelets based on PCL-CD, showing reversible binding of azobenzene-functionalized dyes (NMI-Azo) through host–guest interactions with β-cyclodextrin under alternating UV and visible light. Competitive binding of adamantane-functionalized dyes (BDP-Ad) can displace NMI-Azo from the cyclodextrin cavity, resulting in a color change from green to red. (b) Schematic illustration of region-specific surface functionalization using platelets containing distinct supramolecular motifs. The inner region of the platelet is functionalized with PCL-CD, enabling host–guest interactions with azobenzene-functionalized dyes (NMI-Azo), while the outer region is functionalized with PCL-BA, allowing sextuple hydrogen bonding with Hamilton wedge-functionalized dyes (BDP-HW). Selective removal (“wipe-clean”) of bound species is achieved by competitive binding with free adamantane (Ad) for host–guest interaction and by disruption of hydrogen bonding using trifluoroethanol (TFE).

## Results and Discussion

Established poly­(ε-caprolactone) (PCL) based homopolymers (HP) and poly­(ε-caprolactone)-*block*-polydimethylacrylamide (PCL_50_-*b*-PDMA_193_) block copolymers (BCP) mixtures were employed to generate seeds and platelets using a living CDSA approach (Figure 1a).^16,19,44^ To introduce noncovalent interactions, the PCL HP chain ends were modified with representative motifs: β-cyclodextrin (CD), adamantane (Ad), barbiturate (BA), or a Hamilton wedge (HW). CD can form a supramolecular complex with Ad through host–guest interactions, while BA associates with HW via sextuple hydrogen-bonding to yield a supramolecular complex. These supramolecular functional homopolymers (PCL_67_-CD, PCL_54_-Ad, PCL_41_-BA, PCL_75_-HW) were synthesized via end-group modification of PCL–OH with CD-NH_2_ and ring-opening polymerization using supramolecular alcohol initiators (Ad–C3–OH, BA–OH, HW–OH). Although the resulting end-functionalized homopolymers possess different degrees of polymerization (DP = 41–75), this variation does not disrupt the CDSA process or the formation of platelet structures, but instead leads to differences in platelet aspect ratio.^44^ These homopolymers structures were confirmed by NMR spectroscopy, with molar mass and dispersity characterized by size-exclusion chromatography (SEC) analysis (Figures S1–S21 and S58–S64 and Table S1). A typical living CDSA protocol involved the addition of a unimer solution consisting of supramolecular motif-functionalized PCL-based HP and PCL-*b*-PDMA BCP mixtures (5 μL, 10 mg mL^–1^, *m*_HP_/*m*_BCP_ = 1/1, PCL_50_-*b*-PDMA_193_ was fixed as BCP) into a seed solution (500 μL, 0.01 mg mL^–1^ in EtOH), affording well-defined platelet nanostructures. In all cases, transmission electron microscopy (TEM) characterization (Figures 1a and S65) confirmed that the introduction of supramolecular motifs into PCL did not disrupt crystallization or morphology, while preserving the living growth behavior and enabling the formation of platelets with predictable sizes (Figure S66). Because of the crystallization-driven packing of the PCL core, the supramolecular units located at the polymer chain-ends remain exposed on the platelet surface rather than incorporated into the crystalline lattice. Complementary dyes bearing matching motifs, such as naphthalimide (NMI) functionalized adamantane (NMI-Ad) and β-cyclodextrin (NMI-CD), boron-dipyrromethene 630/650 (BDP) functionalized adamantane (BDP-Ad), Hamilton wedge (BDP-HW), and barbiturate (BDP-BA), and spiropyran (SPY) functionalized Hamilton wedge (SPY-HW), were also prepared to probe noncovalent interactions in subsequent studies (Figures S22–S57).

**Figure 1:**
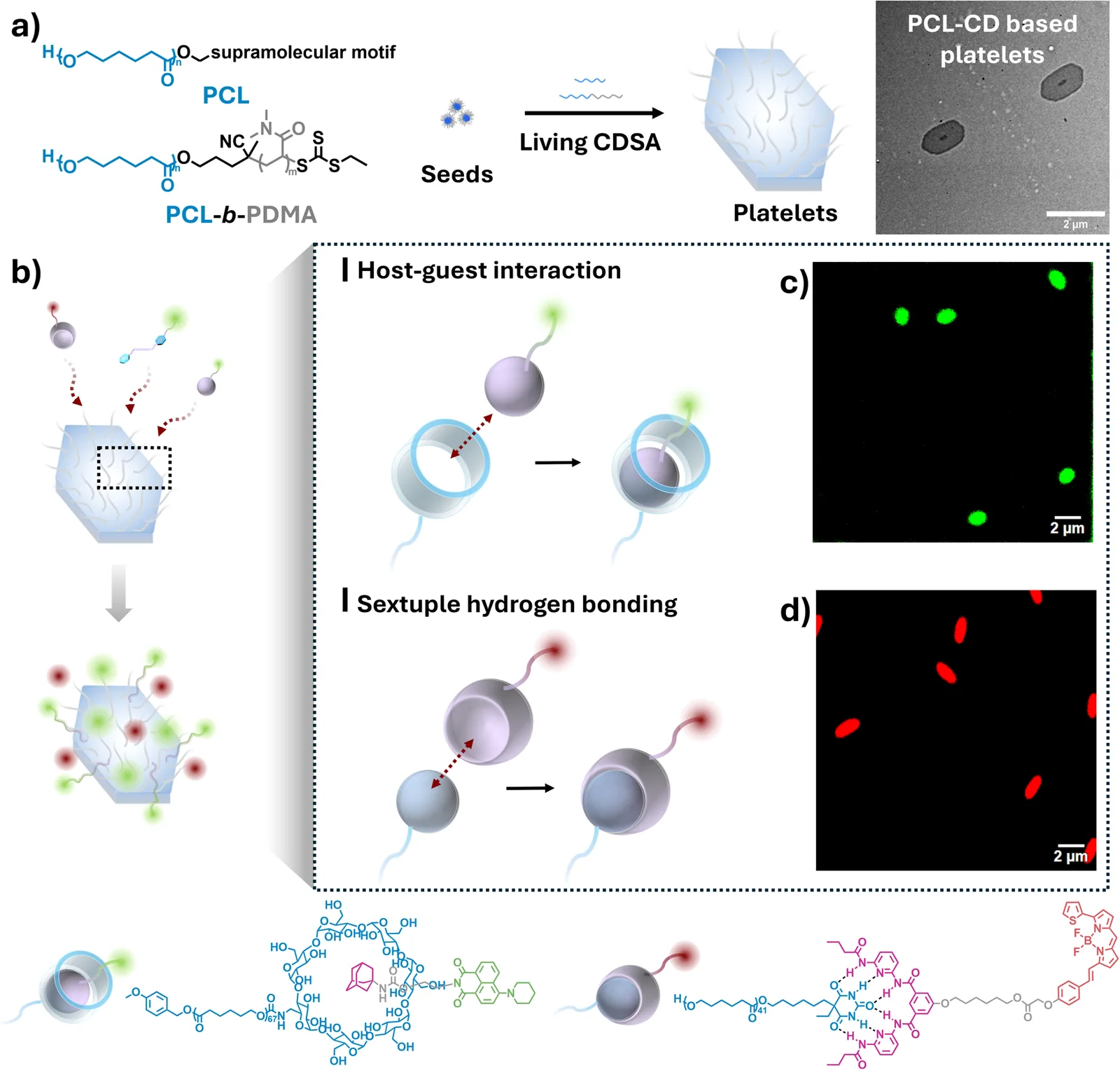
Fluorescent platelets through noncovalent surface functionalization. (a) Chemical structures of PCL HP and PCL-*b*-PDMA BCP, and schematic illustration of the preparation of supramolecularly functionalized platelets via the living CDSA method in EtOH, and a representative TEM image of PCL-CD based platelets. Scale bar: 2 μm. (b) Schematic representation of noncovalent interactions enabling targeted dye localization on polymeric platelets. Two distinct interactions are utilized: host–guest interactions between CD and Ad, and sextuple hydrogen bonding between BA and HW. (c) Confocal image of PCL-CD-based platelets mixed with NMI-Ad. (d) Confocal image of PCL-BA platelets mixed with BDP-HW. Samples were imaged at 60× magnification upon excitation using a λ_ex_ = 405 nm laser (green channel, λ_em_ = 450–550 nm) and 640 nm laser (red channel, λ_em_ = 650–750 nm). Scale bar: 2 μm.

Initially, we focused on whether the confined motifs retained their activity to mediate selective recognition and binding events. The activity of the confined supramolecular units was evaluated by mixing PCL-CD-based platelets with a dye molecule, NMI-Ad. A typical supramolecular functionalization protocol involved adding 5 μL of the small-molecule solution to 500 μL of the platelet solution, allowing the molecules to bind to the platelet surface via noncovalent interactions, followed by stirring overnight at room temperature to ensure equilibrium binding. The selective localization of dye molecules on the platelets confirmed successful host–guest interactions (Figures 1b,c and S67). To further confirm the specificity of host–guest interactions, various combinations of platelets (PCL-CD, PCL-Ad, and PCL–OH-based) and dye molecules (NMI-CD, NMI-Ad, and NMI–OH) were analyzed. Fluorescence was observed exclusively on platelets when both the dye molecules and platelets were modified with complementary supramolecular building blocks (PCL-CD and NMI-Ad, and PCL-Ad and NMI-CD), whereas other combinations exhibited nonspecific fluorescence distribution (Figure S68). Host–guest interactions within the CDSA system are highly selective and can be readily established under mild conditions.

Förster resonance energy transfer (FRET) was further employed to confirm the spatial organization and functional activity of noncovalent interactions. Platelets were coassembled from PCL-CD and boron-dipyrromethene 630/650 functionalized poly­(ε-caprolactone) (PCL-BDP) via living CDSA. Upon adding NMI-Ad, the green emissive dye NMI selectively attached to the platelets (Figure S69). Under 405 nm irradiation, FRET occurred because of the local confinement of NMI and BDP within approximately 10 nm, resulting in red emission from BDP. These results confirm the precise spatial arrangement and retained functionality of noncovalent interactions within the nanostructured assemblies. To explore the generality of this supramolecular strategy, other noncovalent interaction, multiple hydrogen-bonding interactions, was employed for the noncovalent surface-functionalization. As expected, sextuple hydrogen-bonding interaction resulted in the selective localization of red fluorescence on the platelets after mixing PCL-BA-based platelets with dye of SPY-HW or BDP-HW (Figures 1d and S70), further showcasing the versatility of supramolecular strategy in modifying CDSA assemblies. To examine the aqueous compatibility of this system, the platelets were transferred from ethanol into water and several aqueous media while retaining their structural integrity. Supramolecular surface functionalization in these aqueous media was then evaluated by combining PCL-CD or PCL-BA-based platelets with the complementary dyes NMI-Ad or BDP-HW, respectively. Platelet-localized fluorescence was observed in water, 1× phosphate-buffered saline (PBS, pH 7.3), 20 mM HEPES buffer (pH 7.5), 10 mM Tris-HCl containing 5 mM MgCl_2_ (pH 7.2), and Dulbecco’s modified Eagle’s medium (DMEM), indicating that the supramolecular interactions are retained in water, these buffered aqueous media and DMEM (Figures S71 and S72).

Having confirmed that supramolecular motifs embedded within platelets retain both specificity and functionality, we next investigated whether these interactions could be harnessed to impart dynamic and reversible behavior. In particular, we explored whether external stimuli could be used to regulate binding and release of dye molecules. In the host–guest system, we exploited the reversible nature by implementing two complementary strategies: light-responsive guest and competitive guest exchange (Figure 2a,b). As a light-controlled model system, we synthesized an azobenzene-functionalized naphthalimide (NMI-Azo) that binds to PCL-CD platelets (Figure 2c). In the thermodynamically stable *trans* configuration, NMI-Azo bound to CD, as confirmed by confocal microscopy, which showed localized fluorescence on the platelet surfaces. Upon UV irradiation, *trans–cis* isomerization of Azo significantly reduced the binding affinity, resulting in dye release and redistribution into the solution, observed as diffuse background fluorescence. Subsequent exposure to visible light regenerated the *trans* isomer, restoring CD binding and platelet-localized emission. Ultraviolet–visible (UV–vis) absorption spectroscopy corroborated the photoreversible switching, with alternating absorbance changes under UV and visible irradiation (Figure S73). Although a gradual decrease in intensity was noted after multiple cycles due to minor photooxidation, the assemblies retained reversible functionality. This phenomenon demonstrates that NMI-Azo/CD host–guest complexes can be effectively implemented on platelets, enabling reversible, light-controlled insertion and release of dye molecules without compromising the nanostructure.

**Figure 2:**
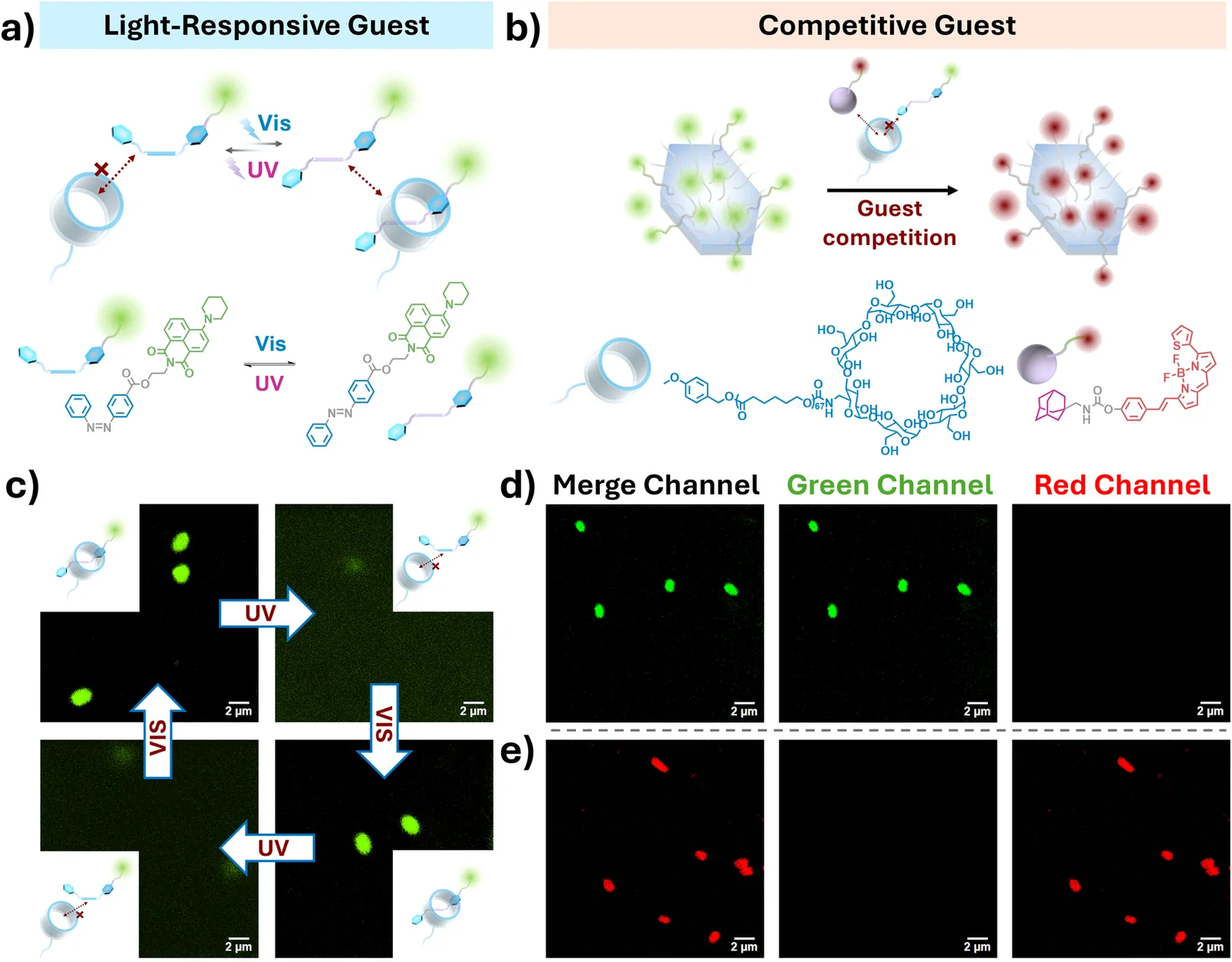
Dynamic and reversible surface-functionalization of platelets. (a) Schematic illustration of the light-responsiveness of host–guest interactions between CD and Azo. (b) Schematic illustration of the dynamics of host–guest interactions on CDSA platelets through guest competition. (**c**) Confocal fluorescence images of PCL-CD platelets mixed with NMI-Azo, sequentially irradiated with 340 nm UV and visible light. (d) Confocal images of PCL-CD platelets mixed with NMI-Azo before adding BDP-Ad. (e) Confocal images of PCL-CD platelets mixed with NMI-Azo after adding BDP-Ad. Samples were imaged at 60× magnification upon excitation using a λ_ex_ = 405 nm laser (green channel, λ_em_ = 450–550 nm) and 640 nm laser (red channel, λ_em_ = 650–750 nm). Scale bar: 2 μm.

The dynamic property of the supramolecular CDSA platelet was also investigated through competitive guest experiments. Ad and Azo are selected as competing guests because the association constant (*K*_a_) of Ad with CD (*K*_a_ = 3.0–5.0 × 10^4^ M^–1^)^48,49^ is significantly higher than that of Azo with CD (*K*_a_ = 1.7–2.4 × 10^3^ M^–1^).^50,51^ PCL-CD-based platelets preloaded with green-emissive NMI-Azo were subsequently treated with red-emissive BDP-Ad (Figure 2d). Following equilibration, guest exchange was observed: the stronger Ad–CD interaction displaced NMI-Azo,^52^ as evidenced by the loss of green fluorescence and emergence of red platelet-localized emission (Figure 2e). This dynamic exchange resembles competitive molecular recognition, providing a simple yet effective mechanism for “reprogramming” or controllable release in supramolecular assemblies. To demonstrate this approach in reversible small-molecule loading, camptothecin (CPT) have been initially loaded into PCL-CD-based platelets via host–guest interactions in a water system (Figure S74), as CPT is known to form inclusion complexes with β-cyclodextrin.^53^ Subsequent addition of adamantane competitively occupies the cyclodextrin cavity, leading to displacement and release of the loaded CPT (Figures S74 and S75).

A similar phenomenon was observed in the sextuple hydrogen-bonding system: PCL-BA-based platelets coupled with SPY-HW through multiple hydrogen bonding also exhibited dynamic behavior. The addition of trifluoroethanol (TFE) effectively disrupted the sextuple hydrogen-bonding interactions, resulting in loss of fluorescence (Figure S76). This process was further confirmed by ^1^H NMR spectroscopic analysis with the disappearance of the resonance associated with hydrogen-bonding between the host and guest at δ = 12.78 ppm after adding TFE (Figure S77). Together, these results demonstrate that CDSA nanostructures can be engineered with reversible, stimulus-responsive, and reconfigurable properties, advancing their potential as adaptive soft materials.

Building on this foundation, we sought to determine whether orthogonal noncovalent interactions could be combined within a single nanostructure to achieve higher spatial and functional complexity (Figure 3a). The Ad–CD host–guest system and HW–BA hydrogen-bonding pair are mutually noninterfering, exhibiting high orthogonality that enables both interactions to operate independently within one assembly.^45,54^ Two-layered platelets with concentric, laterally extended structures were prepared through a sequential living CDSA process involving two successive additions of supramolecular motif-functionalized unimers (PCL-CD or PCL-BA-based HP, Figure S78). In this architecture, one layer incorporated CD groups to enable host–guest interaction with Ad-labeled molecules and the other contained BA motifs capable of forming sextuple hydrogen bonding with HW-labeled species. This design allowed spatial confinement of distinct supramolecular domains, enabling selective and independent functionalization of each layer. When a mixture of two complementary dyes, green-emissive NMI-Ad and red-emissive BDP-HW, was added to the solution of two layered platelets, confocal microscopy revealed distinct spatial segregation, with each dye selectively localizing within its corresponding supramolecular layer. NMI-Ad localized exclusively to one layer, while BDP-HW bound only to the complementary domain, with no detectable cross-binding or interference between the two systems (Figures 3b,c and S79). This precise spatial recognition highlights the robustness of orthogonal design. The ability to direct different supramolecular motifs into discrete regions of the same nanostructure provides a powerful strategy for constructing hierarchically functionalized assemblies. Such region-selective precise functionalization enables multiplexed chemical encoding, spatially resolved signal integration, and the modular combination of independent functions within a single architecture.

**Figure 3:**
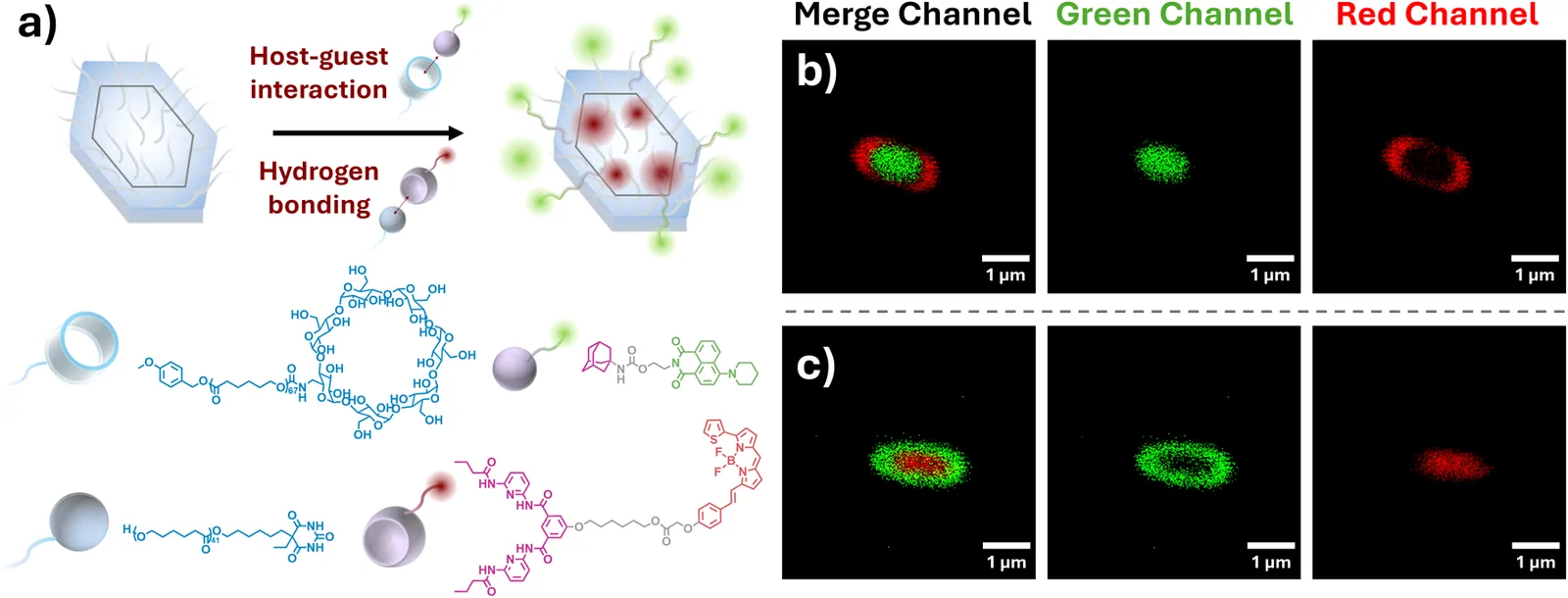
Selective surface-functionalization of platelets. (a) Schematic illustration and (b, c) Confocal images showing selective surface-functionalization of platelets, where host–guest and sextuple hydrogen-bonding interactions enable spatially selective localization of NMI-Ad and BDP-HW dyes within distinct platelet layers. Samples were imaged at 60× magnification upon excitation using a λ_ex_ = 405 nm laser (green channel, λ_em_ = 450–550 nm) and 640 nm laser (red channel, λ_em_ = 650–750 nm). Scale bar: 1 μm.

Having demonstrated the feasibility of region-selective functionalization, we next sought to extend this design to dynamic control. Specifically, we aimed to establish a sequential and controllable “wipe-clean” process, in which bound molecules could be selectively erased in response to tailored stimuli (Figure 4a). As a model system, we employed double-layered platelets in which host–guest and sextuple hydrogen-bonding interactions were confined to distinct layers. This spatial segregation enabled independent functionalization of each layer with fluorophores: NMI-Azo bound to the CD domain, giving green emission, while BDP-HW attached to the BA domain, producing red emission (Status 1, Figure 4b). Targeted erasure was achieved by introducing specific competitive stimuli. The addition of Ad led to selective displacement of NMI-Azo from the CD domain, resulting in complete loss of the green signal while leaving the red fluorescence intact (Status 2, Figure 4c). Subsequent treatment with TFE, a hydrogen-bonding disruptor, cleaved the BA-HW interaction, leading to release of BDP-HW and disappearance of the red signal (Status 3, Figure 4d). Thus, the sequential application of orthogonal triggers enabled stepwise “wiping” of distinct layers, restoring the platelets to a blank state. Conversely, the sequence of erasure can also be reversed, first disrupting the hydrogen bonding followed by cleavage of the host–guest complexes, demonstrating the programmability and bidirectional control of the “wipe-clean” process (Figure S80). This programmable release process exemplifies how supramolecular orthogonality and dynamics can be translated into functional reset mechanisms. Such “wipe-clean” nanostructures establish a conceptual basis for rewritable soft and optical materials, dynamic molecular barcodes, and adaptive information storage platforms.

**Figure 4:**
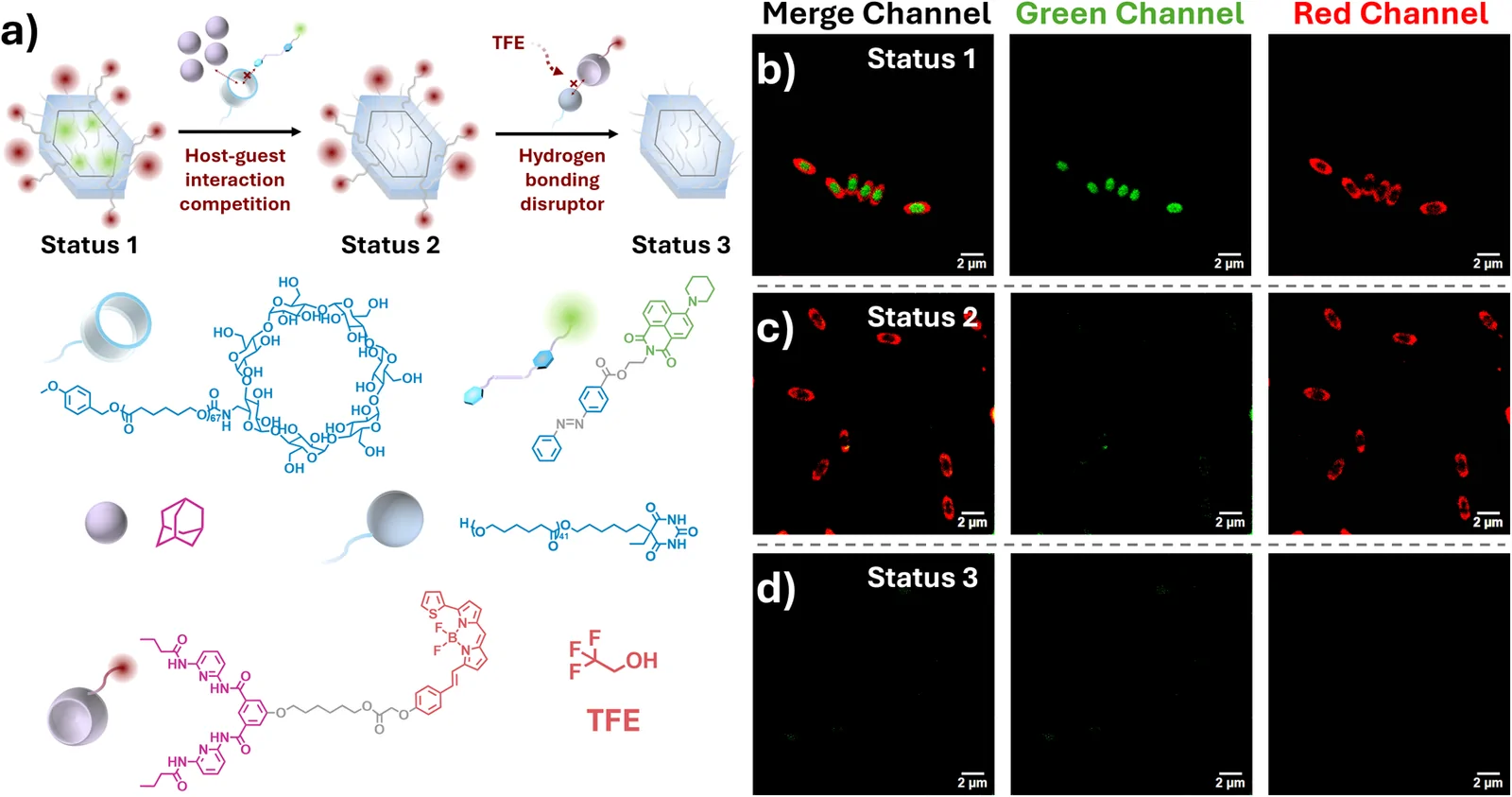
Controllable “wipe-clean” functionality of platelets. (a) Schematic illustration of sequential fluorescence erasure: initial double-layered platelets functionalized via host–guest (green) and sextuple hydrogen-bonding (red) interactions (Status 1); addition of Ad displaces NMI-Azo from CD sites, selectively removing green fluorescence (Status 2); subsequent treatment with TFE disrupts hydrogen-bonding, erasing red fluorescence and returning platelets to a blank state (Status 3). (b–d) Confocal images corresponding to Status 1–3, demonstrating stepwise, programmable removal of supramolecular signals. Samples were imaged at 60× magnification upon excitation using a λ_ex_ = 405 nm laser (green channel, λ_em_ = 450–550 nm) and 640 nm laser (red channel, λ_em_ = 650–750 nm). Scale bar: 2 μm.

## Conclusion

In summary, we report a supramolecular CDSA strategy that unites the structural fidelity of CDSA with the reversibility of noncovalent interactions. By embedding host–guest and hydrogen-bonding motifs into polymeric platelets, this approach enables selective/orthogonal molecular recognition, reversible change, and sequential erasure under mild conditions. Unlike covalent methods, which require specialized reagents and multistep protocols, the supramolecular CDSA strategy offers an operationally simple and modular platform for introducing dynamic and reconfigurable functionality. These results establish supramolecular CDSA as a versatile framework for engineering adaptive polymeric materials, with implications for molecular encoding, responsive loading and release, and the development of reconfigurable devices that enable programmable and reconfigurable soft materials.

## Supplementary Material



## Notes

Materials, characterization, instrumentation, methods, experimental procedures, and additional data (NMR, Mass spectroscopy, FT-IR, SEC, TEM, AFM, HPLC, and Confocal images) (PDF)
