# Supplementary material for: Dynamically and Reversibly Functionalized Self-Assembled Platelets via Noncovalent Interactions
Source: J Am Chem Soc. 2026 Oct 29;148(44):48572–9. doi: 10.1021/jacs.6c17888 (PMC13761275; doi:10.1021/jacs.6c17888)
Supplement: Supplementary file 1 [file ja6c17888_si_001.pdf]

Supporting Information for

Dynamically and Reversibly Functionalized Self-Assembled Platelets via Noncovalent Interactions

Tianlai Xia,[1#] Peng Sun,[1,2#] Laihui Xiao,[1] Andrew P. Dove,[1*] Rachel K. O'Reilly[1*]

[1]School of Chemistry, University of Birmingham, Edgbaston, Birmingham, B15 2TT, U.K.

[2]School of Interdisciplinary Science, Beijing Institute of Technology, Beijing, 100081, China

*Corresponding Author: a.dove@bham.ac.uk (APD) and r.oreilly@bham.ac.uk (ROR)

[#]T.X. and P.S. contributed equally to this work.

Table of Contents

Materials 4

Characterization and Instrumentation 6

Nuclear magnetic resonance (NMR) 6

Electrospray ionization mass spectrometry (ESI-MS) 6

Fourier transform infrared (FT-IR) 6

Size exclusion chromatography (SEC) 6

Transmission electron microscopy (TEM) 6

Atomic Force Microscopy (AFM) 7

Confocal laser scanning microscopy (CLSM) 7

Ultraviolet–visible (UV–vis) absorption spectroscopy 7

High performance liquid chromatography (HPLC) 7

Synthesis Method 8

Synthesis of PCL_{50}-CTA 8

Synthesis of PCL_{50}-*b*-$PDMA_{193}$ 8

Synthesis of PCL_{67}-OH 9

Synthesis of PCL_{67}-CD 10

Synthesis of Ad-C3-OH 11

Synthesis of PCL_{54}-Ad 11

Synthesis of BA-OH 12

Synthesis of PCL_{41}-BA 13

Synthesis of HW-OH 14

Synthesis of PCL_{75}-HW 16

Synthesis of NMI-OH 16

Synthesis of NMI-Ad 17

Synthesis of NMI-CD 18

Synthesis of SPY-HW 19

Synthesis of BDP-HW 20

Synthesis of NMI-Azo 21

Synthesis of BDP-Ad 21

Preparation of seed solutions 22

Epitaxial growth of platelets 22

Epitaxial growth of multi-layered platelets 23

Supramolecular interaction protocol of CDSA assemblies and small molecules 23

Transfer platelets into different buffer solutions 23

Separation procedure of platelets and free molecules 24

Supplementary Figures and Tables 25

NMR spectra 25

Mass spectrometry 43

FT-IR spectra 49

Size exclusion chromatogram 55

Polymer information table 61

Living CDSA and supramolecular interaction 62

Materials

4-Methoxybenzyl alcohol (98%, Sigma-Aldrich), 1-adamantanecarboxylic acid (Ad-COOH, 99%, ACROS Organics), 1-adamantanemethylamine (Ad-NH_2, 98%, Sigma-Aldrich), 1-adamantylmethanol (Ad-OH, 98%, Thermo Scientific), sulfuryl chloride solution (1.0 M in methylene chloride, Sigma-Aldrich), 3-amino-1-propanol (99%, Merck), 4-nitrophenyl chloroformate (96%, Sigma-Aldrich), 6-bromo-1-hexanol (97%, Sigma-Aldrich), diethyl ethylmalonate (97%, Sigma-Aldrich), urea (99%, Sigma-Aldrich), 2,6-diaminopyridine (98%, Sigma-Aldrich), butyryl chloride (98%, Sigma-Aldrich), thionyl chloride ($SOCl_2$, 99%, Sigma-Aldrich), 4-bromo-1,8-naphthalic anhydride (95%, Sigma-Aldrich), ethanolamine (98%, Sigma-Aldrich), piperidine (99%, Sigma-Aldrich), triethylamine (NEt_3, 99%, Sigma-Aldrich), methacryloyl chloride (97%, contains monomethyl ether hydroquinone as stabilizer, Sigma-Aldrich), 1-adamantylamine (98%, Alfa Aesar), 4-(phenylazo)benzoic acid (98%, Sigma-Aldrich), N-(3-dimethylaminopropyl)-N'-ethylcarbodiimide hydrochloride (EDC·HCl, Sigma-Aldrich), 4-(dimethylamino)pyridine (DMAP, 98%, Sigma-Aldrich), sodium hydride (NaH, 60% dispersion in mineral oil, Sigma-Aldrich), 5-hydroxyisophthalic acid (97%, Sigma-Aldrich), succinic anhydride (99%, Sigma-Aldrich), 4-phenylazophenol (98%, Sigma-Aldrich), 6-monodeoxy-6-monoamino-β-cyclodextrin (β-CD-NH_2, 99%, Aladdin), boron-dipyrromethene 630/650 carboxylic acid (BDP-630/650-COOH, 95%, Lumiprobe), diphenyl phosphate (DPP, 99%, Sigma-Aldrich), ε-caprolactone (CL, 99%, ACROS Organics), 2,2'-azobis(2-methylpropionitrile) (AIBN, 98%, Sigma-Aldrich), N,N-dimethylacrylamide (DMA, 99%, 500 ppm monomethyl ether hydroquinone, Sigma-Aldrich), trifluoroethanol (TFE, 99%, Sigma-Aldrich), deuterated chloroform ($CDCl_3$, 99.8 atom % D, Apollo Scientific), deuterated dimethyl sulfoxide (DMSO-d_6, 99.8 atom % D, Apollo Scientific), phosphorus pentoxide (P_2O_5, 99%, Sigma-Aldrich), calcium hydride (CaH_2, 95%, Sigma-Aldrich), ammonium chloride (NH_4Cl, 99.5%, Sigma-Aldrich), anhydrous sodium sulfate (Na_2SO_4, 99%, Sigma-Aldrich), sodium bicarbonate ($NaHCO_3$, 99.7%, Sigma-Aldrich), potassium carbonate (K_2CO_3, 99%, Sigma-Aldrich), ammonium fluoroborate (NH_4BF_4, 97%, Sigma-Aldrich), basic alumina (standard grade, Sigma-Aldrich), hydrochloric acid (HCl, 37%, Sigma-Aldrich), camptothecin (CPT, ≥90% (HPLC), Fisher), anhydrous 1,4-dioxane (99.8%, ≤ 25 ppm butylated hydroxytoluene, Sigma-Aldrich), dichloromethane (CH_2Cl_2, 99.8%, Fisher), N,N-dimethylformamide (DMF, 99.5%, Fisher), ethyl acetate (EA, 99%, VWR), tetrahydrofuran (THF, 99.5%, Fisher), methanol (MeOH, 99.8%, Fisher), ethanol (EtOH, 99.8%, Fisher), anhydrous diethyl ether (Et_2O, 99%, Fisher), toluene (analytical reagent grade, Fisher),

chloroform ($CHCl_3$, 99.8%, Honeywell), acetone (99%, Fisher), and n-hexane (95%, Fisher) were purchased from commercial suppliers. The dual-headed chain transfer agent (CTA) 2-cyano-5-hydroxypentan-2-yl ethyl carbonotrithioate (CHPET) and hydroxy-substituted spiropyran (SPY-OH) were synthesized according to a previously reported procedure.[1-3] 1× phosphate-buffered saline (PBS, pH 7.3) was prepared from PBS tablets (Oxoid, Dulbecco A, Ref. BR0014G). HEPES buffer (20 mM, pH 7.5) was prepared using 4-(2-hydroxyethyl)piperazine-1-ethanesulfonic acid (HEPES) purchased from Sigma-Aldrich (Cat. No. H3375-500G). Tris-HCl buffer containing $MgCl_2$ (10 mM Tris-HCl, 5 mM $MgCl_2$, pH 7.2) was prepared using tris(hydroxymethyl)aminomethane (Fisher Scientific, Code T/3710/60) and magnesium chloride hexahydrate (Sigma-Aldrich, Cat. No. M9272). Dulbecco's Modified Eagle's Medium, high glucose (DMEM; Sigma-Aldrich, Cat. No. D5796-500ML), was used as received.

All chemicals and solvents were used as received unless otherwise stated. CHPET and DPP were dried over P_2O_5 under static vacuum in a desiccator for one week prior to use. CL was vacuum distilled twice over CaH_2, then stored and used in a nitrogen-filled glovebox. AIBN was recrystallized twice from MeOH and stored at 4 °C in the dark. Anhydrous 1,4-dioxane and DMA were passed through a plug of basic alumina immediately before use to remove inhibitors. All dry solvents were dispensed from an in-house solvent drying/degassing tower under inert gas.

Characterization and Instrumentation

Nuclear magnetic resonance (NMR)

All NMR spectra were recorded on a Bruker 400 MHz (DPX-400) spectrometer. Chemical shifts (δ) are reported in parts per million (ppm). Samples were prepared in deuterated solvents and referenced to residual non-deuterated solvent signals ($CDCl_3$ @ 7.26 ppm ^{1}H NMR; $CDCl_3$ @ 77.2 ppm ^{13}C NMR; DMSO-d_6 @ 2.50 ppm ^{1}H NMR; DMSO-d_6 @ 39.5 ppm ^{13}C NMR). The resonance multiplicities are described as s (singlet), d (doublet), t (triplet), q (quartet), or m (multiplet).

Electrospray ionization mass spectrometry (ESI-MS)

High-resolution electrospray ionization mass spectrometry (HR-ESI-MS) was performed on a Waters Synapt G2-S mass spectrometer equipped with an Advion NanoMate nanospray ionization source.

Fourier transform infrared (FT-IR)

Fourier transform infrared spectroscopy was carried out using a PerkinElmer UATR Two Fourier transform infrared spectrometer. A total of 16 scans from 400 to 4000 cm^{-1} were taken at a resolution of 2 cm^{-1}, and the spectra were corrected for background absorbance.

Size exclusion chromatography (SEC)

SEC analysis was performed on an Agilent 1260 Infinity II system fitted with differential refractive index (RI) and 309 nm ultraviolet (UV) detectors, equipped with a PLGel 3 µm (50 × 7.5 mm) guard column and two PLGel 5 µm (300 × 7.5 mm) mixed-D columns, with $CHCl_3$ containing 0.5% NEt_3 or DMF containing 5 mM NH_4BF_4 as the mobile phase. Molecular weight (number-average molecular weight (M_n) and weight-average molecular weight (M_w)) and molecular weight distributions ($Đ_M$ = M_w/M_n) were calibrated against poly(methyl methacrylate) (PMMA) standards and analyzed using Agilent SEC software.

Transmission electron microscopy (TEM)

Dry state TEM imaging was performed using a JEOL 1400 Bio (1720/GB06) transmission electron microscope at an acceleration voltage of 80 kV. Crystallization-driven self-assembly (CDSA) samples were deposited onto graphene oxide-coated copper grids. The grid was then dried, and analyzed.

Atomic Force Microscopy (AFM)

AFM imaging samples were prepared by depositing approximately 10–20 μL of the assemblies solution onto a clean cover slip, followed by spin coating to ensure complete drying. Prior to preparing the samples, the cover slips and pipette tips used for handling the solution were thoroughly cleaned with ethanol to ensure cleanliness and prevent contamination. Imaging and analysis were performed on a JPK Nanowizard 4 system in quantitative imaging (QI) mode. The tips for the AFM analysis (PPP-NCHAuD) were purchased from NANOSENSORSTM, with resonance frequency (kHz) in the window of 204–497 and force constant in the range of 10–130. The AFM pictures and data were analysed and collected using the JPK Data Processing software with QI mode.

Confocal laser scanning microscopy (CLSM)

FV3000 (Olympus) confocal microscope with an IX-81 inverted base (Olympus) and the 20× and 60× oil-immersion objectives (Olympus) was used for imaging. The FV3000 system was driven with the FV31S-SW Viewer software platform (Olympus) with a scan rate of 1 μs pixel^{-1}; images were acquired at 512 × 512 to 1024 × 1024 pixels. Samples were imaged at 60× magnification upon excitation using a λ_{ex} = 405 nm laser (blue channel, λ_{em} = 410–510 nm), λ_{ex} = 405 nm laser (green channel, λ_{em} = 450–550 nm), and 640 nm laser (red channel, λ_{em} = 650–750 nm). Fluorescence images were processed using CellSens (Olympus) and ImageJ image processing software. Samples were prepared by spin-coating 10–20 μL of sample solution on coverslip, the spin program was 3000 rpm for 50 seconds and 5000 rpm for 10 seconds.

Ultraviolet–visible (UV–vis) absorption spectroscopy

UV–vis spectroscopy was carried out on an Agilent Cary 3500 UV–vis Spectrometer at 20 °C, wavelength range of 250–800 nm.

High performance liquid chromatography (HPLC)

HPLC measurements were performed using modular Shimadzu systems equipped with combinations of LC-20AD pumps, SIL-20AC HT autosamplers, CTO-20AC column ovens, CBM-20A communication modules, SPD-M20A photodiode array (PDA) detectors, and RF-20A fluorescence detectors. Degassing was performed using a DGU-20A5R degassing unit. Reverse-phase HPLC measurements were typically conducted using water/acetonitrile solvent systems containing 0.1 vol% formic acid. CPT analysis was monitored at 359 nm using the PDA detector.

Synthesis Method

Synthesis of PCL_{50}-CTA

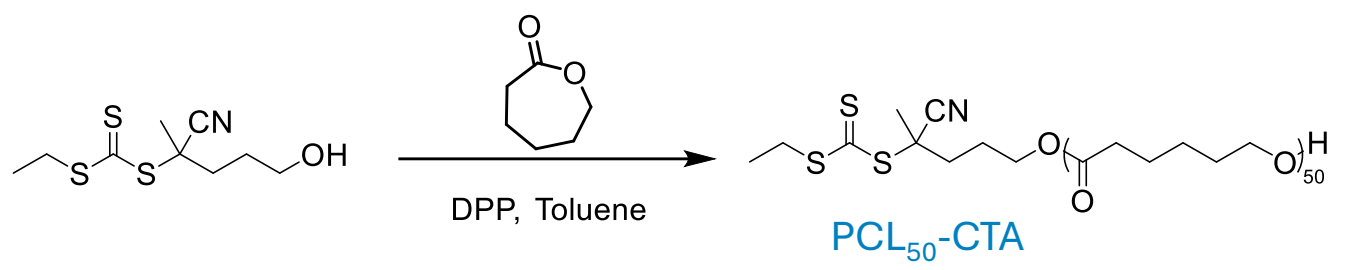

In a nitrogen-filled glove box (oxygen and water content < 0.1 ppm), a solution of DPP (50.2 mg, 0.201 mmol) in dry toluene (5 mL) and a solution of CHPET CTA (50.0 mg, 0.201 mmol) in dry toluene (7 mL) were added to CL (1601.1 mg, 14.033 mmol) dissolved in dry toluene (7 mL). The mixture was stirred at room temperature for 7 hours. After the reaction, the solution was removed from the glovebox and precipitated dropwise into cold Et_2O (3 times). The resulting polymer PCL_{50}-CTA was collected by centrifugation and dried under vacuum. The final product was characterized by NMR (**Figure S1** and **S2**) and SEC (**Figure S58** and **Table S1**).

^{1}H NMR (400 MHz, $CDCl_3$, 298 K) δ (ppm): 4.12 (t, 2H), 4.05 (t, 100 H), 3.64 (t, 2H), 3.33 (q, 2H), 2.30 (t, 100H), 1.88 (s, 3H), 1.72–1.54 (m, 209H), 1.45–1.28 (m, 104H), 1.25 (s, 3H). (**Figure S1**)

^{13}C NMR (101 MHz, $CDCl_3$, 298 K) δ (ppm): 217.3, 173.7, 119.4, 64.3, 63.2, 62.8, 46.9, 35.9, 34.3, 32.5, 31.5, 28.5, 25.7, 25.5, 24.7, 12.9. (**Figure S2**)

The DP of PCL-CTA was determined from the ^{1}H NMR spectrum (**Figure S1**) by comparing the integral of the methylene protons adjacent to the carbonyl group in the PCL repeat unit (–$\boldsymbol{CH_2}$–CO–, δ ≈ 2.30 ppm, peak g) with that of the characteristic end-group protons derived from the CTA moiety (–S$\boldsymbol{CH_2}$–, δ ≈ 3.33 ppm, peak b). Based on the ratio of these integrals, the DP was calculated to be approximately 50.

PCL_{50}-CTA. ^{1}H NMR (400 MHz, $CDCl_3$): $M_{n,NMR}$ = 6.0 kg mol^{-1}. SEC (Chloroform, PMMA standard): $M_{n,GPC}$ = 12.0 kg mol^{-1}, $Đ_M$ = 1.07. (**Figure S58**)

Synthesis of PCL_{50}-*b*-$PDMA_{193}$

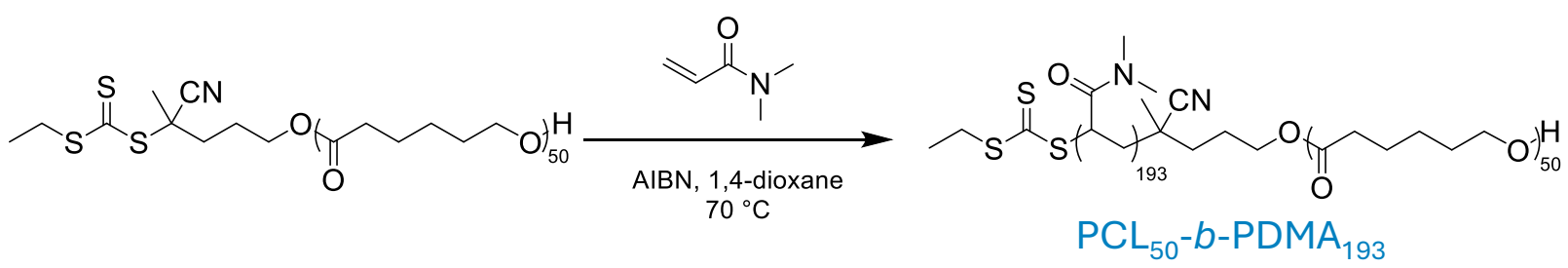

PCL_{50}-CTA (100.0 mg, 16 μmol), DMA (408.3 mg, 4.118 mmol) and AIBN (0.32 mg, 2 μmol) were dissolved in 1,4-dioxane (1 mL) and placed in an ampoule. The solution was subjected to nitrogen bubbling for 20 minutes and then heated for 2 hours at 70°C. The reaction was

quenched by immersion of the ampoule in liquid nitrogen, and the poly(ε-caprolactone)-*block*-poly(N,N-dimethylacrylamide) (PCL-*b*-PDMA) was precipitated in cold Et_2O (3 times) before being dried under vacuum, and then analysed by NMR (**Figure S3** and **S4**) and SEC (**Figure S58** and **Table S1**).

^{1}H NMR (400 MHz, $CDCl_3$, 298 K) δ (ppm): 4.06 (t, 102 H), 3.32–2.72 (m, 1158 H), 2.31 (t, 100 H), 1.62 (m, 206H), 1.38 (m, 102H). (**Figure S3**)

^{13}C NMR (101 MHz, $CDCl_3$, 298 K) δ (ppm): 174.9, 174.8, 174.6, 173.7, 172.7, 64.3, 62.7, 37.6, 37.2, 36.4, 35.9, 35.8, 34.2, 32.4, 28.5, 25.6, 24.7, 20.9. (**Figure S4**)

The DP of the PDMA blocks in PCL_{50}-*b*-PDMA was determined from the ^{1}H NMR spectrum (**Figure S3**) by comparing the integral of the methylene protons adjacent to the carbonyl group in the PCL repeat unit (–***CH₂***–CO–, δ ≈ 2.31 ppm, peak c) with that of the characteristic protons of the PDMA block (–N$(CH_3)_2$, δ ≈ 3.47–2.43 ppm, peaks a + b). Based on the ratio of these integrals, the DP values of the PDMA blocks were calculated to be approximately 193.

PCL_{50}-*b*-$PDMA_{193}$. ^{1}H NMR (400 MHz, $CDCl_3$): $M_{n,NMR}$ = 25.1 kg mol^{-1}. SEC (Chloroform, PMMA standard): $M_{n,GPC}$ = 30.8 kg mol^{-1}, $Đ_M$ = 1.12. (**Figure S58**)

Synthesis of PCL_{67}-OH

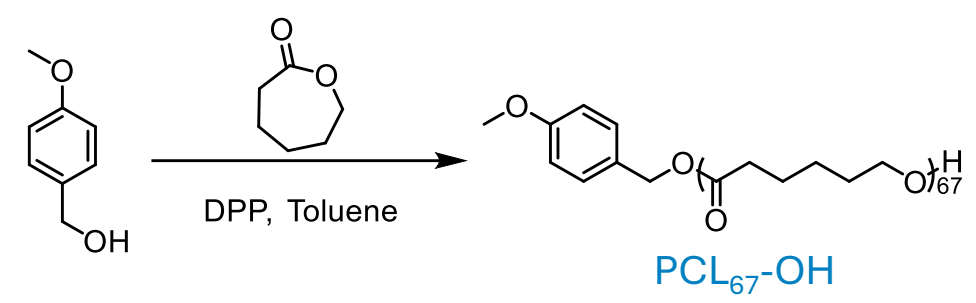

In a nitrogen-filled glove box (oxygen and water content < 0.1 ppm), a solution of DPP (153.4 mg, 0.613 mmol) in dry toluene (10 mL) and a solution of 4-methoxybenzyl alcohol (84.7 mg, 0.613 mmol) in dry toluene (15 mL) were added to CL (7000.0 mg, 61.328 mmol) dissolved in dry toluene (15 mL). The mixture was stirred at room temperature for 7 hours. After the reaction, the solution was removed from the glovebox and precipitated dropwise into cold Et_2O (3 times). The resulting polymer PCL_{67}-OH was collected by centrifugation and dried under vacuum. The final product was characterized by NMR (**Figure S5** and **S6**) and SEC (**Figure S59** and **Table S1**).

^{1}H NMR (400 MHz, $CDCl_3$, 298 K) δ (ppm): 7.28 (d, 2H), 6.87 (d, 2H), 5.02 (s, 2H), 4.04 (t, 134H), 3.79 (s, 3H), 3.62 (t, 2H), 2.28 (t, 134H), 1.63 (m, 270H), 1.36 (m, 134H). (**Figure S5**)

^{13}C NMR (101 MHz, $CDCl_3$, 298 K) δ (ppm): 173.7, 130.2, 114.1, 66.1, 64.3, 62.7, 55.4, 34.3, 32.5, 28.5, 25.7, 24.7. (**Figure S6**)

The DP of PCL-OH was determined from the ^{1}H NMR spectrum (**Figure S5**) by comparing the integral of the methylene protons adjacent to the carbonyl group in the PCL repeat unit (–***CH₂***–CO–, δ ≈ 2.28 ppm, peak e) with that of the aromatic protons of the initiator moiety (δ ≈ 6.87 ppm, peak b). Based on the ratio of these integrals, the DP was calculated to be approximately 67.

PCL_{67}-OH. ^{1}H NMR (400 MHz, $CDCl_3$): $M_{n,NMR}$ = 7.8 kg mol^{-1}. SEC (Chloroform, PMMA standard): $M_{n,GPC}$ = 16.1 kg mol^{-1}, $Đ_M$ = 1.08. SEC (DMF, PMMA standard): $M_{n,GPC}$ = 12.8 kg mol^{-1}, $Đ_M$ = 1.09. (**Figure S59**)

Synthesis of PCL_{67}-CD

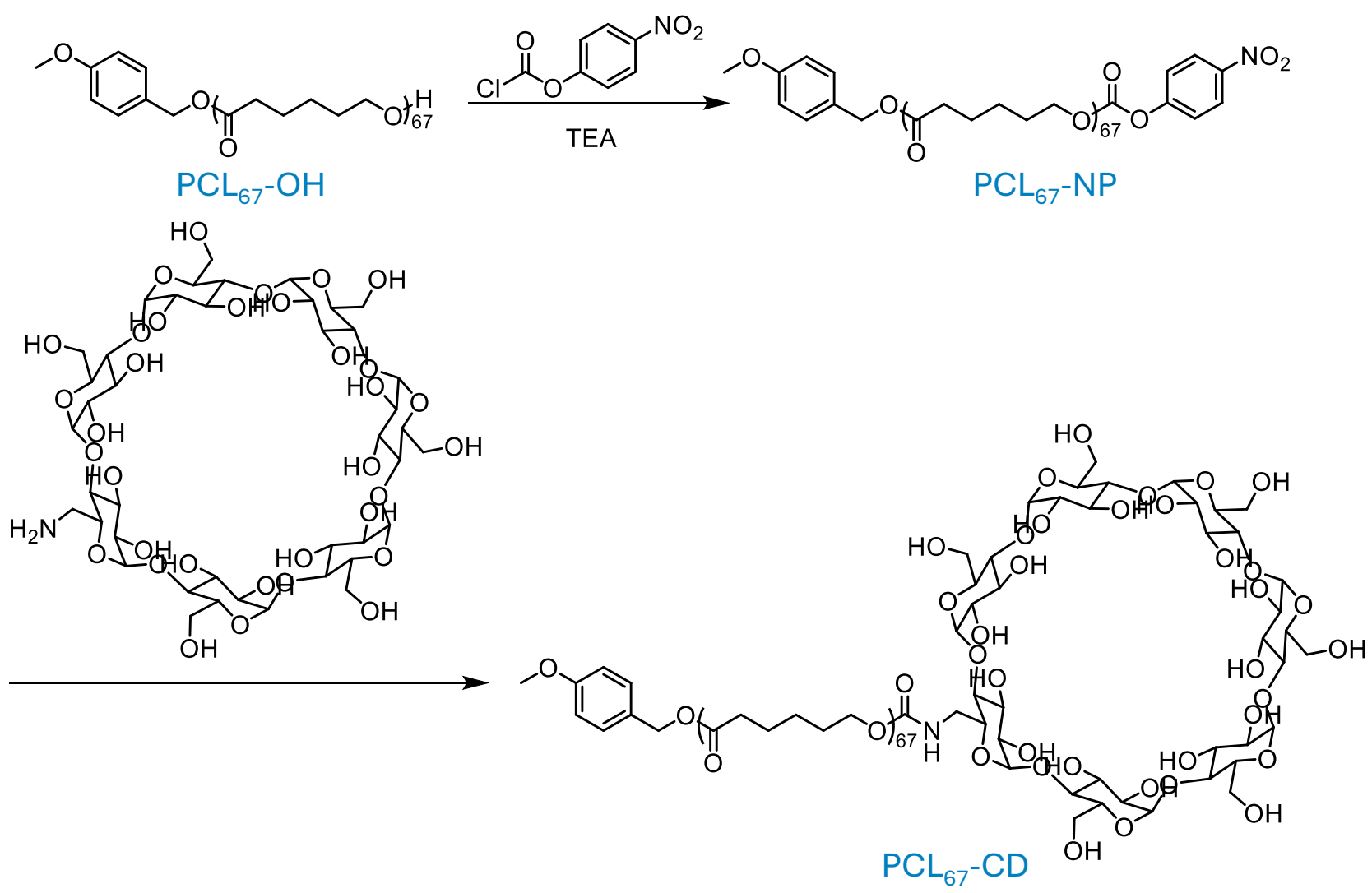

PCL_{67}-OH (0.10 g, 14 μmol), 4-nitrophenyl chloroformate (25.0 mg, 0.13 mmol), and NEt_3 (14.5 mg, 0.14 mmol) were dissolved in CH_2Cl_2 (2 mL). The reaction mixture was stirred at room temperature for 1 hour. After completion, the product was purified by precipitation into cold Et_2O (3 times) to afford PCL_{67}-NP.

^{1}H NMR (400 MHz, $CDCl_3$, 298 K) δ (ppm): 8.20 (m, 2H), 7.33 (m, 2H), 7.22 (m, 2H), 6.83 (m, 2H), 4.98 (s, 2H), 3.99 (t, 143H), 3.74 (s, 3H), 2.24 (t, 135H), 1.69–1.51 (m, 292H), 1.40–1.24 (m, 135H).

PCL_{67}-NP (0.10 g, 14 μmol), β-CD-NH_2 (15.0 mg, 0.013 mmol), and NEt_3 (14.5 mg, 0.14 mmol) were dissolved in DMF (2 mL). The reaction mixture was stirred at room temperature for 24 hours. The crude product was then purified by precipitation into cold Et_2O (3 times) to afford PCL_{67}-CD. The final product was characterized by NMR (**Figure S7**) and SEC (**Figure S60** and **Table S1**).

^{1}H NMR (400 MHz, DMSO-d$_6$, 298 K) δ (ppm): 7.29 (m, 2H), 6.90 (m, 2H), 5.04 (s, 2H), 4.05 (t, 135H), 3.80 (s, 3H), 2.30 (t, 138H), 1.72–1.54 (m, 286H), 1.45–1.28 (m, 138H). (**Figure S7**)

PCL$_{67}$-CD. ^{1}H NMR (400 MHz, DMSO-d$_6$): $M_{n,NMR}$ = 8.9 kg mol^{-1}. SEC (Chloroform, PMMA standard): $M_{n,GPC}$ = 16.4 kg mol^{-1}, $Đ_M$ = 1.09. SEC (DMF, PMMA standard): $M_{n,GPC}$ = 15.5 kg mol^{-1}, $Đ_M$ = 1.13. (**Figure S60**)

Synthesis of Ad-C3-OH

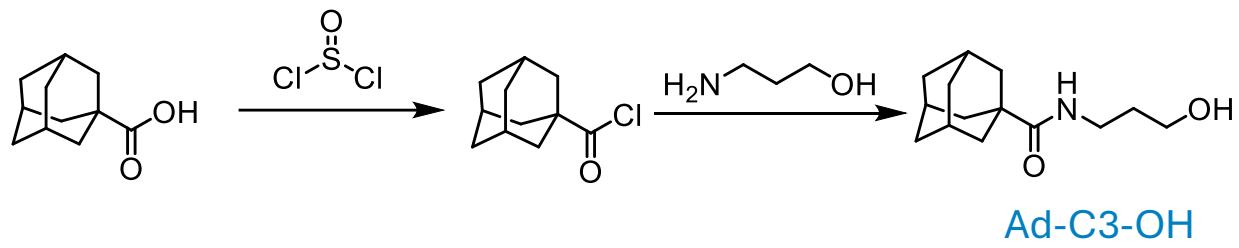

Ad-COOH (1.8 g, 10 mmol) was dissolved in $SOCl_2$ (6 mL) and the mixture was heated at 40 °C for 4 hours. After the reaction, the excess $SOCl_2$ was removed by rotary evaporation under reduced pressure. The resulting 1-adamantanecarbonyl chloride was dissolved in CH_2Cl_2 (10 mL). Separately, a solution of 3-amino-1-propanol (0.9 g, 12 mmol) and pyridine (0.8 g, 10 mmol) in CH_2Cl_2 (5 mL) was prepared. This solution was added dropwise to the CH_2Cl_2 solution of 1-adamantanecarbonyl chloride at room temperature. The reaction mixture was stirred at room temperature for 1 hour, then quenched with saturated NH_4Cl solution (30 mL). The organic layer was separated, dried over anhydrous Na_2SO_4, filtered, and concentrated under reduced pressure. The crude product was purified by column chromatography using EA/n-hexane (1:3, v/v) as the eluent to afford the desired product Ad-C3-OH (0.6 g, 25% yield).
^{1}H NMR (400 MHz, $CDCl_3$, 298 K) δ (ppm): 6.13 (s, 1H), 3.67 (s, 1H), 3.57 (t, 2H), 3.39 (q, 2H), 2.03 (s, 3H), 1.84 (s, 6H), 1.71 (m, 8H). (**Figure S8**)
^{13}C NMR (101 MHz, $CDCl_3$, 298 K) δ (ppm): 179.7, 59.2, 40.7, 39.4, 36.6, 36.1, 32.4, 28.2. (**Figure S9**)
ESI-MS (*m/z*) calculated for $[M+H]^+$: 238.1802, found: 238.1839. (**Figure S36**)
FT-IR υ (cm^{-1}): 2902, 2848, 1626, 1526, 1450, 1343, 1279, 1186, 1072, 976, 948, 673, 641, 484, 439. (**Figure S47**)

Synthesis of PCL$_{54}$-Ad

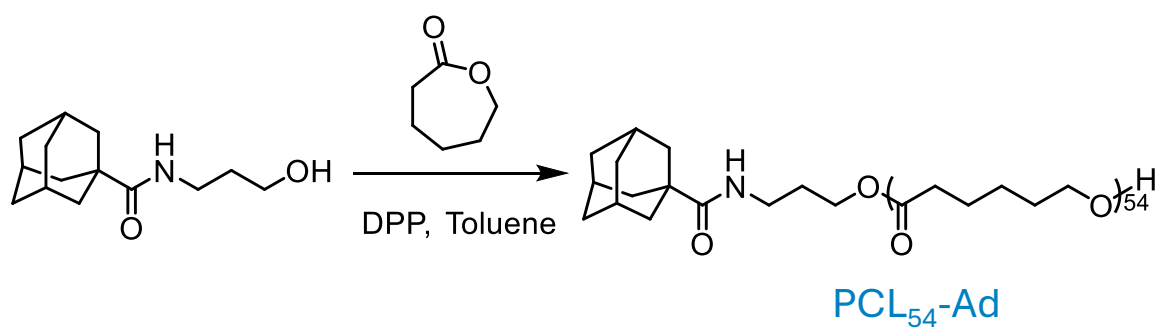

In a nitrogen-filled glove box (oxygen and water content < 0.1 ppm), a solution of DPP (36.9 mg, 0.148 mmol) in dry toluene (3 mL) and a solution of Ad-C3-OH (35.0 mg, 0.148 mmol) in dry toluene (3 mL) were added to CL (1684.4 mg, 14.757 mmol) dissolved in dry toluene (4 mL). The mixture was stirred at room temperature for 7 hours. After the reaction, the solution was removed from the glove box and precipitated dropwise into cold Et_2O (3 times). The resulting polymer was collected by centrifugation, dried under vacuum, and analyzed by NMR (**Figure S10** and **S11**) and SEC (**Figure S62** and **Table S1**).

^{1}H NMR (400 MHz, $CDCl_3$, 298 K) δ (ppm): 4.05 (t, 106H), 3.64 (t, 2H), 3.28 (q, 2H), 2.29 (t, 108H), 2.03 (m, 3H), 1.84 (m, 6H), 1.72–1.54 (m, 219H), 1.45–1.28 (m, 108H). (**Figure S10**)

^{13}C NMR (101 MHz, $CDCl_3$, 298 K) δ (ppm): 173.7, 64.3, 62.8, 39.4, 36.7, 36.2, 34.4, 34.3, 32.5, 28.5, 28.3, 25.7, 24.7. (**Figure S11**)

The DP of PCL-Ad was determined from the ^{1}H NMR spectrum (**Figure S10**) by comparing the integral of the methylene protons adjacent to the carbonyl group in the PCL repeat unit (–***CH_2***–CO–, δ ≈ 2.29 ppm, peak e) with that of the characteristic protons of the adamantane end-group (δ ≈ 2.03 ppm, peak a). Based on the ratio of these integrals, the DP was calculated to be approximately 54.

PCL_{54}-Ad. ^{1}H NMR (400 MHz, $CDCl_3$): $M_{n,NMR}$ = 6.4 kg mol^{-1}. SEC (Chloroform, PMMA standard): $M_{n,GPC}$ = 15.1 kg mol^{-1}, $Đ_M$ = 1.08. (**Figure S62**)

Synthesis of BA-OH

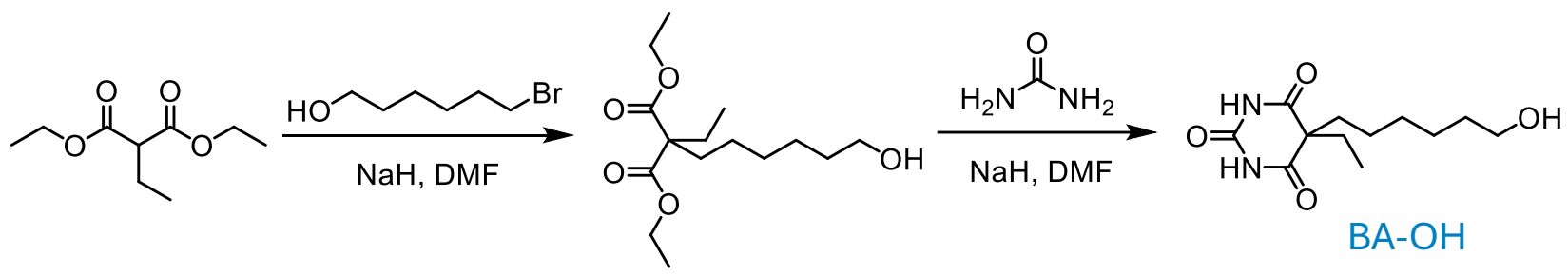

According to a previously reported procedure,[4] BA-OH was synthesized via the following procedure:

NaH (0.43 g, 18.0 mmol) was dispersed in anhydrous DMF (4 mL) and cooled in an ice-water bath. Diethyl ethylmalonate (2.1 g, 11.1 mmol) was added dropwise to the suspension, and the mixture was stirred at room temperature for 30 minutes. The reaction mixture was then cooled to 0 °C, and a solution of 6-bromo-1-hexanol (2.5 g, 13.8 mmol) in DMF (5 mL) was added dropwise. After the addition was complete, the reaction mixture was stirred at 50 °C for 20 hours. Upon completion, the reaction was cooled to room temperature and the solvent was removed under reduced pressure. The residue was diluted with water (40 mL) and Et_2O (30 mL), and the layers were separated. The aqueous phase was extracted with an additional portion

of Et_2O (30 mL). The combined organic layers were washed with water (2 × 30 mL), dried over anhydrous Na_2SO_4, filtered, and concentrated under reduced pressure to afford the crude intermediate diethyl-2-ethyl-2-(6-hydroxyhexyl)malonate. NaH (1.2 g, 50.0 mmol) was then dispersed in DMF (5 mL) and cooled in an ice-water bath. A solution of urea (1.1 g, 18.3 mmol) in DMF (10 mL) was added dropwise, followed by the dropwise addition of a solution of diethyl-2-ethyl-2-(6-hydroxyhexyl)malonate in DMF (5 mL). After the addition was complete, the reaction mixture was stirred at 100 °C for 12 hours. The mixture was cooled to room temperature and poured into saturated $NaHCO_3$ (200 mL). The aqueous phase was extracted with EA (3 × 70 mL), and the combined organic extracts were washed with 1 M HCl, then dried over anhydrous Na_2SO_4. After filtration and concentration under reduced pressure, the crude product was further purified by recrystallization from CH_2Cl_2 to afford the pure compound BA-OH (1.1 g, 39% yield).

^{1}H NMR (400 MHz, $CDCl_3$, 298 K) δ (ppm): 10.13 (s, 2H), 3.54 (t, 2H), 2.64 (s, 1H), 1.95 (m, 4H), 1.45 (m, 2H), 1.23 (m, 6H), 0.81 (t, 3H). (**Figure S12**)

^{13}C NMR (101 MHz, $CDCl_3$, 298 K) δ (ppm): 173.5, 150.0, 62.4, 57.0, 38.6, 32.5, 32.3, 29.2, 25.3, 25.0, 9.4. (**Figure S13**)

ESI-MS (*m/z*) calculated for $[M\text{+Na}]^+$: 279.1315, found: 279.1312. (**Figure S37**)

FT-IR υ (cm^{-1}): 3202, 2933, 2855, 1700, 1409, 1374, 1334, 1309, 1232, 1024, 1001, 823, 759, 667, 623, 495. (**Figure S48**)

Synthesis of PCL_{41}-BA

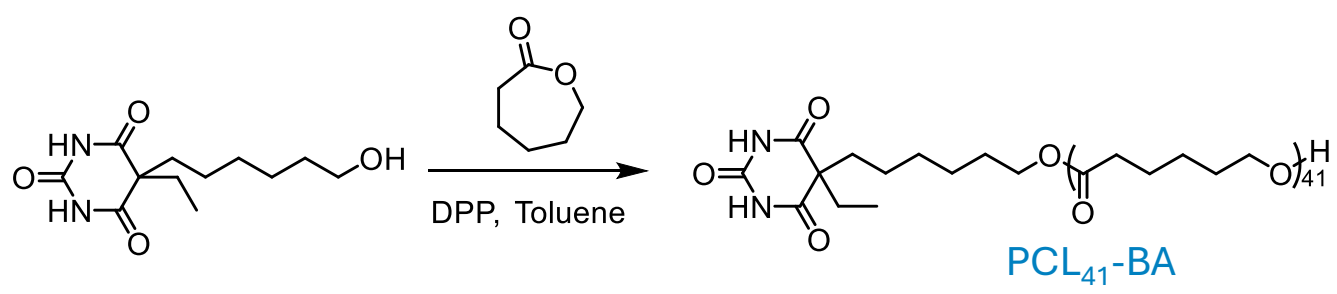

In a nitrogen-filled glove box (oxygen and water content < 0.1 ppm), a solution of DPP (146.5 mg, 0.586 mmol) in dry toluene (3 mL) and a solution of CL (2450.9 mg, 21.472 mmol) in dry toluene (7 mL) were added dropwise to BA-OH (50 mg, 0.195 mmol) dissolved in dry DMF (0.5 mL) under stirring. The mixture was stirred at room temperature for 36 hours. After the reaction, the solution was removed from the glove box and precipitated dropwise into cold Et_2O (3 times). The resulting polymer was collected by centrifugation, dried under vacuum, and analyzed by NMR (**Figure S14** and **S15**) and SEC (**Figure S63** and **Table S1**).

^{1}H NMR (400 MHz, $CDCl_3$, 298 K) δ (ppm): 4.05 (t, 82H), 3.64 (t, 2H), 2.30 (t, 82H), 1.72–1.54 (m, 169H), 1.45–1.28 (m, 82H), 0.88 (t, 3H). (**Figure S14**)

^{13}C NMR (101 MHz, $CDCl_3$, 298 K) δ (ppm): 173.7, 172.4, 148.3, 64.3, 62.8, 57.4, 38.7, 34.3, 32.7, 32.5, 29.1, 28.5, 25.7, 25.5, 25.2, 24.7, 9.6. (**Figure S15**)

The DP of PCL-BA was determined from the ^{1}H NMR spectrum (**Figure S14**) by comparing the integral of the methylene protons adjacent to the carbonyl group in the PCL repeat unit (–$\boldsymbol{CH_2}$–CO–, δ ≈ 2.30 ppm, peak j) with that of the terminal methyl protons of the BA initiator moiety (–$\boldsymbol{CH_3}$, δ ≈ 0.88 ppm, peak c). Based on the ratio of these integrals, the DP was calculated to be approximately 41.

PCL_{41}-BA. ^{1}H NMR (400 MHz, $CDCl_3$): $M_{n,NMR}$ = 4.9 kg mol^{-1}. SEC (Chloroform, PMMA standard): $M_{n,GPC}$ = 10.7 kg mol^{-1}, $Đ_M$ = 1.10. (**Figure S63**)

Synthesis of HW-OH

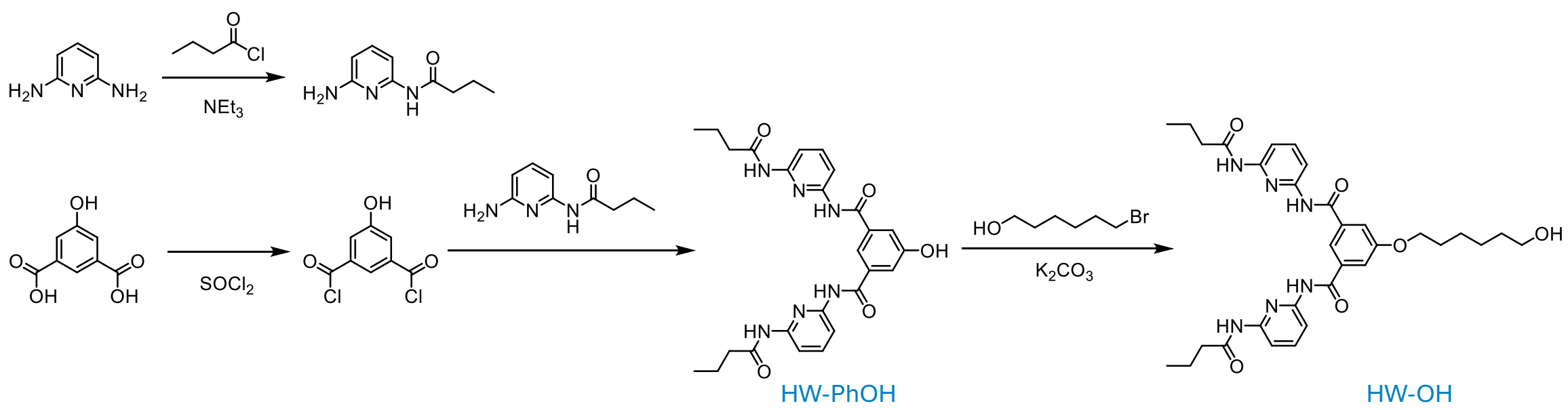

According to previously reported procedures,[5-7] HW-OH was synthesized via the following procedure:

2,6-Diaminopyridine (10.9 g, 100 mmol) and NEt_3 (6.1 g, 60 mmol) were dissolved in THF (200 mL) and cooled in an ice-water bath. A solution of butyryl chloride (5.3 g, 50 mmol) in THF (20 mL) was added dropwise to the reaction mixture. After the addition was complete, the mixture was stirred at room temperature overnight. The reaction mixture was then filtered and concentrated under reduced pressure. The crude product was purified by column chromatography using EA/n-hexane (3:2, v/v) as the eluent to afford the desired compound N-(6-aminopyridin-2-yl)-butyramide as a solid (7.9 g, 88% yield).

^{1}H NMR (400 MHz, $CDCl_3$, 298 K) δ (ppm): 9.78 (s, 1H), 7.31 (t, 1H), 7.23 (d, 1H), 6.15 (d, 1H), 5.70 (s, 2H), 2.29 (t, 2H), 1.56 (m, 2H), 0.87 (t, 3H).

5-Hydroxyisophthalic acid (4.0 g, 22.0 mmol) was heated at 40 °C with $SOCl_2$ (5 mL) and a drop of DMF until gas evolution ceased. Excess $SOCl_2$ was then removed under reduced pressure. The liquid residue crystallized upon cooling to room temperature to yield 5-hydroxy-benzene-1,3-dicarbonyl chloride as a white crystalline product. N-(6-aminopyridin-2-yl)-butyramide (0.9 g, 5.0 mmol) and NEt_3 (0.7 mL, 5.0 mmol) were dissolved in anhydrous THF

(45 mL). The reaction mixture was cooled in an ice-water bath. 5-Hydroxy-benzene-1,3-dicarbonyl chloride (0.5 g, 2.5 mmol) dissolved in 20 mL anhydrous THF was added dropwise over 1 hour. After stirring for 12 hours at room temperature, 20 mL of water was added and the reaction mixture was stirred for an additional 20 minutes. THF was then removed under reduced pressure and the resulting precipitate was filtered and dried at room temperature. The crude product was purified by column chromatography using CH_2Cl_2/MeOH (20:1, v/v) as the eluent to afford the desired compound HW-PhOH as a solid (0.5 g, 39% yield).

^{1}H NMR (400 MHz, DMSO-d_6, 298 K) δ (ppm): 10.33 (s, 2H), 10.18 (s, 1H), 10.08 (s, 2H), 8.00 (s, 1H), 7.82 (m, 7H), 7.52 (s, 2H), 2.39 (t, 4H), 1.62 (m, 4H), 0.91 (t, 6H). (**Figure S16**)

^{13}C NMR (101 MHz, DMSO-d_6, 298 K) δ (ppm): 172.1, 165.2, 157.7, 150.6, 150.1, 140.0, 135.6, 118.2, 117.8, 110.4, 110.0, 38.0, 18.4, 13.6. (**Figure S17**)

ESI-MS (*m/z*) calculated for $[M+H]^+$: 505.2194, found: 505.2218; calculated for $[M+Na]^+$: 527.2013, found: 527.1976. (**Figure S38**)

FT-IR υ (cm^{-1}): 2962, 2873, 1669, 1584, 1507, 1443, 1294, 1240, 1155, 1084, 995, 970, 861, 798, 601, 549, 738, 678. (**Figure S49**)

HW-PhOH (0.25 g, 0.50 mmol), K_2CO_3 (0.10 g, 0.75 mmol), and 6-bromo-1-hexanol (0.10 g, 0.6 mmol) were dissolved in DMF (2 mL). The reaction mixture was stirred at 50 °C overnight. After completion, DMF was removed under reduced pressure. The residue was dissolved in CH_2Cl_2 (10 mL) and extracted with water (3 × 10 mL). The combined organic layers were dried over anhydrous Na_2SO_4, filtered, and concentrated under reduced pressure. The crude product was purified by column chromatography using CH_2Cl_2/MeOH (20:1, v/v) as the eluent to afford the desired compound HW-OH (210.0 mg, 70% yield).

^{1}H NMR (400 MHz, DMSO-d_6, 298 K) δ (ppm): 10.47 (s, 2H), 10.11 (s, 2H), 8.11 (s, 1H), 7.84 (m, 7H), 7.70 (s, 1H), 4.36 (t, 1H), 4.14 (t, 2H), 3.42 (q, 2H), 2.38 (t, 4H), 1.78 (t, 2H), 1.62 (m, 4H), 1.46 (m, 6H), 0.91 (t, 6H). (**Figure S18**)

^{13}C NMR (101 MHz, DMSO-d_6, 298 K) δ (ppm): 172.1, 164.9, 158.7, 150.6, 150.1, 140.0, 135.6, 119.6, 117.1, 110.6, 110.0, 68.1, 60.6, 38.0, 32.5, 28.6, 25.4, 25.3, 18.4, 13.6. (**Figure S19**)

ESI-MS (*m/z*) calculated for $[M+H]^+$: 605.3082, found: 605.3201. (**Figure S39**)

FT-IR υ (cm^{-1}): 3384, 3298, 2943, 2856, 1696, 1660, 1622, 1582, 1521, 1451, 1408, 1344, 1313, 1266, 1197, 1152, 1063, 984, 877, 795, 752, 734, 652, 614. (**Figure S50**)

Synthesis of PCL_{75}-HW

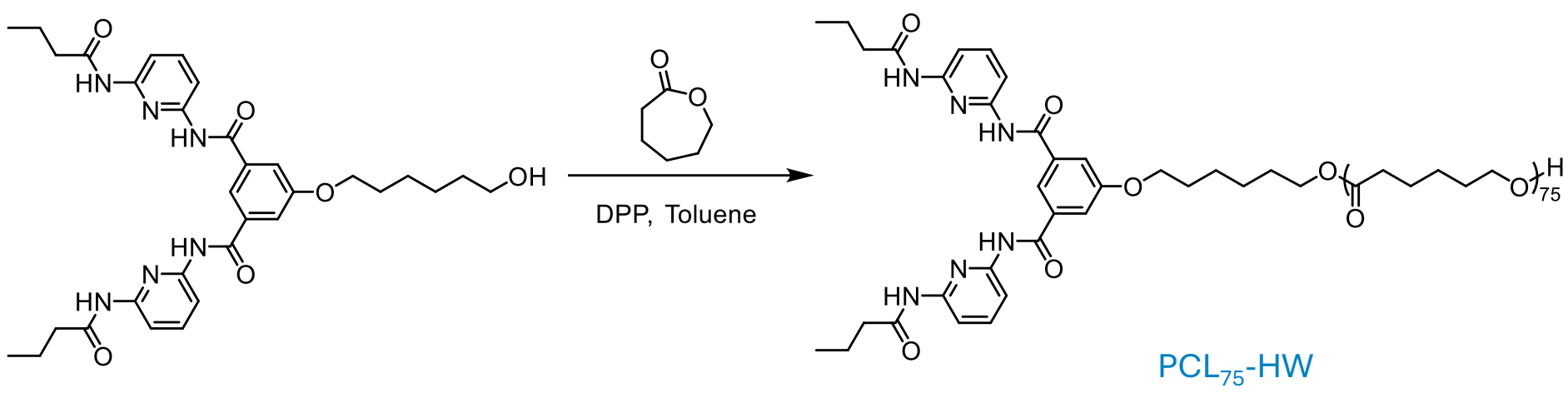

In a nitrogen-filled glove box (oxygen and water content < 0.1 ppm), a solution of DPP (261 mg, 1.043 mmol) in dry toluene (3 mL) and a solution of CL (1637.2 mg, 14.344 mmol) in dry toluene (7 mL) were added dropwise to HW-OH (78.8 mg, 0.130 mmol) dissolved in dry DMF (0.5 mL) under stirring. The mixture was stirred at room temperature for 28 hours. After the reaction, the solution was removed from the glove box and precipitated dropwise into cold Et_2O (3 times). The resulting polymer was collected by centrifugation, dried under vacuum, and analyzed by NMR (**Figure S20** and **S21**) and SEC (**Figure S64** and **Table S1**).

^{1}H NMR (400 MHz, $CDCl_3$, 298 K) δ (ppm): 7.80 (s, 2H), 7.17 (m, 14H), 7.03 (m, 4H), 4.05 (t, 150H), 3.64 (t, 2H), 2.30 (t, 151H), 1.72–1.54 (m, 304H), 1.45–1.28 (m, 153H), 0.87 (t, 6H). (**Figure S20**)

^{13}C NMR (101 MHz, $CDCl_3$, 298 K) δ (ppm): 173.7, 129.5, 124.0, 120.4, 64.3, 62.7, 34.3, 32.5, 28.5, 25.7, 24.7, 18.2, 13.7. (**Figure S21**)

The DP of PCL-HW was determined from the ^{1}H NMR spectrum (**Figure S20**) by comparing the integral of the methylene protons adjacent to the carbonyl group in the PCL repeat unit (–$\boldsymbol{CH_2}$–CO–, δ ≈ 2.30 ppm, peak p) with that of the terminal methyl protons of the HW initiator moiety (–$\boldsymbol{CH_3}$, δ ≈ 0.87 ppm, peak a). Based on the ratio of these integrals, the DP was calculated to be approximately 75.

PCL_{75}-HW. ^{1}H NMR (400 MHz, $CDCl_3$): $M_{n,NMR}$ = 9.1 kg mol^{-1}. SEC (Chloroform, PMMA standard): $M_{n,GPC}$ = 15.2 kg mol^{-1}, $Đ_M$ = 1.15. (**Figure S64**)

Synthesis of NMI-OH

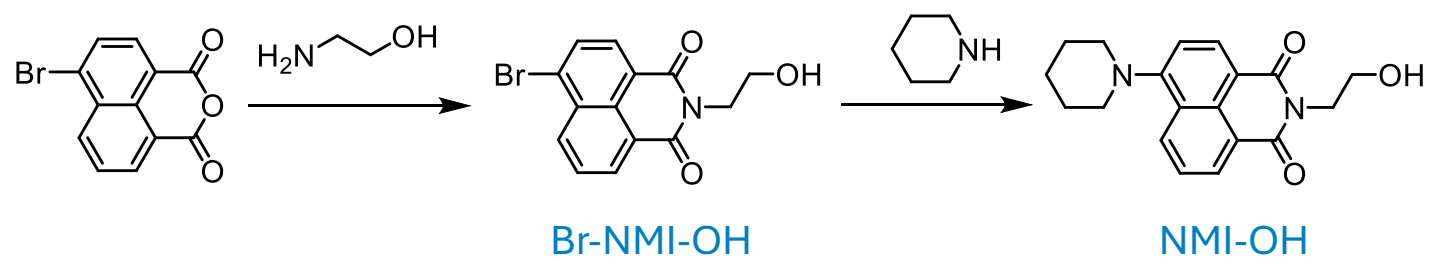

According to a previously reported procedure,[8] NMI-OH was synthesized via the following procedure:

4-Bromo-1,8-naphthalic anhydride (1.0 g, 3.609 mmol) was dissolved in 20 mL EtOH in a round-bottom flask. To this solution, ethanolamine (0.44 g, 7.218 mmol) was added, and the reaction mixture was refluxed at 70 °C for 24 hours. After completion, the reaction mixture was cooled to room temperature, leading to the precipitation of the product. The solid was collected by filtration and washed thoroughly with a saturated $NaHCO_3$ solution. The obtained solid was dried under vacuum to yield Br-NMI-OH as a cream-colored solid (756.6 mg, 65% yield).

^{1}H NMR (400 MHz, DMSO-d_6, 298 K) δ (ppm): 8.57 (t, 2H), 8.35 (d, 1H), 8.24 (d, 1H), 8.01 (t, 1H), 4.81 (br s, 1H), 4.15 (t, 2H), 3.64 (m, 2H).

Br-NMI-OH (0.3 g, 1.01 mmol) was taken in a round-bottom flask along with an excess of piperidine (17.2 g, 202 mmol). The reaction mixture was refluxed at 110 °C for 48 hours. After cooling to room temperature, the resulting solid was collected by filtration and washed thoroughly with water several times to remove excess piperidine. The product was then dried under reduced pressure to afford the final product, NMI-OH, as a yellow solid (232.9 mg, 71% yield).

^{1}H NMR (400 MHz, $CDCl_3$, 298 K) δ (ppm): 8.54 (d, 1H), 8.46 (d, 1H), 8.36 (d, 1H), 7.65 (t, 1H), 7.16 (d, 1H), 4.43 (t, 2H), 3.96 (t, 2H), 3.23 (t, 4H), 2.64 (s, 1H), 1.88 (m, 4H), 1.72 (m, 2H). (**Figure S22**)

^{13}C NMR (101 MHz, $CDCl_3$, 298 K) δ (ppm): 165.6, 165.2, 157.8, 133.2, 131.5, 131.1, 130.2, 126.3, 125.5, 122.9, 115.5, 114.8, 62.3, 54.7, 42.9, 26.3, 24.5. (**Figure S23**)

ESI-MS (*m/z*) calculated for $[M+H]^+$: 325.1547, found: 325.1552. (**Figure S40**)

FT-IR υ (cm^{-1}): 2935, 2851, 2806, 2246, 1688, 1644, 1584, 1574, 1514, 1448, 1376, 1349, 1232, 1177, 1129, 1080, 1053, 1025, 972, 901, 861, 845, 780, 757, 727, 645, 608, 585, 665, 499, 459, 417. (**Figure S51**)

Synthesis of NMI-Ad

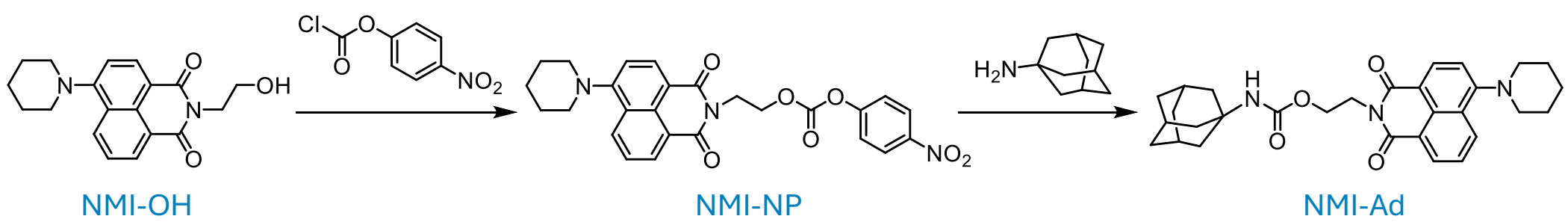

NMI-OH (162.0 mg, 0.50 mmol) and 4-nitrophenyl chloroformate (120.0 mg, 0.60 mmol) were dissolved in CH_2Cl_2 (5 mL). NEt_3 (50 µL, 0.36 mmol) was then added, and the mixture was stirred at room temperature for 1 hour. After completion of the reaction, the mixture was

concentrated under reduced pressure and purified by column chromatography using CH_2Cl_2 as the eluent to afford the product NMI-NP (180.0 mg, 74% yield).

^{1}H NMR (400 MHz, $CDCl_3$, 298 K) δ (ppm): 8.52 (d, 1H), 8.42 (d, 2H), 8.25 (d, 2H), 7.85 (t, 1H), 7.51 (d, 2H), 7.34 (d, 1H), 4.53 (d, 4H), 3.22 (t, 4H), 1.83 (m, 4H), 1.67 (m, 2H).

NMI-NP (50.0 mg, 0.10 mmol) and 1-adamantylamine (30.2 mg, 0.20 mmol) were dissolved in DMF (2 mL). NEt_3 (30 μL, 0.22 mmol) was added dropwise at room temperature, and the reaction mixture was stirred overnight. Upon completion of the reaction, the solvent was removed under reduced pressure. The residue was purified by column chromatography using CH_2Cl_2/n-hexane (10:1, v/v) as the eluent to give the desired product NMI-Ad (43.0 mg, 86% yield).

^{1}H NMR (400 MHz, $CDCl_3$, 298 K) δ (ppm): 8.56 (d, 1H), 8.48 (d, 1H), 8.37 (d, 1H), 7.66 (t, 1H), 7.16 (d, 1H), 4.45 (d, 3H), 4.34, (s, 2H), 3.22 (t, 4H), 1.98 (m, 7H), 1.81 (m, 8H), 1.59 (s, 6H). (**Figure S24**)

^{13}C NMR (101 MHz, $CDCl_3$, 298 K) δ (ppm): 164.7, 164.2, 157.5, 132.9, 131.3, 130.8, 130.2, 126.4, 125.5, 123.2, 116.0, 114.8, 61.6, 54.7, 50.7, 41.8, 39.4, 36.4, 29.6, 26.4, 24.5. (**Figure S25**)

ESI-MS (*m/z*) calculated for $[M+H]^+$: 502.2700, found: 502.2742; calculated for $[M+Na]^+$: 524.2520, found: 524.2564. (**Figure S41**)

FT-IR υ (cm^{-1}): 2906, 2848, 2249, 1694, 1651, 1588, 1512, 1451, 1377, 1353, 1276, 1293, 1417, 1233, 1223, 1186, 1154, 1061, 910, 861, 780, 758, 727, 645, 587, 500, 457. (**Figure S52**)

Synthesis of NMI-CD

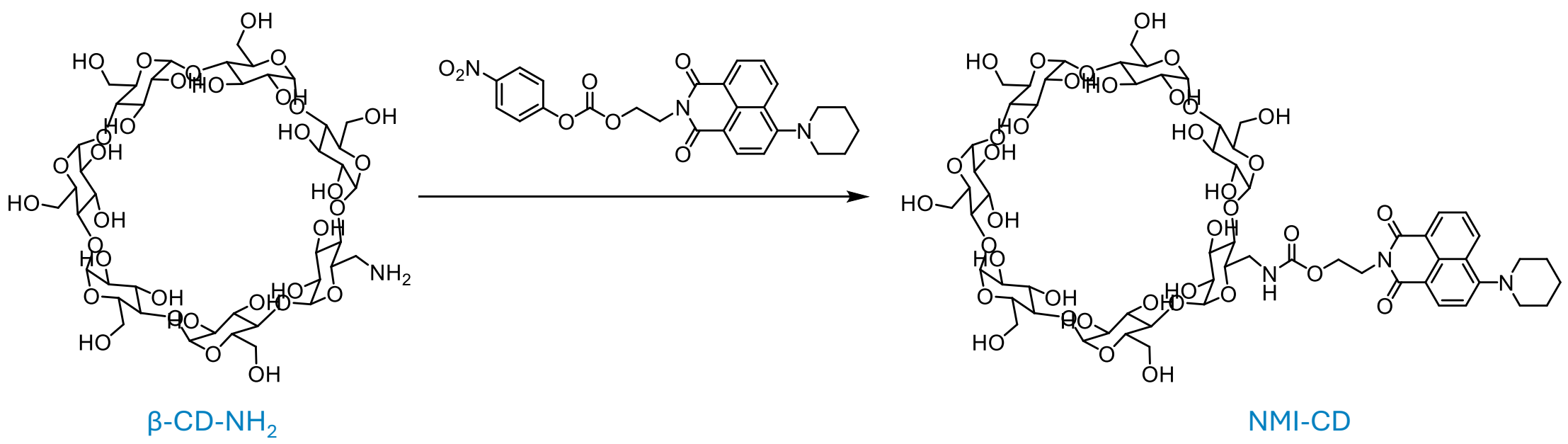

NMI-NP (50.0 mg, 0.1 mmol) and β-CD-NH_2 (117.7 mg, 0.1 mmol) were dissolved in DMF (2 mL). NEt_3 (0.1 mL, 0.7 mmol) was added dropwise at room temperature, and the reaction mixture was stirred overnight. Upon completion of the reaction, the solution was poured into 50 mL acetone. The precipitate was collected by filtration, washed with acetone (3 × 30 mL), and dried under vacuum to give NMI-CD as a white solid (130.0 mg, 85% yield).

^{1}H NMR (400 MHz, DMSO-d_6, 298 K) δ (ppm): 8.48 (d, 1H), 8.42 (m, 2H), 7.81 (t, 1H), 7.33 (d, 1H), 6.83 (t, 1H), 5.69 (m, 14H), 4.82 (m, 7H), 4.47 (m, 10H), 3.63 (m, 26H), 3.35 (m, 24H), 1.82 (s, 4H), 1.66 (s, 2H). (**Figure S26**)

^{13}C NMR (101 MHz, DMSO-d_6, 298 K) δ (ppm): 163.7, 163.2, 156.8, 156.2, 132.4, 130.8, 130.7, 129.3, 125.9, 125.5, 122.5, 115.0, 102.0, 101.7, 83.3, 81.6, 81.4, 73.0, 72.5, 72.4, 72.1, 72.0, 69.9, 68.5, 60.7, 59.9, 59.6, 54.0, 45.8, 41.2, 25.8, 23.9. (**Figure S27**)

ESI-MS (*m/z*) calculated for $[M+H]^+$: 1484.5197, found: 1484.5365; calculated for $[M+Na]^+$: 1506.5016, found: 1506.5216. (**Figure S42**)

FT-IR υ (cm^{-1}): 3349, 2925, 1694, 1644, 1586, 1516, 1376, 1237, 1152, 1080, 1026, 943, 860, 784, 758, 704, 579, 528. (**Figure S53**)

Synthesis of SPY-HW

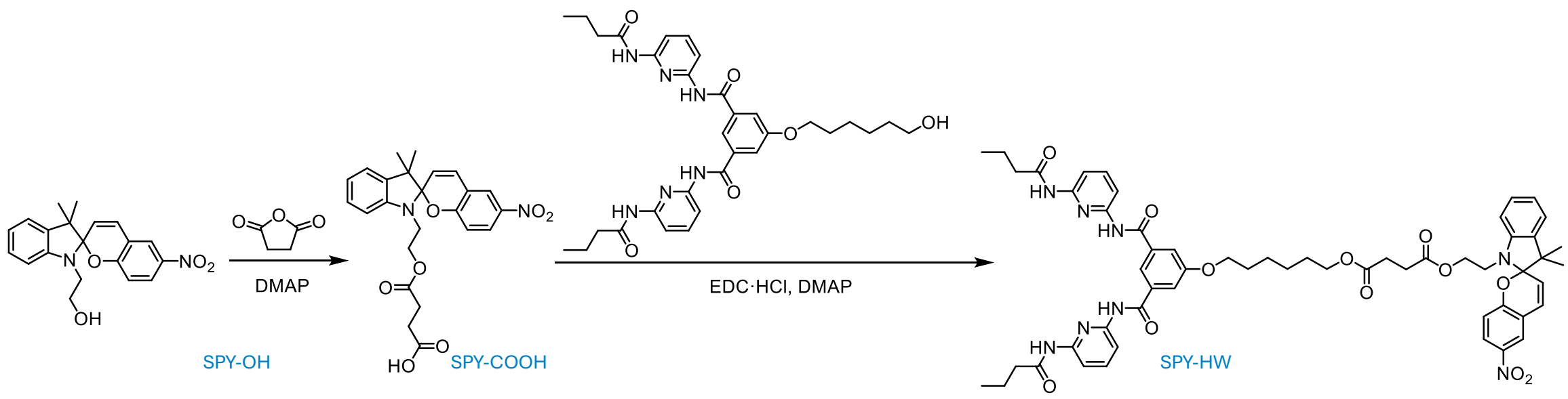

SPY-OH (0.10 g, 0.28 mmol), succinic anhydride (34.0 mg, 0.34 mmol), and DMAP (38.0 mg, 0.31 mmol) were dissolved in CH_2Cl_2 (3 mL). The reaction mixture was stirred at room temperature overnight. After completion, the solvent was removed under reduced pressure, and the crude product was purified by column chromatography using EA/n-hexane (1:1, v/v) as the eluent to afford SPY-COOH (90.0 mg, 67% yield).

^{1}H NMR (400 MHz, $CDCl_3$, 298 K) δ (ppm): 8.00 (m, 2H), 7.20 (m, 1H), 7.08 (m, 1H), 6.90 (m, 2H), 6.76 (m, 1H), 6.68 (d, 1H), 5.90 (d, 1H), 4.27 (m, 2H), 3.48 (m, 2H), 2.62 (m, 4H), 1.28 (m, 4H), 1.165 (s, 3H).

SPY-COOH (90.0 mg, 0.20 mmol), HW-OH (74.0 mg, 0.12 mmol), EDC·HCl (63.0 mg, 0.33 mmol), and DMAP (25.0 mg, 0.20 mmol) were dissolved in CH_2Cl_2 (2 mL). The reaction mixture was stirred at room temperature overnight. After the reaction was complete, the solvent was removed under reduced pressure, and the crude product was purified by column chromatography using EA/n-hexane (1:1, v/v) as the eluent to afford the desired compound SPY-HW (90.0 mg, 73% yield).

^{1}H NMR (400 MHz, $CDCl_3$, 298 K) δ (ppm): 8.55 (s, 2H), 7.99 (m, 9H), 7.71 (t, 2H), 7.58 (s, 2H), 7.18 (t, 1H), 7.06 (d, 1H), 6.89 (m, 2H), 6.72 (d, 1H), 6.65 (d, 1H), 5.86 (d, 1H), 4.21 (m,

2H), 4.08 (t, 2H), 3.99 (t, 2H), 3.46 (m, 2H), 2.58 (d, 4H), 2.37 (t, 4H), 1.76 (m, 6H), 1.64 (m, 2H), 1.42 (m, 4H), 1.25 (s, 3H), 1.14 (s, 3H), 1.00 (t, 6H). (**Figure S28**)

^{13}C NMR (101 MHz, $CDCl_3$, 298 K) δ (ppm): 172.5, 172.4, 164.5, 150.0, 149.4, 146.7, 141.0, 136.1, 135.8, 128.5, 128.0, 126.1, 122.9, 122.0, 121.8, 120.1, 117.3, 115.6, 110.3, 109.7, 106.8, 106.6, 64.9, 62.8, 53.0, 42.4, 39.7, 29.1, 29.0, 28.6, 26.0, 25.7, 20.0, 18.9, 13.9. (**Figure S29**)

ESI-MS (*m/z*) calculated for $[M+H]^+$: 1039.4560, found: 1039.4537. (**Figure S43**)

FT-IR υ (cm^{-1}): 2961, 2933, 2871, 2250, 1733, 1682, 1582, 1511, 1443, 1334, 1294, 1240, 1155, 1126, 1087, 1026, 953, 907, 800, 730, 680, 646, 551. (**Figure S54**)

Synthesis of BDP-HW

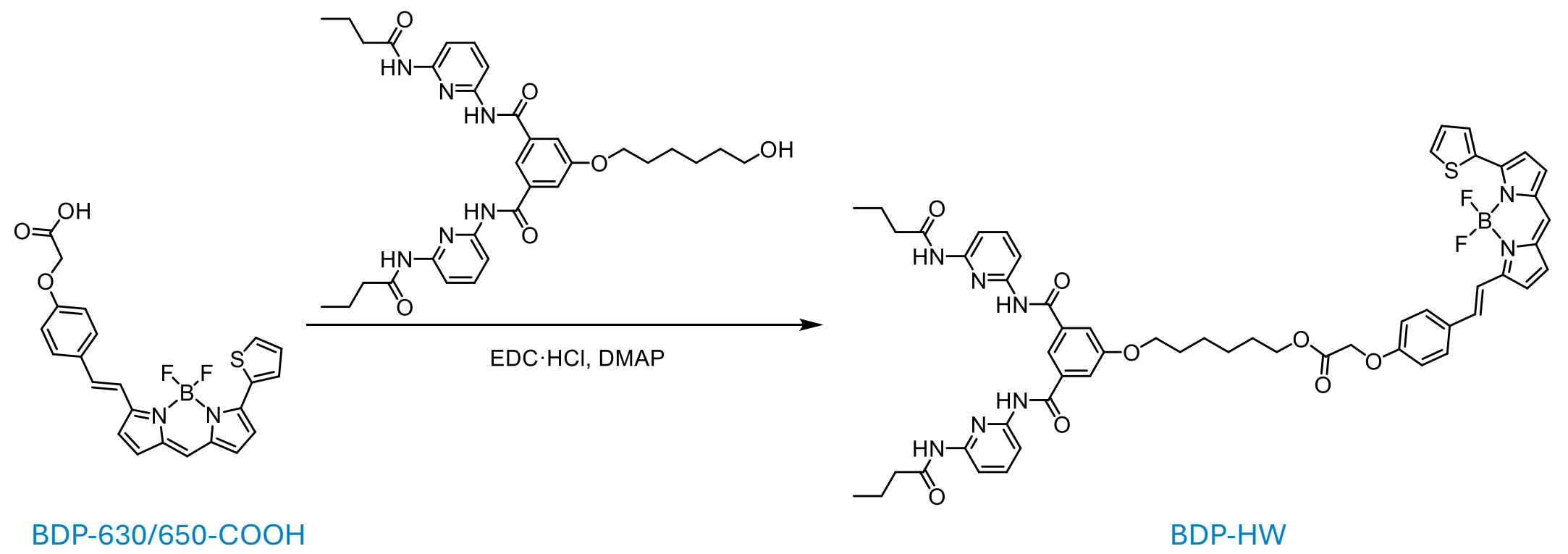

BDP-630/650-COOH (20.0 mg, 0.044 mmol), HW-OH (40.0 mg, 0.066 mmol), EDC·HCl (13.0 mg, 0.068 mmol), and DMAP (7.0 mg, 0.057 mmol) were dissolved in CH_2Cl_2 (2 mL). The reaction mixture was stirred at room temperature overnight. After completion, the solvent was removed under reduced pressure, and the crude product was purified by column chromatography using CH_2Cl_2/EA/n-hexane (1:1:1, v/v/v) as the eluent to afford BDP-HW (25.0 mg, 55% yield).

^{1}H NMR (400 MHz, $CDCl_3$, 298 K) δ (ppm): 8.44 (s, 2H), 8.17 (d, 1H), 8.00 (d, 2H), 7.95 (d, 2H), 7.88 (s, 1H), 7.75 (t, 2H), 7.60 (s, 2H), 7.51 (m, 5H), 7.42 (d, 1H), 7.14 (m, 2H), 6.89 (m, 4H), 6.83 (d, 1H), 6.75 (d, 1H), 6.65 (d, 1H), 4.67 (s, 2H), 4.27 (t, 2H), 3.96 (t, 2H), 2.28 (t, 4H), 1.72 (m, 8H), 1.42 (m, 4H), 0.99 (t, 6H). (**Figure S30**)

^{13}C NMR (101 MHz, $CDCl_3$, 298 K) δ (ppm): 171.6, 164.2, 159.9, 149.8, 149.4, 140.9, 137.5, 135.9, 130.1, 130.0, 129.5, 129.1, 128.9, 123.9, 117.6, 117.0, 115.0, 110.1, 109.7, 68.5, 65.6, 65.3, 39.7, 31.7, 30.5, 30.3, 29.8, 29.0, 28.5, 25.6, 25.4, 18.9, 13.9. (**Figure S31**)

ESI-MS (*m/z*) calculated for $[M+H]^+$: 1037.3997, found: 1037.4010. (**Figure S44**)

FT-IR υ (cm^{-1}): 2961, 2926, 2871, 1737, 1683, 1595, 1511, 1445, 1297, 1242, 1174, 1126, 1085, 1044, 1009, 979, 847, 800, 690. (**Figure S55**)

Synthesis of NMI-Azo

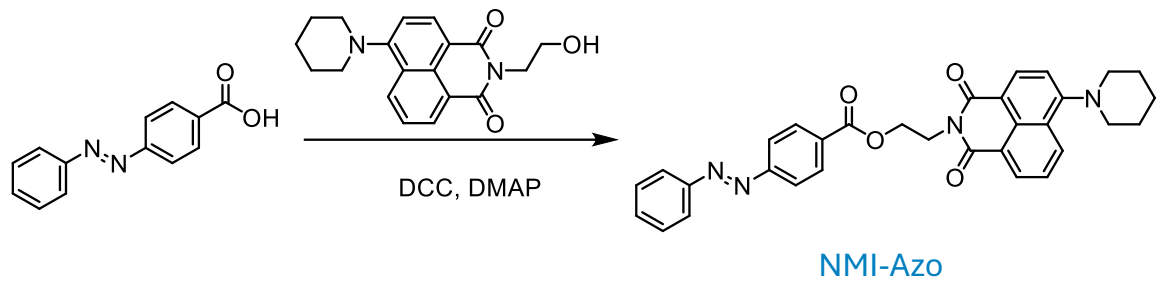

4-(Phenylazo)benzoic acid (226.0 mg, 1.0 mmol), NMI-OH (338.0 mg, 1.05 mmol), and EDC·HCl (422.0 mg, 2.2 mmol) were dissolved in CH_2Cl_2 (10 mL). The resulting solution was cooled in an ice-water bath, and DMAP (171.0 mg, 1.4 mmol) was added in portions. The mixture was then stirred at room temperature overnight. Upon completion, saturated brine (20 mL) was added, and the organic layer was extracted and collected. The organic phase was dried over anhydrous Na_2SO_4, filtered, and concentrated under reduced pressure. The crude product was purified by column chromatography using EA/n-hexane/CH_2Cl_2 (1:3:1, v/v/v) as the eluent to afford the desired compound NMI-Azo (350.0 mg, 66% yield).

^{1}H NMR (400 MHz, $CDCl_3$, 298 K) δ (ppm): 8.56 (d, 1H), 8.48 (d, 1H), 8.37 (d, 1H), 8.12 (d, 2H), 7.89 (m, 4H), 7.66 (t, 1H), 7.50 (m, 3H), 7.17 (d, 1H), 4.68 (s, 4H), 3.22 (s, 4H), 1.87 (m, 4H), 1.72 (m, 2H). (**Figure S32**)

^{13}C NMR (101 MHz, $CDCl_3$, 298 K) δ (ppm): 166.0, 164.7, 164.2, 157.6, 155.1, 152.7, 133.0, 132.0, 131.7, 131.3, 130.9, 130.9, 130.2, 129.3, 126.4, 125.5, 123.2, 123.0, 122.7, 115.8, 114.9, 62.9, 54.6, 38.8, 26.3, 24.6. (**Figure S33**)

ESI-MS (*m/z*) calculated for $[M+H]^+$: 533.2183, found: 533.2154; calculated for $[M+Na]^+$: 555.2003, found: 555.1993. (**Figure S45**)

FT-IR υ (cm^{-1}): 3069, 2935, 2809, 2851, 2255, 1719, 1694, 1651, 1586, 1575, 1514, 1464, 1371, 1352, 1265, 1232, 1183, 1112, 1080, 1063, 1012, 910, 861, 779, 759, 730, 696, 646, 582, 546, 459. (**Figure S56**)

Synthesis of BDP-Ad

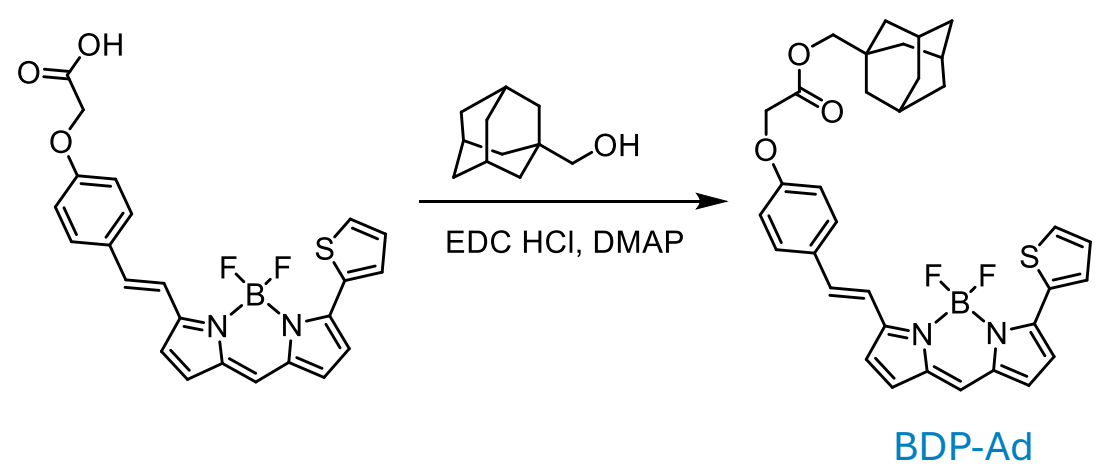

BDP-630/650-COOH (20.0 mg, 0.044 mmol), Ad-OH (11.1 mg, 0.066 mmol), EDC·HCl (13.0 mg, 0.068 mmol), and DMAP (7.0 mg, 0.057 mmol) were dissolved in CH_2Cl_2 (2 mL). The reaction mixture was stirred at room temperature overnight in the dark. After completion, the solvent was removed under reduced pressure, and the crude product was purified by column

chromatography using EA/n-hexane (1:3, v/v) as the eluent to afford BDP-Ad (18.2 mg, 68% yield).

^{1}H NMR (400 MHz, $CDCl_3$, 298 K) δ (ppm): 8.20 (d, 1H), 7.57 (t, 3H), 7.46 (d, 1H), 7.34 (d, 1H), 7.21 (t, 1H), 7.05 (t, 2H), 6.94 (m, 4H), 6.79 (d, 1 H), 4.71 (s, 2H), 3.81 (s, 2H), 1.96 (s, 3H), 1.70 (d, 3H), 1.62 (d, 3H), 1.48 (d, 6H). (**Figure S34**)

^{13}C NMR (101 MHz, $CDCl_3$, 298 K) δ (ppm): 169.0, 159.1, 156.8, 149.2, 137.7, 136.9, 136.8, 134.6, 130.7, 130.2, 130.0, 129.5, 129.1, 128.9, 128.8, 124.1, 119.8, 117.7, 117.5, 115.2, 75.0, 65.4, 39.2, 37.0, 33.4, 28.1. (**Figure S35**)

ESI-MS (*m/z*) calculated for $[M+H]^+$: 599.2346, found: 599.2383; calculated for $[M+Na]^+$: 621.2165, found: 621.2136. (**Figure S46**)

FT-IR υ (cm^{-1}): 2902, 2848, 1753, 1595, 1534, 1511, 1469, 1424, 1408, 1343, 1297, 1275, 1247, 1198, 1174, 1124, 1085, 1041, 1009, 979, 846, 822, 776, 690. (**Figure S57**)

Preparation of seed solutions

According to literature,[9] the seeds have been prepared by flash freezing method. The preparation of flash-frozen seeds was carried out in a 7 mL vial with the polymer concentration maintained at 0.1 mg mL^{-1}. Specifically, 4 µL of PCL-*b*-PDMA stock solution (50 mg mL^{-1} in THF) was introduced into 2 ml of EtOH. The mixture was heated to 75 °C for a specified duration (1–60 minutes) and then rapidly cooled by immersion into a dry-ice-acetone bath (or ice bath and natural cooling) for several minutes (1–5 minutes). The resulting seed micelles were obtained after equilibration to room temperature. Seed samples of different concentrations were prepared by tuning the addition of PCL-*b*-PDMA stock solution. For determination of the average seed length at least 100 particles were analyzed.

Epitaxial growth of platelets

Blends of PCL based homopolymer (such as PCL_{50}-CTA, PCL_{67}-OH, PCL_{67}-CD, PCL_{54}-Ad, PCL_{41}-BA, PCL_{75}-HW) and PCL_{50}-*b*-$PDMA_{193}$ block copolymer (1:1, w/w) were dissolved in $CHCl_3$, THF, DMF, or solvent mixtures to prepare unimer solutions (10 mg mL^{-1}). These unimers solutions were added to dispersions of crystalline seeds (0.01 or 0.005 mg mL^{-1} in EtOH) in glass vials. A typical living CDSA procedure involved adding 10 µL of unimer solution (10 mg mL^{-1}) to 1 mL of seed solution (0.01 mg mL^{-1}), corresponding to a unimer-to-seed ratio of 10. During addition, the mixture (1 mL scale in a 1.75 mL glass vial with a 5 × 2 mm stir bar) was stirred at 1000 rpm for 10 seconds at room temperature, followed by aging for 1 hour. The unimer-to-seed ratio was adjusted by varying the volume of unimer solution added to the seed dispersion.

Epitaxial growth of multi-layered platelets

Blends of PCL based homopolymer (such as PCL_{50}-CTA, PCL_{67}-OH, PCL_{67}-CD, PCL_{54}-Ad, PCL_{41}-BA, PCL_{75}-HW) and PCL_{50}-*b*-$PDMA_{193}$ block copolymer (1:1, w/w) were dissolved in $CHCl_3$, THF, DMF, or solvent mixtures to prepare unimer solutions (10 mg mL^{-1}). A defined volume of the unimer solution was added to a dispersion of pre-formed platelet seeds (0.01 mg mL^{-1} in EtOH). During unimer addition, the mixture was stirred at 1000 rpm with a 5 × 2 mm stir bar in 1.75 mL glass vial for 10 seconds at room temperature, followed by aging for 1 hour prior to analysis by microscopic characterization.

Supramolecular interaction protocol of CDSA assemblies and small molecules

To evaluate supramolecular interactions between CDSA assemblies and small molecules, 3–20 µL (the exact volume depending on prepared platelet size) of a supramolecular motif solution (e.g., NMI-Ad, NMI-CD, NMI-Azo, SPY-HW, BDP-HW or CPT) in EtOH, DMF or $CHCl_3$ at the desired concentration (see table below) was added to 500 µL of a solution of pre-formed CDSA assemblies' solution. The mixtures were stirred gently at 150 rpm with a 5 × 2 mm stir bar in a 1.75 mL glass vial overnight and then equilibrated before subsequent characterizations.

Substance	Solvent	Concentration (mg mL^{-1})
Ad-OH	EtOH	0.1
NMI-OH	EtOH	0.1
NMI-Ad	EtOH	0.15
NMI-CD	DMF	0.5
NMI-Azo	$CHCl_3$	0.15
BDP-Ad	EtOH	0.4
SPY	EtOH	0.15
SPY-HW	EtOH	0.33
BDP-HW	EtOH	0.5
CPT	EtOH	0.1

Transfer platelets into different buffer solutions

The platelet dispersion in ethanol was allowed to evaporate naturally until dry. The same volume of the desired buffer solution was then added to the vial, and the sample was shaken overnight to redisperse the platelets in the buffer.

Separation procedure of platelets and free molecules

After CPT loading or competitive CPT release, the mixture was subjected to centrifugal filtration (Amicon Ultra/Minicon, 10 kDa MWCO) to separate platelet-bound species from free (unbound) small molecules. The dispersion was transferred into a spin filter unit and centrifuged, allowing the platelet to be retained while free molecules passed through the membrane. The retained platelets were subsequently washed several times with fresh ultrapure water to ensure complete removal of unbound species, and then redispersed for further characterization.

Supplementary Figures and Tables

NMR spectra

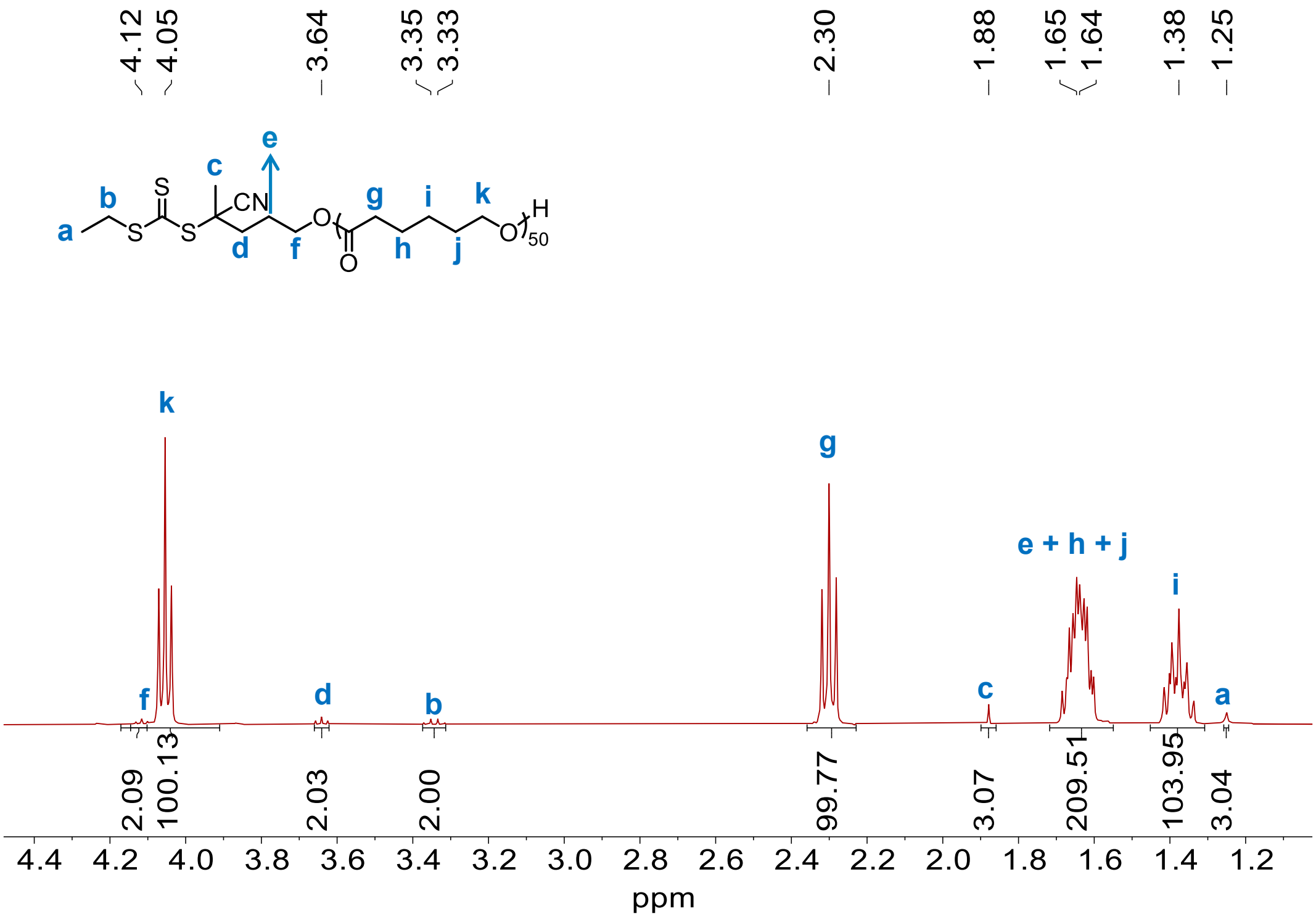

Supplementary Figure S1. ^{1}H NMR spectrum ($CDCl_3$, 400 MHz, 298 K) of PCL_{50}-CTA.

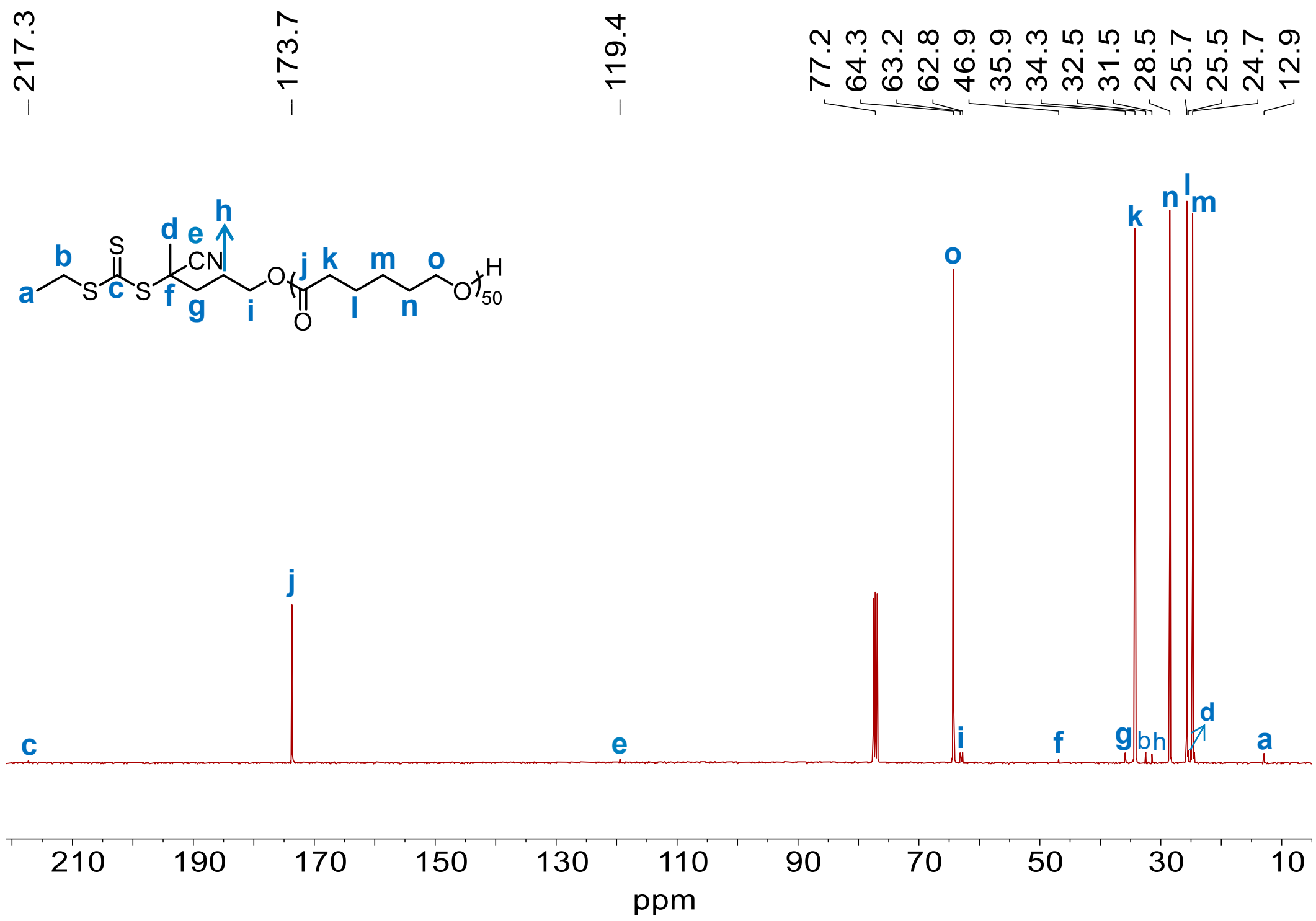

Supplementary Figure S2. ^{13}C NMR spectrum ($CDCl_3$, 101 MHz, 298 K) of PCL_{50}-CTA.

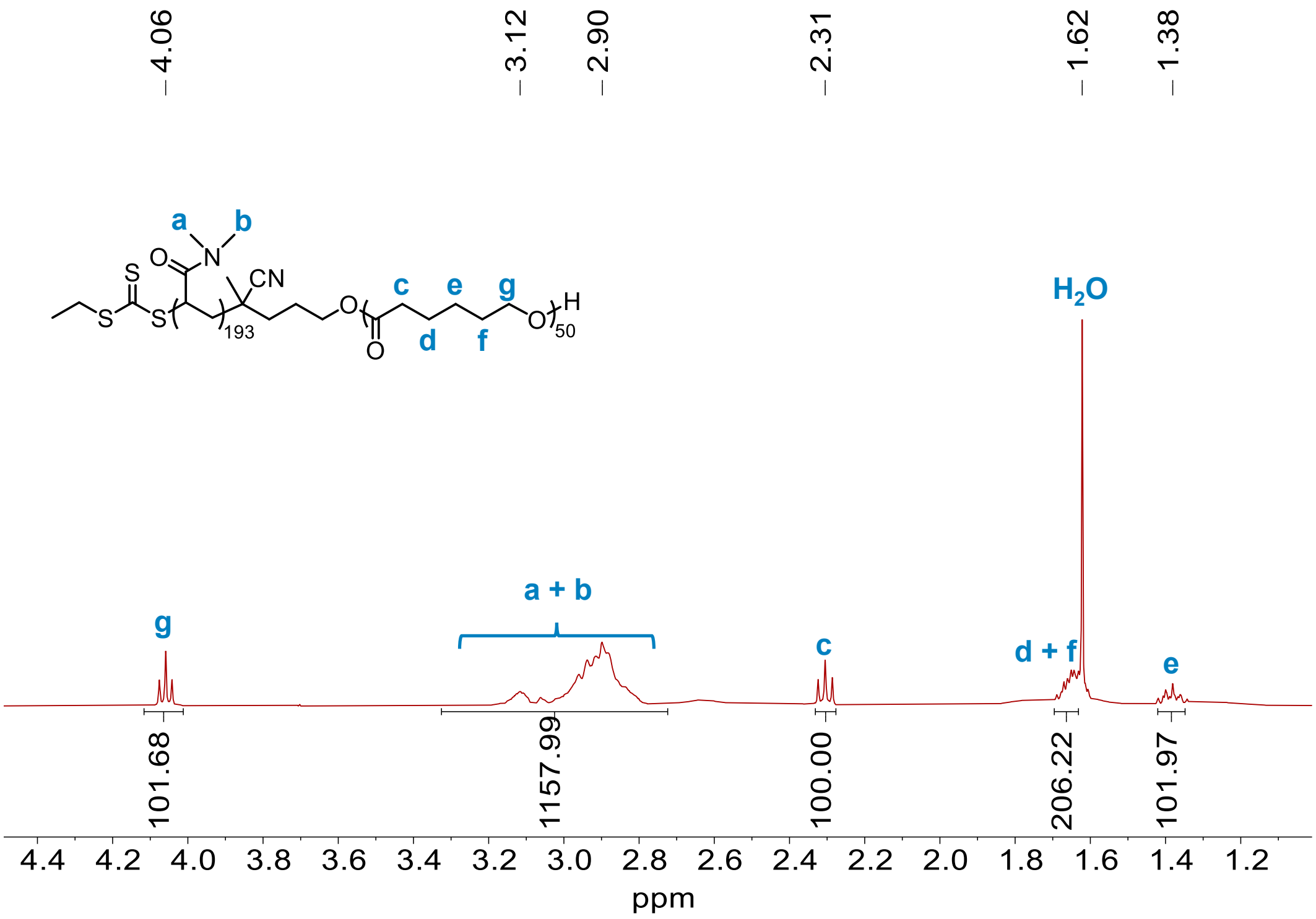

Supplementary Figure S3. ^{1}H NMR spectrum ($CDCl_3$, 400 MHz, 298 K) of PCL_{50}-*b*-$PDMA_{193}$.

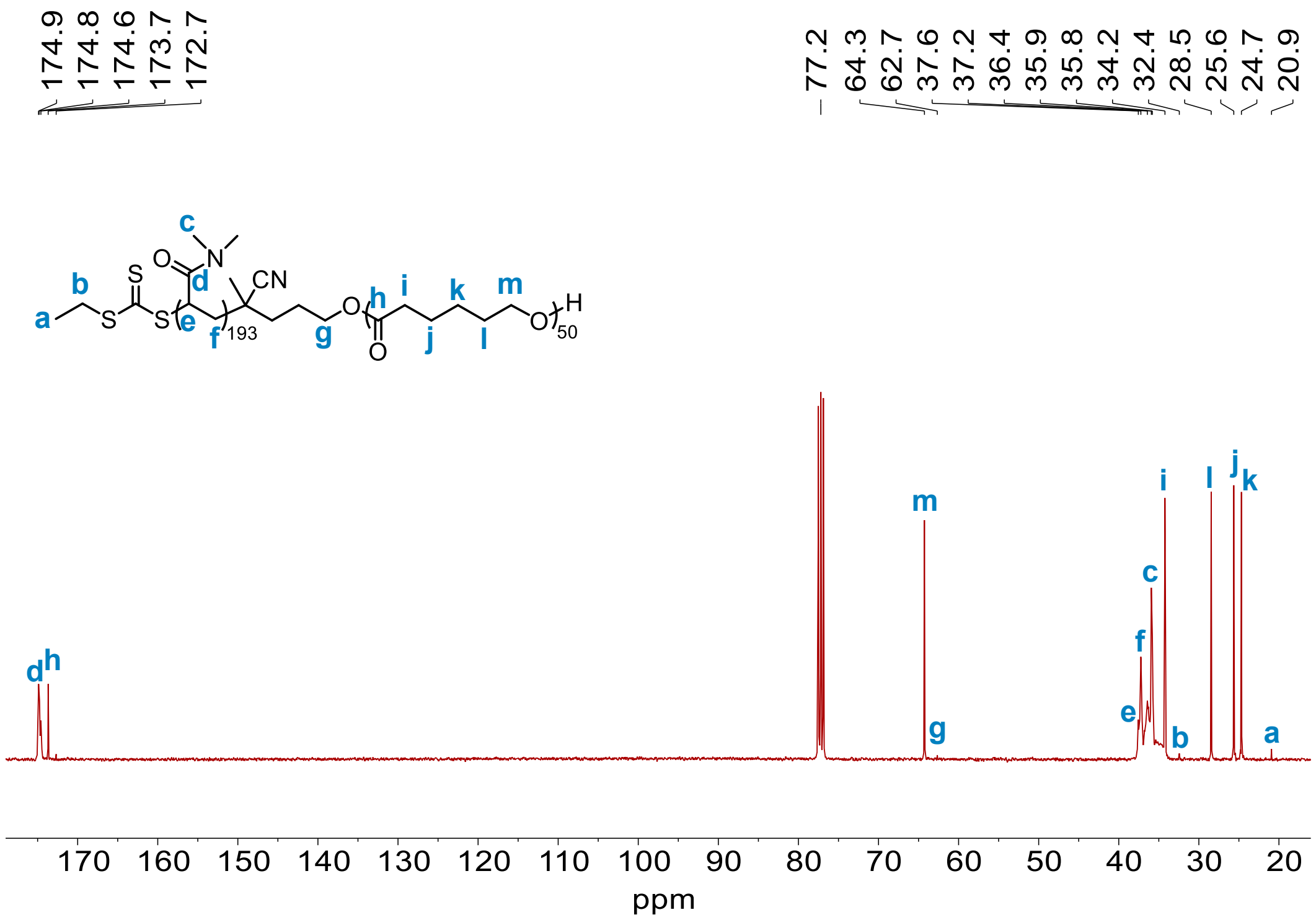

Supplementary Figure S4. ^{13}C NMR spectrum ($CDCl_3$, 101 MHz, 298 K) of PCL_{50}-*b*-$PDMA_{193}$.

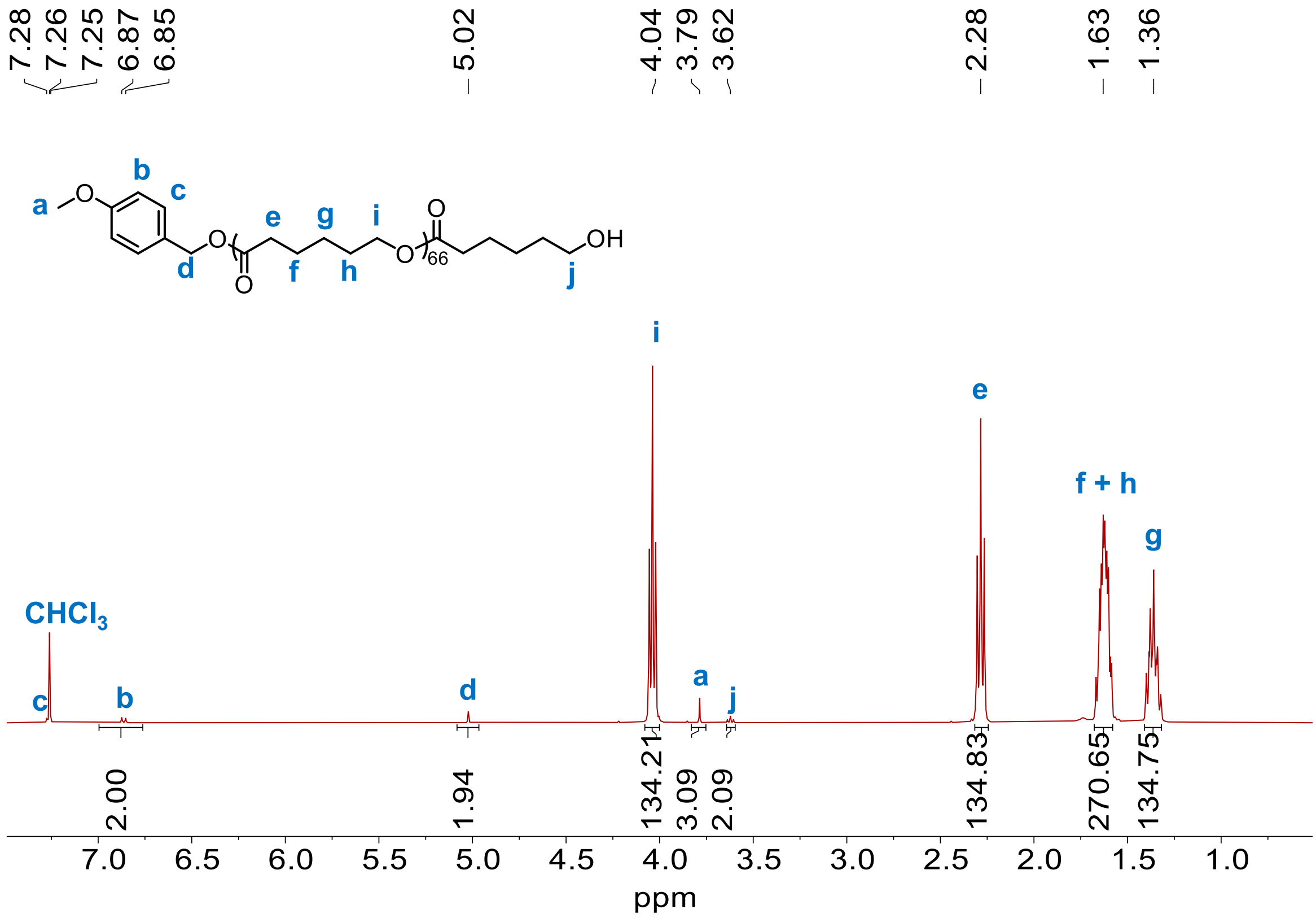

Supplementary Figure S5. ^{1}H NMR spectrum ($CDCl_3$, 400 MHz, 298 K) of PCL_{67}-OH.

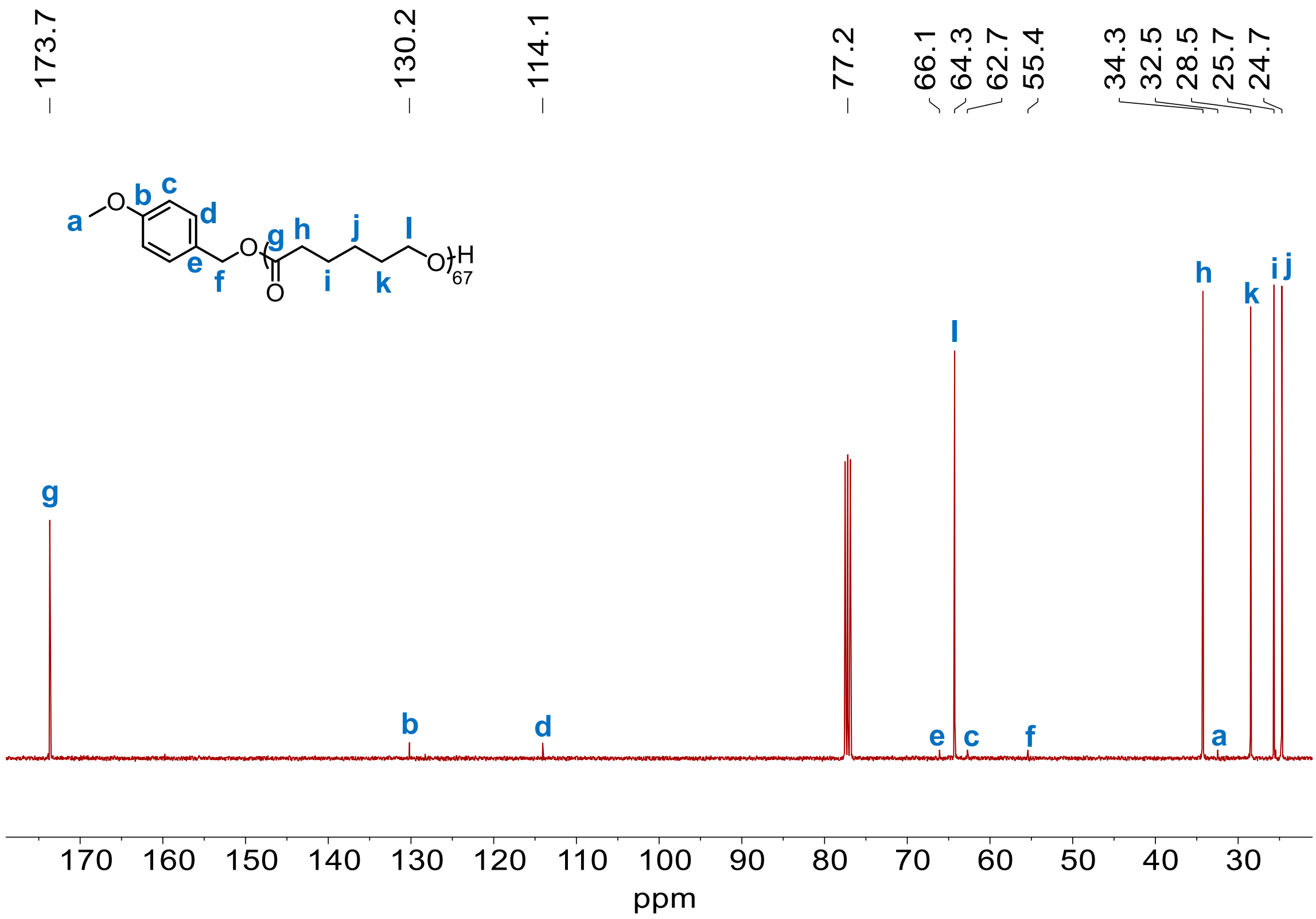

Supplementary Figure S6. ^{13}C NMR spectrum ($CDCl_3$, 101 MHz, 298 K) of PCL_{67}-OH.

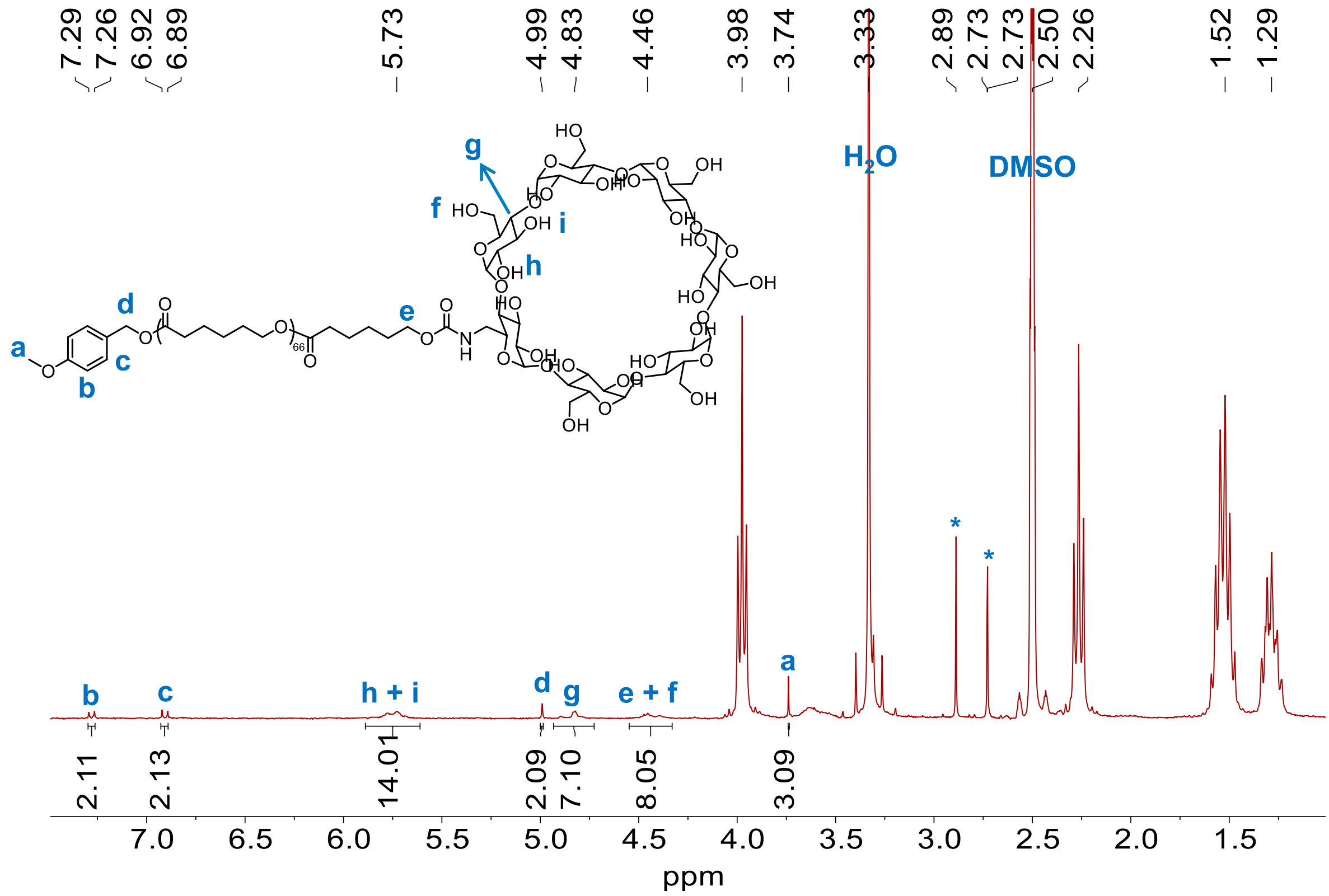

Supplementary Figure S7. ^{1}H NMR spectrum (DMSO-d_6, 400 MHz, 298 K) of PCL$_{67}$-CD. Residual DMF peaks are marked with an asterisk.

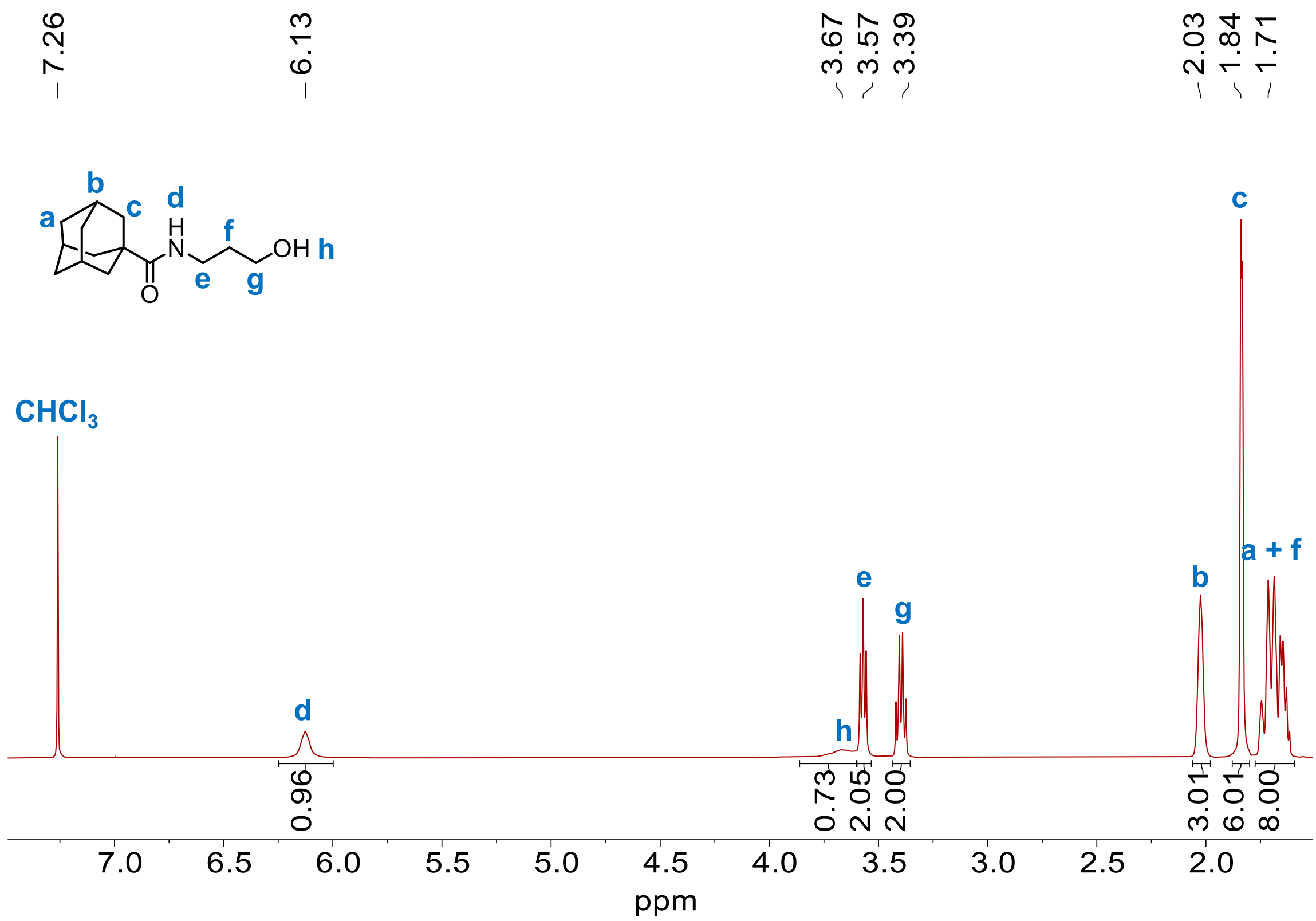

Supplementary Figure S8. ^{1}H NMR spectrum (CDCl$_3$, 400 MHz, 298 K) of Ad-C3-OH.

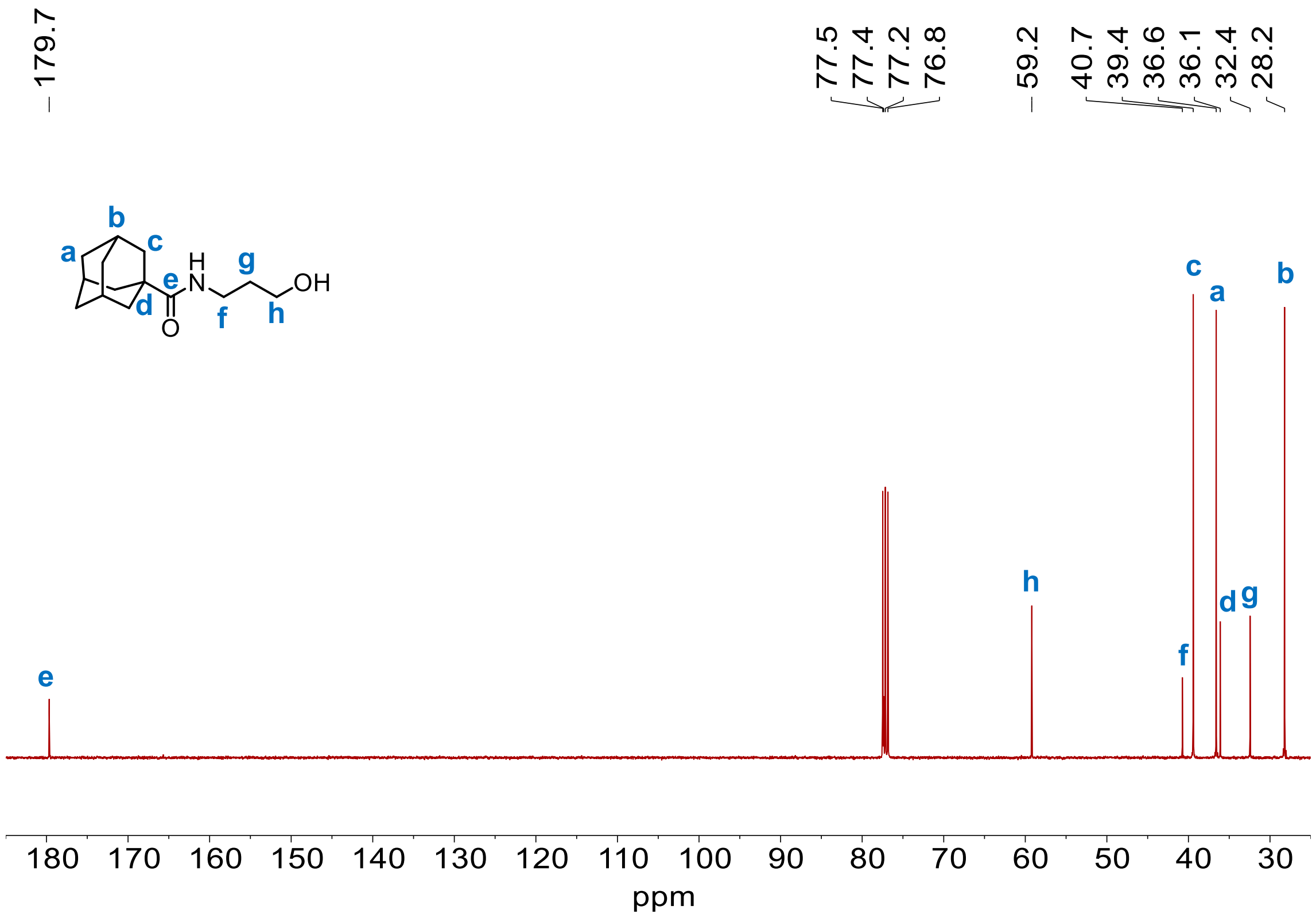

Supplementary Figure S9. ^{13}C NMR spectrum ($CDCl_3$, 101 MHz, 298 K) of Ad-C3-OH.

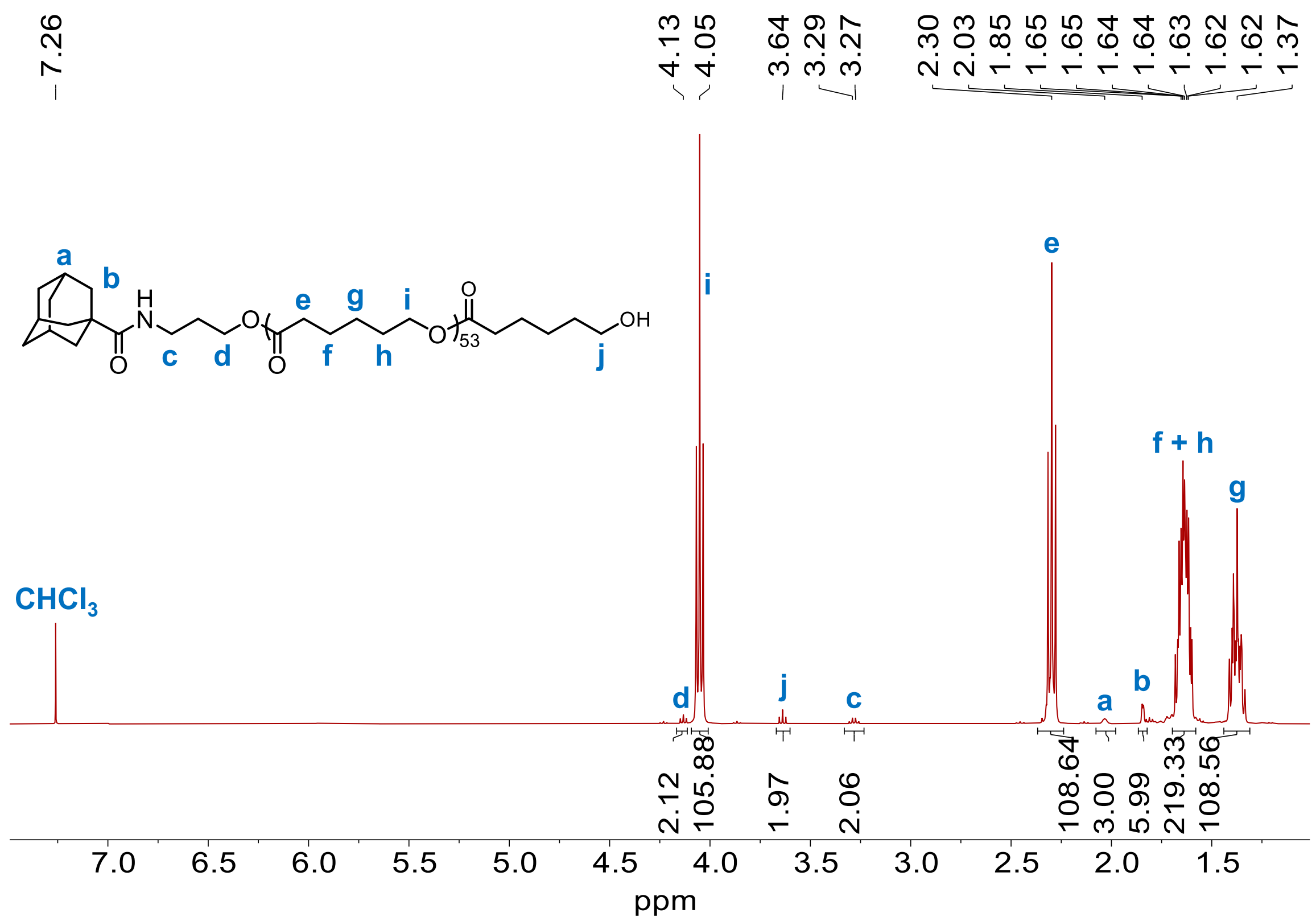

Supplementary Figure S10. ^{1}H NMR spectrum ($CDCl_3$, 400 MHz, 298 K) of PCL_{54}-Ad.

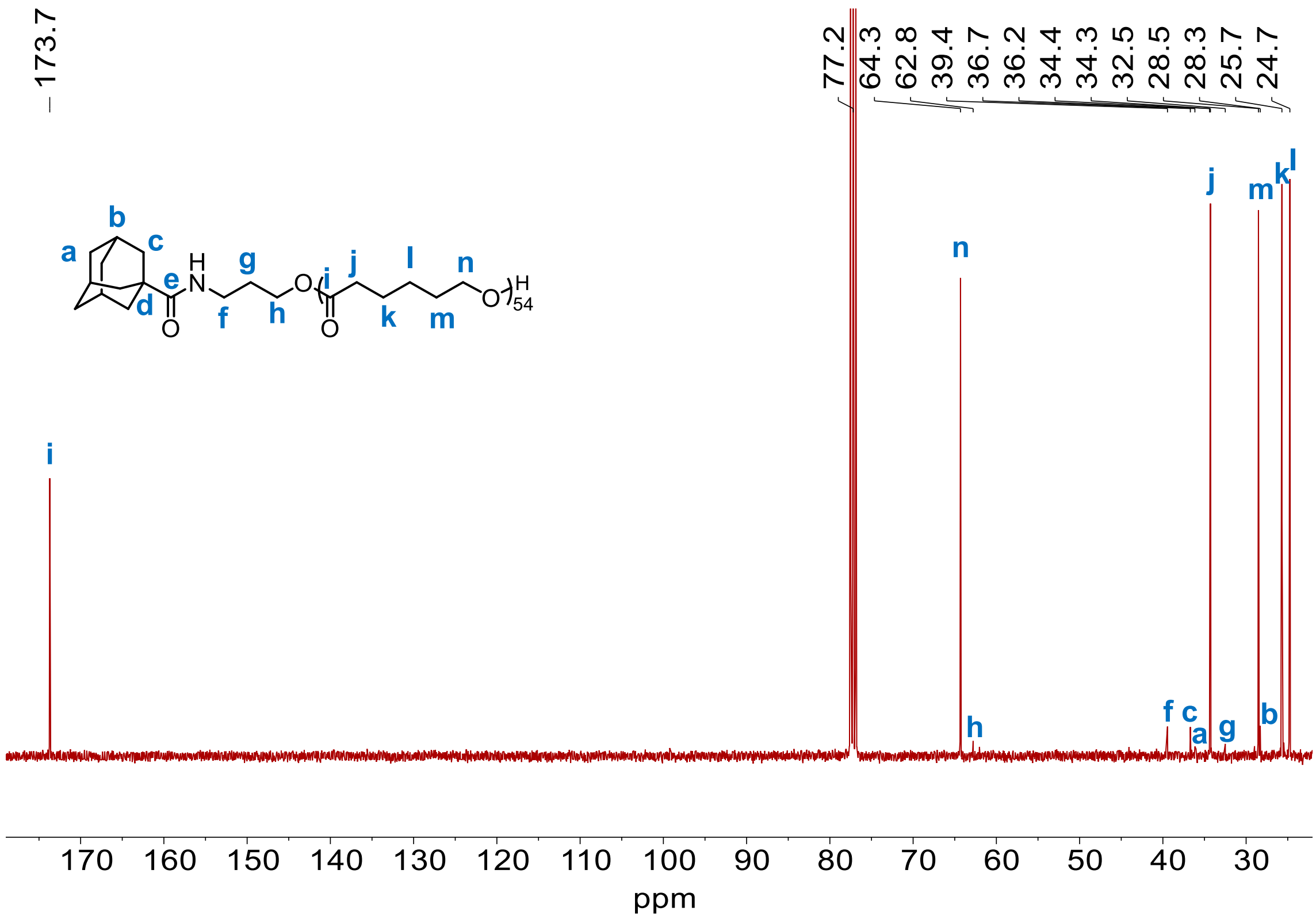

Supplementary Figure S11. ^{13}C NMR spectrum ($CDCl_3$, 101 MHz, 298 K) of PCL_{54}-Ad.

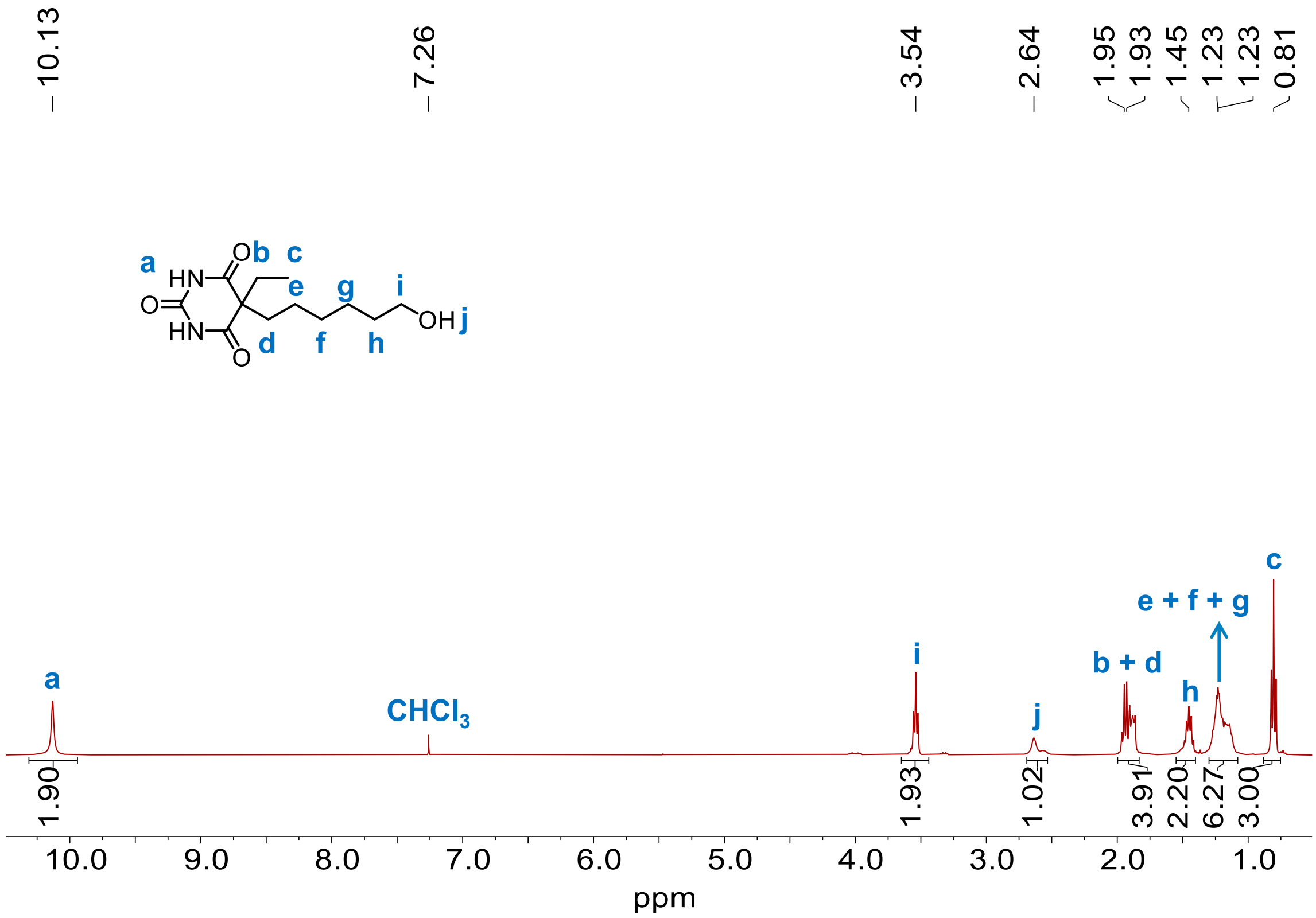

Supplementary Figure S12. ^{1}H NMR spectrum ($CDCl_3$, 400 MHz, 298 K) of BA-OH.

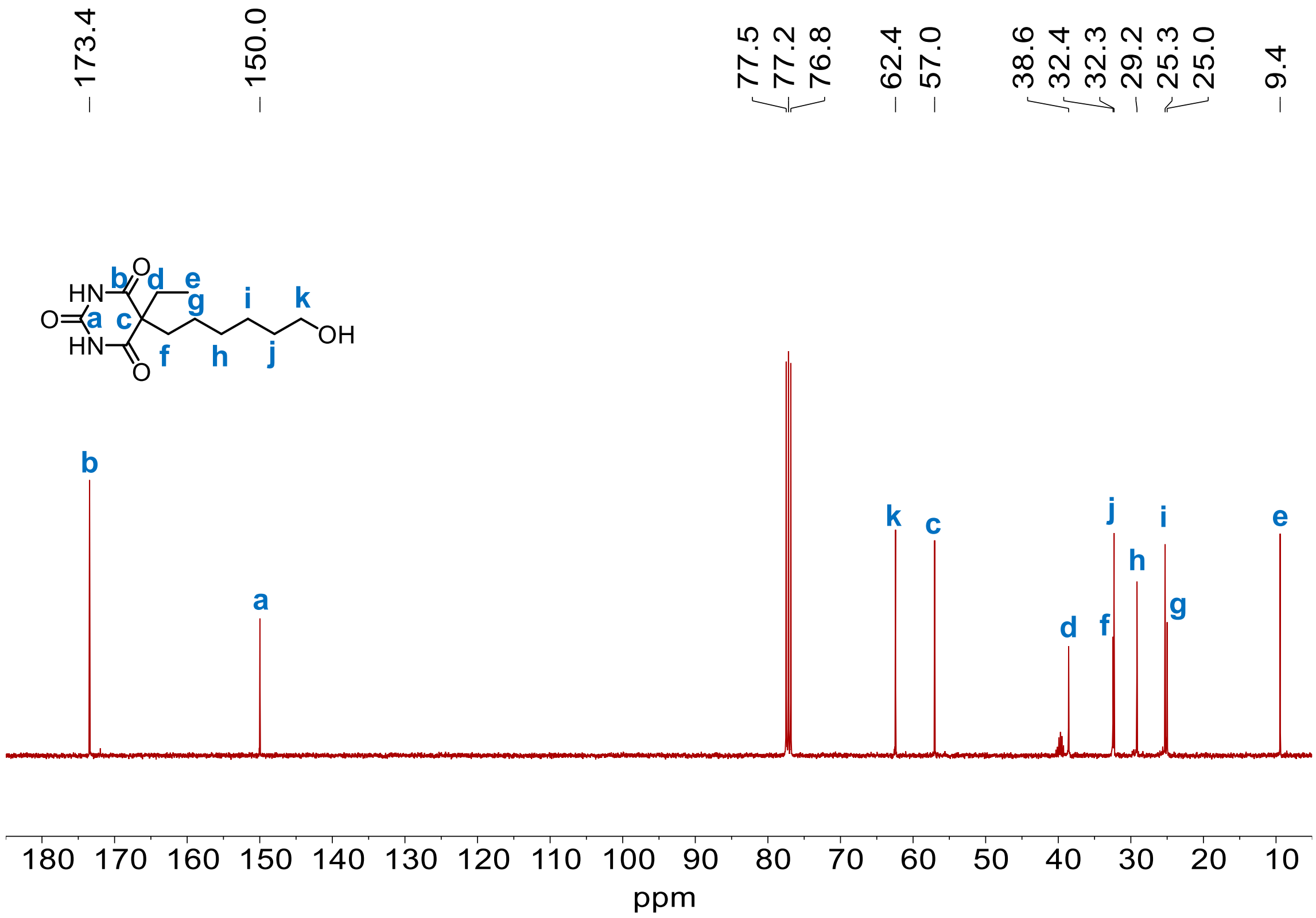

Supplementary Figure S13. ^{13}C NMR spectrum ($CDCl_3$, 101 MHz, 298 K) of BA-OH.

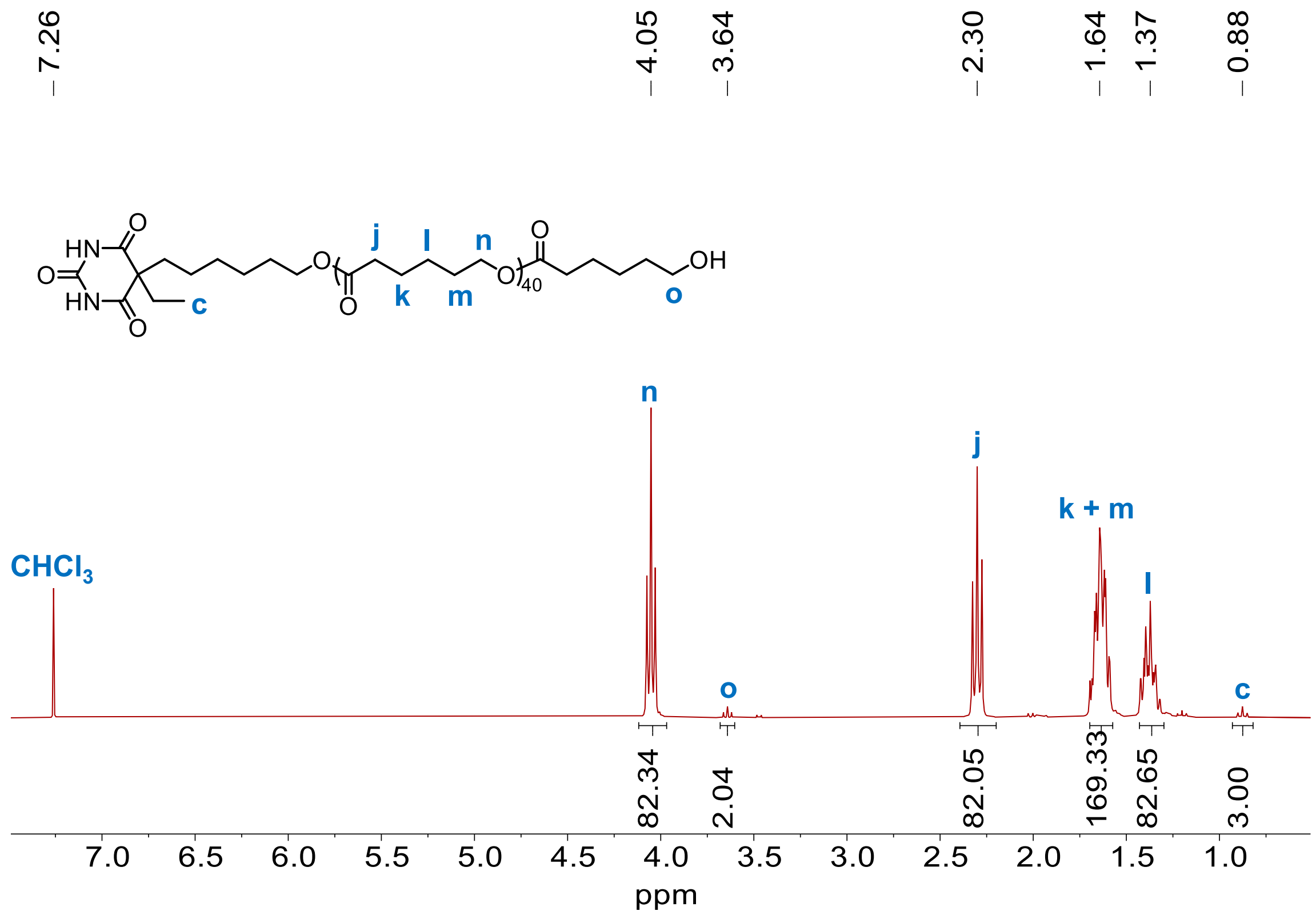

Supplementary Figure S14. ^{1}H NMR spectrum ($CDCl_3$, 400 MHz, 298 K) of PCL_{41}-BA.

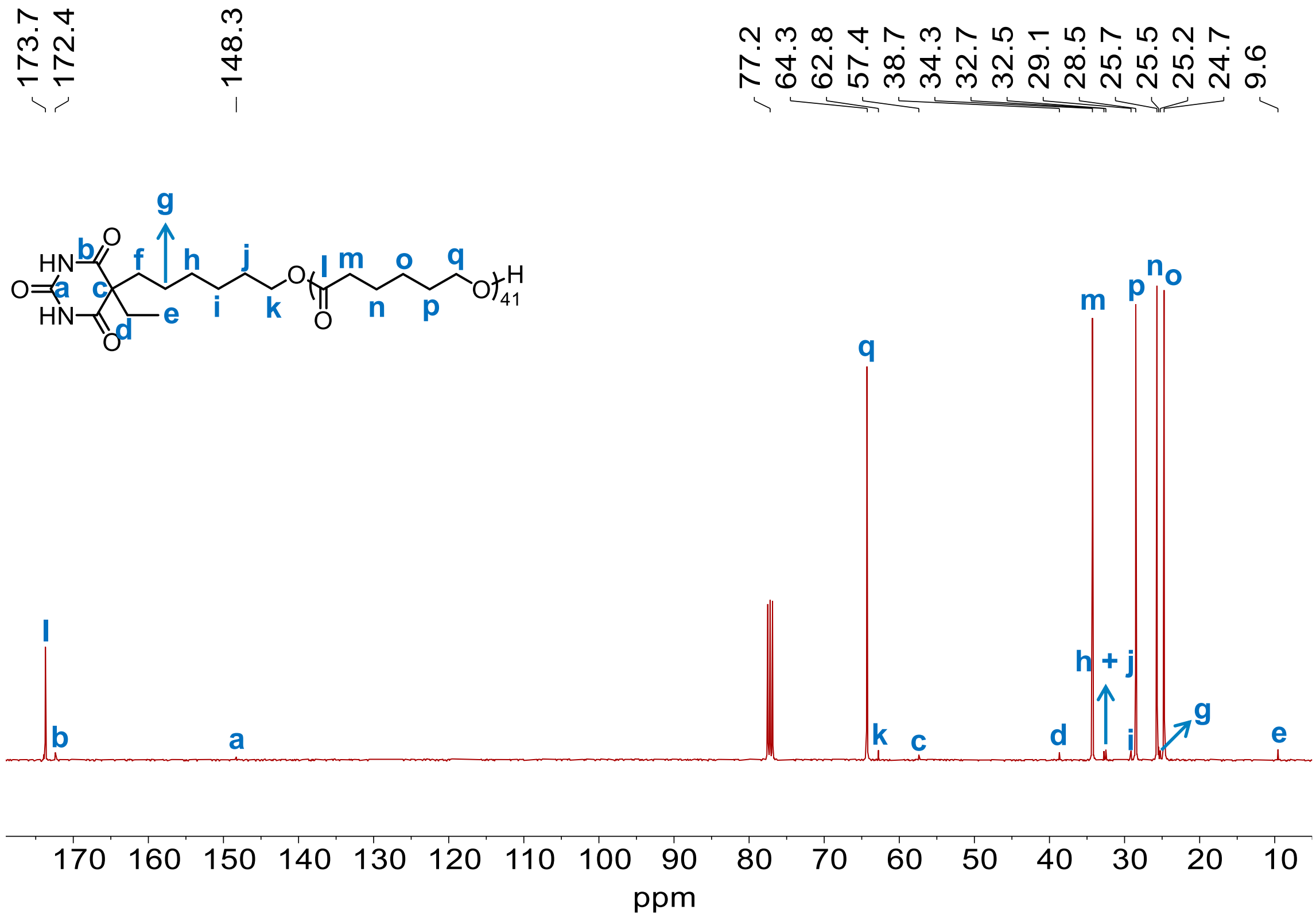

Supplementary Figure S15. ^{13}C NMR spectrum ($CDCl_3$, 101 MHz, 298 K) of PCL_{41}-BA.

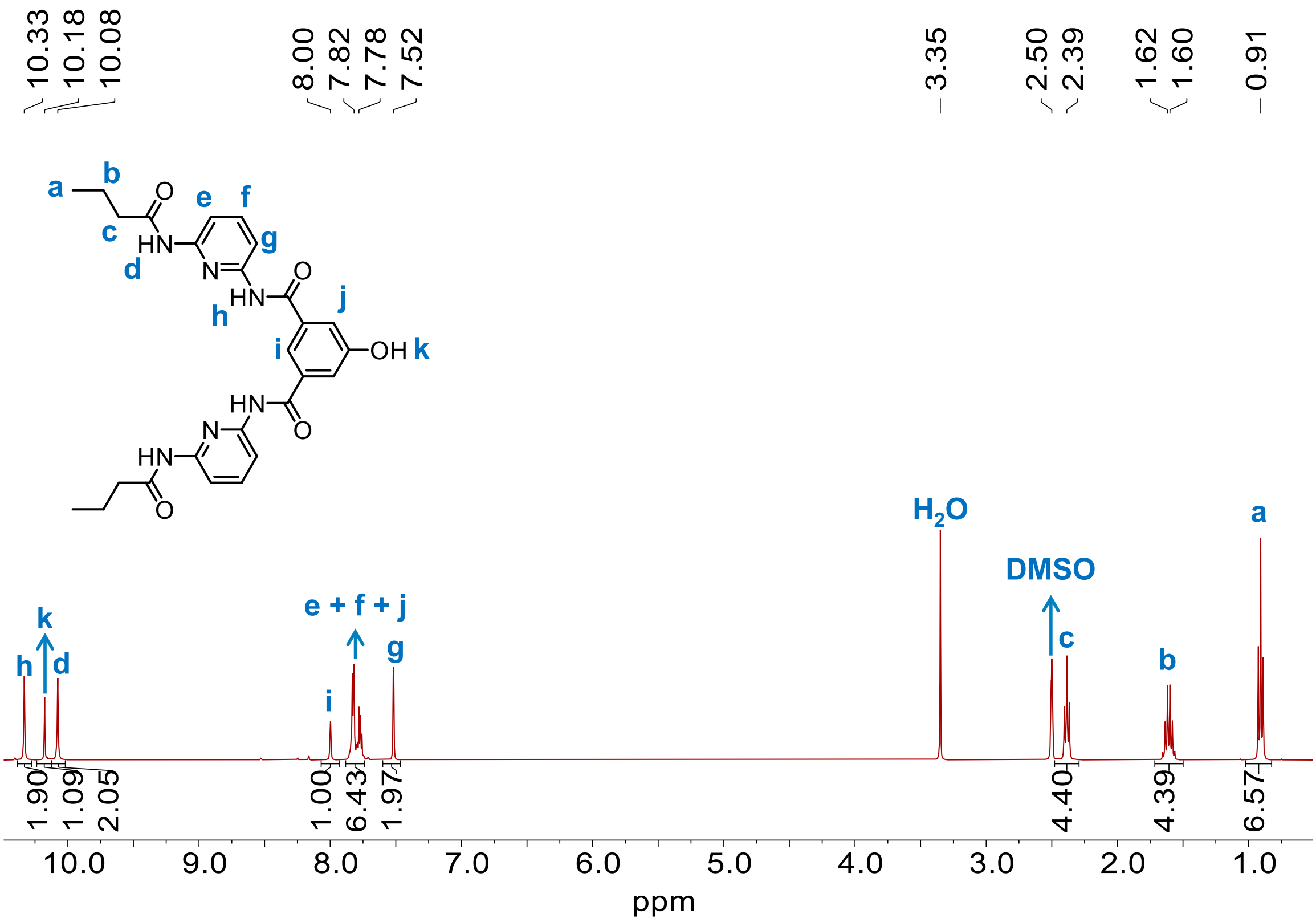

Supplementary Figure S16. ^{1}H NMR spectrum (DMSO-d_6, 400 MHz, 298 K) of HW-PhOH.

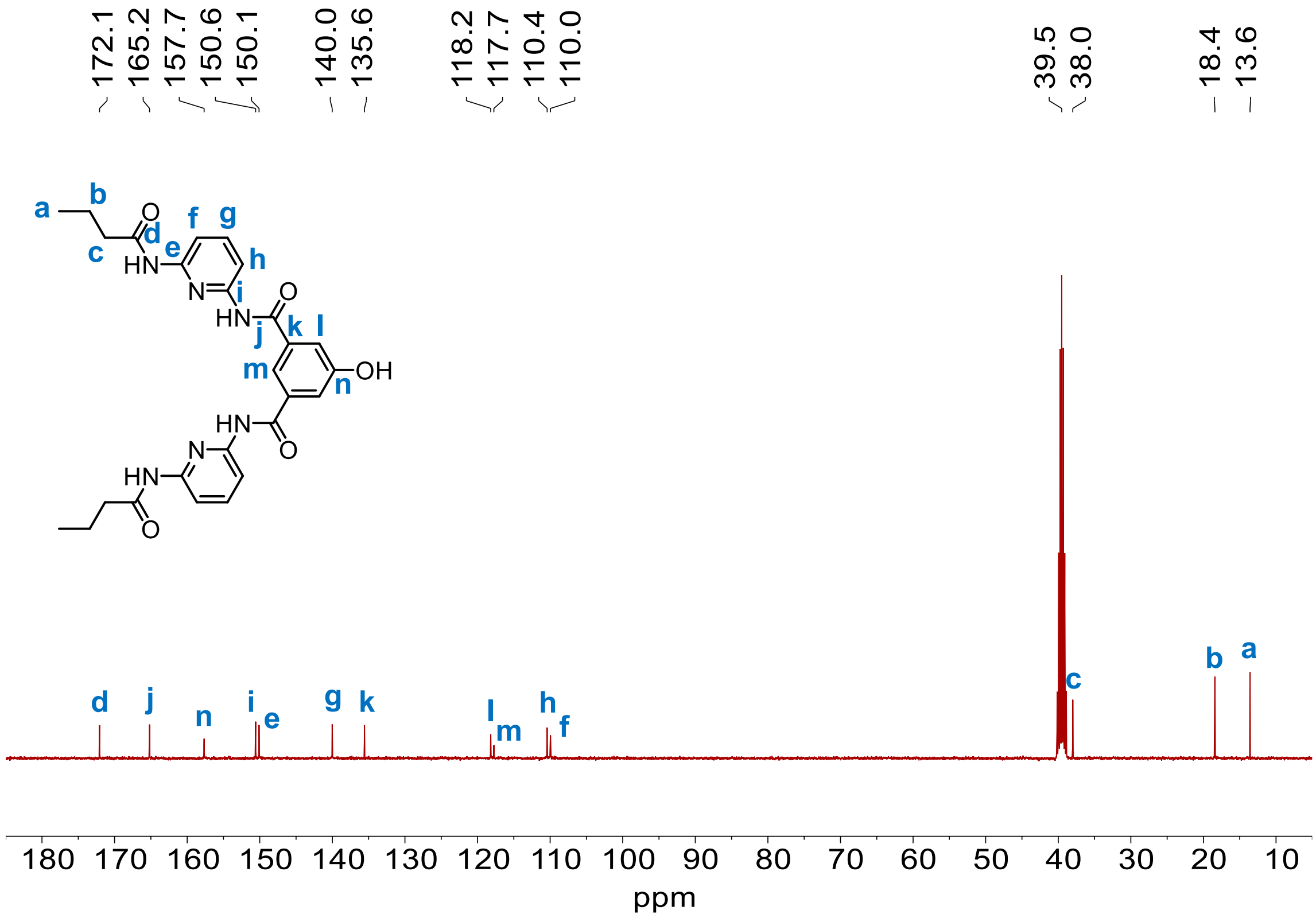

Supplementary Figure S17. ^{13}C NMR spectrum (DMSO-d_6, 101 MHz, 298 K) of HW-PhOH.

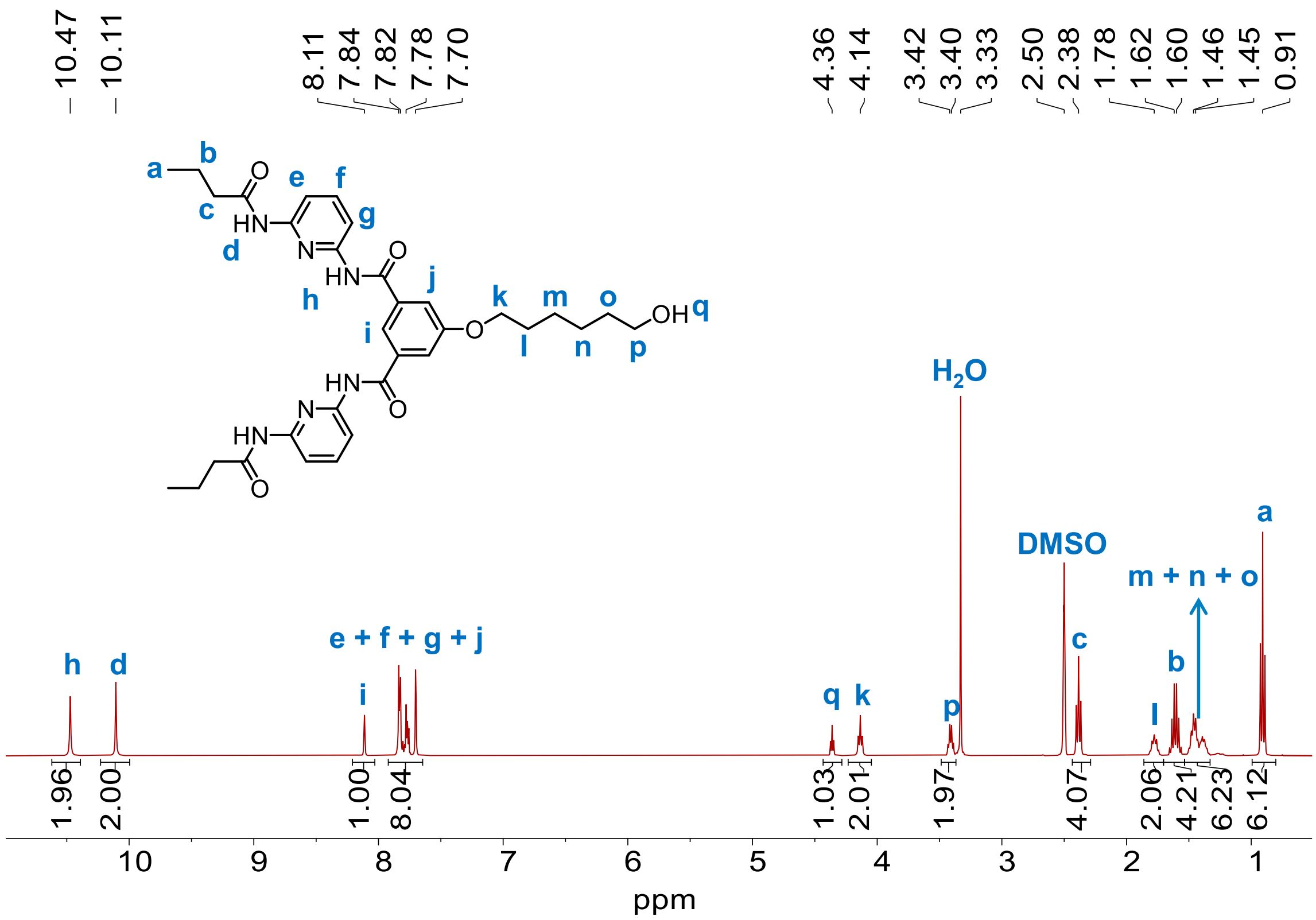

Supplementary Figure S18. ^{1}H NMR spectrum (DMSO-d_6, 400 MHz, 298 K) of HW-OH.

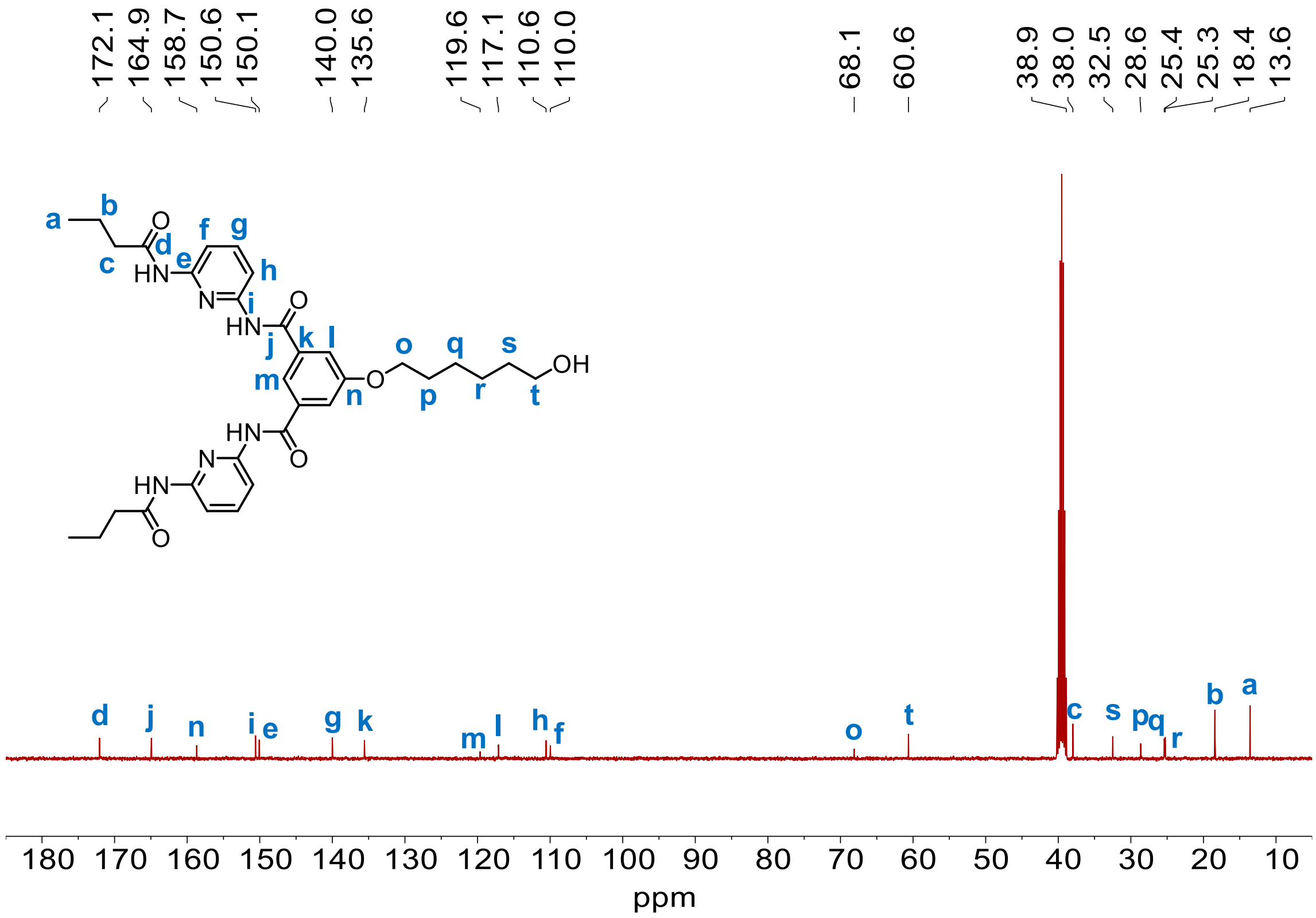

Supplementary Figure S19. ^{13}C NMR spectrum (DMSO-d_6, 101 MHz, 298 K) of HW-OH.

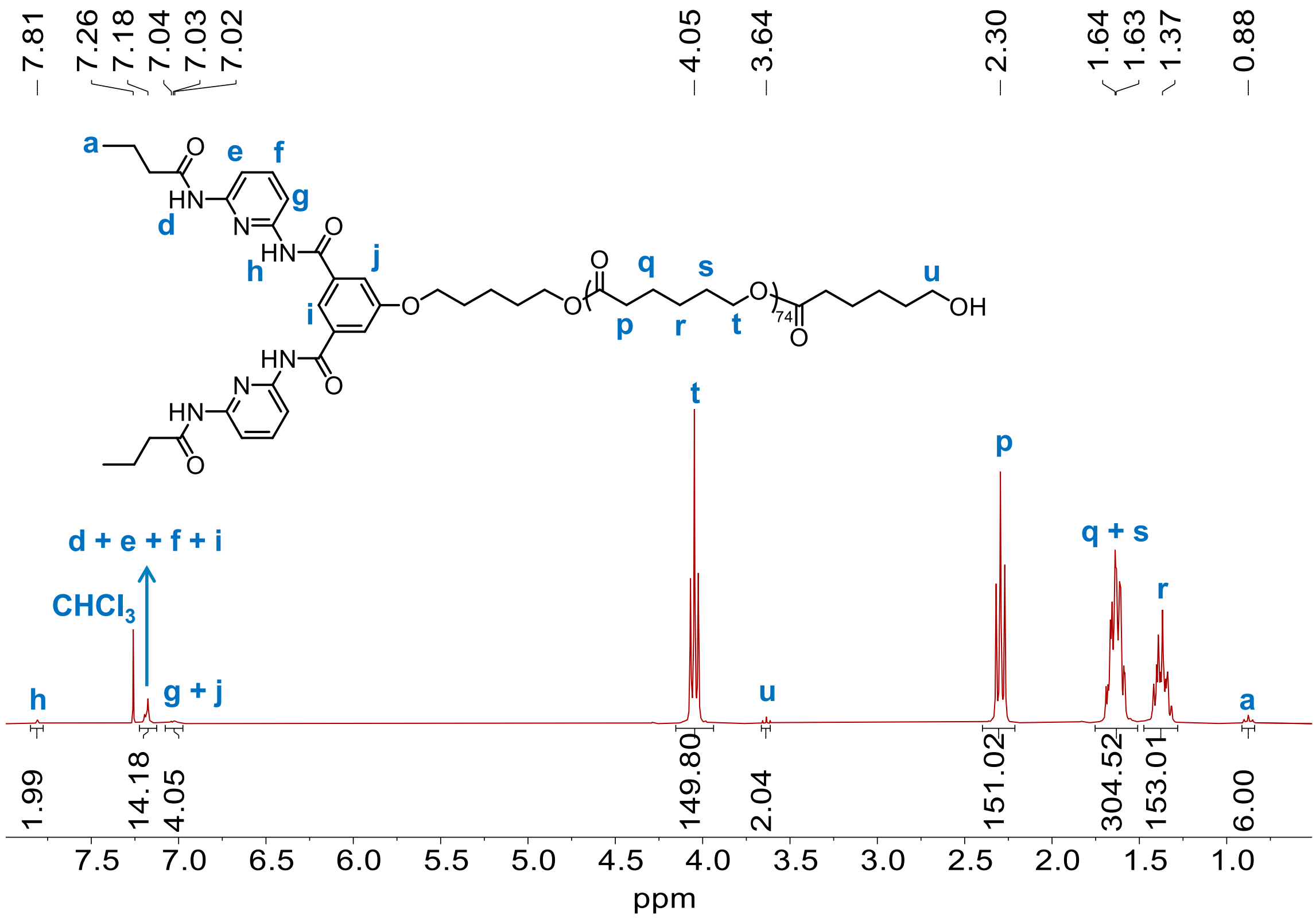

Supplementary Figure S20. ^{1}H NMR spectrum ($CDCl_3$, 400 MHz, 298 K) of PCL_{75}-HW.

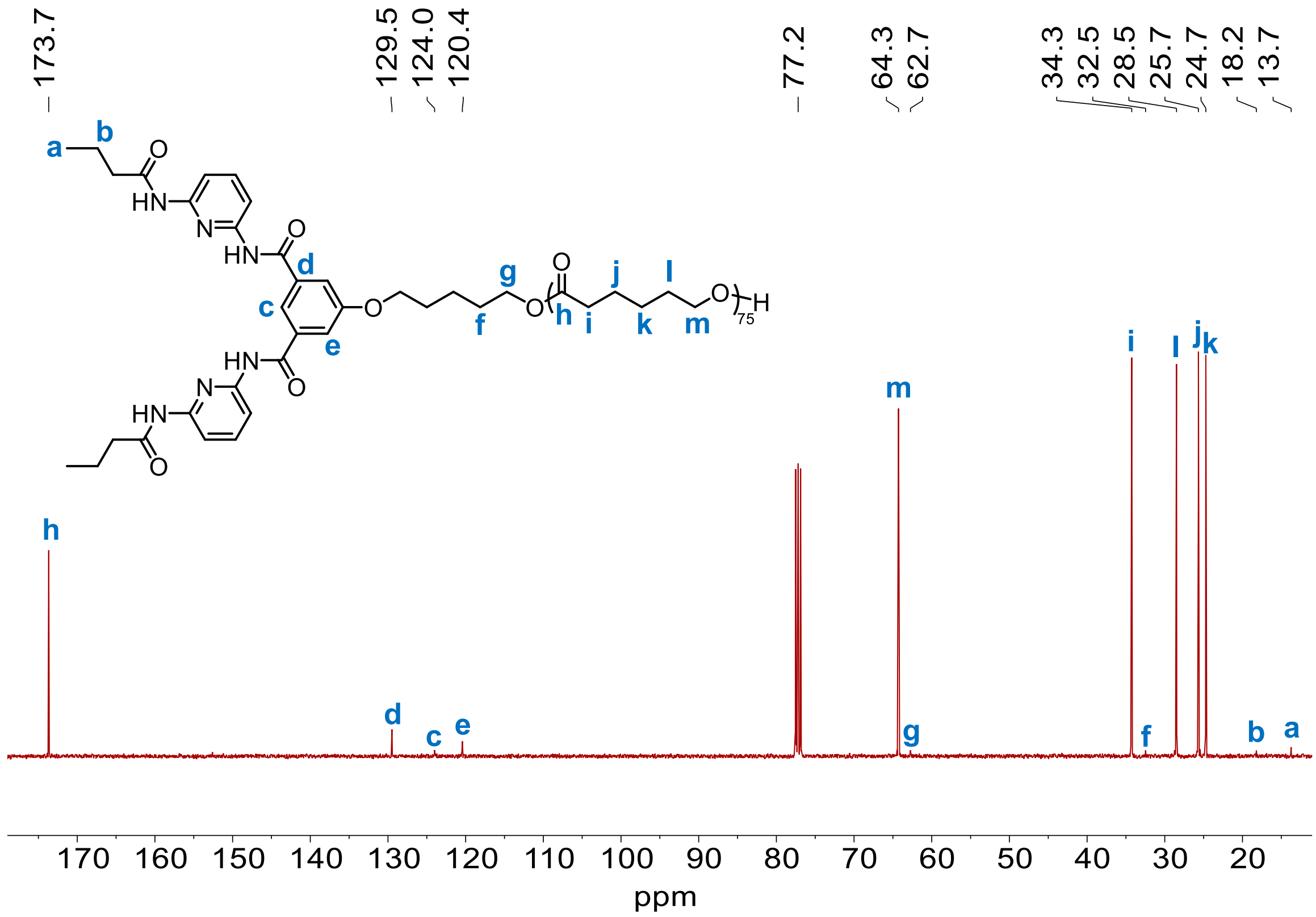

Supplementary Figure S21. ^{13}C NMR spectrum ($CDCl_3$, 101 MHz, 298 K) of PCL_{75}-HW.

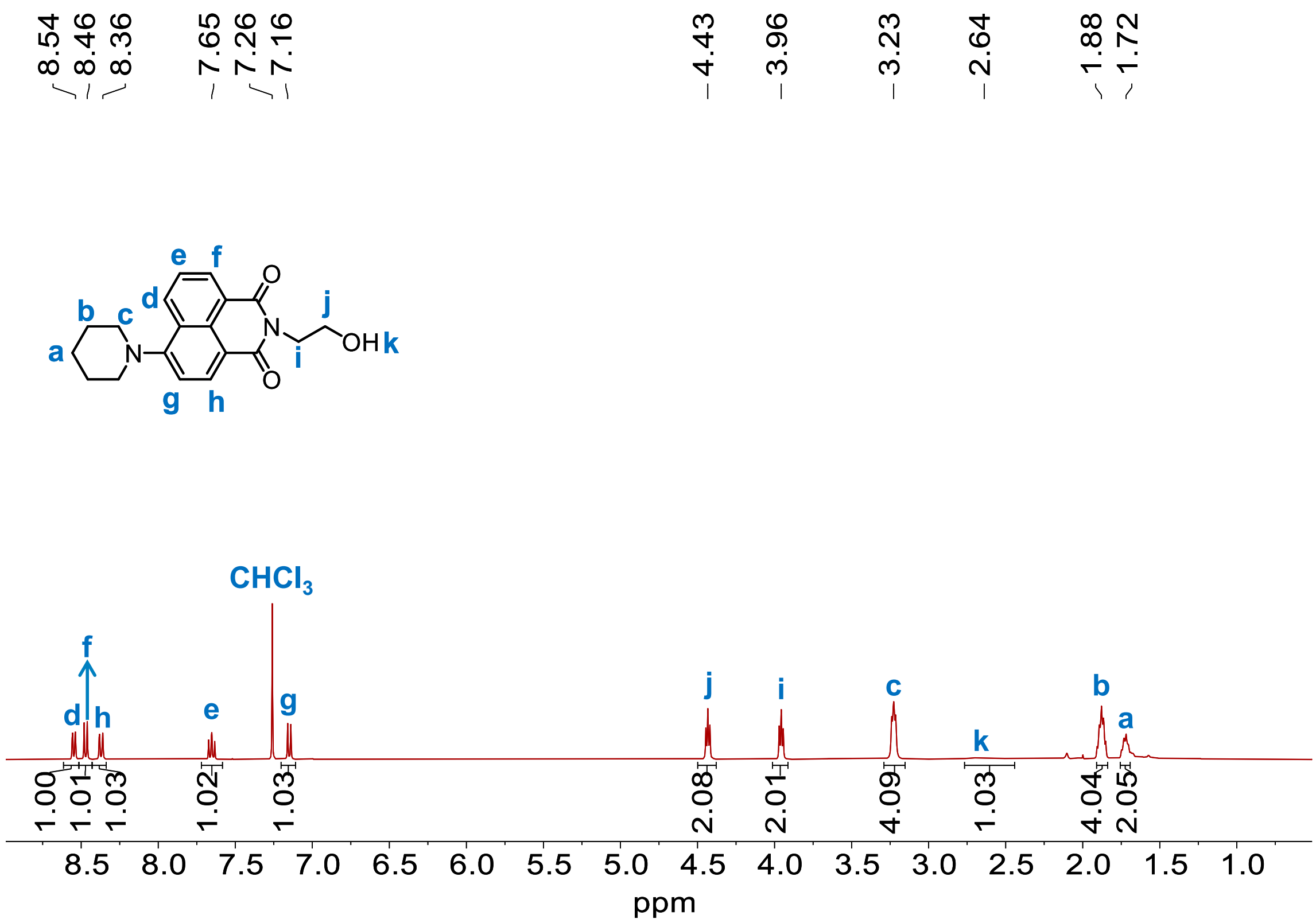

Supplementary Figure S22. ^{1}H NMR spectrum ($CDCl_3$, 400 MHz, 298 K) of NMI-OH.

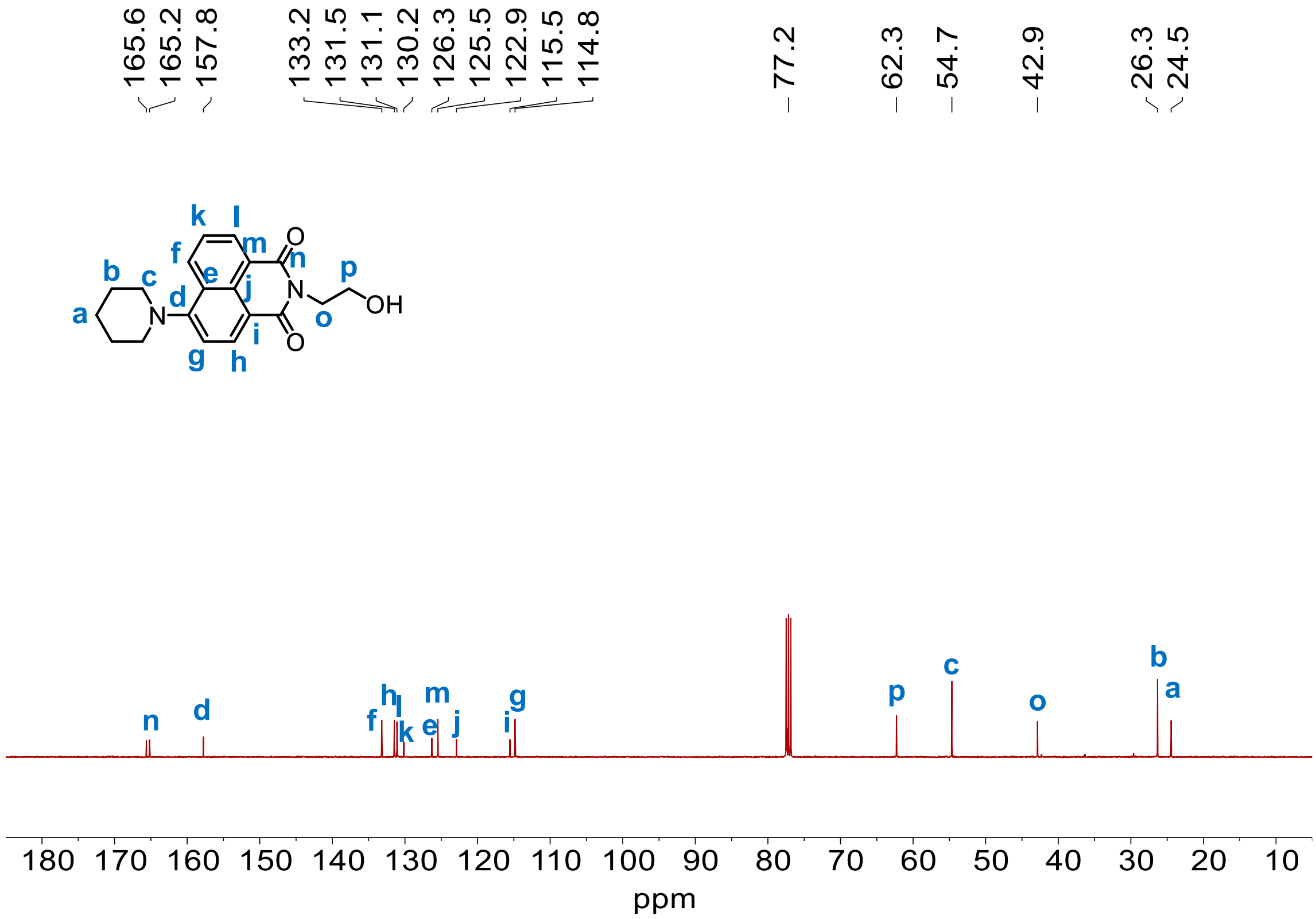

Supplementary Figure S23. ^{13}C NMR spectrum ($CDCl_3$, 101 MHz, 298 K) of NMI-OH.

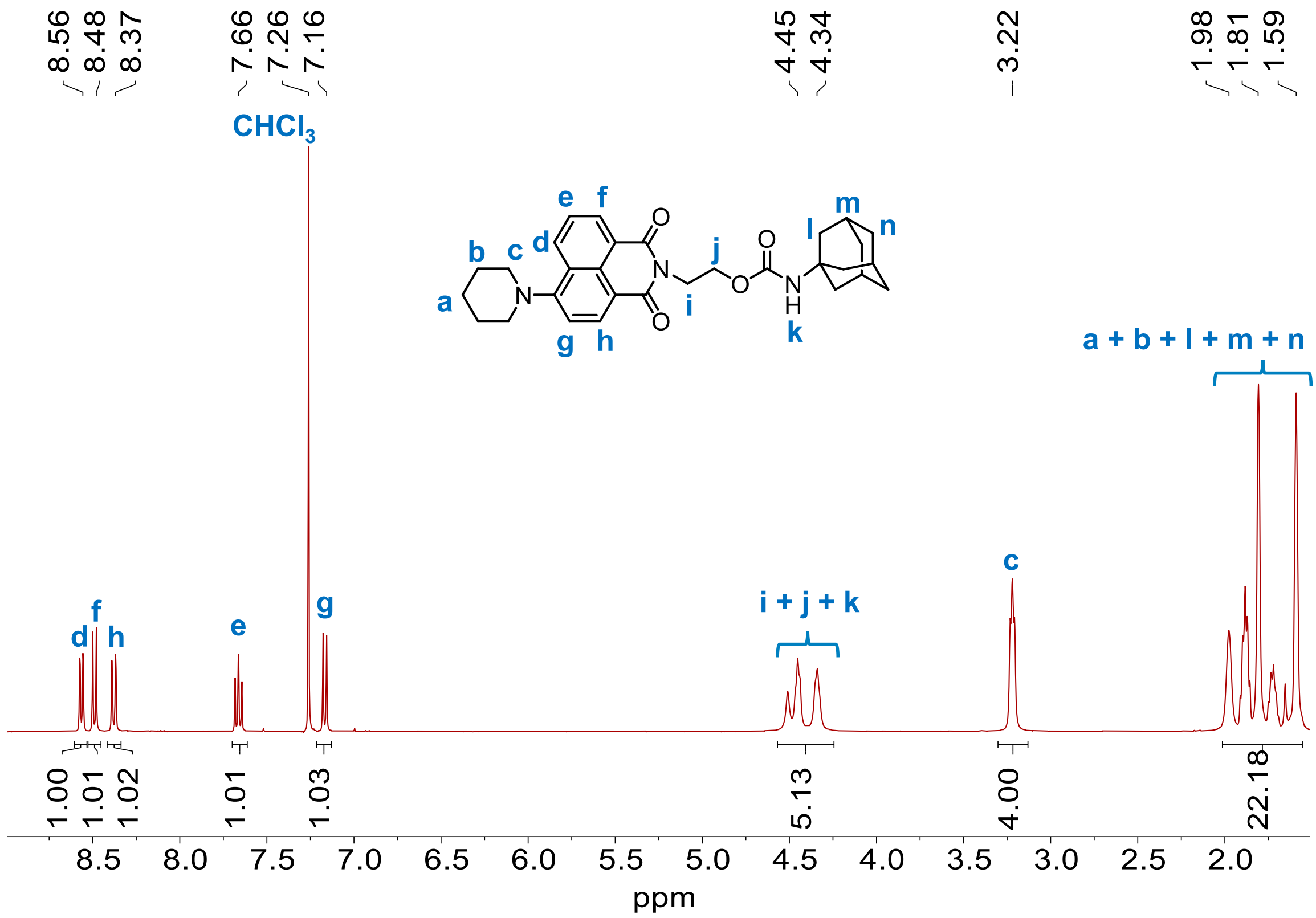

Supplementary Figure S24. ^{1}H NMR spectrum ($CDCl_3$, 400 MHz, 298 K) of NMI-Ad.

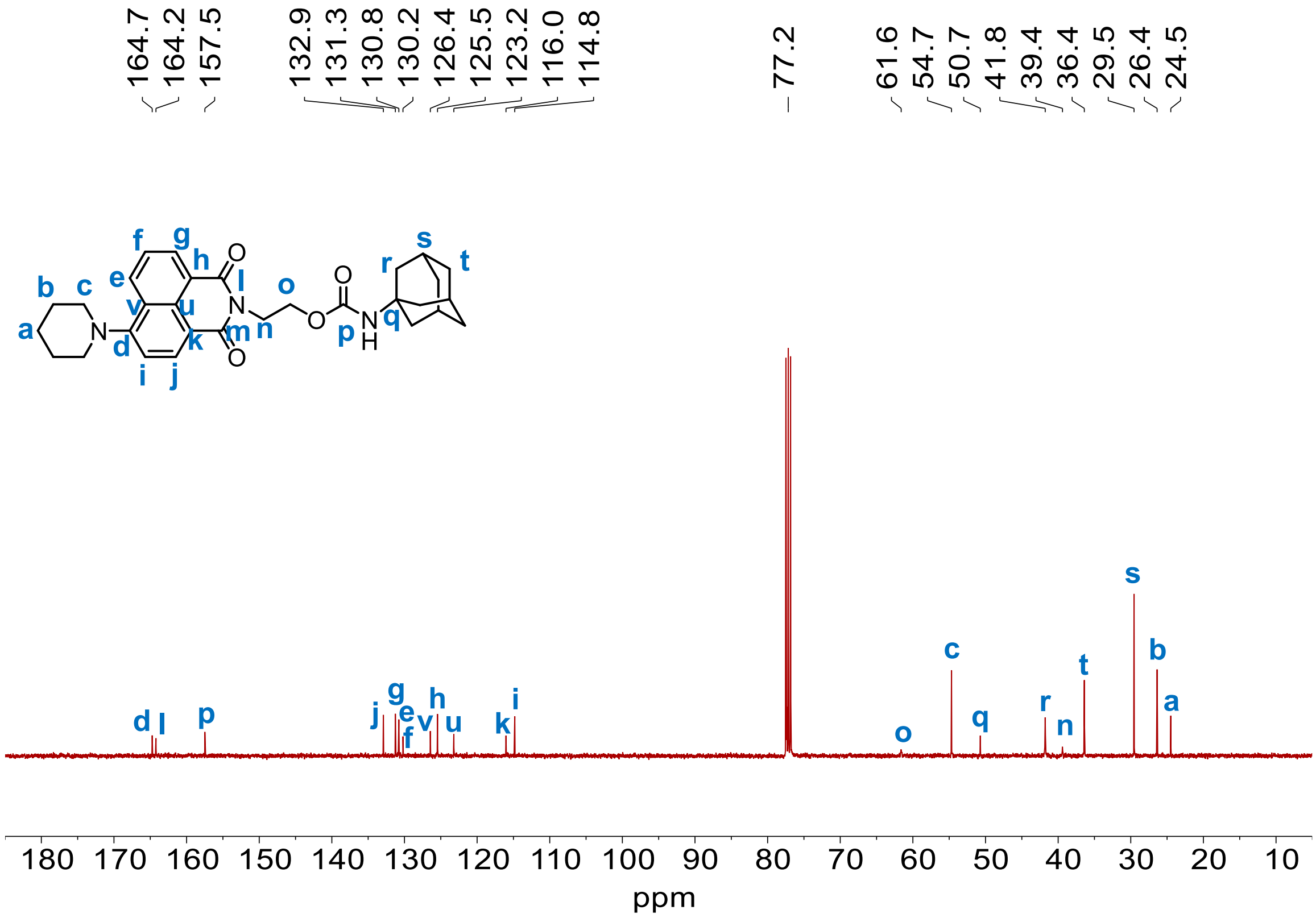

Supplementary Figure S25. ^{13}C NMR spectrum ($CDCl_3$, 101 MHz, 298 K) of NMI-Ad.

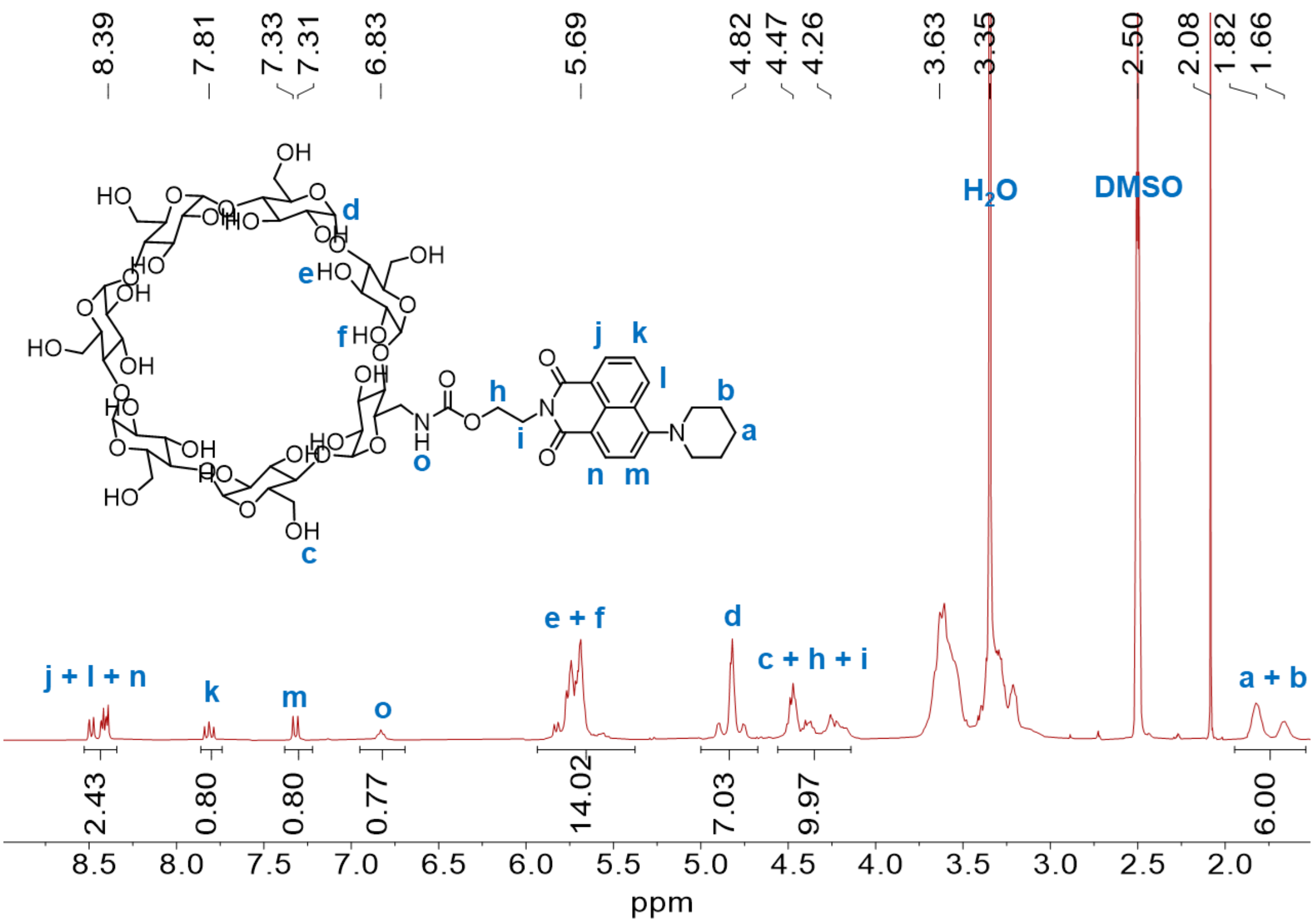

Supplementary Figure S26. ^{1}H NMR spectrum (DMSO-d_6, 400 MHz, 298 K) of NMI-CD.

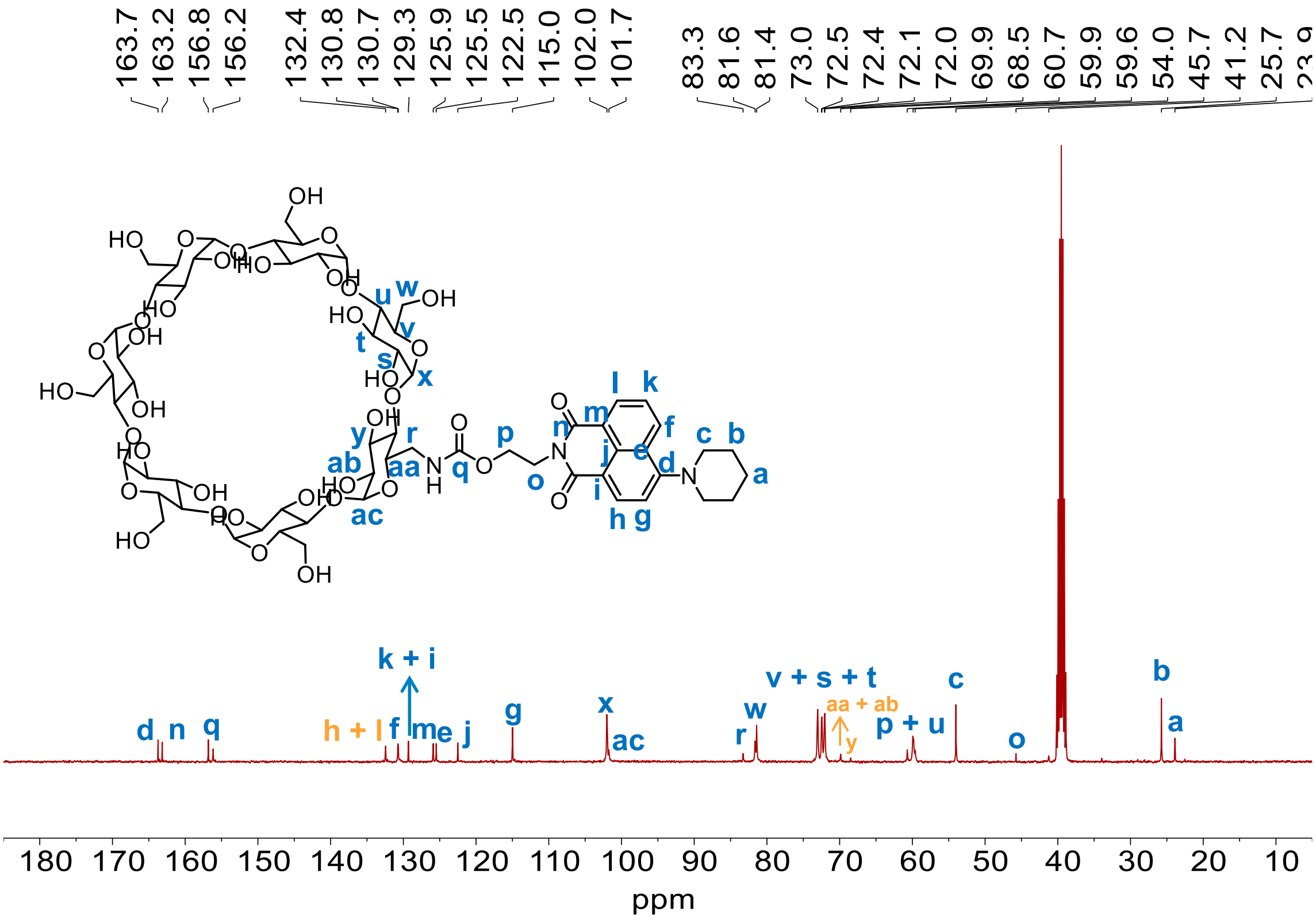

Supplementary Figure S27. ^{13}C NMR spectrum (DMSO-d_6, 101 MHz, 298 K) of NMI-CD.

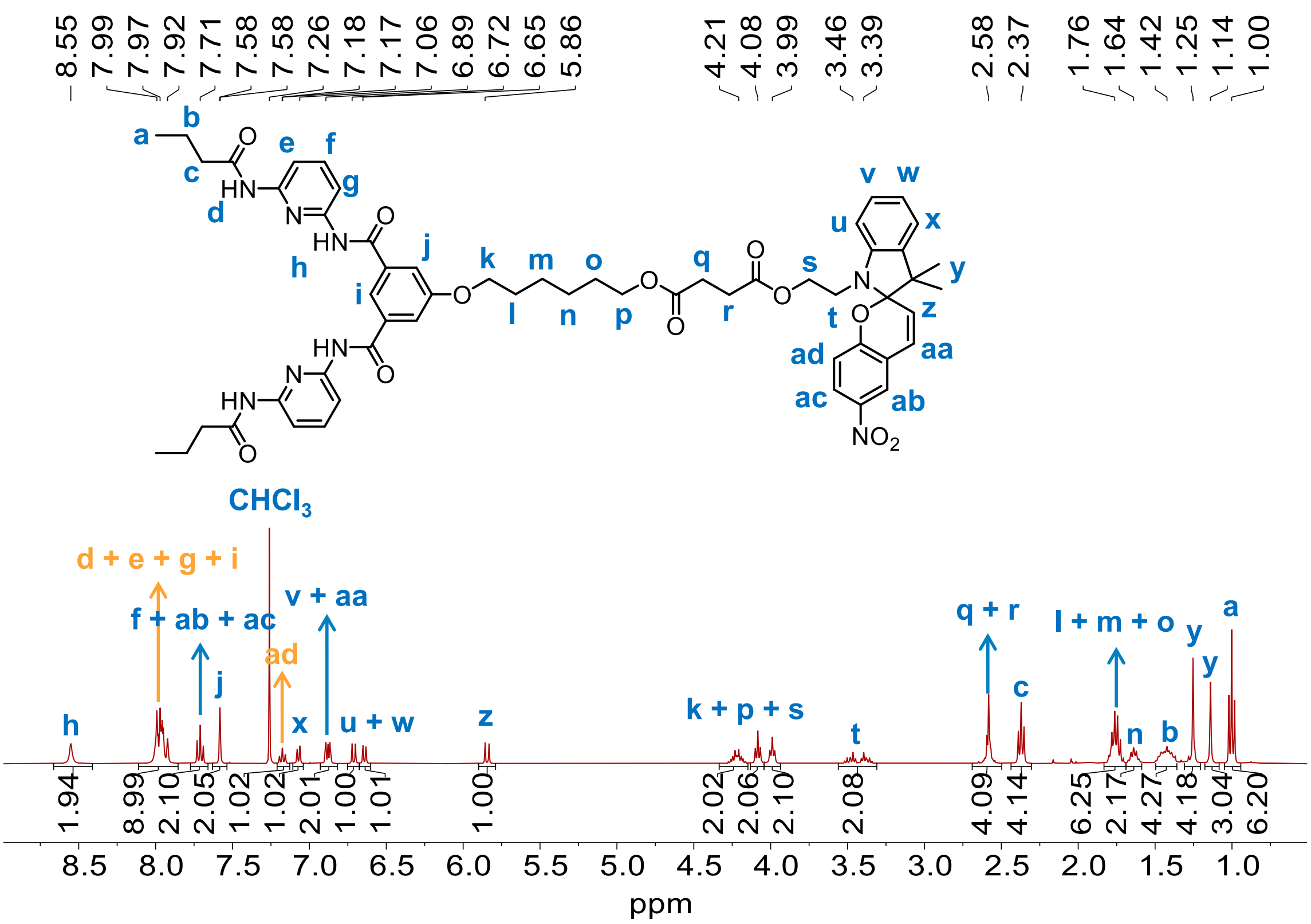

Supplementary Figure S28. ^{1}H NMR spectrum ($CDCl_3$, 400 MHz, 298 K) of SPY-HW.

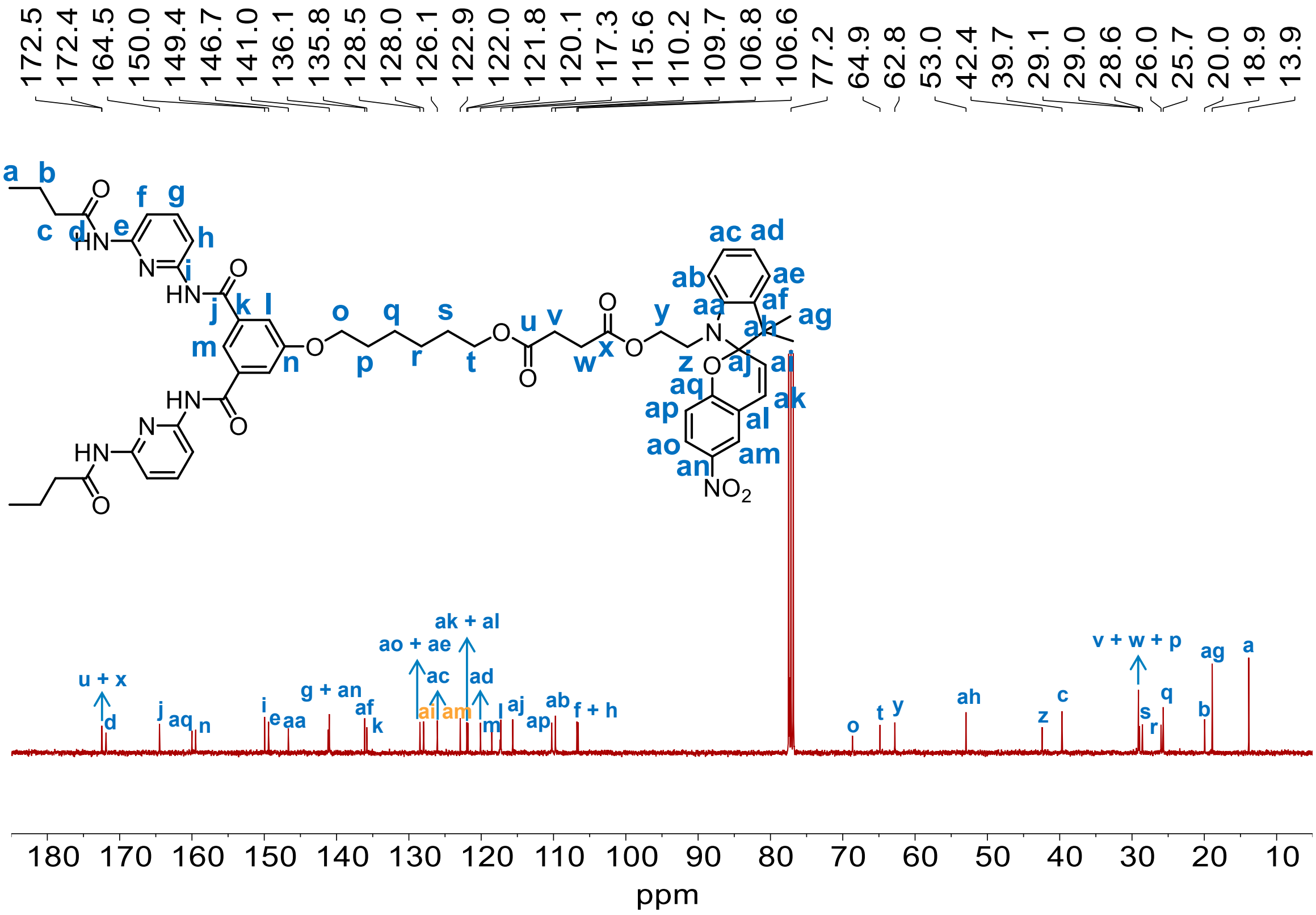

Supplementary Figure S29. ^{13}C NMR spectrum ($CDCl_3$, 101 MHz, 298 K) of SPY-HW.

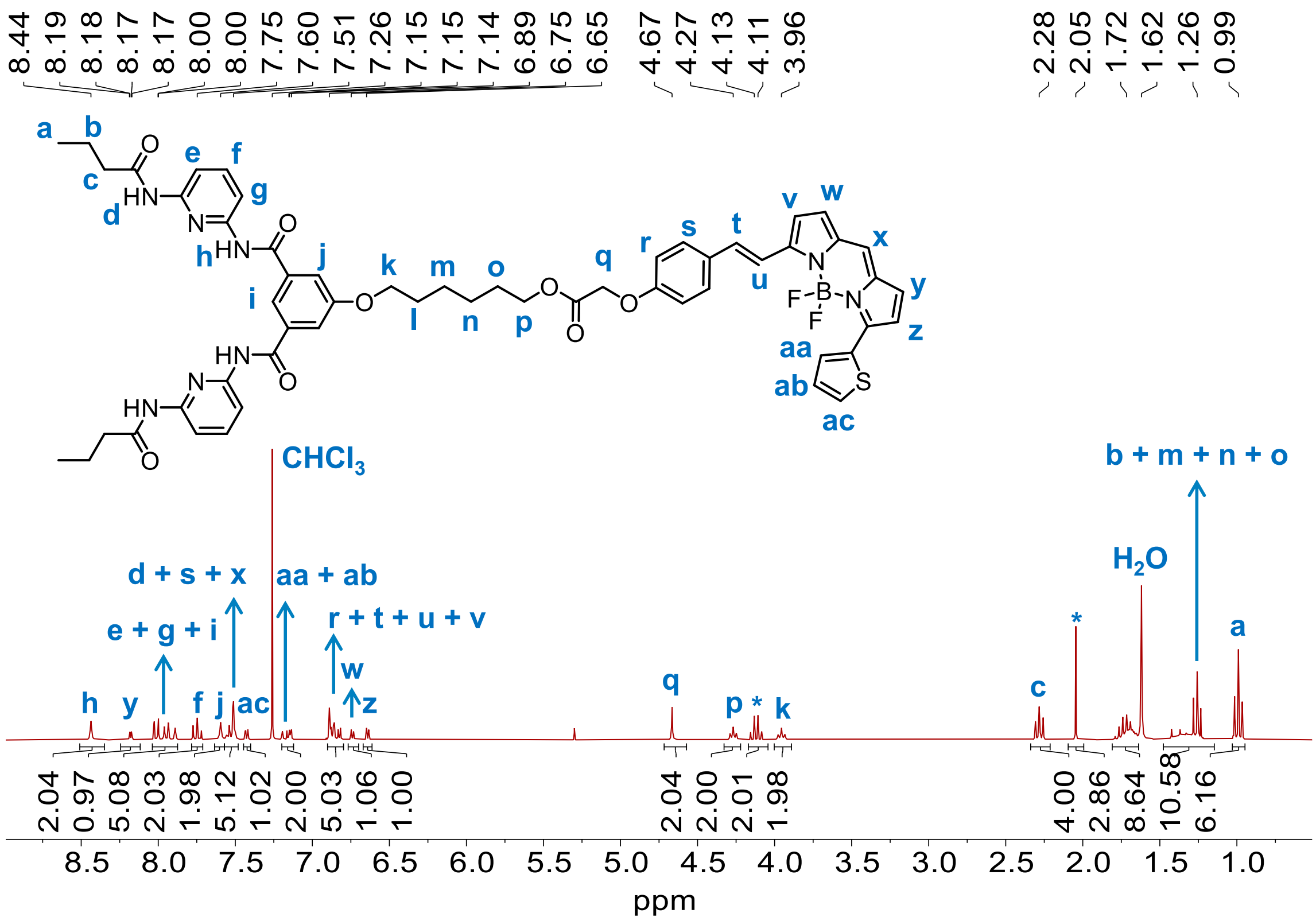

Supplementary Figure S30. ^{1}H NMR spectrum ($CDCl_3$, 400 MHz, 298 K) of BDP-HW. Residual EA peaks are marked with an asterisk.

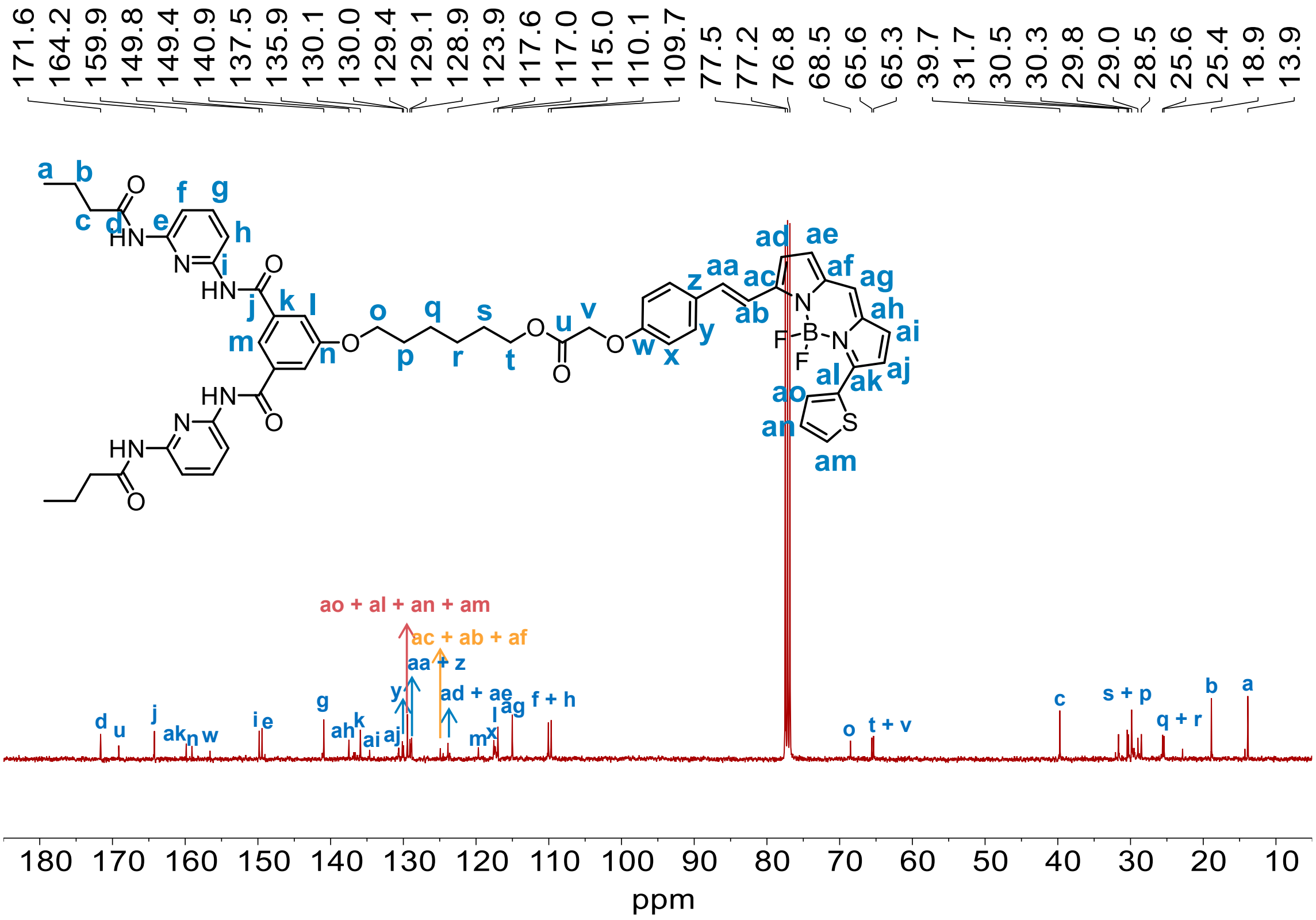

Supplementary Figure S31. ^{13}C NMR spectrum ($CDCl_3$, 101 MHz, 298 K) of BDP-HW.

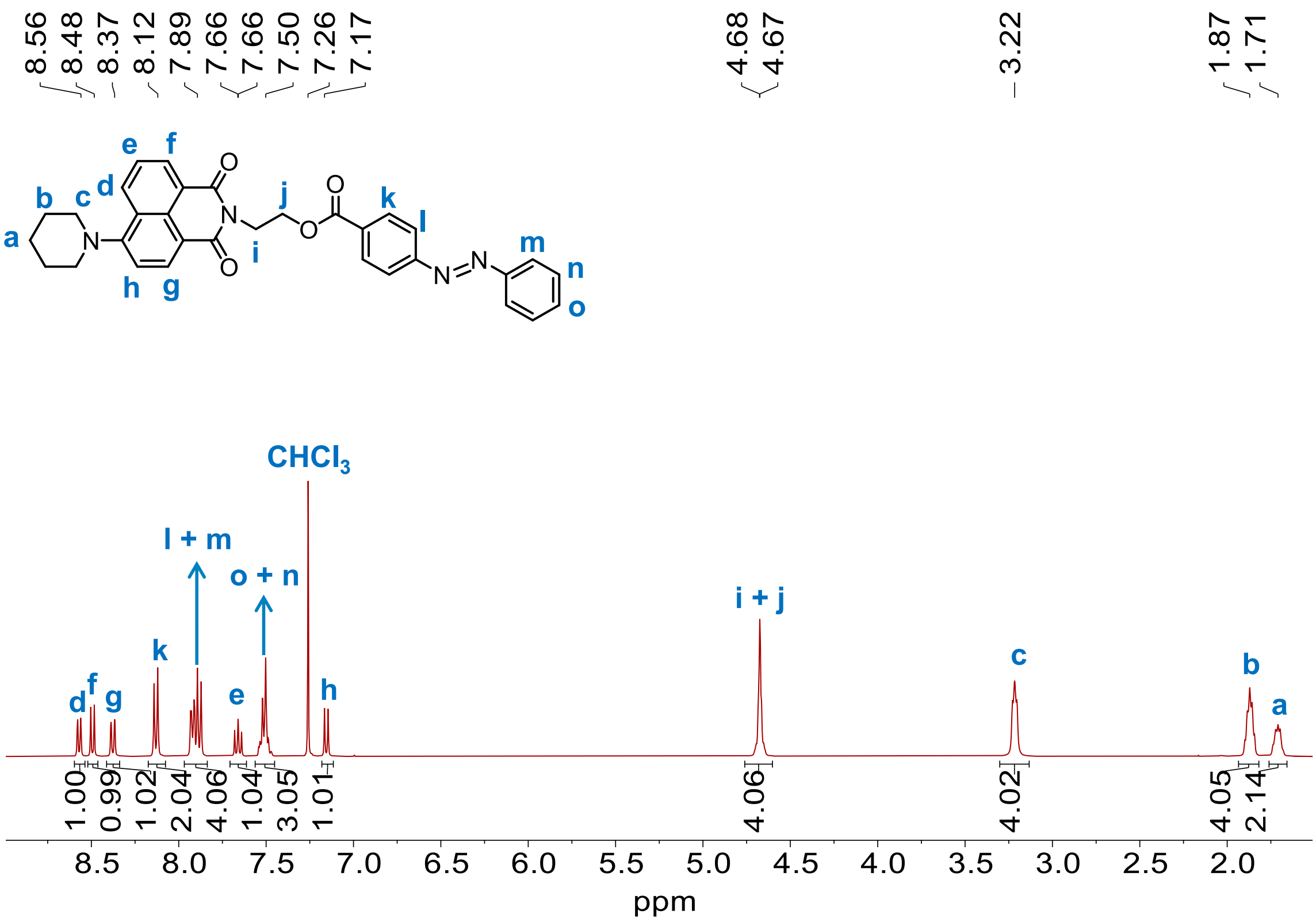

Supplementary Figure S32. ^{1}H NMR spectrum ($CDCl_3$, 400 MHz, 298 K) of NMI-Azo.

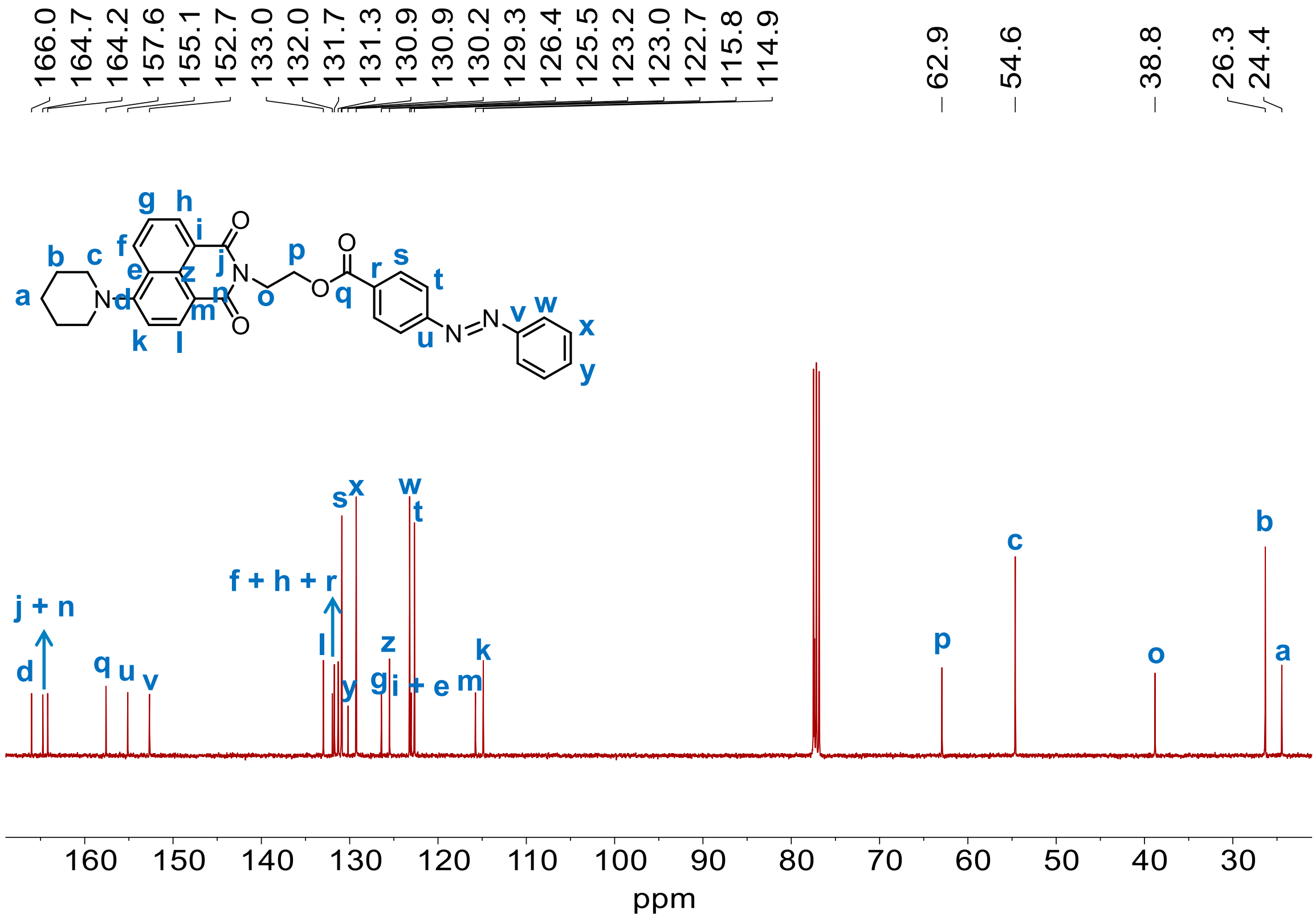

Supplementary Figure S33. ^{13}C NMR spectrum ($CDCl_3$, 101 MHz, 298 K) of NMI-Azo.

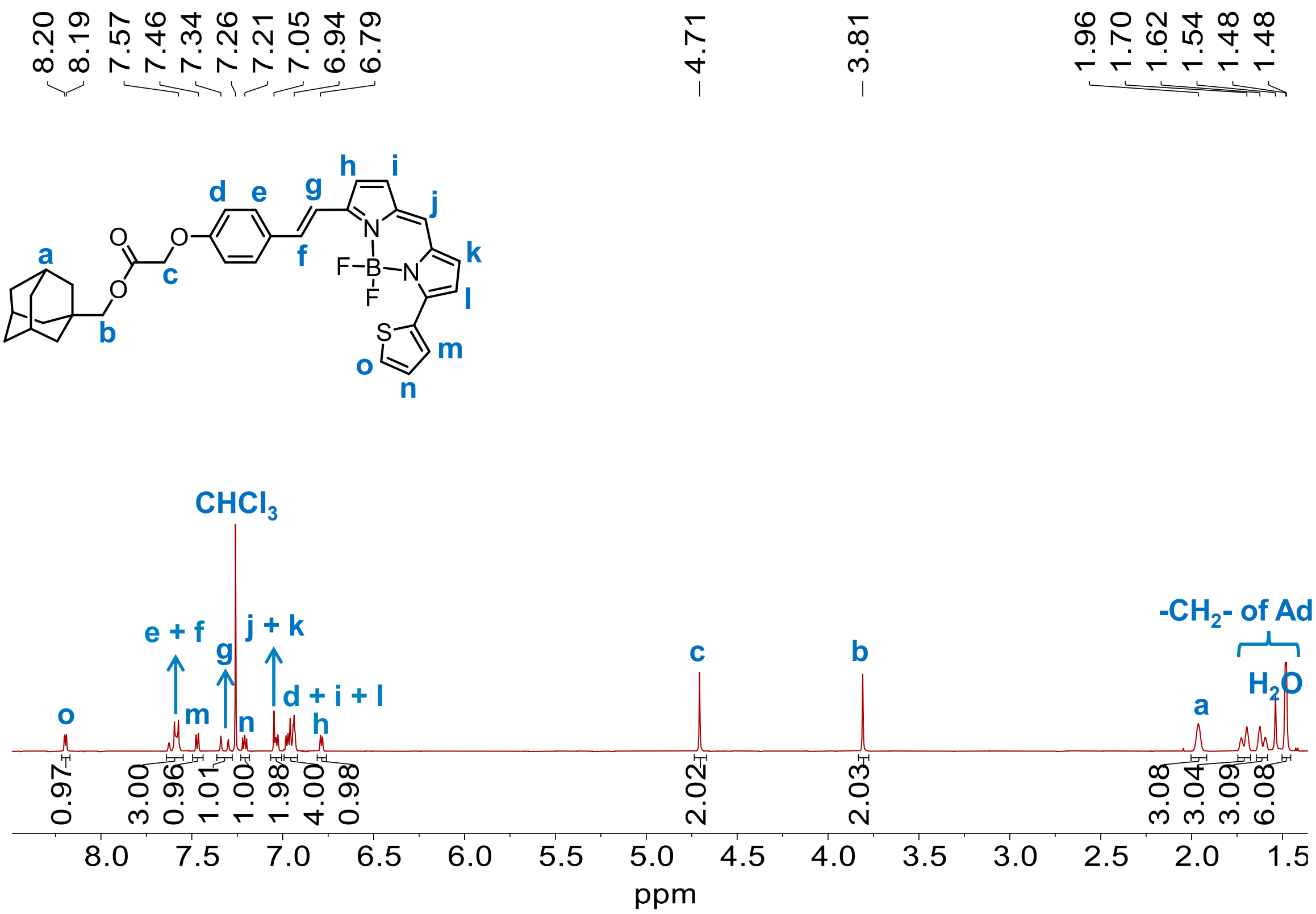

Supplementary Figure S34. ^{1}H NMR spectrum ($CDCl_3$, 400 MHz, 298 K) of BDP-Ad.

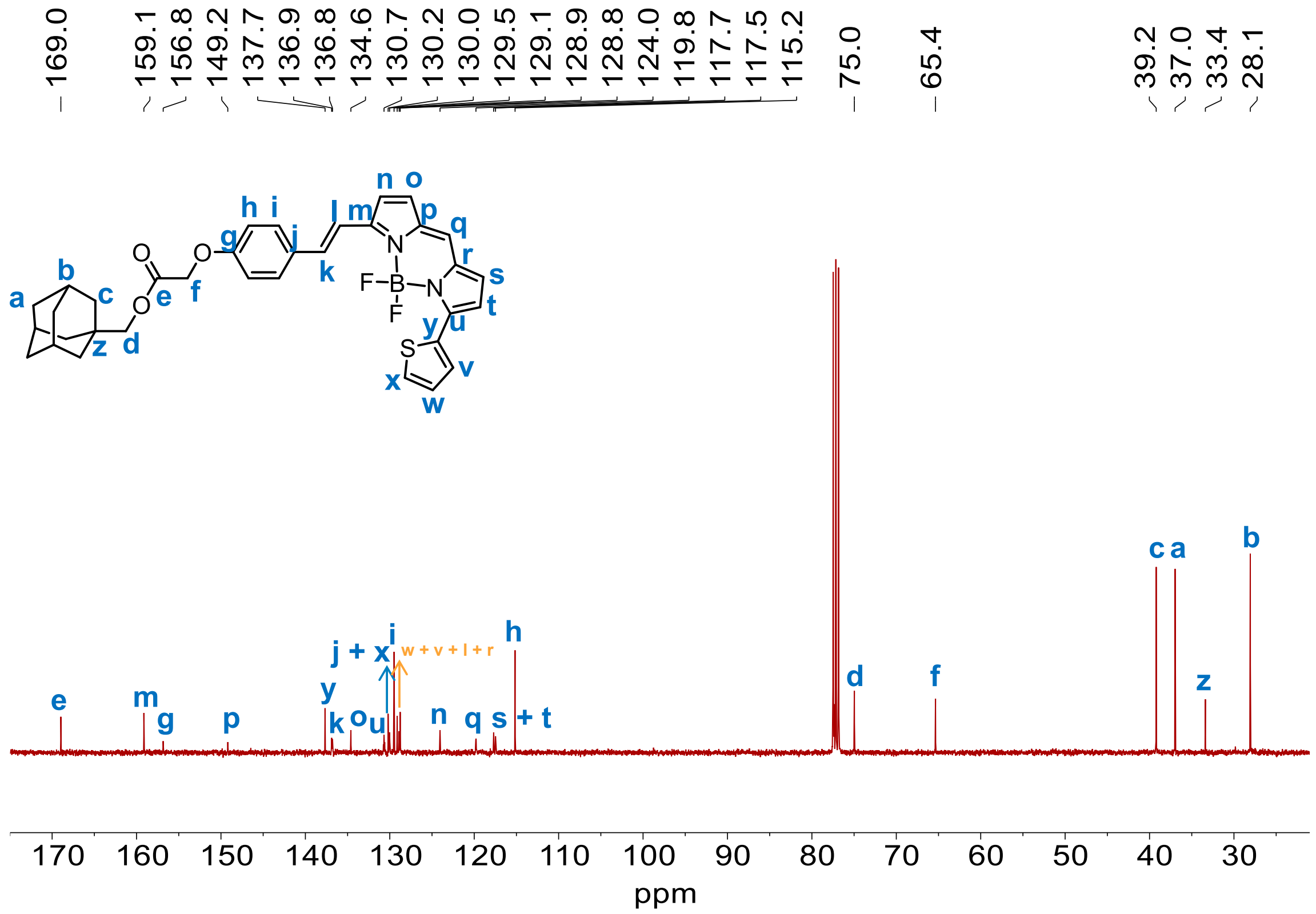

Supplementary Figure S35. ^{13}C NMR spectrum ($CDCl_3$, 101 MHz, 298 K) of BDP-Ad.

Mass spectrometry

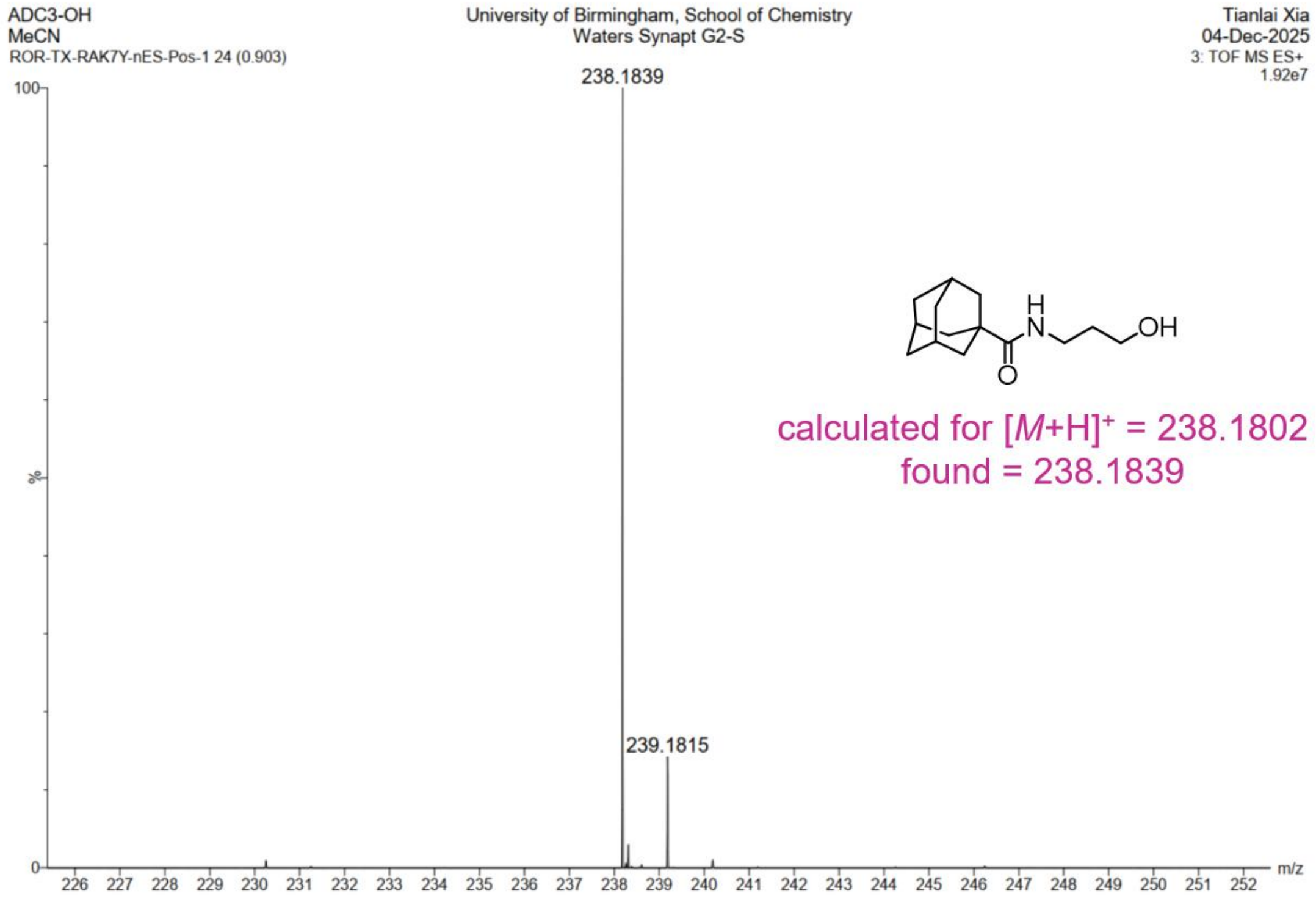

Supplementary Figure S36. Positive mode ESI-MS spectrum of Ad-C3-OH.

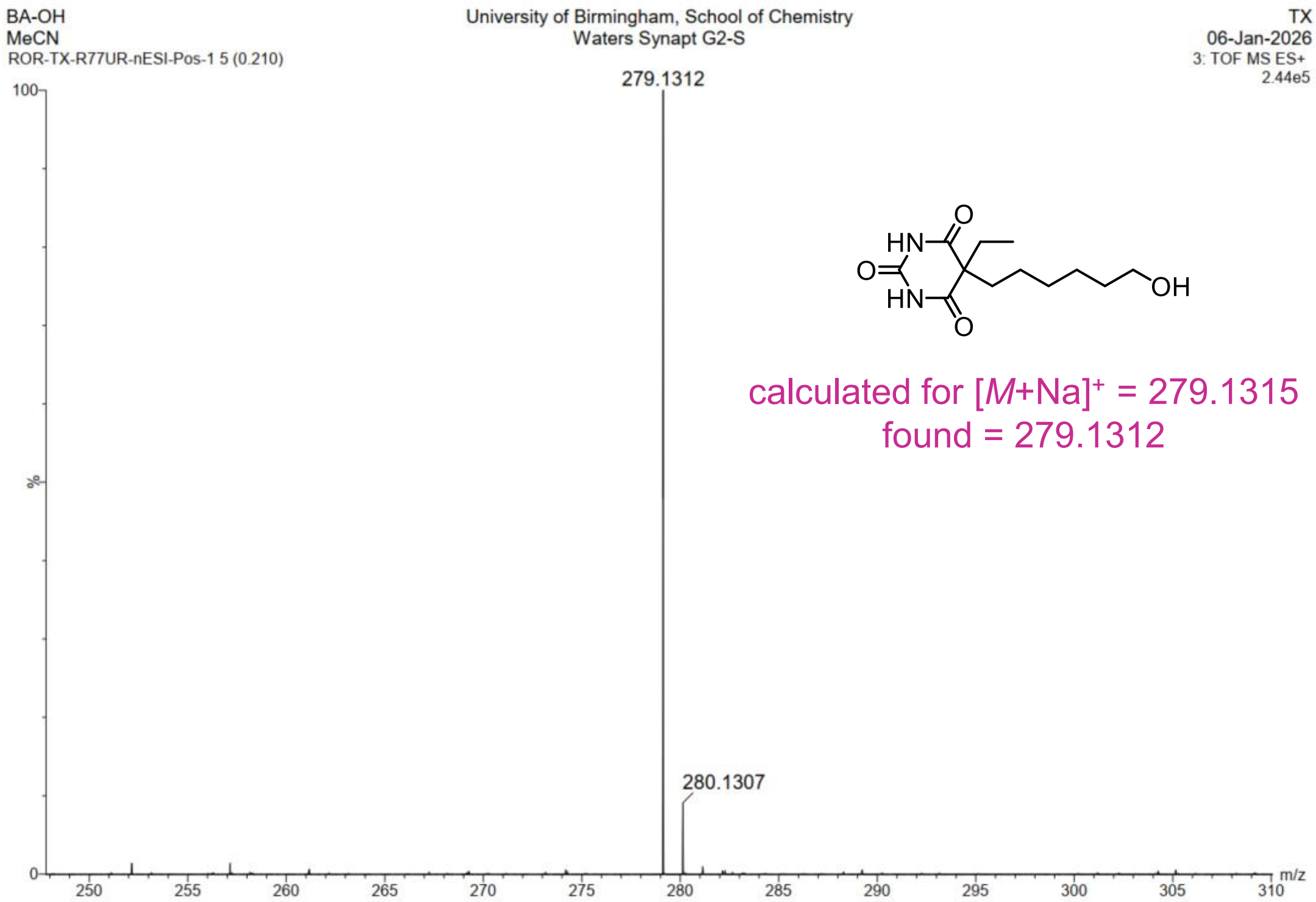

Supplementary Figure S37. Positive mode ESI-MS spectrum of BA-OH.

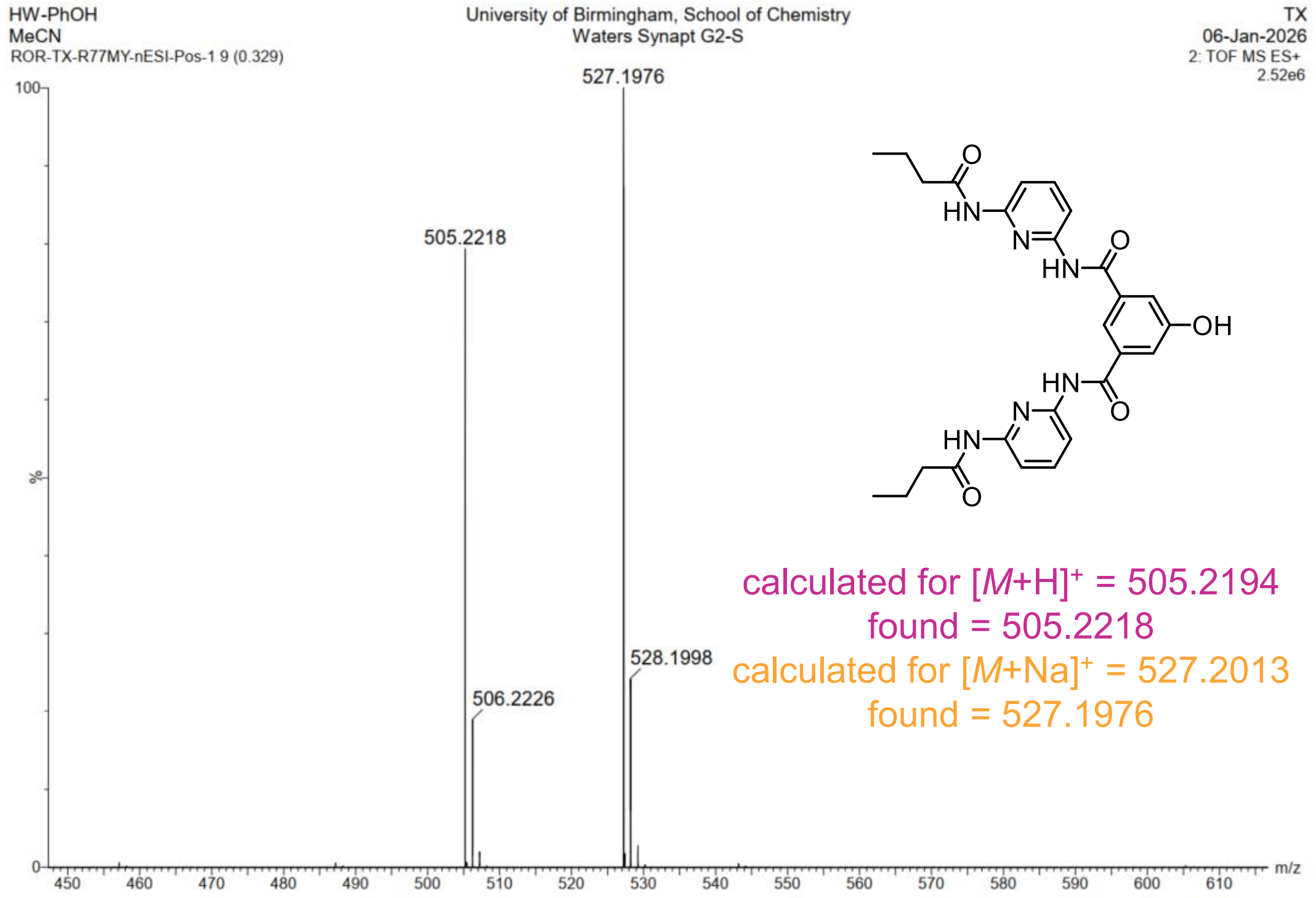

Supplementary Figure S38. Positive mode ESI-MS spectrum of HW-PhOH.

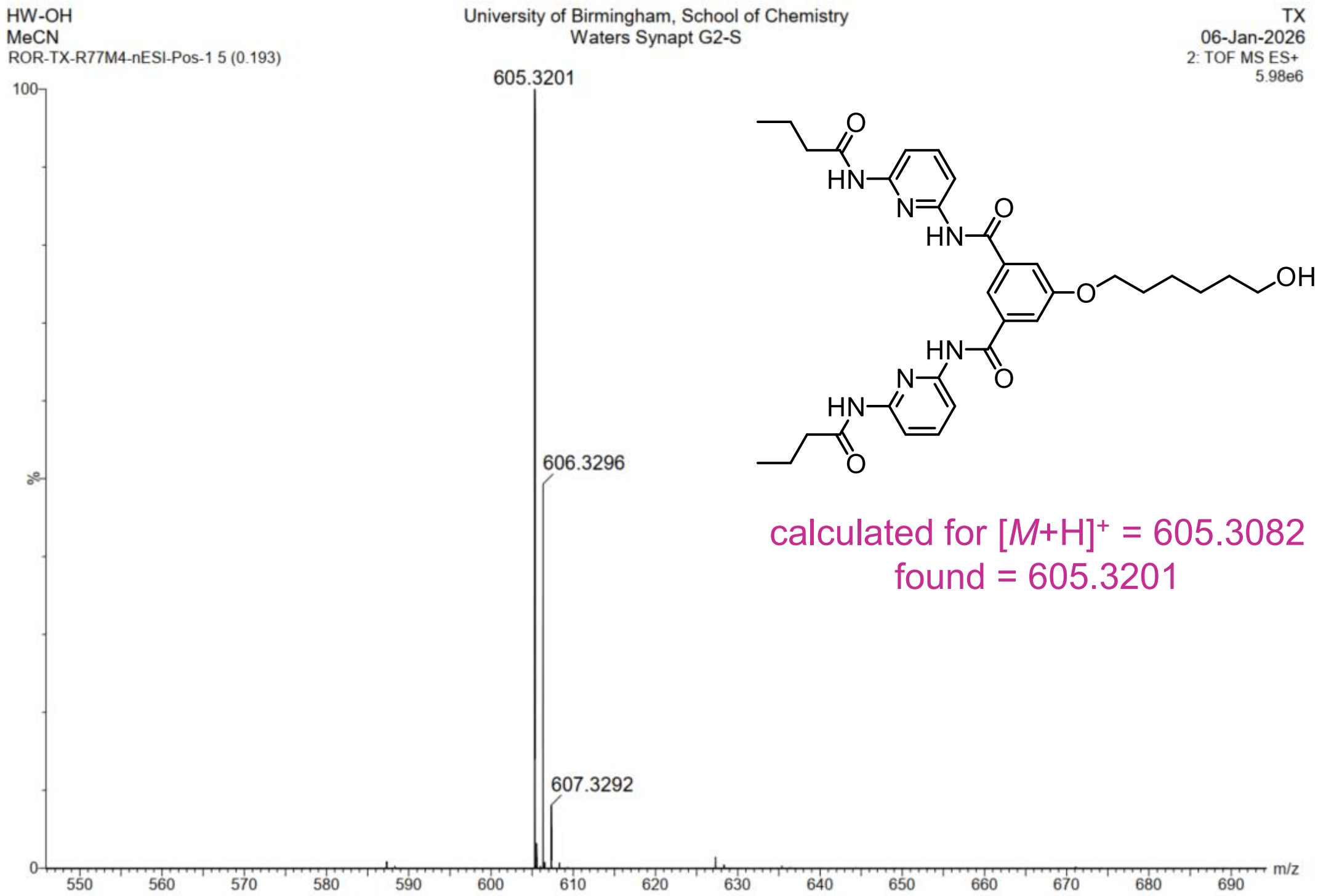

Supplementary Figure S39. Positive mode ESI-MS spectrum of HW-OH.

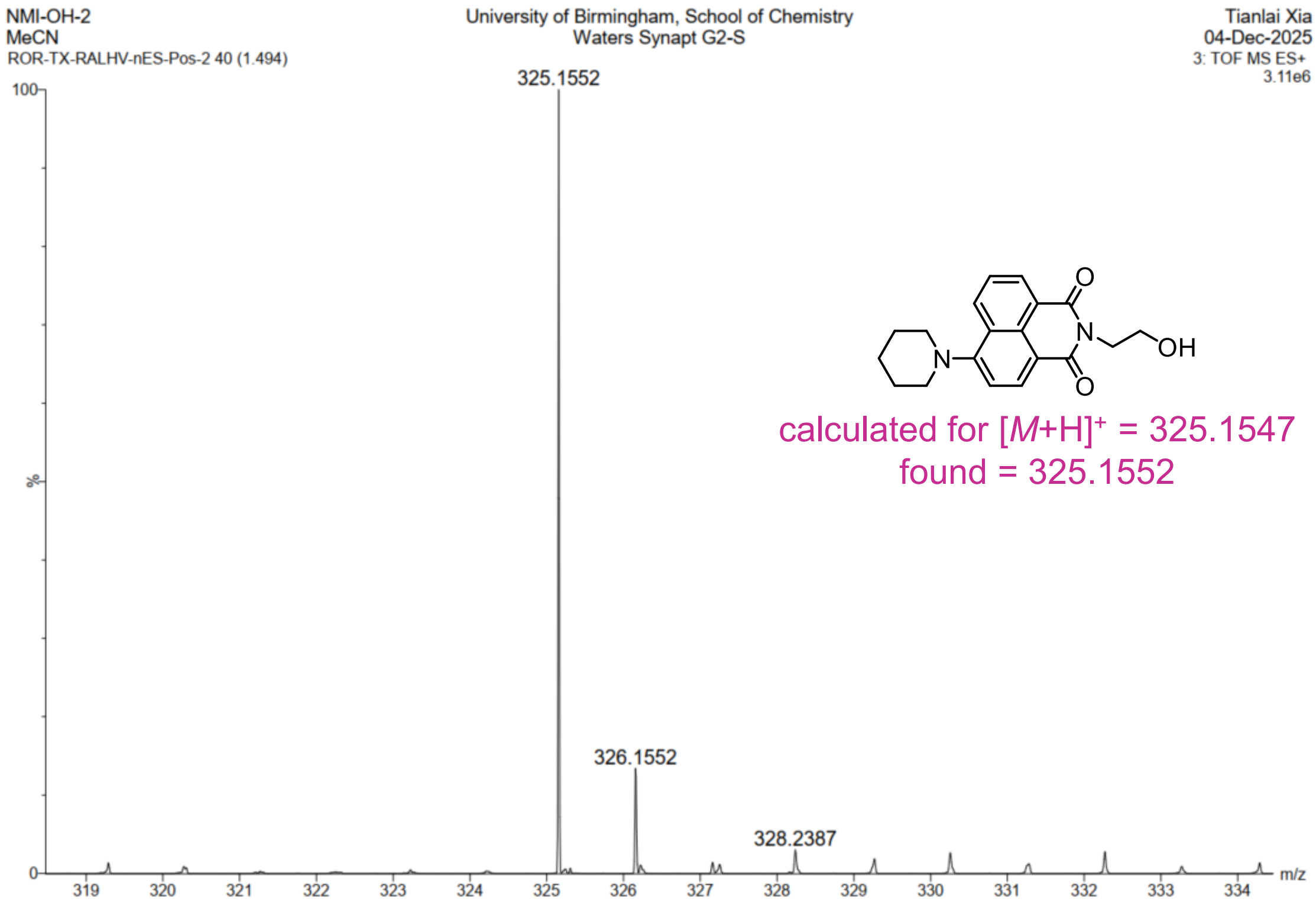

Supplementary Figure S40. Positive mode ESI-MS spectrum of NMI-OH.

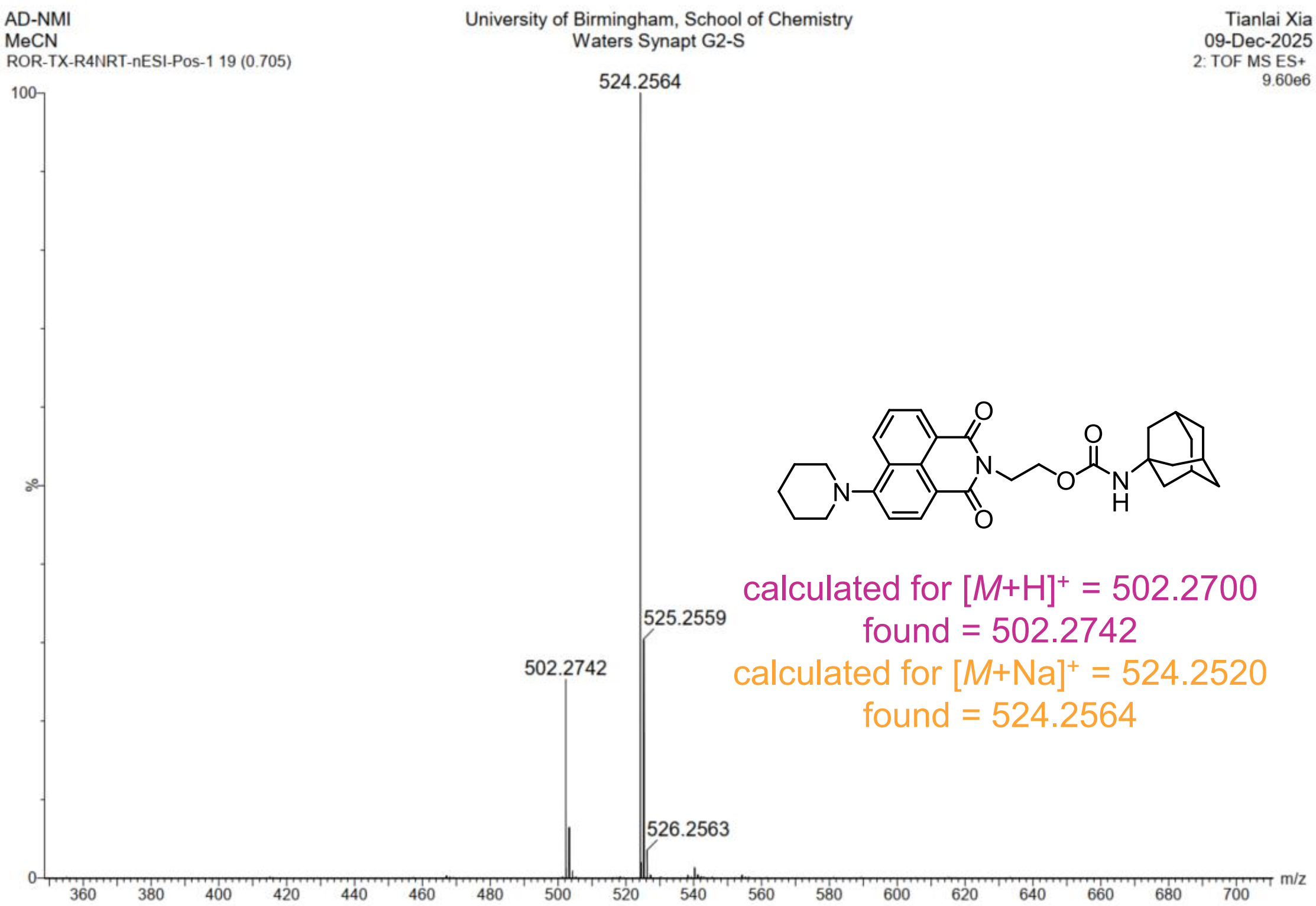

Supplementary Figure S41. Positive mode ESI-MS spectrum of NMI-Ad.

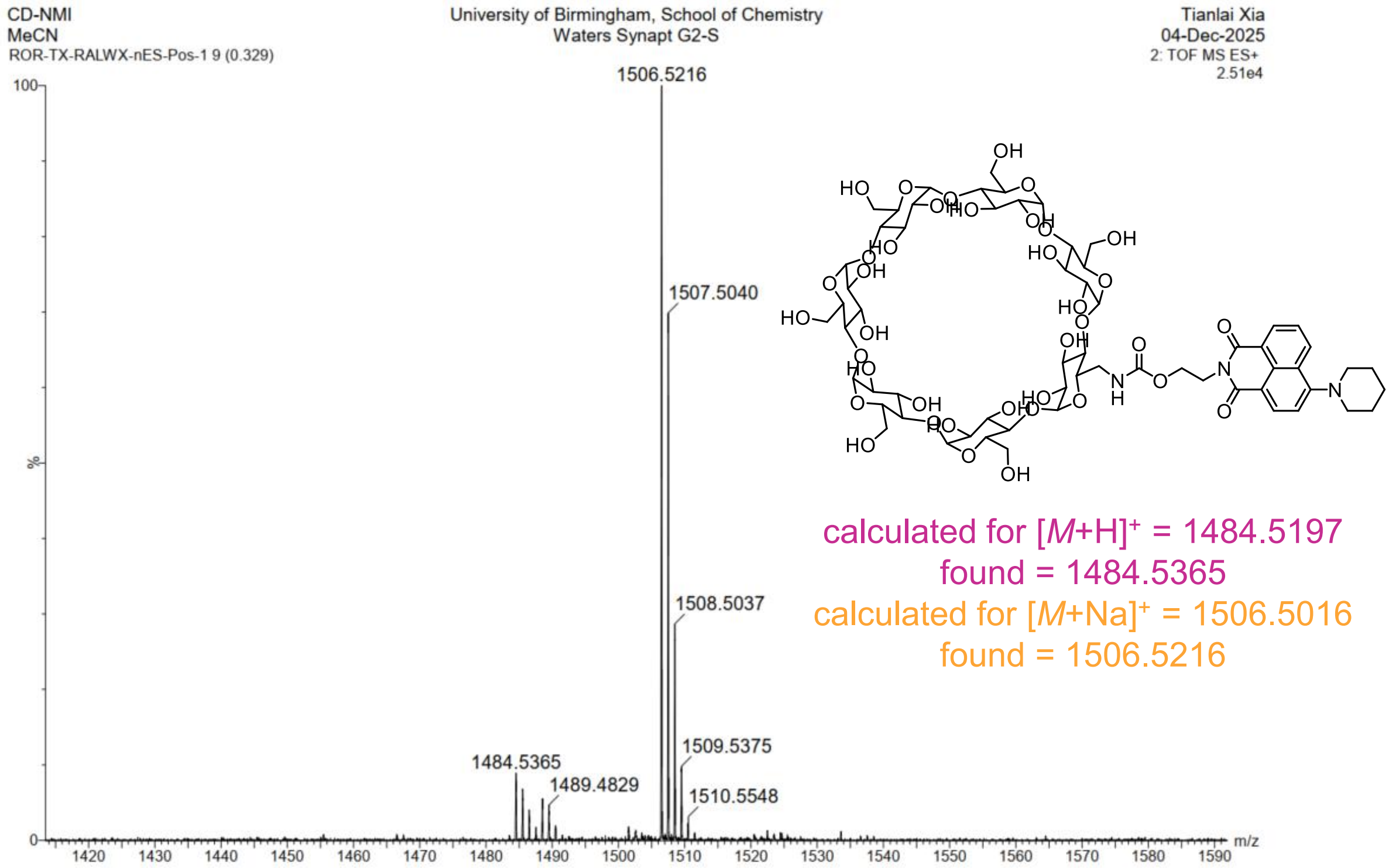

Supplementary Figure S42. Positive mode ESI-MS spectrum of NMI-CD.

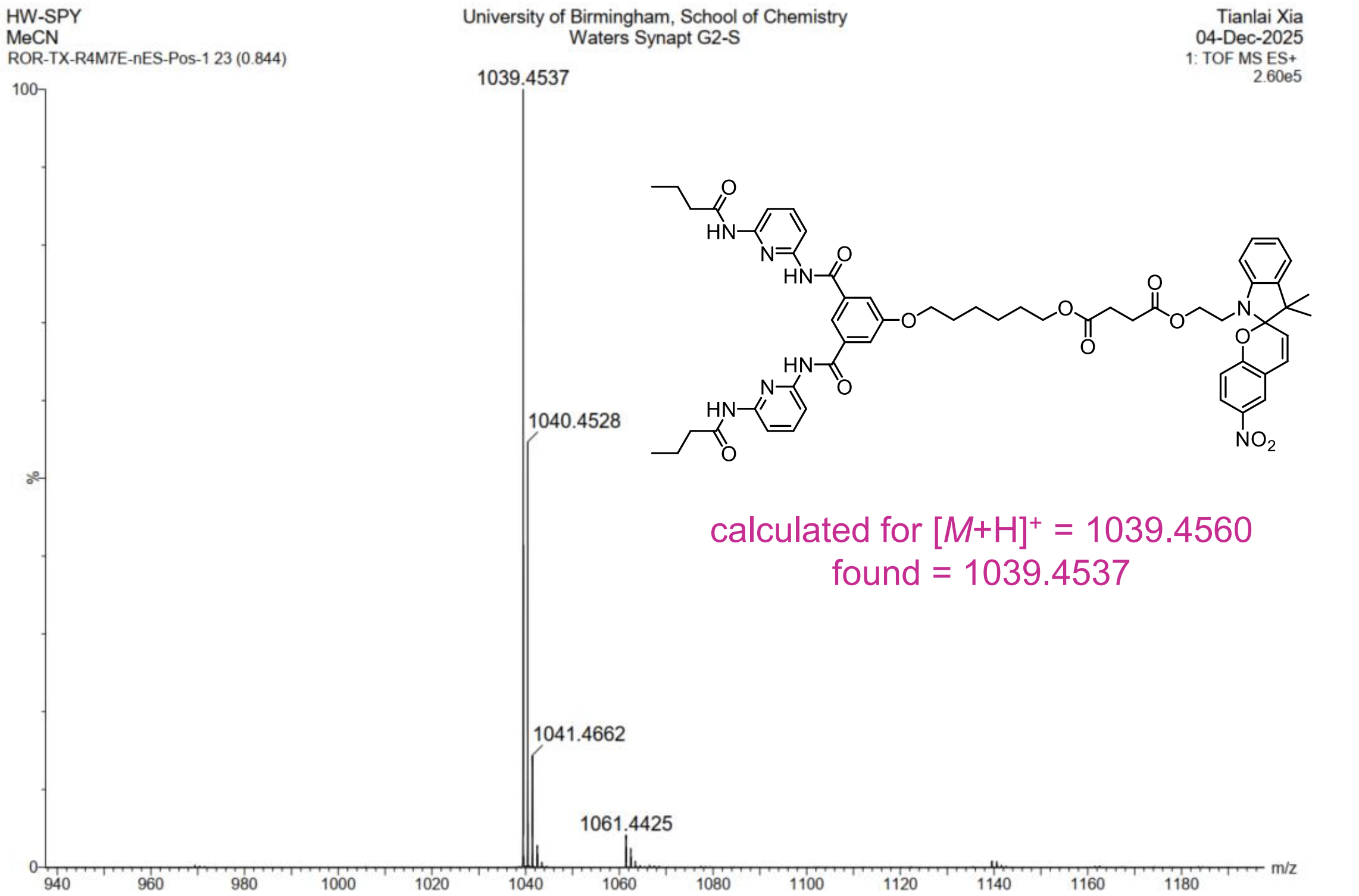

Supplementary Figure S43. Positive mode ESI-MS spectrum of SPY-HW.

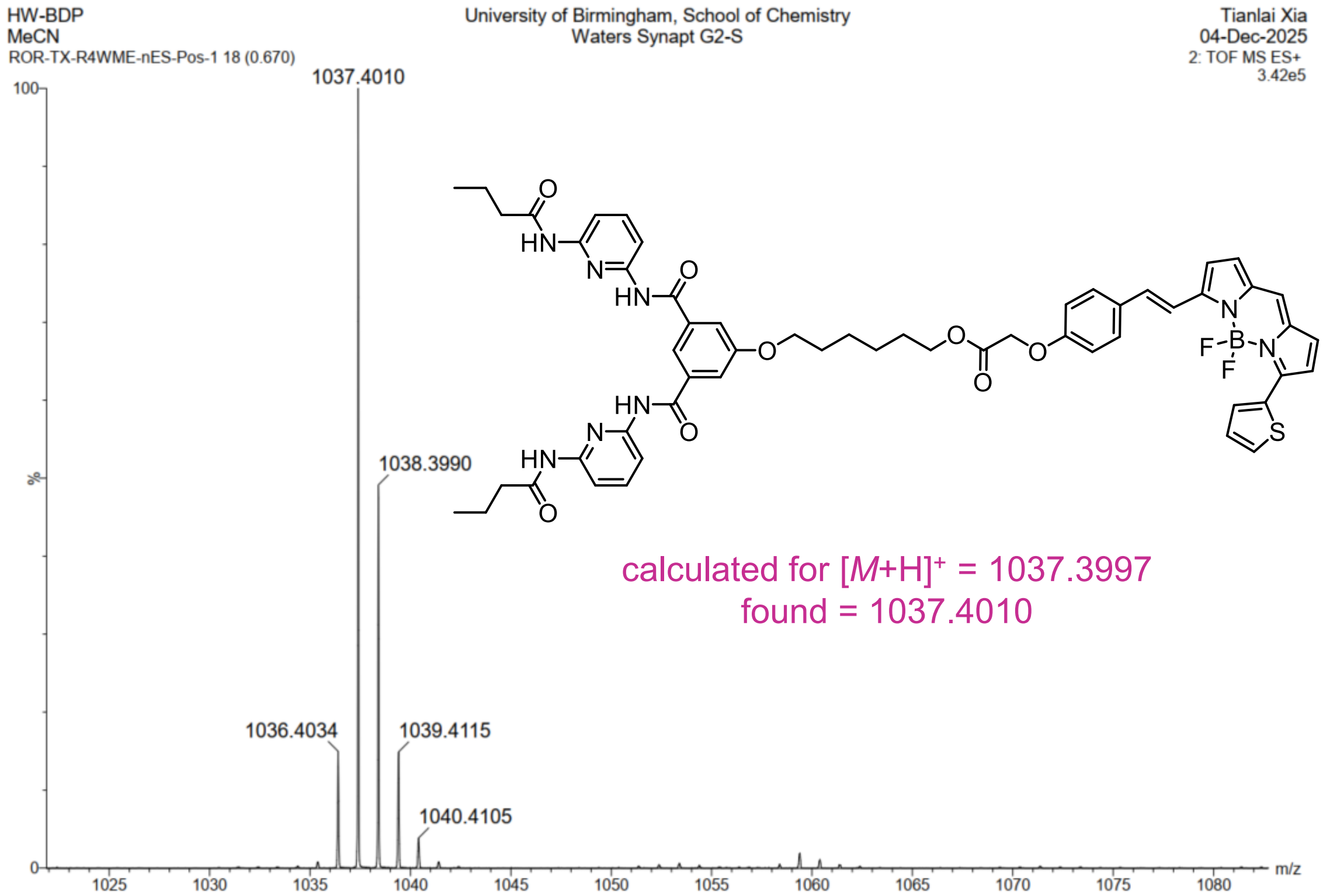

Supplementary Figure S44. Positive mode ESI-MS spectrum of BDP-HW.

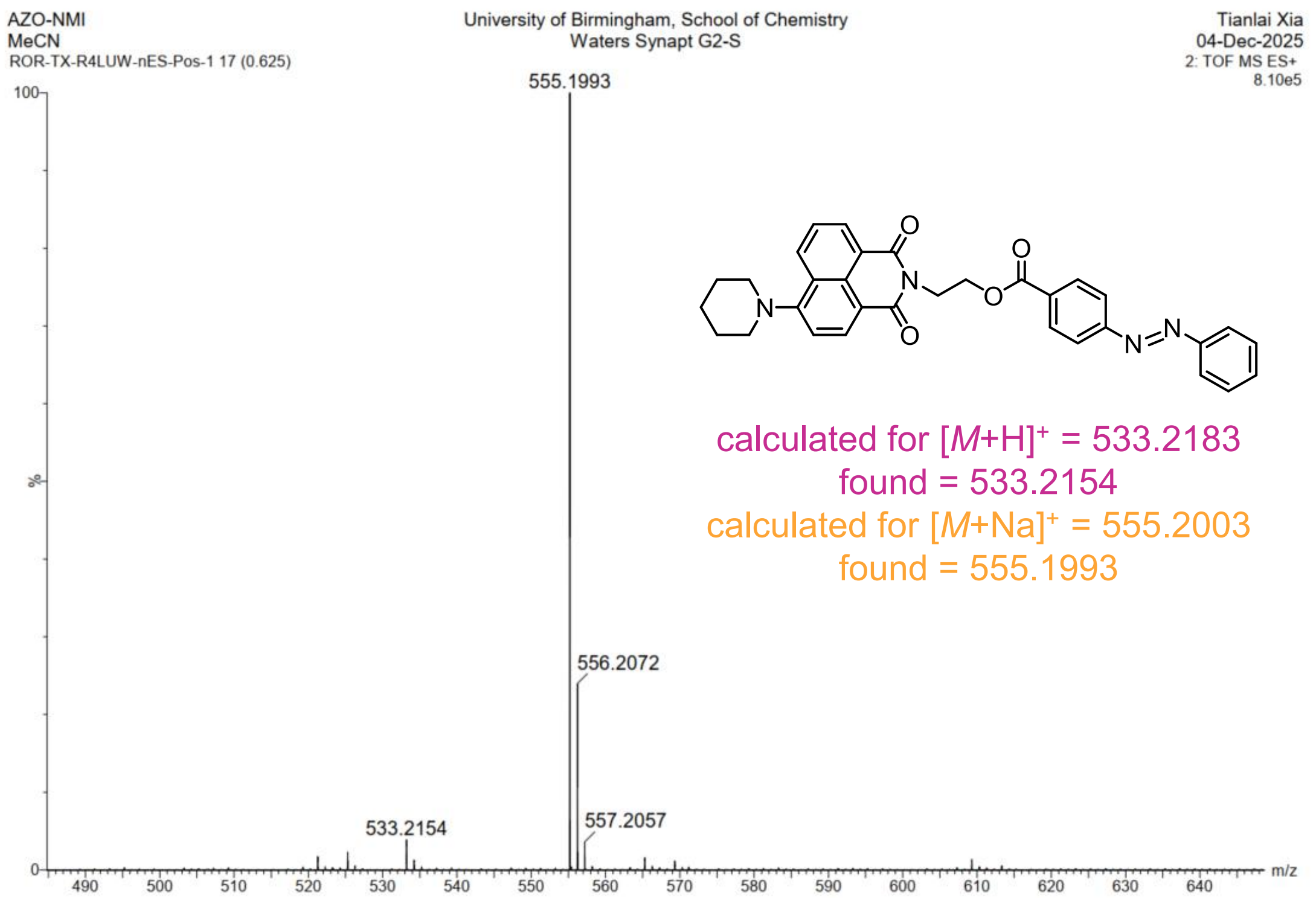

Supplementary Figure S45. Positive mode ESI-MS spectrum of NMI-Azo.

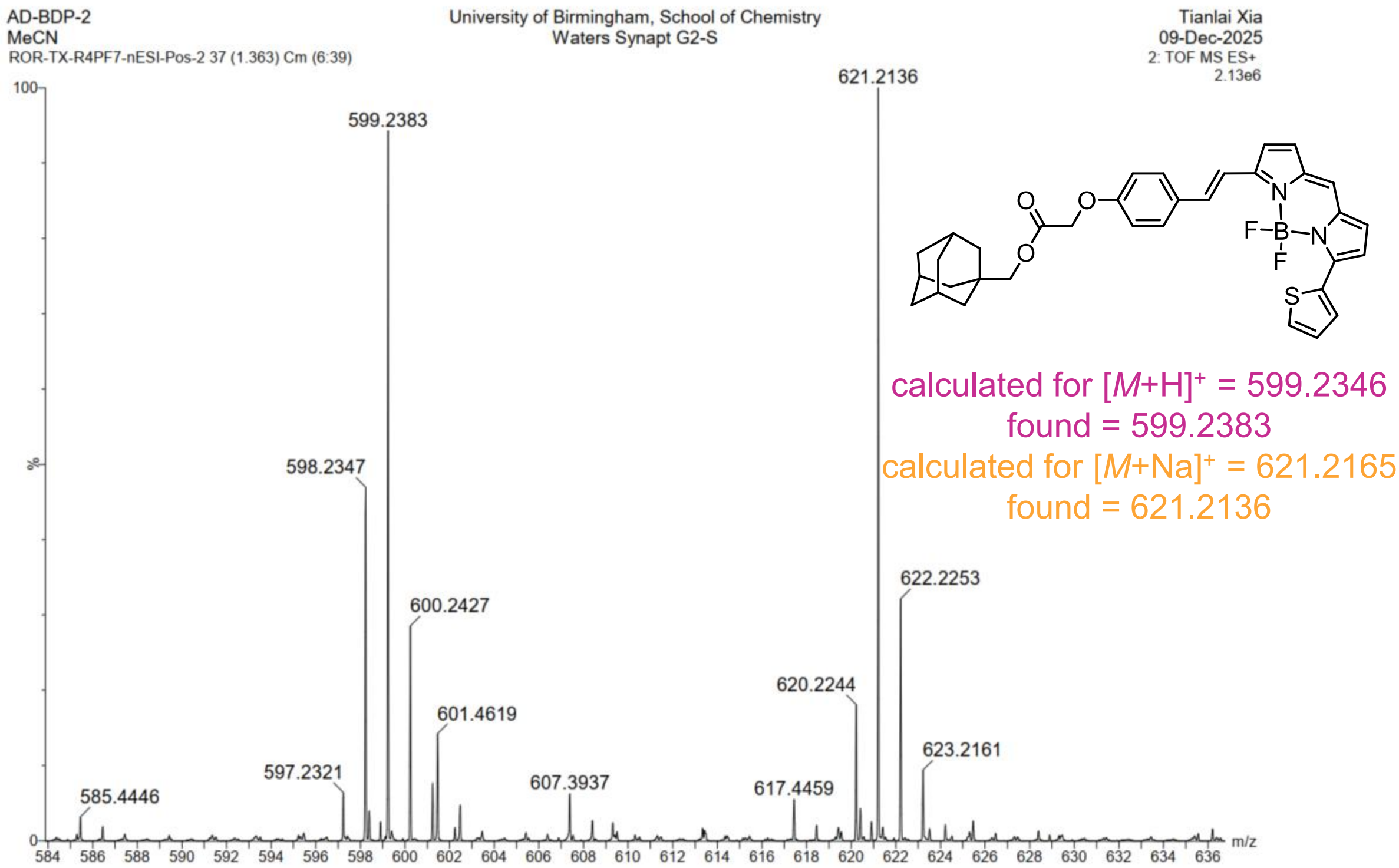

Supplementary Figure S46. Positive mode ESI-MS spectrum of BDP-Ad.

FT-IR spectra

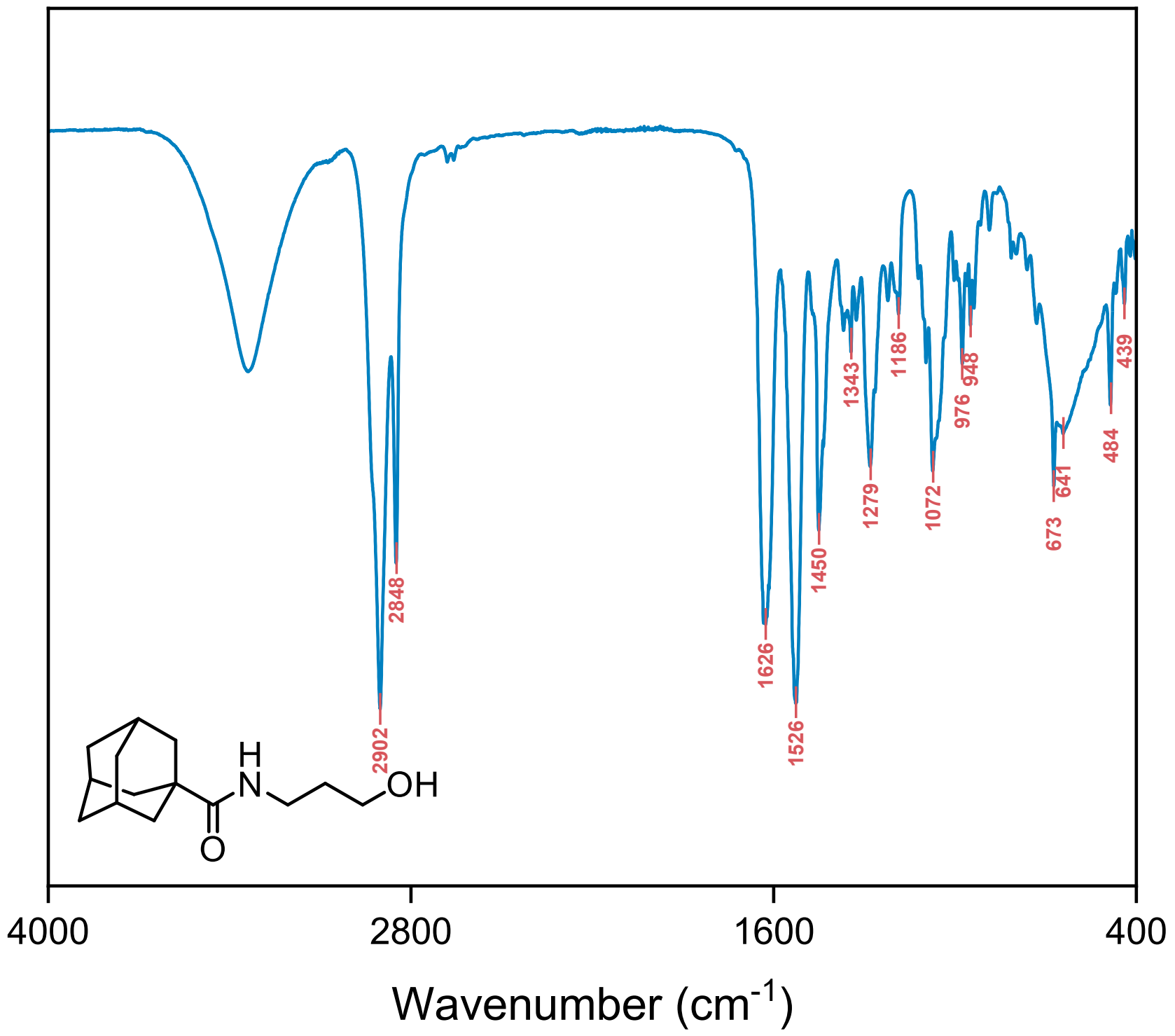

Supplementary Figure S47. FT-IR spectrum of Ad-C3-OH.

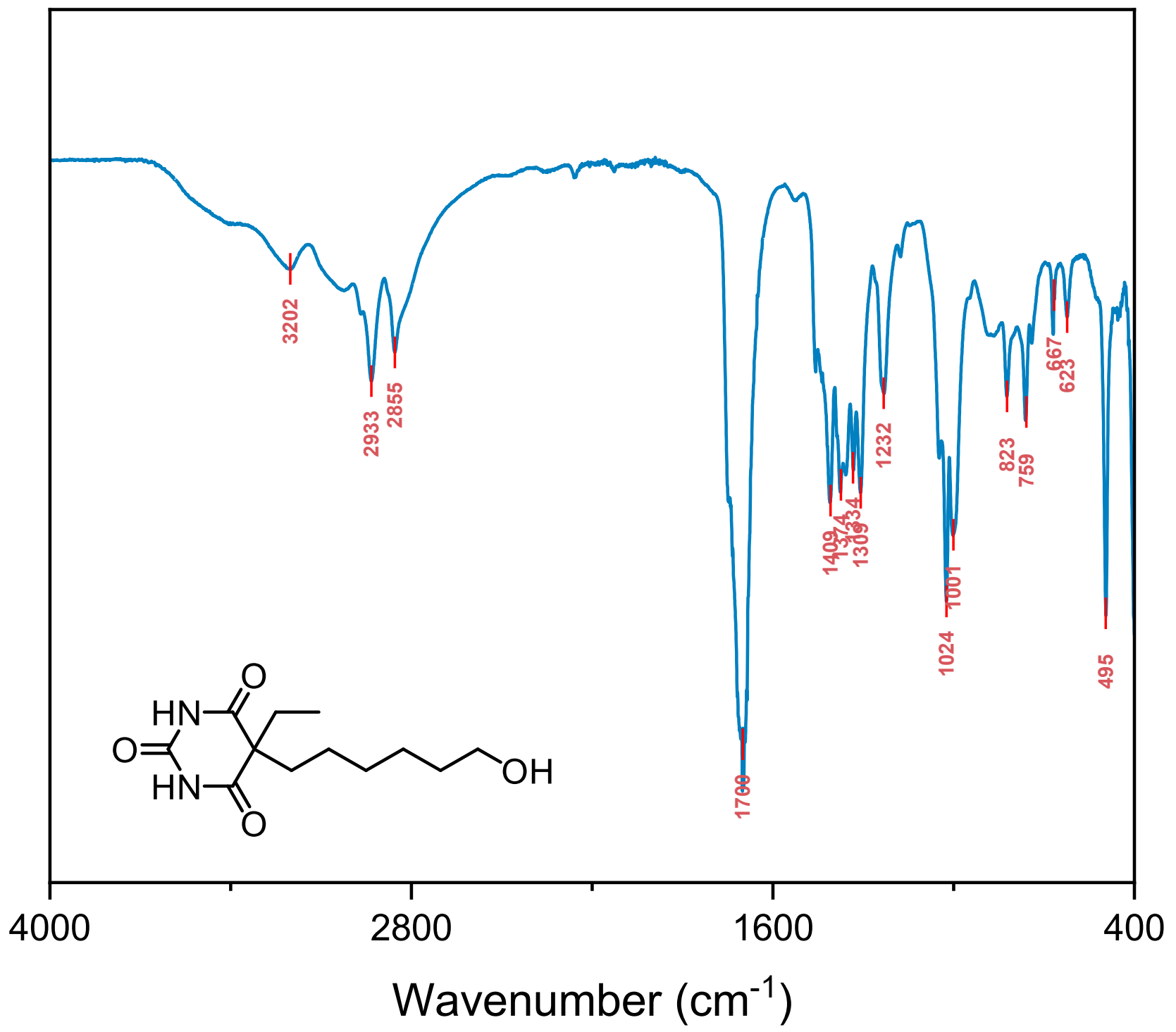

Supplementary Figure S48. FT-IR spectrum of BA-OH.

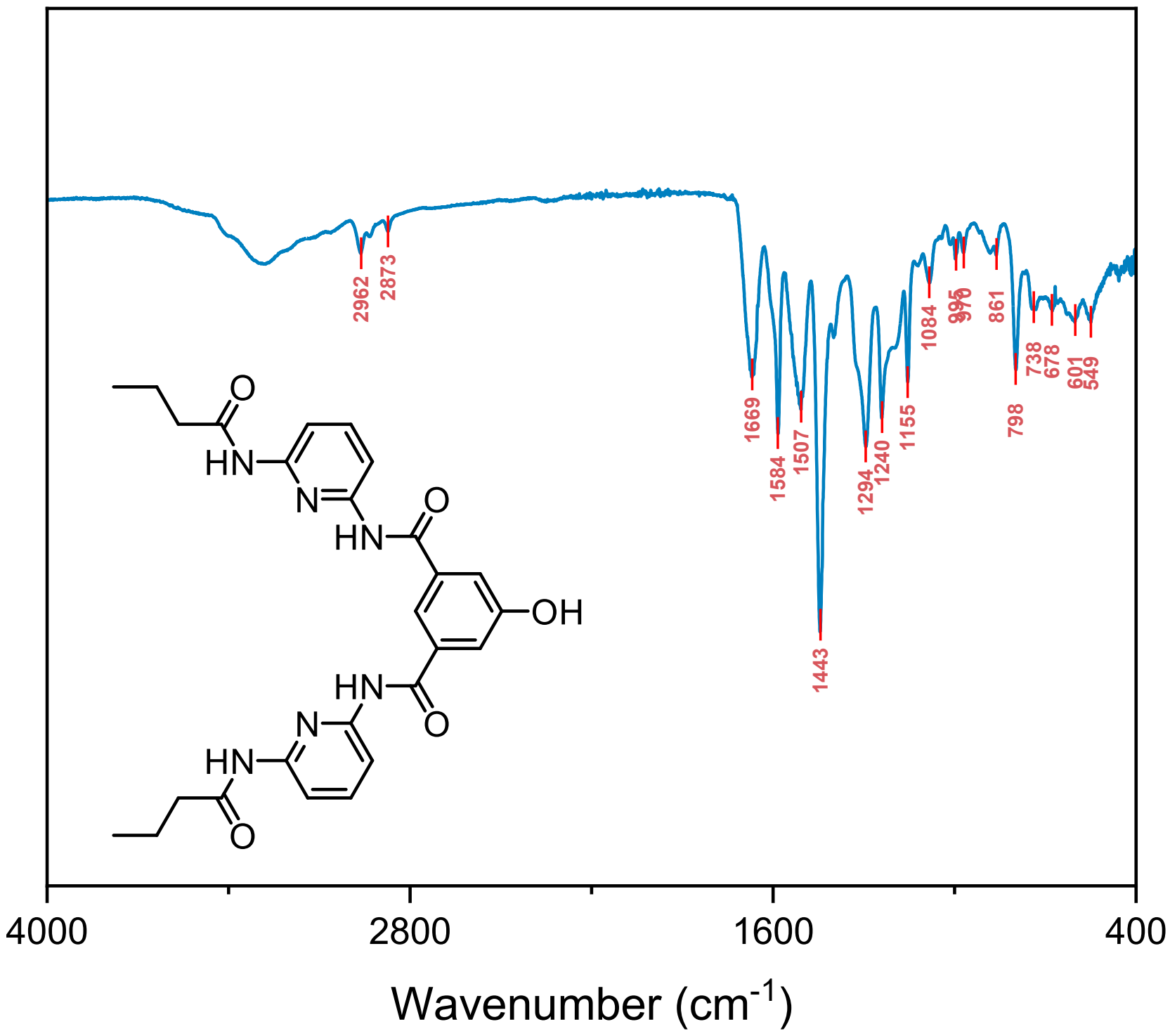

Supplementary Figure S49. FT-IR spectrum of HW-PhOH.

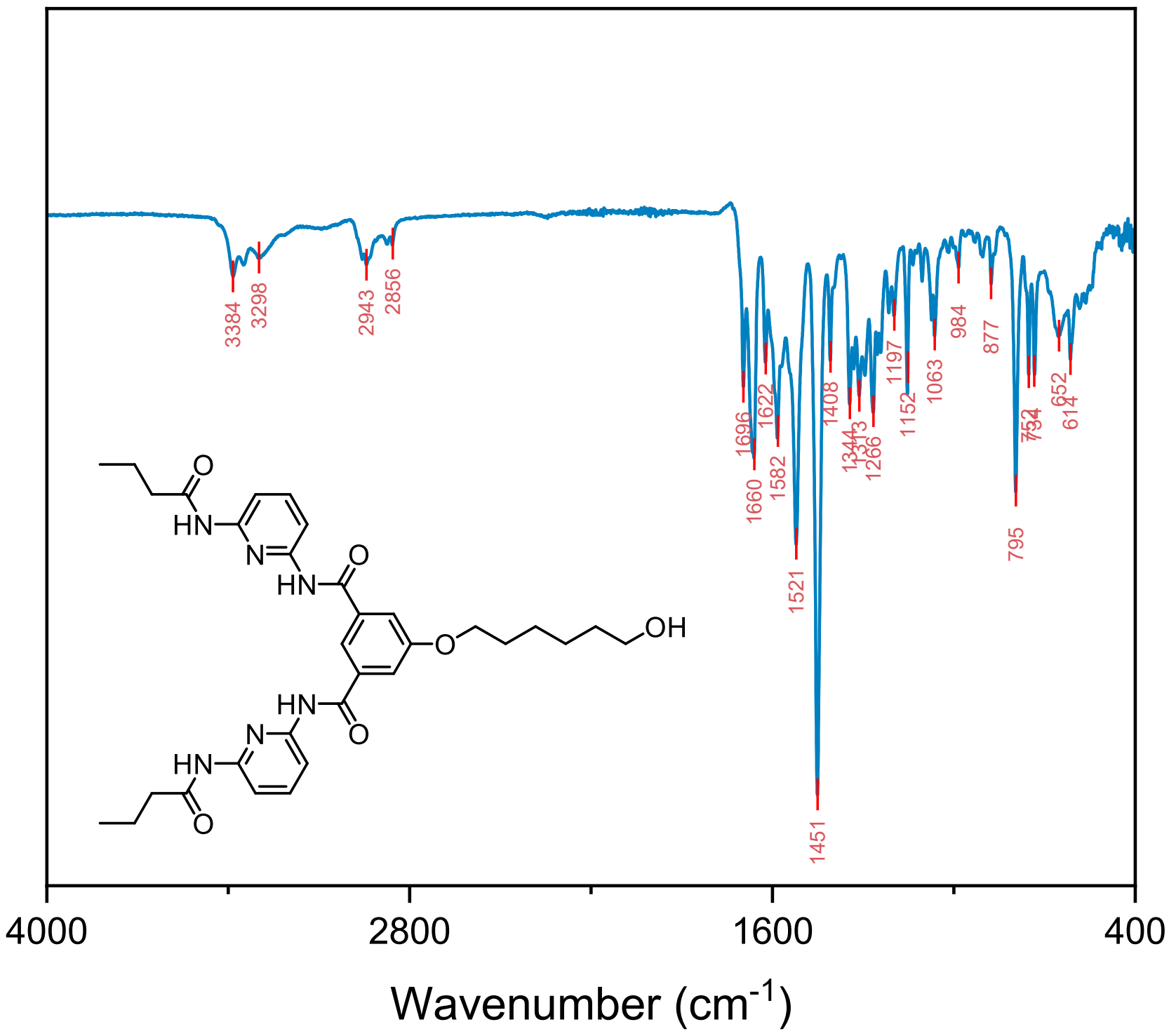

Supplementary Figure S50. FT-IR spectrum of HW-OH.

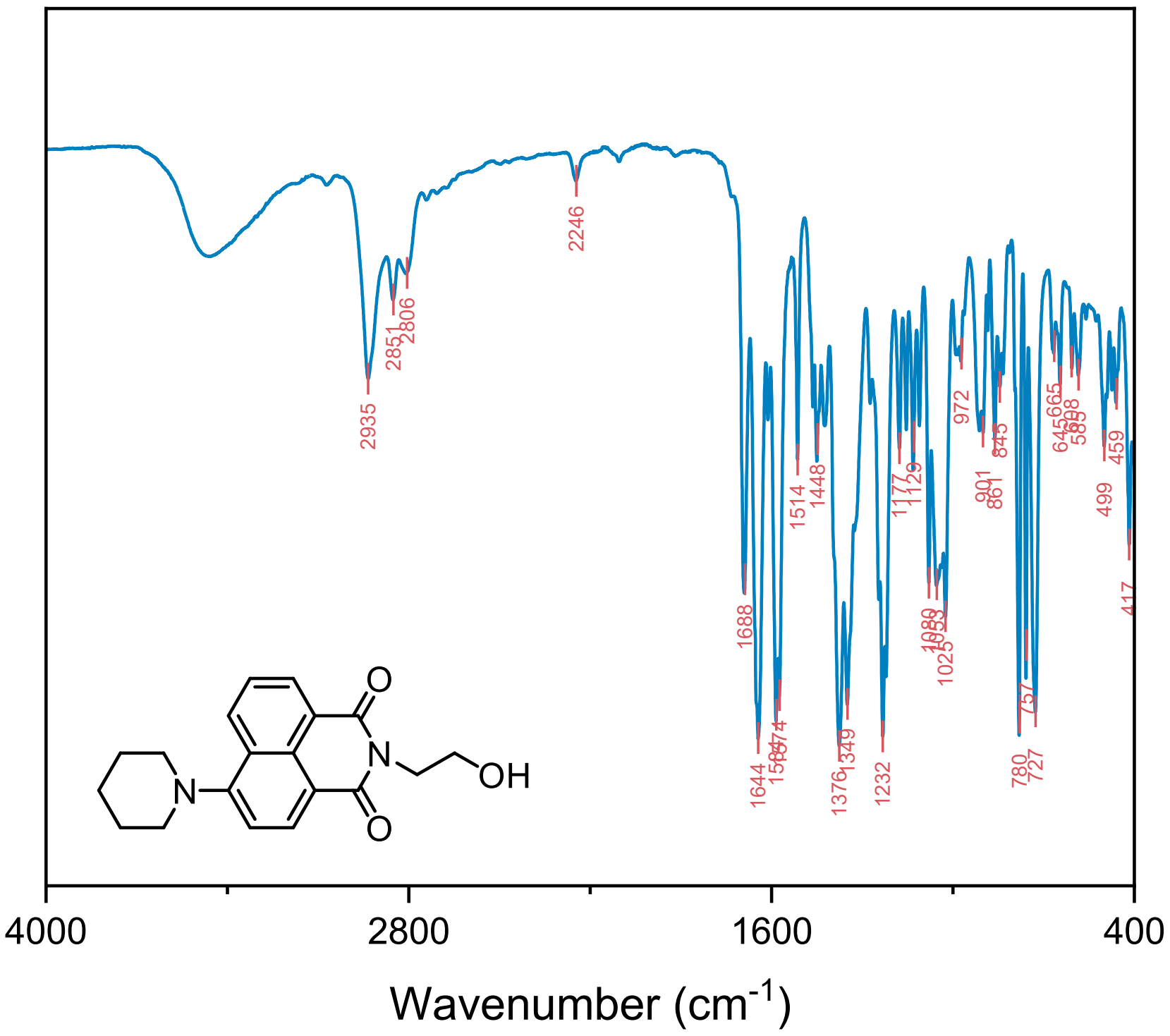

Supplementary Figure S51. FT-IR spectrum of NMI-OH.

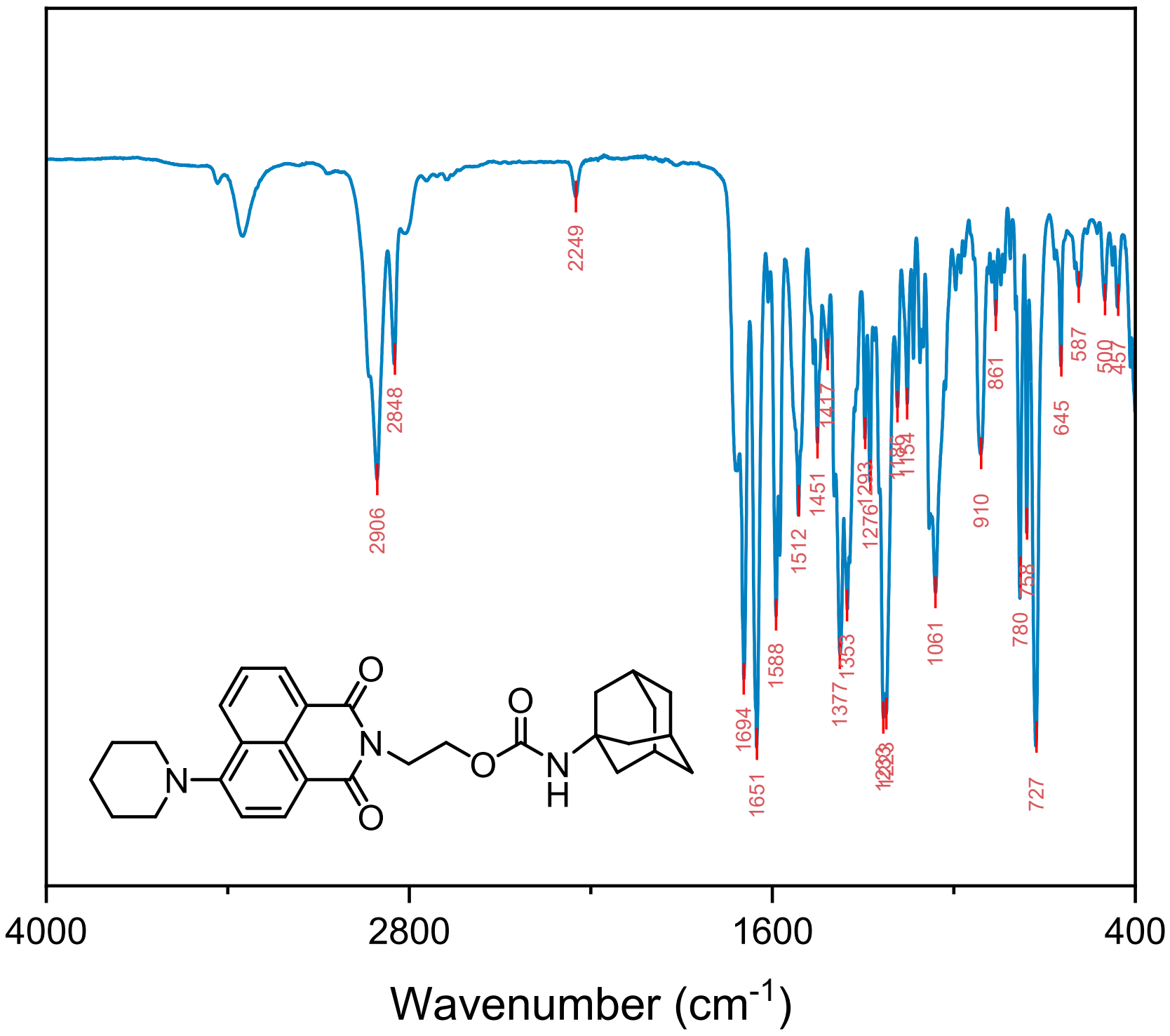

Supplementary Figure S52. FT-IR spectrum of NMI-Ad.

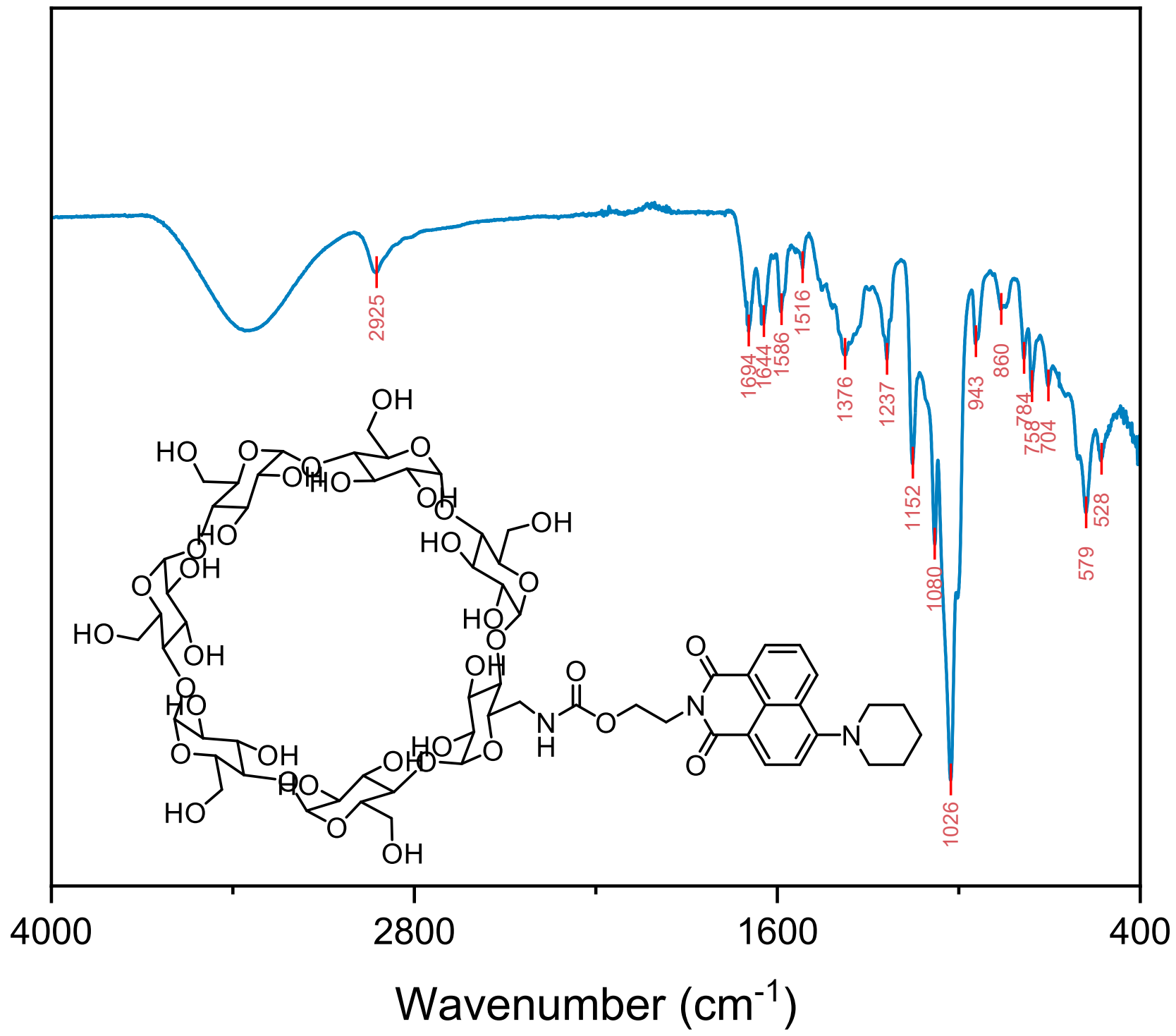

Supplementary Figure S53. FT-IR spectrum of NMI-CD.

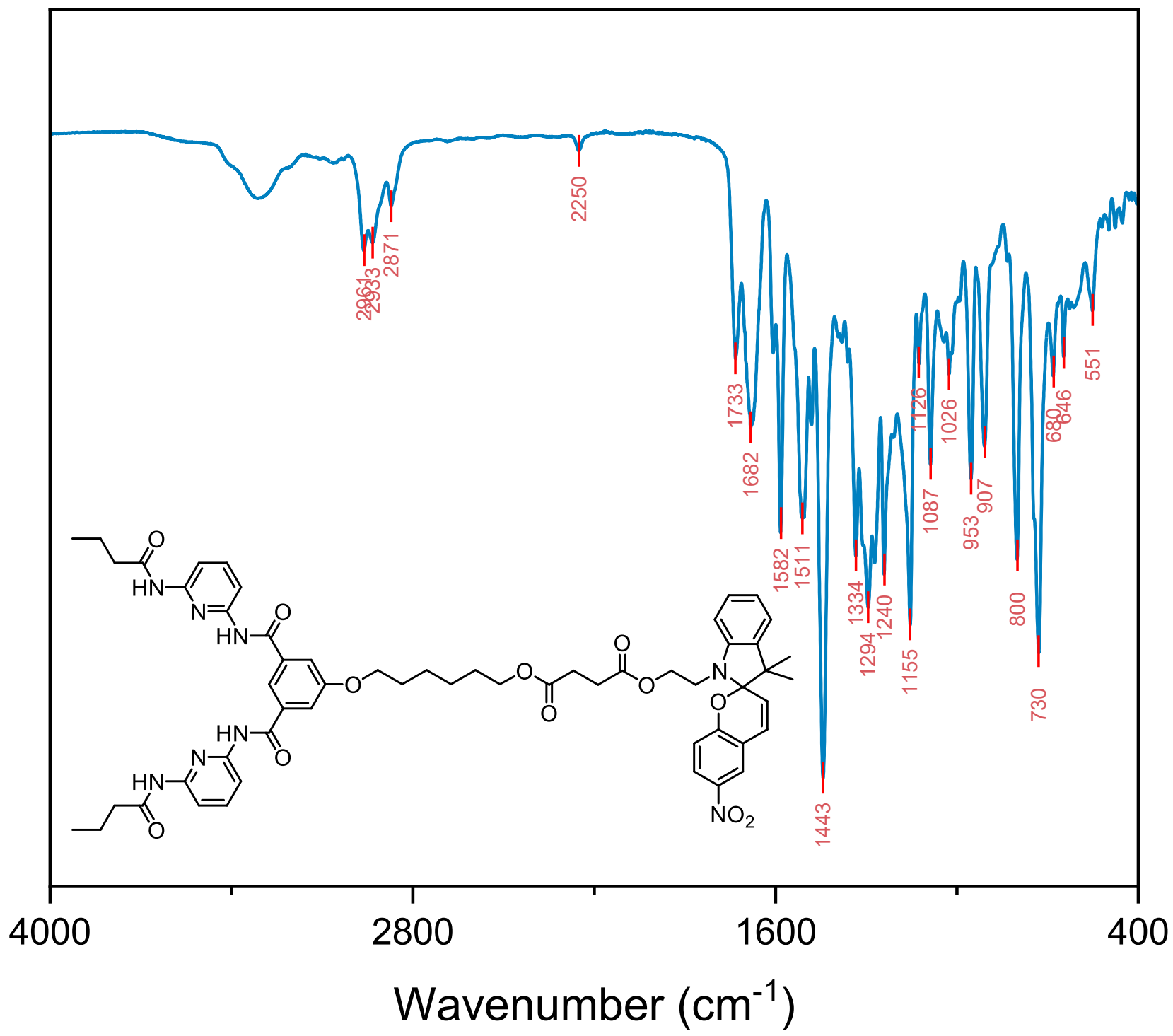

Supplementary Figure S54. FT-IR spectrum of SPY-HW.

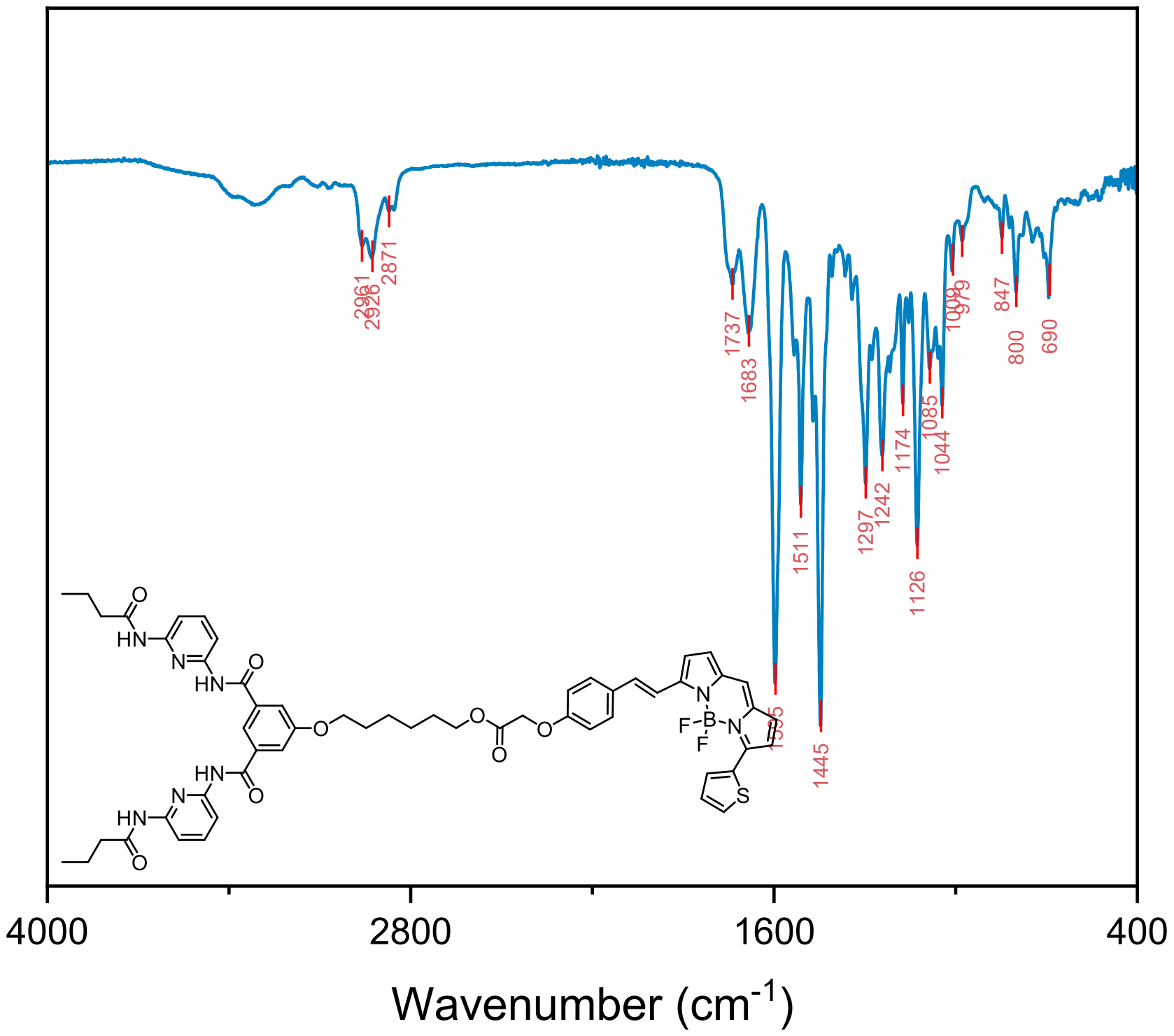

Supplementary Figure S55. FT-IR spectrum of BDP-HW.

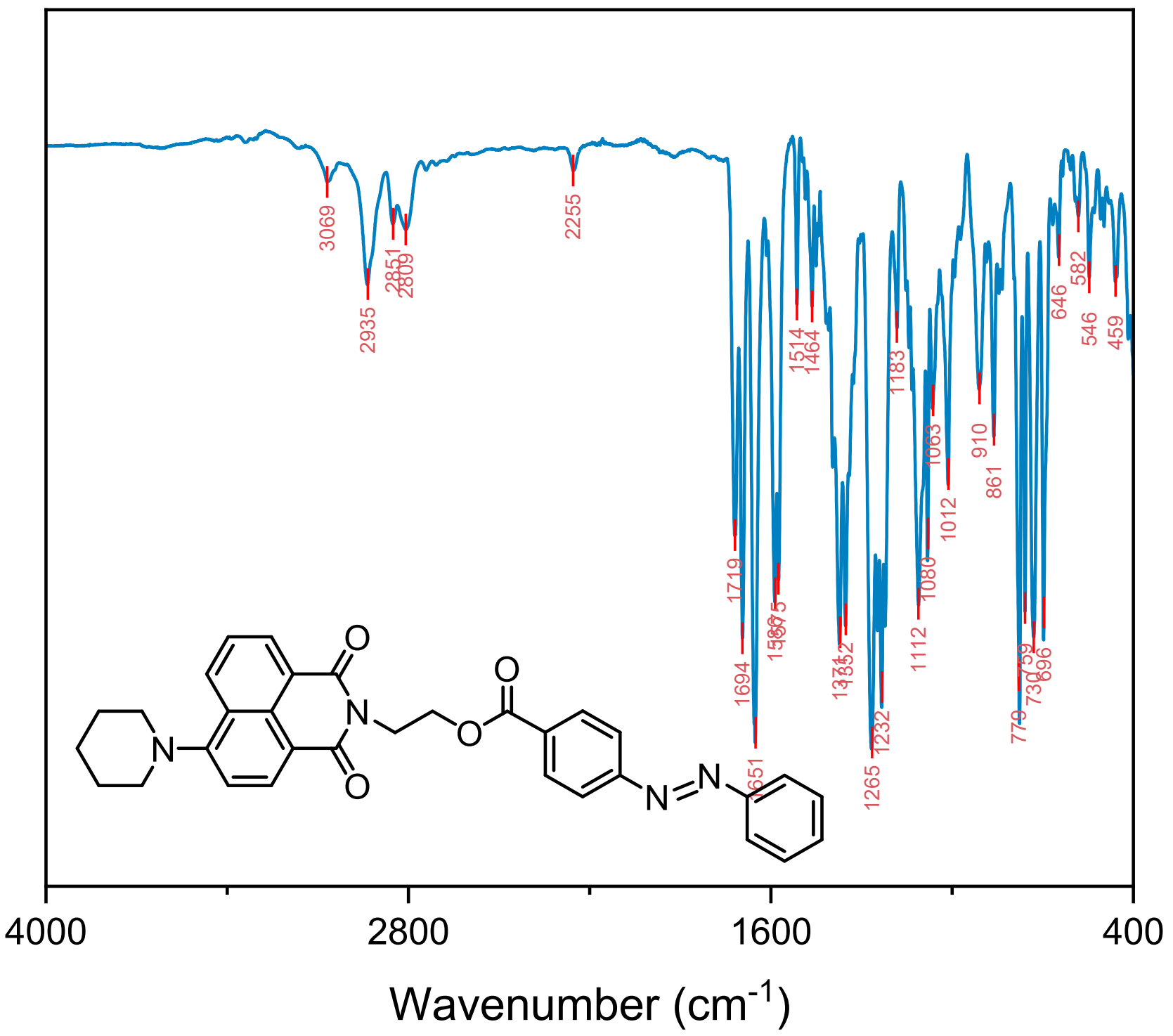

Supplementary Figure S56. FT-IR spectrum of NMI-Azo.

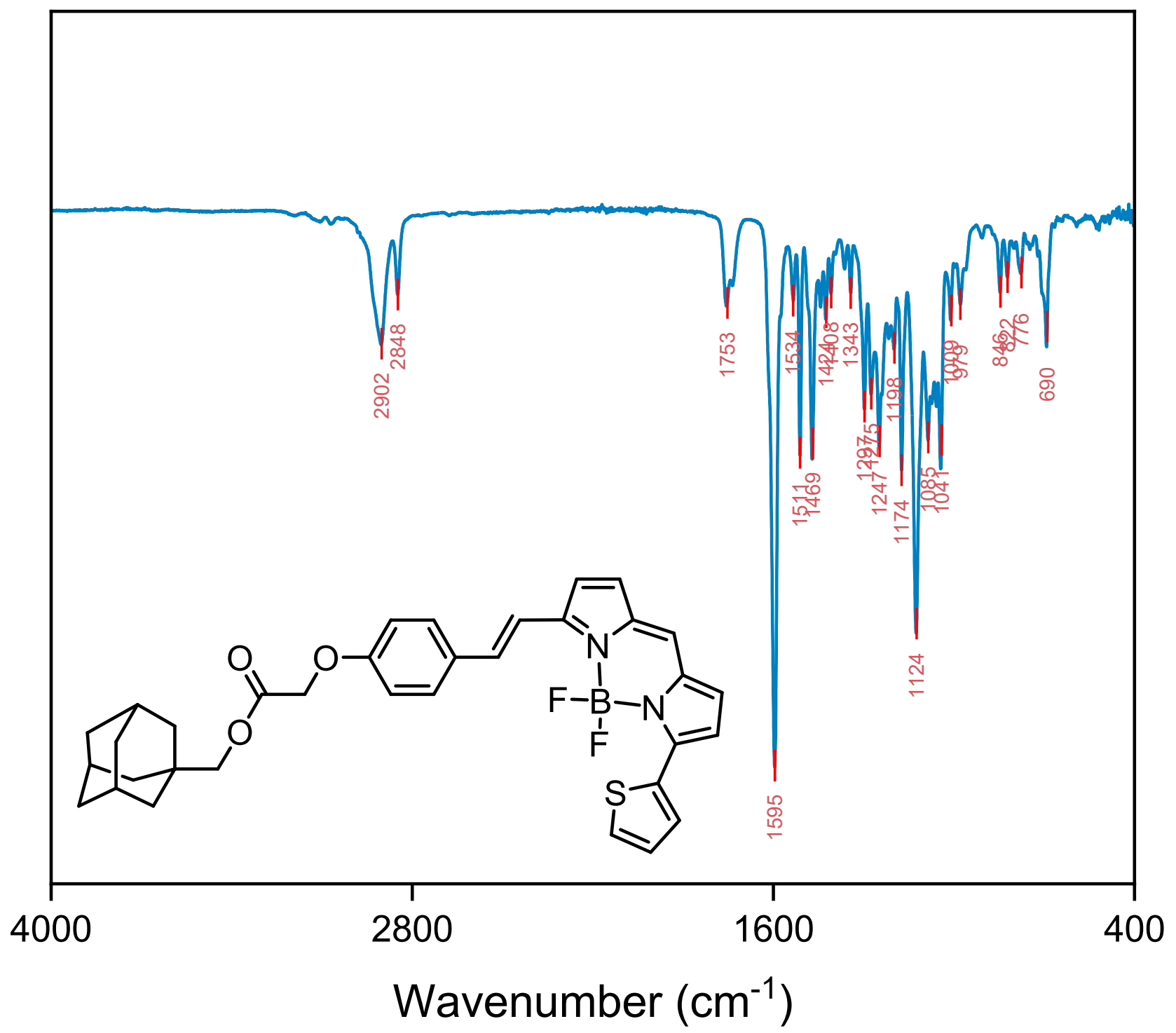

Supplementary Figure S57. FT-IR spectrum of BDP-Ad.

Size exclusion chromatogram

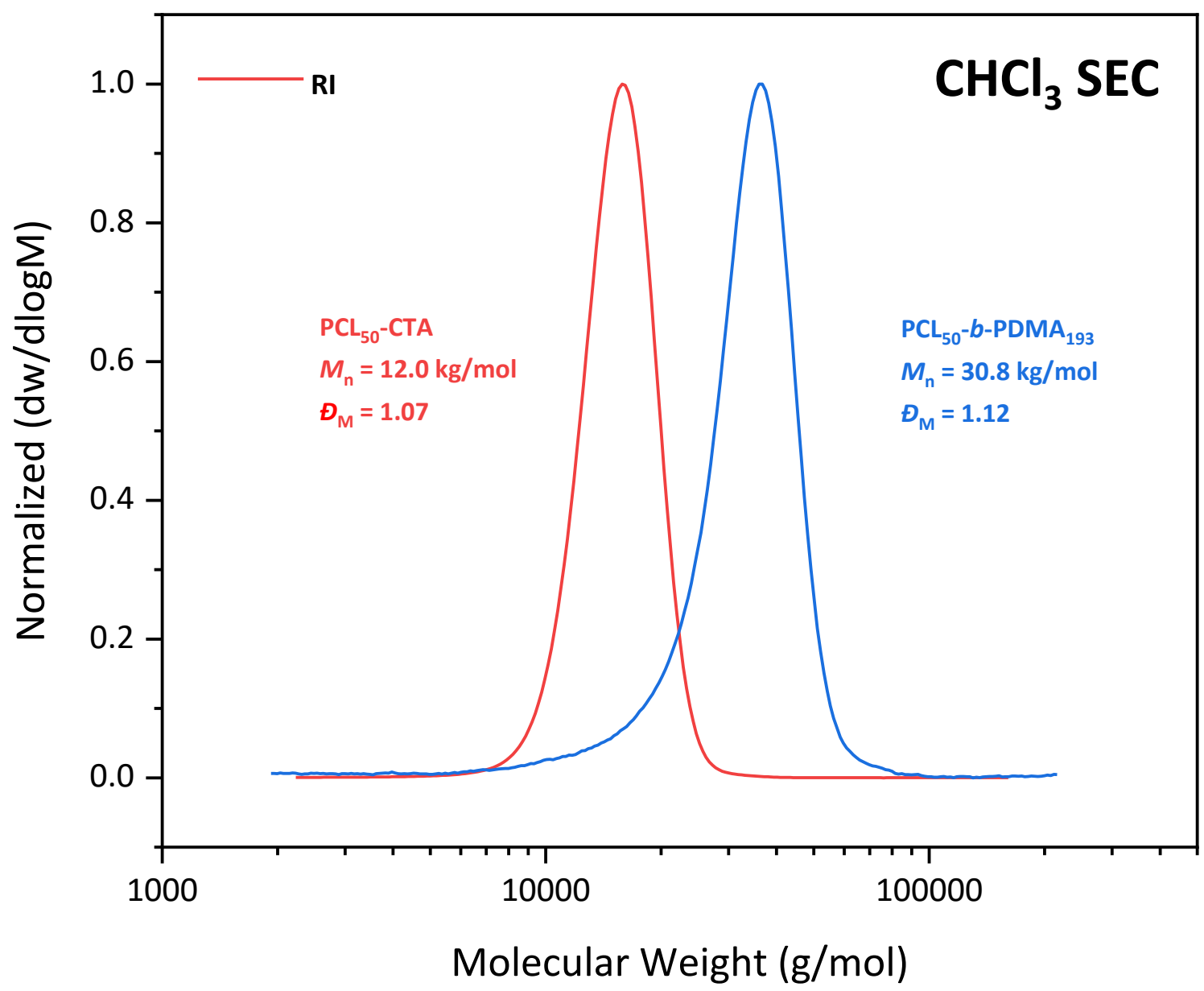

Supplementary Figure S58. Size exclusion chromatogram (RI trace) of PCL_{50}-CTA and PCL_{50}-*b*-$PDMA_{193}$ using $CHCl_3$ with 0.5% NEt_3 as an eluent (PMMA standard).

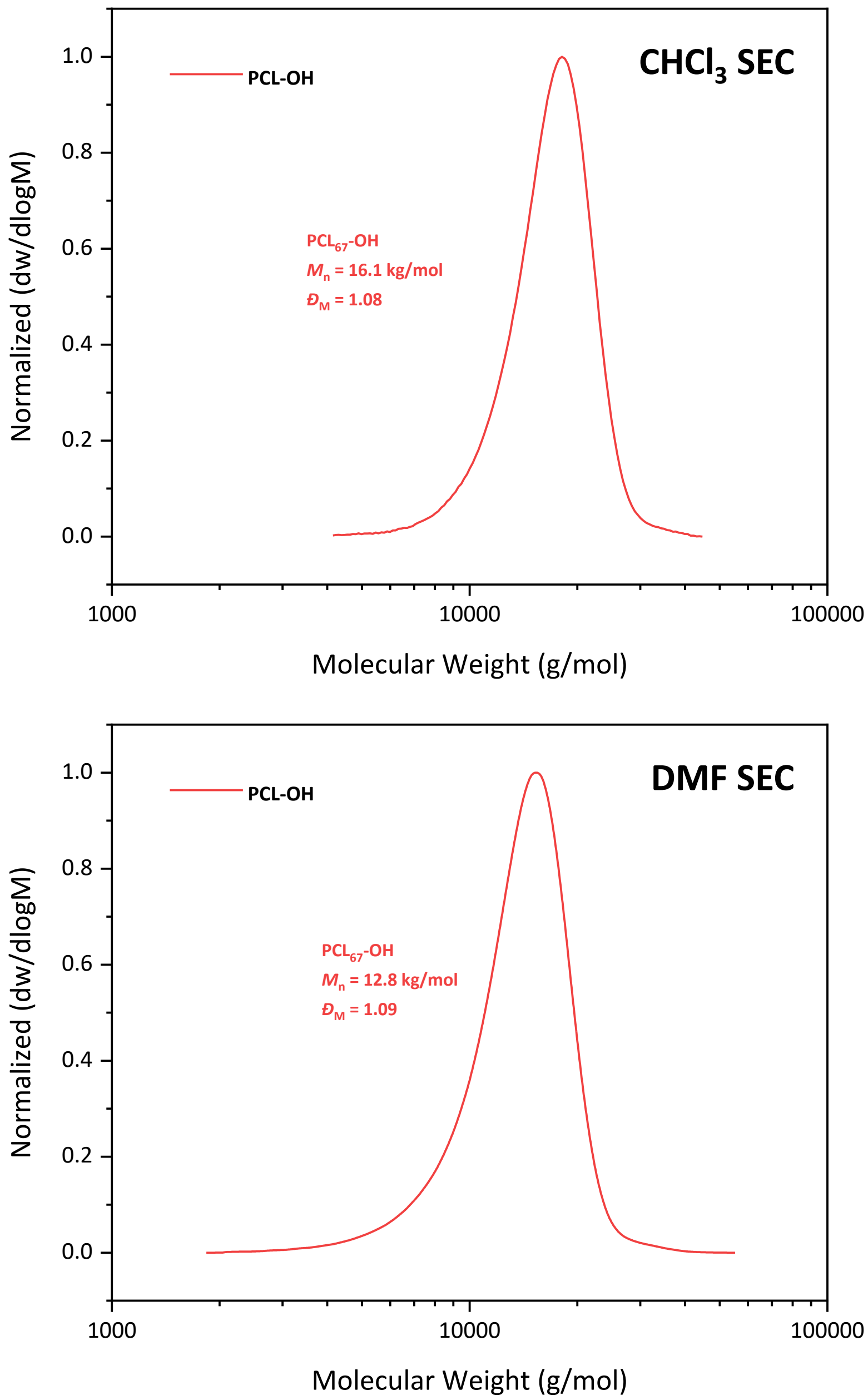

Supplementary Figure S59. Size exclusion chromatogram (RI trace) of PCL_{67}-OH using $CHCl_3$ with 0.5% NEt_3 or DMF with 5 mM NH_4BF_4 as an eluent (PMMA standard).

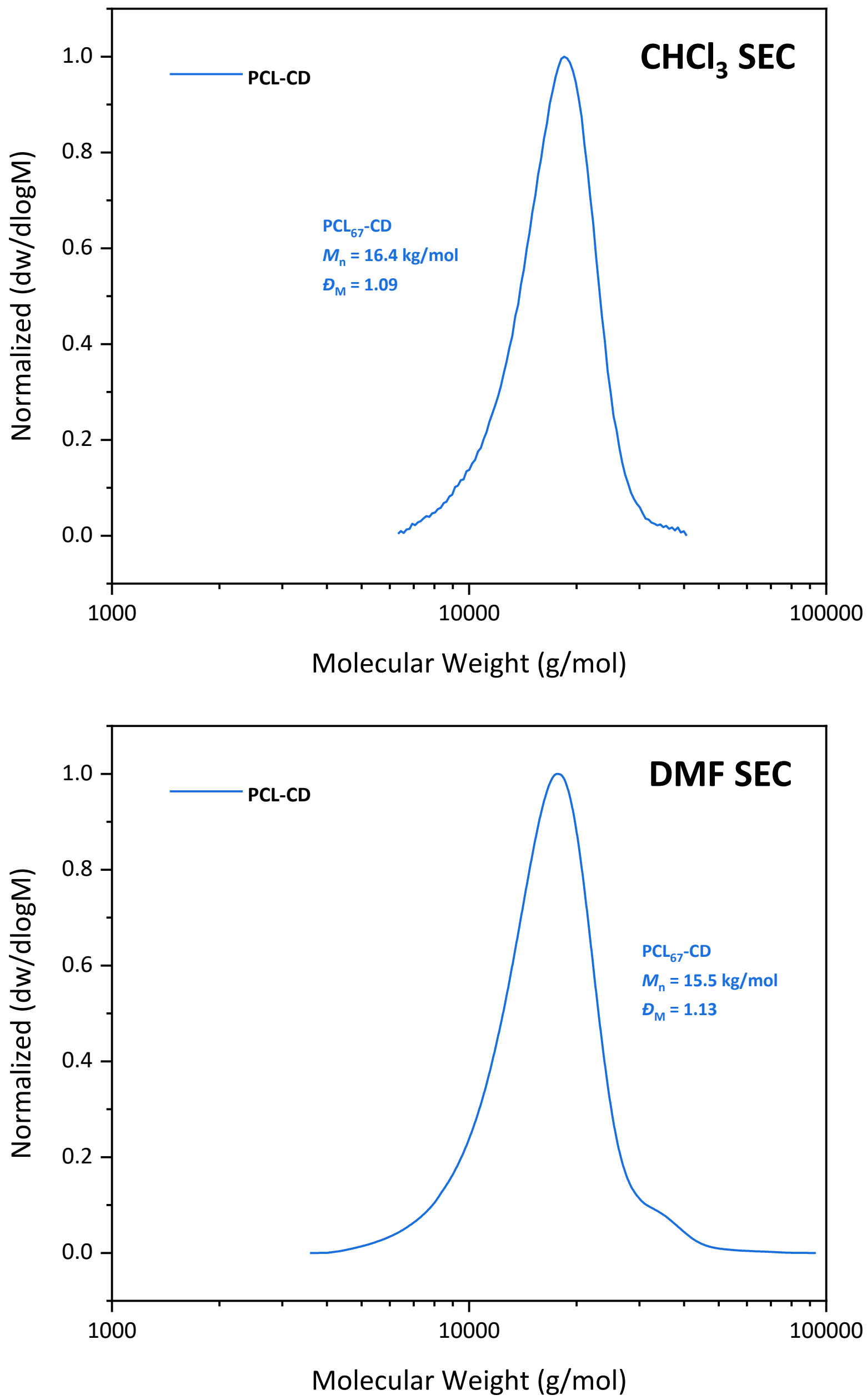

Supplementary Figure S60. Size exclusion chromatogram (RI trace) of PCL_{67}-CD using $CHCl_3$ with 0.5% NEt_3 or DMF with 5 mM NH_4BF_4 as an eluent (PMMA standard).

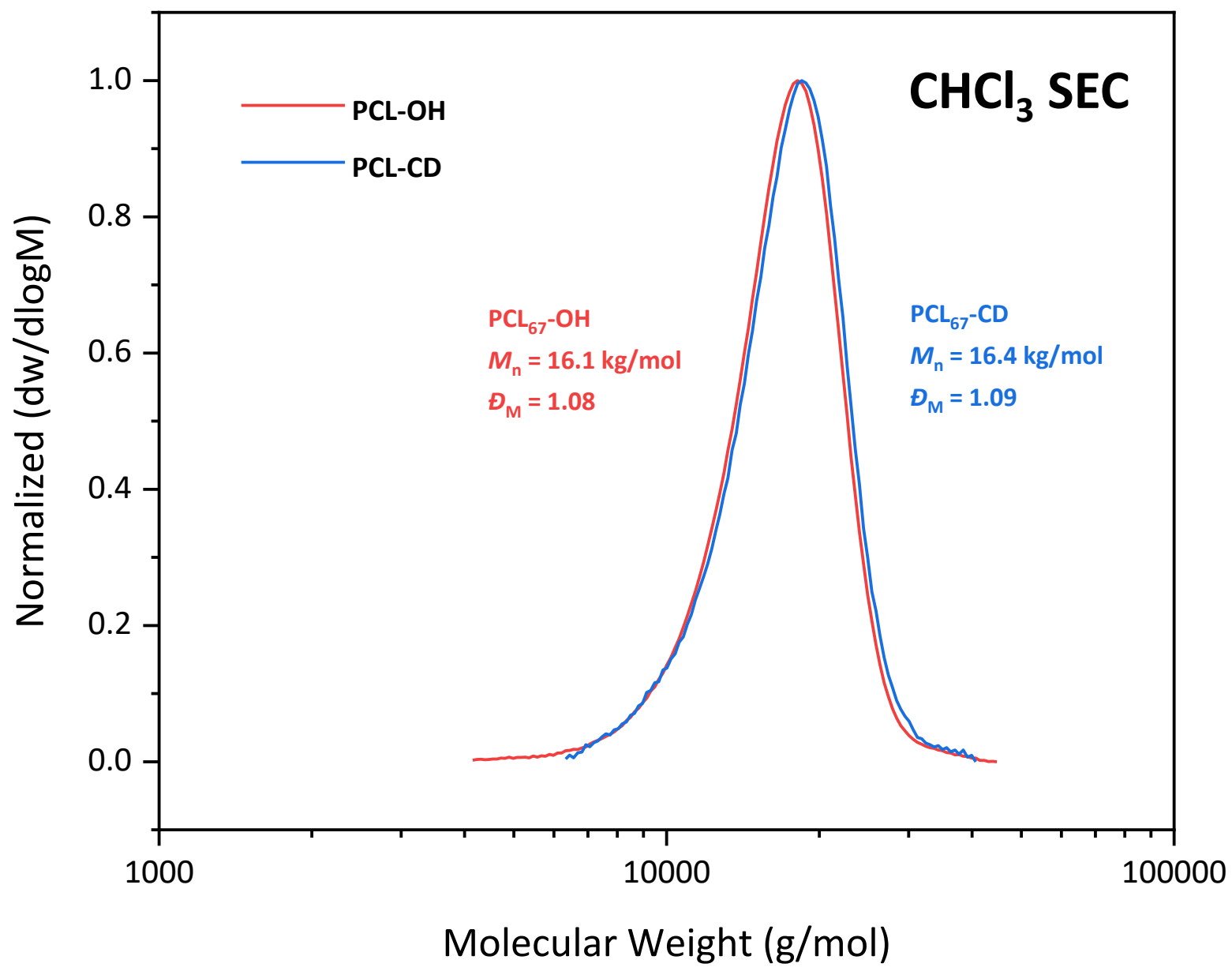

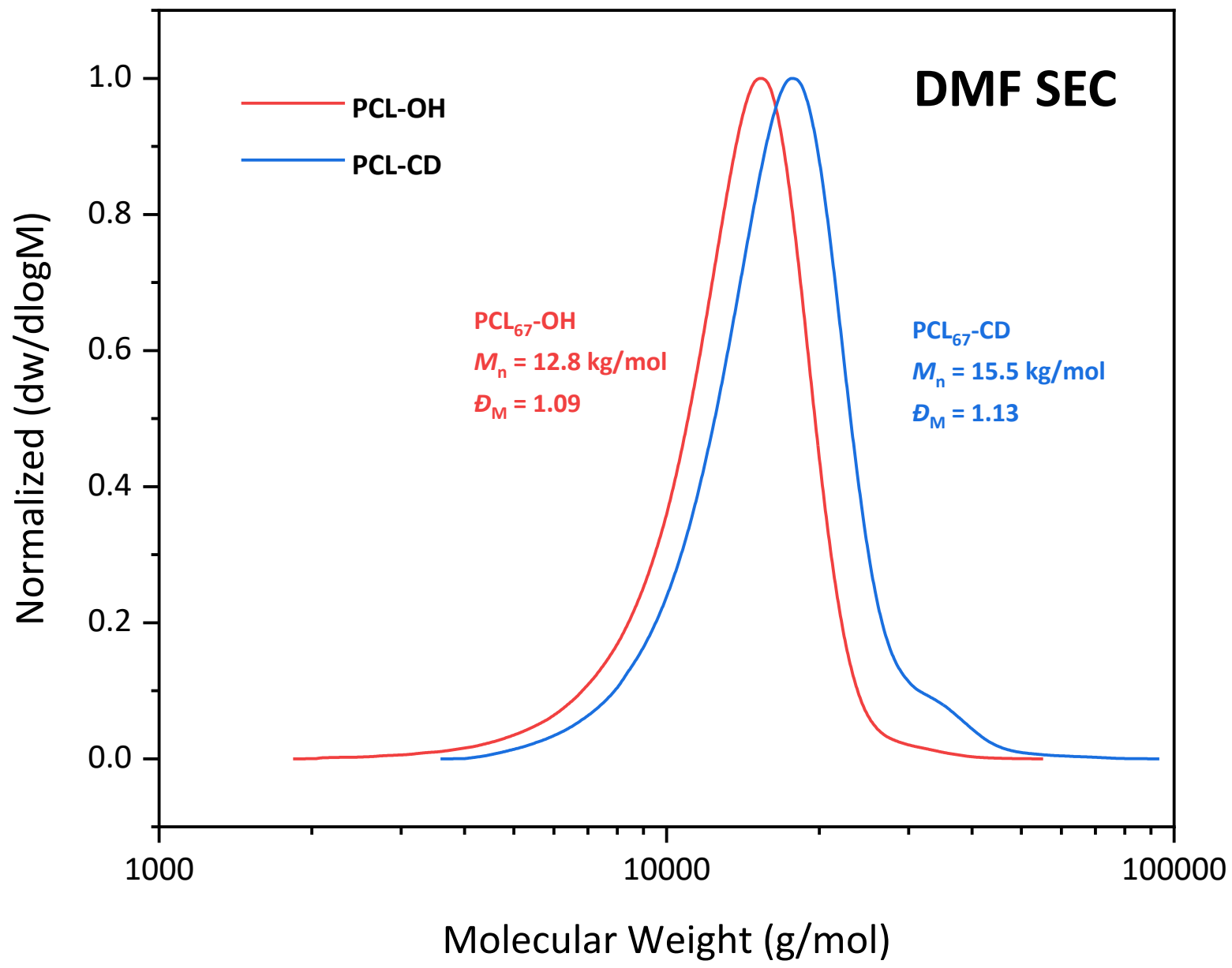

Supplementary Figure S61. Size exclusion chromatogram (RI trace) of PCL_{67}-OH and PCL_{67}-CD comparison using $CHCl_3$ with 0.5% NEt_3 or DMF with 5 mM NH_4BF_4 as an eluent (PMMA standard).

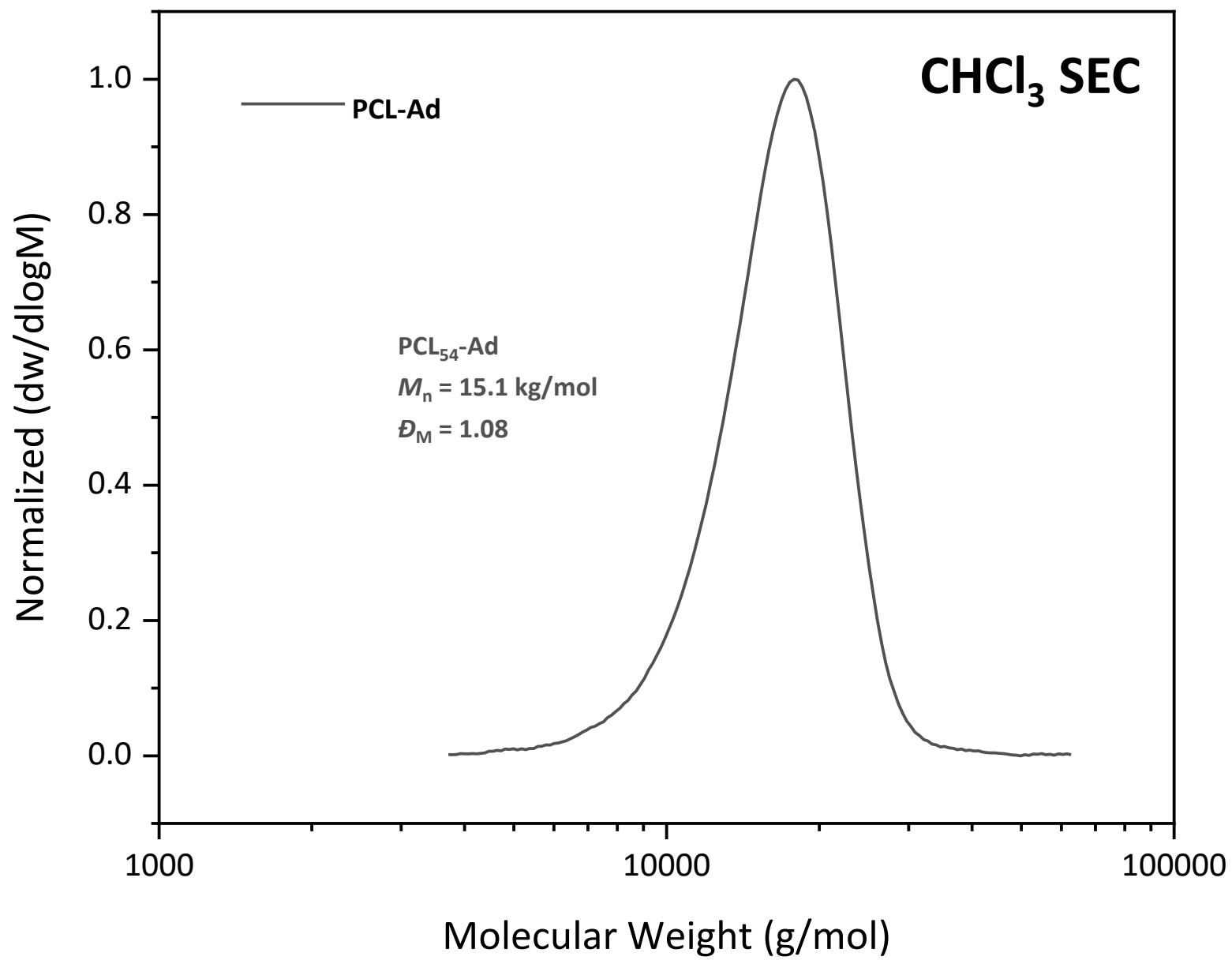

Supplementary Figure S62. Size exclusion chromatogram (RI trace) of PCL_{54}-Ad using $CHCl_3$ with 0.5% NEt_3 as an eluent (PMMA standard).

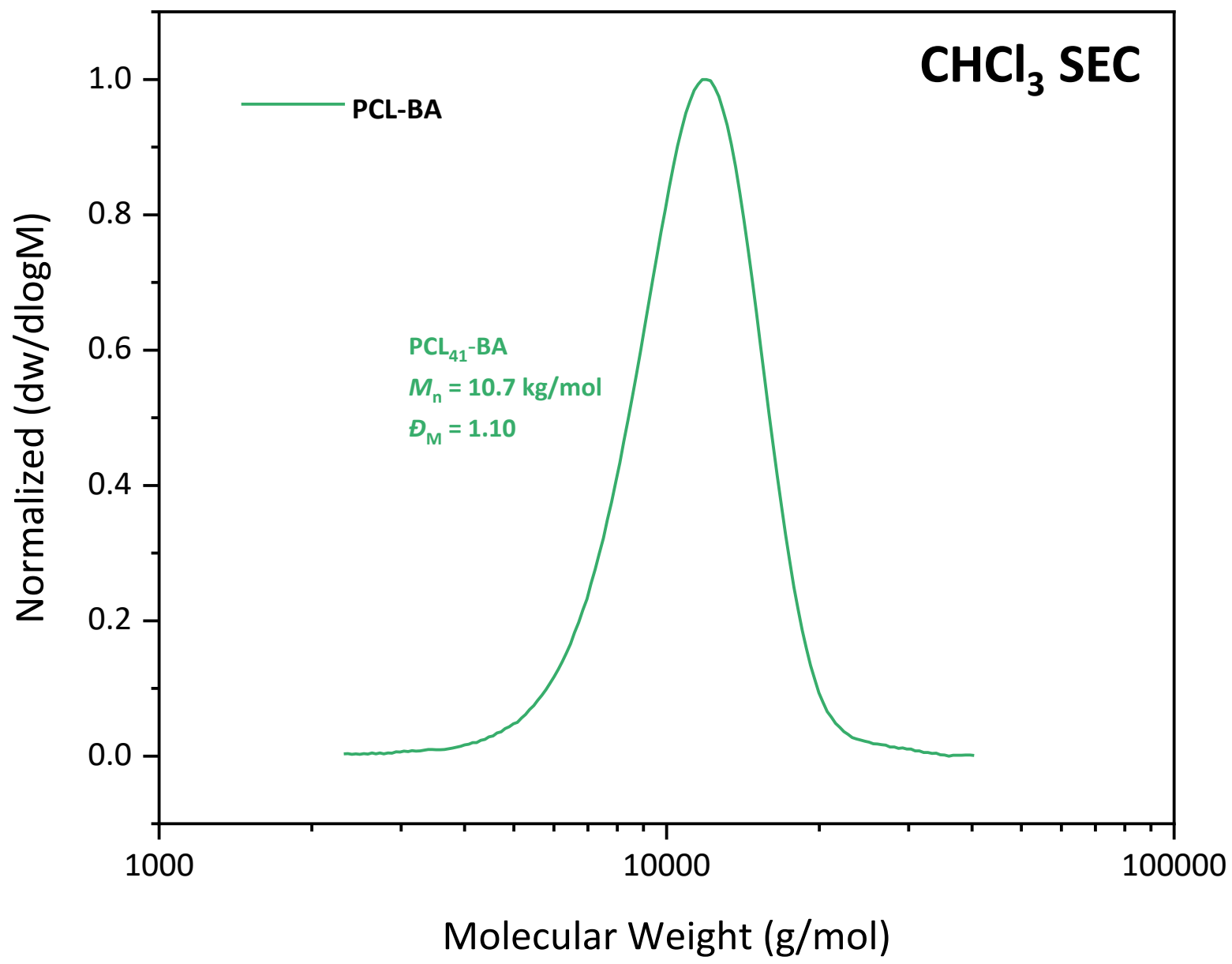

Supplementary Figure S63. Size exclusion chromatogram (RI trace) of PCL_{41}-BA using $CHCl_3$ with 0.5% NEt_3 as an eluent (PMMA standard).

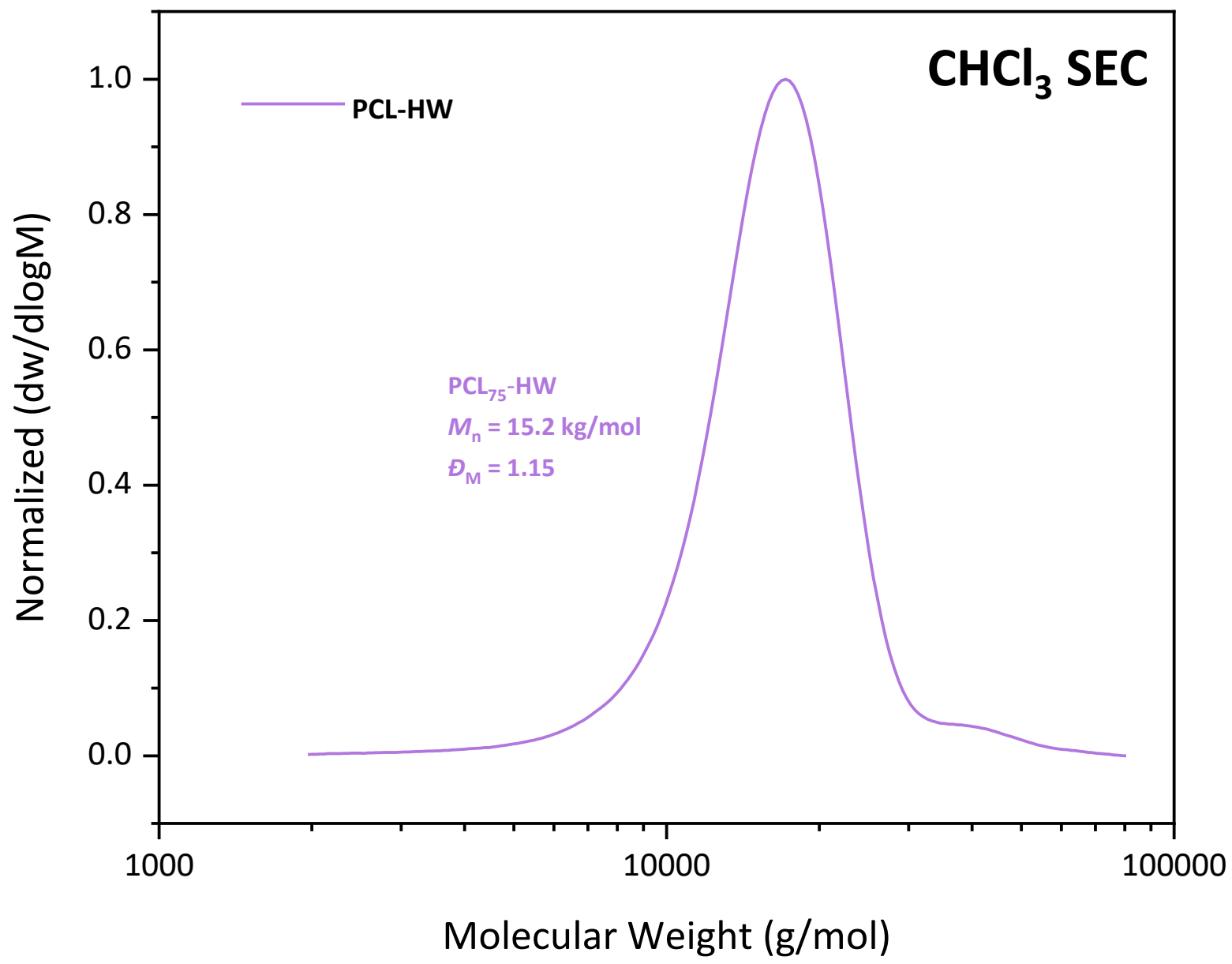

Supplementary Figure S64. Size exclusion chromatogram (RI trace) of PCL_{75}-HW using $CHCl_3$ with 0.5% NEt_3 as an eluent (PMMA standard).

Polymer information table

Supplementary Table S1. Molecular characterization of the polymers used in this study.

**Polymer**	**Eluent solvent**	**$M_{n,NMR}$ (kg/mol)[a]**	**$M_{n,SEC}$ (kg/mol)[b]**	**$Đ_M$[b]**
PCL_{50}-CTA	$CHCl_3$	6.0	12.0	1.07
PCL_{50}-*b*-$PDMA_{193}$	$CHCl_3$	25.1	30.8	1.12
PCL_{67}-OH	$CHCl_3$	7.8	16.1	1.08
	DMF		12.8	1.09
PCL_{67}-CD	$CHCl_3$	8.9	16.4	1.09
	DMF		15.5	1.13
PCL_{54}-Ad	$CHCl_3$	6.4	15.1	1.08
PCL_{41}-BA	$CHCl_3$	4.9	10.7	1.10
PCL_{75}-HW	$CHCl_3$	9.1	15.2	1.15

[a]The polymer compositions were determined from ^{1}H NMR spectra. [b]The dispersity was obtained from SEC analysis using $CHCl_3$ or DMF as the eluent.

Living CDSA and supramolecular interaction

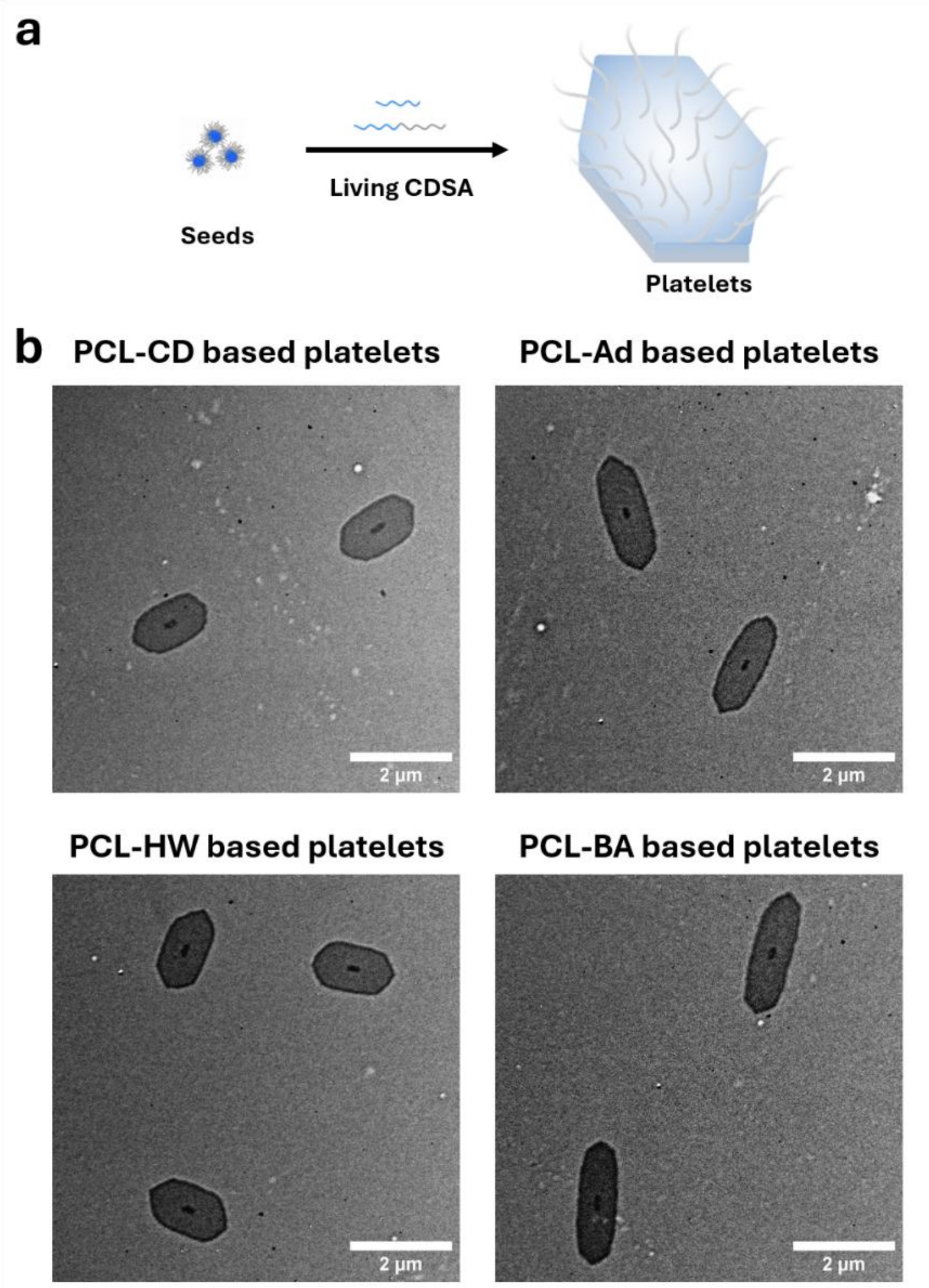

Supplementary Figure S65. (a) Schematic illustration of the preparation of supramolecularly functionalized platelets via the living CDSA method. (b) TEM images of PCL-CD, PCL-Ad, PCL-BA, and PCL-HW based platelets prepared from mixtures of supramolecular motif-functionalized PCL homopolymers (PCL_{67}-CD, PCL_{54}-Ad, PCL_{41}-BA, PCL_{75}-HW) and PCL_{50}-*b*-$PDMA_{193}$ block copolymers (10 mg mL^{-1}, m_{HP}/m_{BCP} = 1:1). The incorporation of supramolecular units does not interfere with the CDSA process, as evidenced by the preservation of characteristic crystallization behaviour and well-defined platelet morphology. Scale bar: 2 µm.

According to our previous report,[3] the PCL chain length will not disrupt the self-assembly process and the morphology of platelets, but the PCL length will affect platelets shape of aspect ratio, longer PCL will produce lower aspect ratio platelets.

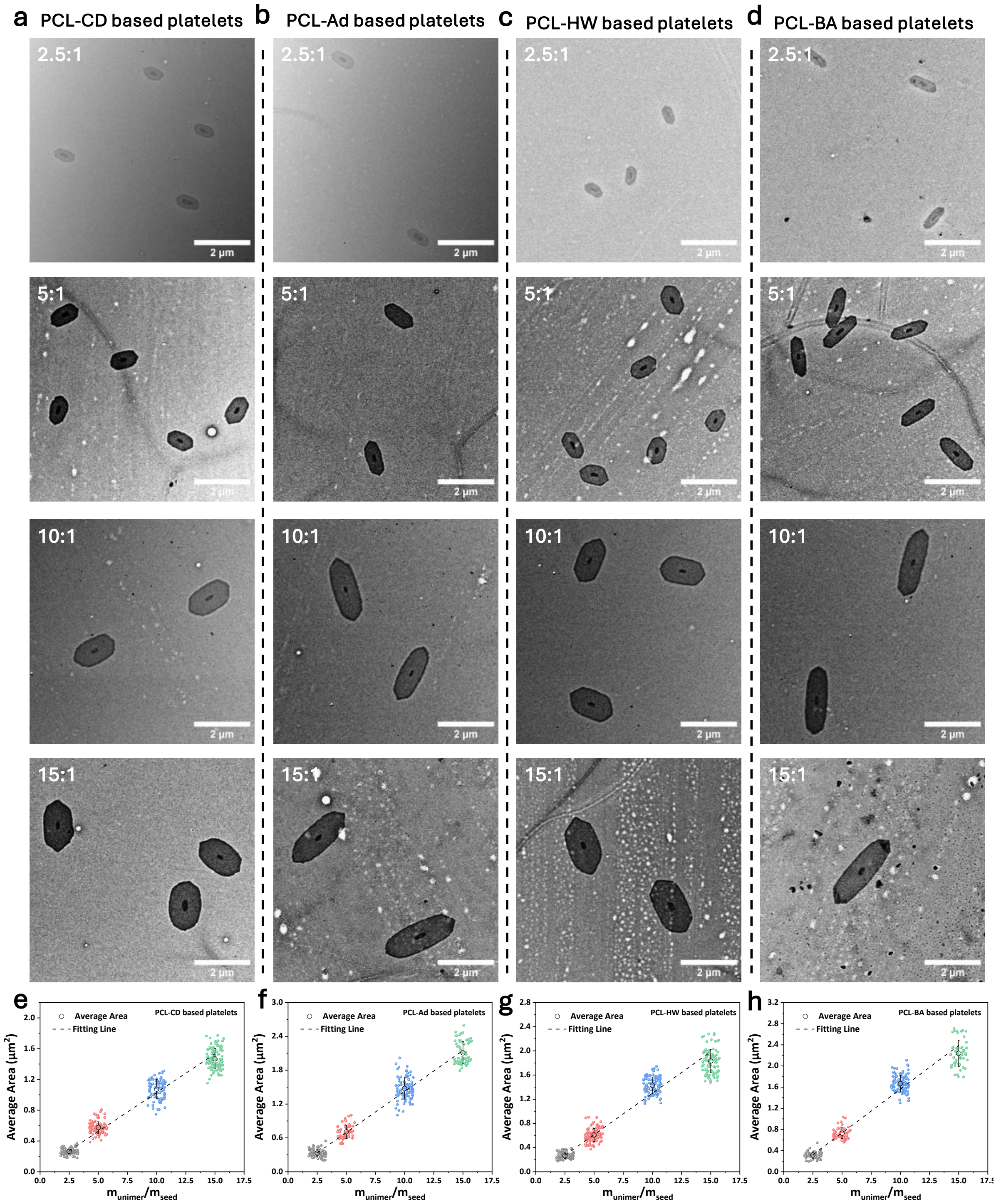

Supplementary Figure S66. Living CDSA of PCL-X/ PCL_{50}-*b*-$PDMA_{193}$ platelets. (a-d) TEM images of PCL-CD, PCL-Ad, PCL-BA, and PCL-HW based platelets prepared from mixtures of supramolecular motif-functionalized PCL homopolymers (PCL_{67}-CD, PCL_{54}-Ad, PCL_{41}-BA, PCL_{75}-HW) and PCL_{50}-*b*-$PDMA_{193}$ block copolymers (10 mg mL^{-1}, m_{HP}/m_{BCP} = 1:1) with unimer-to-seed ratios of 2.5, 5, 10 and 15. (e-h) Plots of area of supramolecularly functionalized platelets against unimer-to-seed ratios. Scale bar: 2 μm.

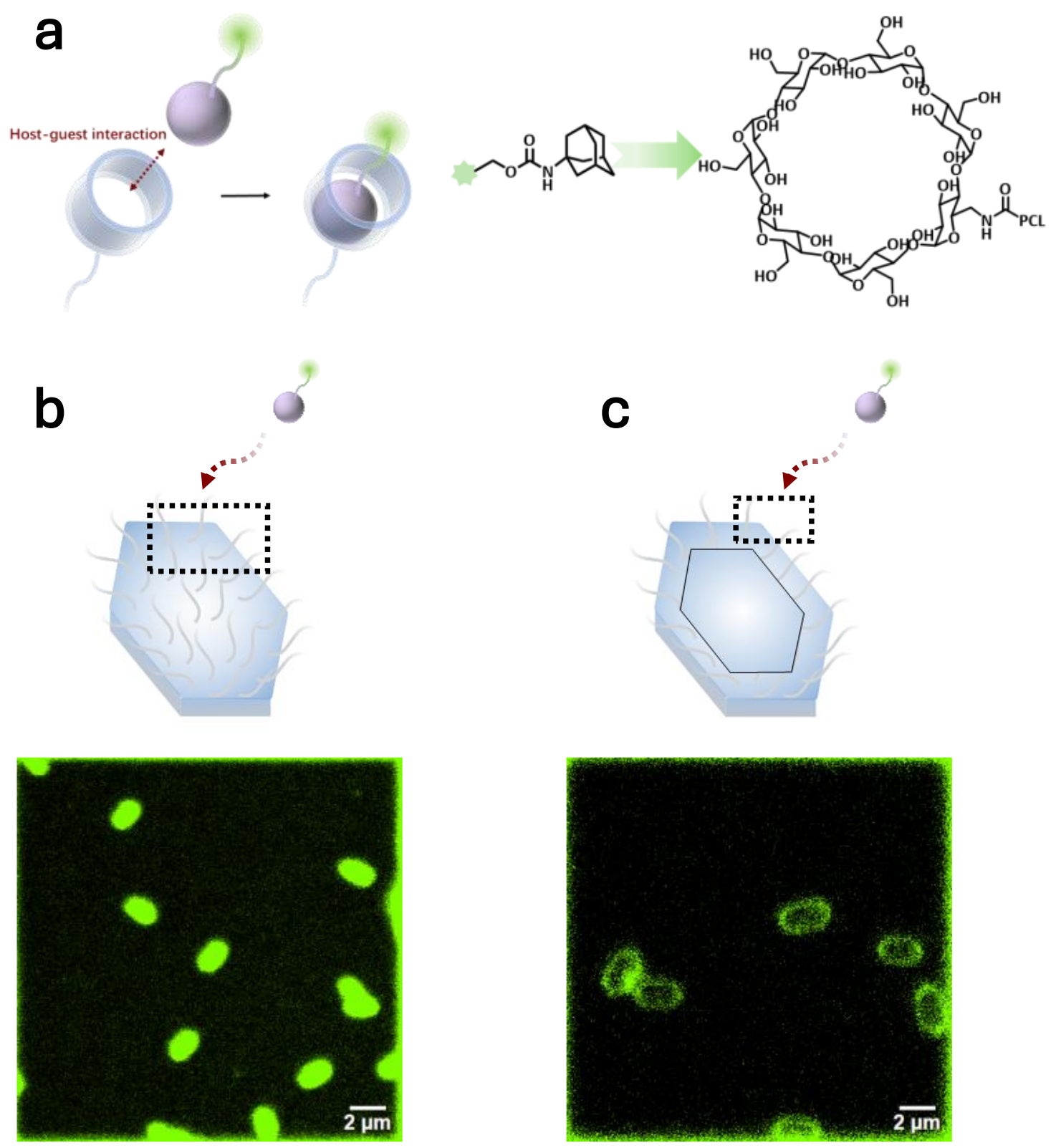

Supplementary Figure S67. Host–guest interactions between PCL-CD based platelets and adamantane-functionalized dye (NMI-Ad). (a) Schematic of host–guest binding between CD units on the platelet surface and Ad-modified fluorescent dye. (b) When CD units are uniformly distributed across the entire platelet, the dye exhibits homogeneous binding. (c) When CD motifs are selectively localized to the outer layer of double-layered platelets, the dye is confined to the platelet periphery, producing a distinct ring-like fluorescence pattern. Samples were imaged at 60× magnification upon excitation using a λ_{ex} = 405 nm laser (green channel, λ_{em} = 450–550 nm). Scale bar: 2 μm.

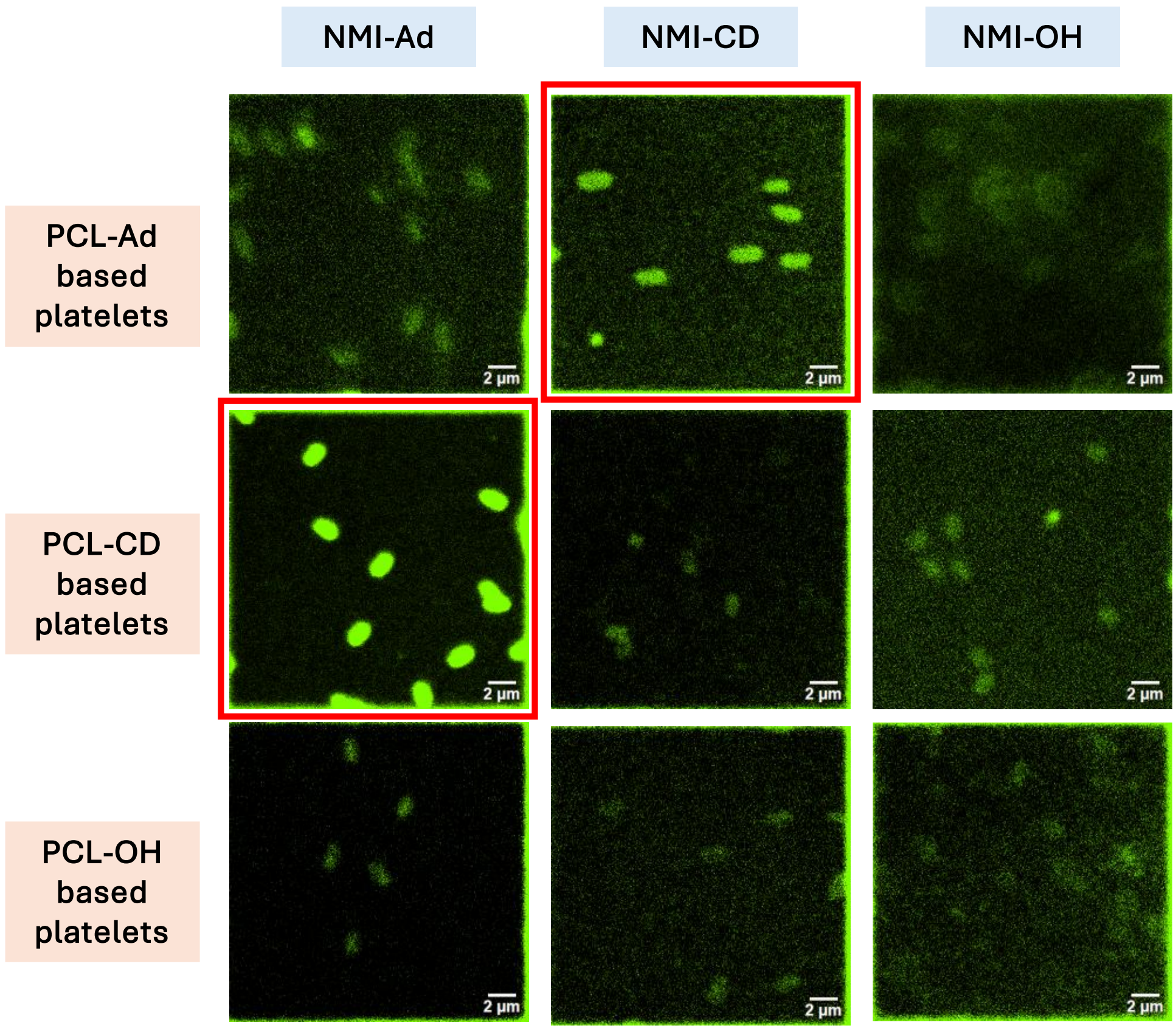

Supplementary Figure S68. Confocal fluorescence images showing different combinations of platelets (PCL-Ad, PCL-CD, and PCL-OH based) mixed with different NMI dyes (NMI-Ad, NMI-CD, and NMI-OH). The results demonstrate the selective host–guest interactions between the platelets and the dyes. Strong fluorescence observed for matching host–guest pairs (highlighted in red boxes) confirm specific binding, while weaker or diffuse signals indicate non-specific adsorption or minimal interaction. Samples were imaged at 60× magnification upon excitation using a λ_{ex} = 405 nm laser (green channel, λ_{em} = 450–550 nm). Scale bar: 2 μm.

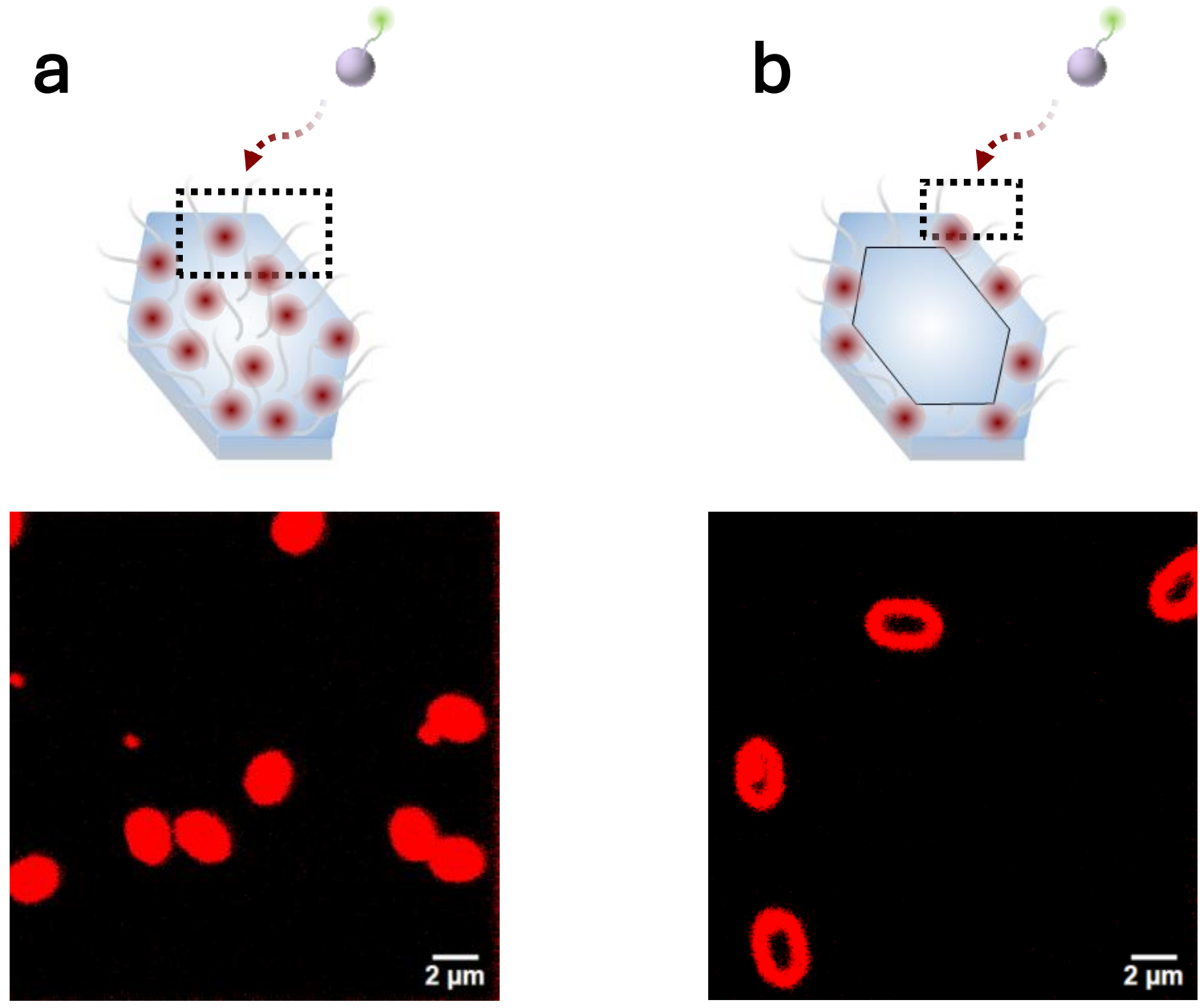

Supplementary Figure S69. Confocal fluorescence images and schematic illustrations showing supramolecular localization and Förster resonance energy transfer (FRET) emission in platelet assemblies. (a) CD units and PCL-BDP are uniformly distributed across the entire platelet surface, resulting in homogeneous dye binding and FRET emission throughout the platelet. (b) CD units and PCL-BDP are confined to the outer layer of double-layered platelets, leading to selective peripheral dye localization and region-specific FRET emission (excitation at 405 nm; emission detection window: 650–750 nm). Samples were imaged at 60× magnification upon excitation using a λ_{ex} = 405 nm laser (red channel, λ_{em} = 650–750 nm). Scale bar: 2 μm.

To enable FRET, BDP was covalently linked to PCL and positioned at the platelet surface via living CDSA, while NMI-Ad was noncovalently attached through host–guest interactions. The observed FRET signal confirms that the distance between the donor (NMI) and acceptor is within 10 nm, verifying close spatial proximity and successful supramolecular binding at the designated region.

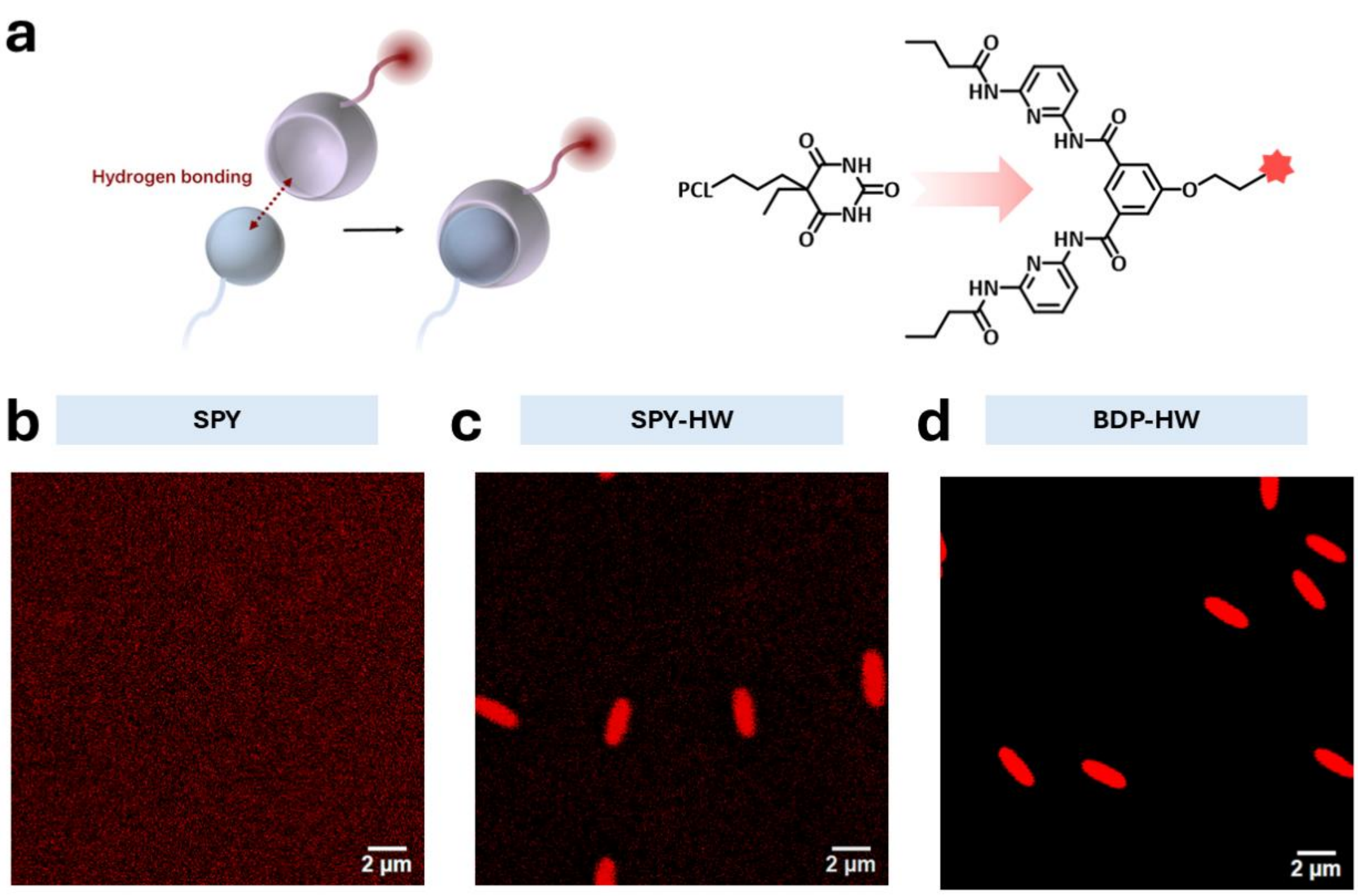

Supplementary Figure S70. Selective supramolecular recognition via sextuple hydrogen-bonding between PCL-BA based platelets and fluorescent dye molecules. (a) Schematic illustration of the interaction between BA motifs on the platelet surface and dyes bearing complementary sextuple hydrogen-bonding (HW) motifs. (b) Control dye (SPY), lacking hydrogen-bonding capability, remains uniformly dispersed without associating with the platelet structures. (c) HW-functionalized spiropyran dye (SPY-HW) exhibits selective binding to PCL-BA based platelets, leading to discrete red fluorescence localized on the platelets. (d) Similarly, HW-functionalized BDP dye (BDP-HW) binds selectively to PCL-BA based platelets, producing clearly localized red fluorescent signals. Samples were imaged at 60× magnification upon excitation using a λ_{ex} = 640 nm laser (red channel, λ_{em} = 650–750 nm). Scale bar: 2 μm.

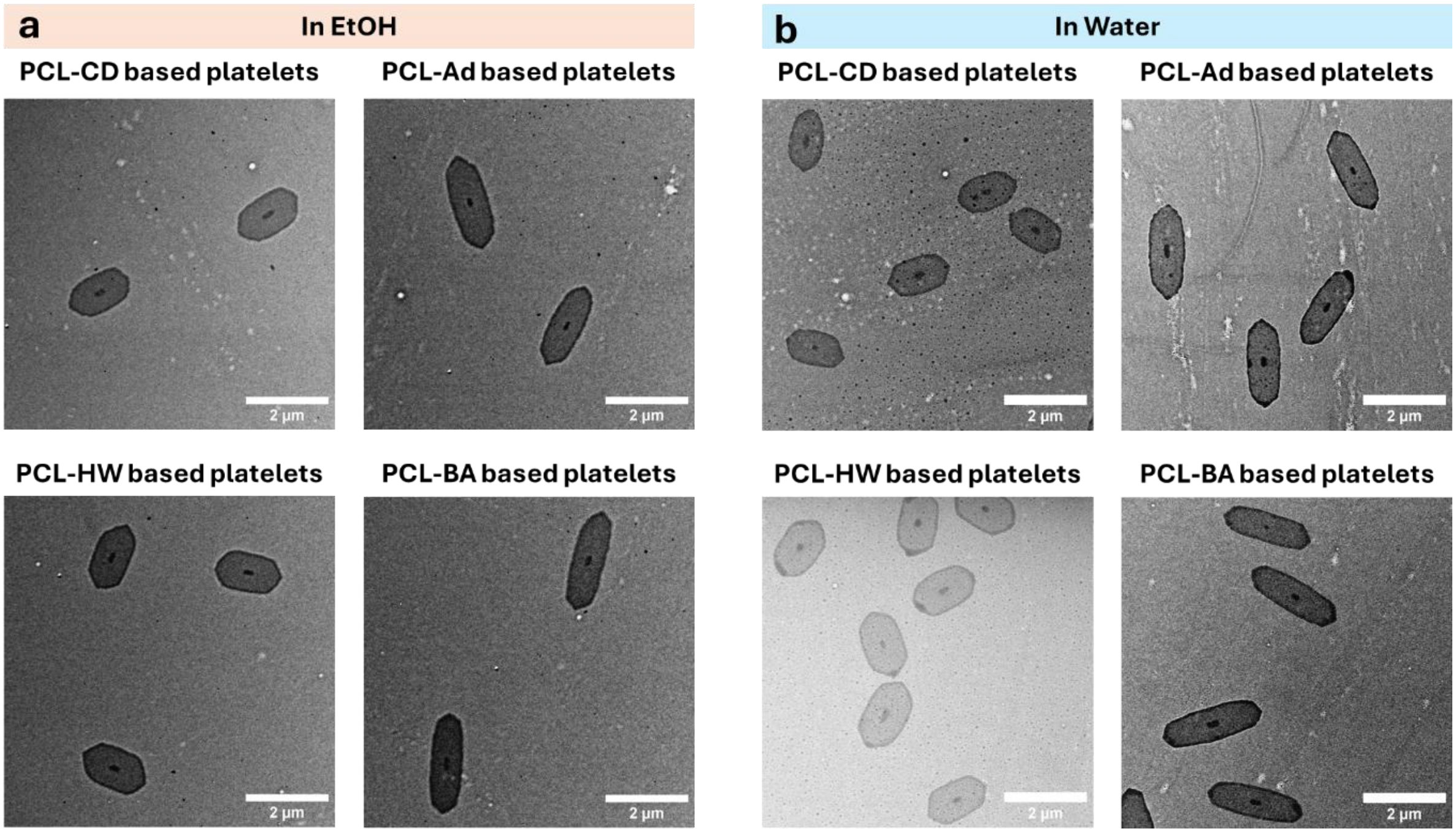

Supplementary Figure S71. (a) TEM images of PCL-CD, PCL-Ad, PCL-BA, and PCL-HW based platelets prepared via living CDSA in ethanol. (b) Corresponding TEM images after transfer into water, demonstrating preservation of the platelet morphology and structural integrity. Scale bar: 2 µm.

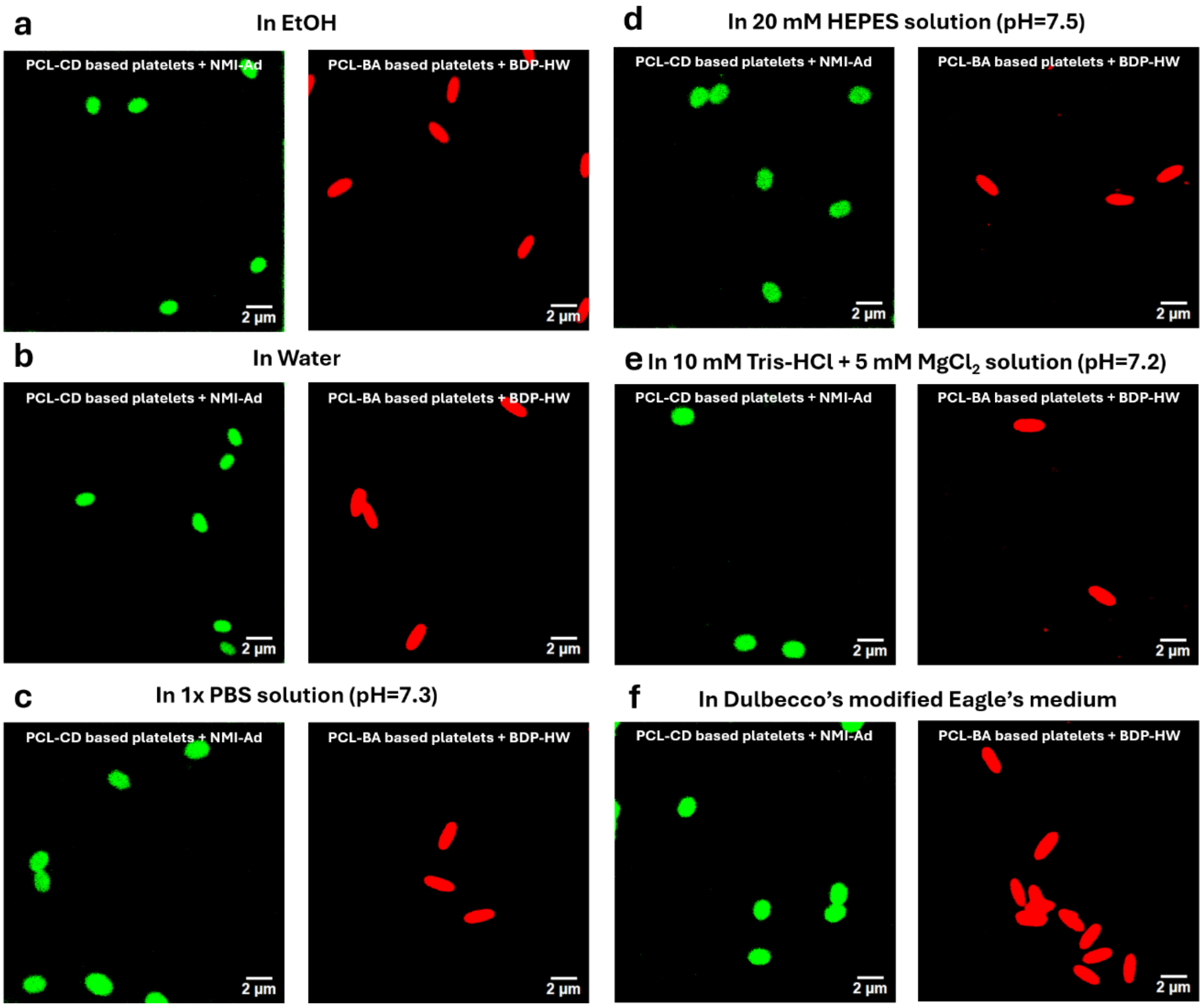

Supplementary Figure S72. Noncovalent interactions enabling targeted dye localization on polymeric platelets in aqueous media. Confocal fluorescence images of PCL-CD based platelets mixed with NMI-Ad and PCL-BA based platelets mixed with BDP-HW in (a) ethanol, (b) water, (c) 1× phosphate-buffered saline (PBS, pH 7.3), (d) 20 mM HEPES buffer (pH 7.5), (e) 10 mM Tris-HCl, 5 mM $MgCl_2$ buffer (pH 7.2), and (f) Dulbecco's modified Eagle's medium (DMEM). Platelet-localized green or red fluorescence indicates that the CD/Ad host–guest interaction and BA/HW hydrogen-bonding interaction are retained under the tested aqueous conditions. Samples were imaged at 60× magnification upon excitation using a λ_{ex} = 405 nm laser (green channel, λ_{em} = 450–550 nm), and 640 nm laser (red channel, λ_{em} = 650–750 nm). Scale bar: 2 µm.

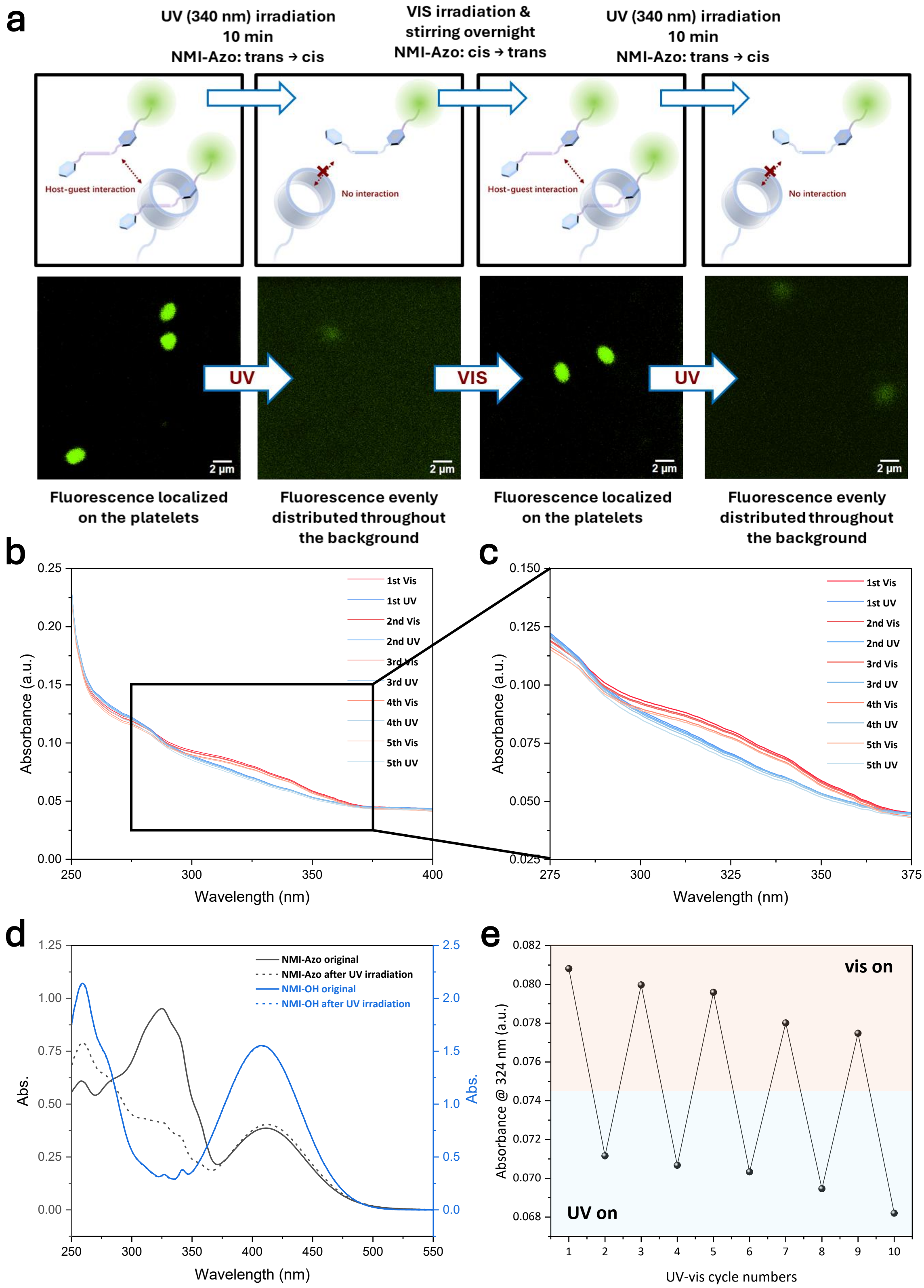

Supplementary Figure S73. UV–vis absorbance switching under alternating UV and visible light irradiation. (a) Schematic illustration of the light-responsiveness of host–guest interactions between CD and Azo. (b) UV–vis absorbance spectra recorded from 250 to 400

nm over five irradiation cycles. (c) Enlarged view of the spectra between 275 and 375 nm, highlighting the reversible spectral changes. (d) UV-vis absorbance spectra of NMI-Azo and NMI-OH before and after UV irradiation. (e) Evolution of absorbance at 324 nm over ten UV–vis switching cycles, demonstrating the repeatable photo response upon alternating UV and visible light exposure.

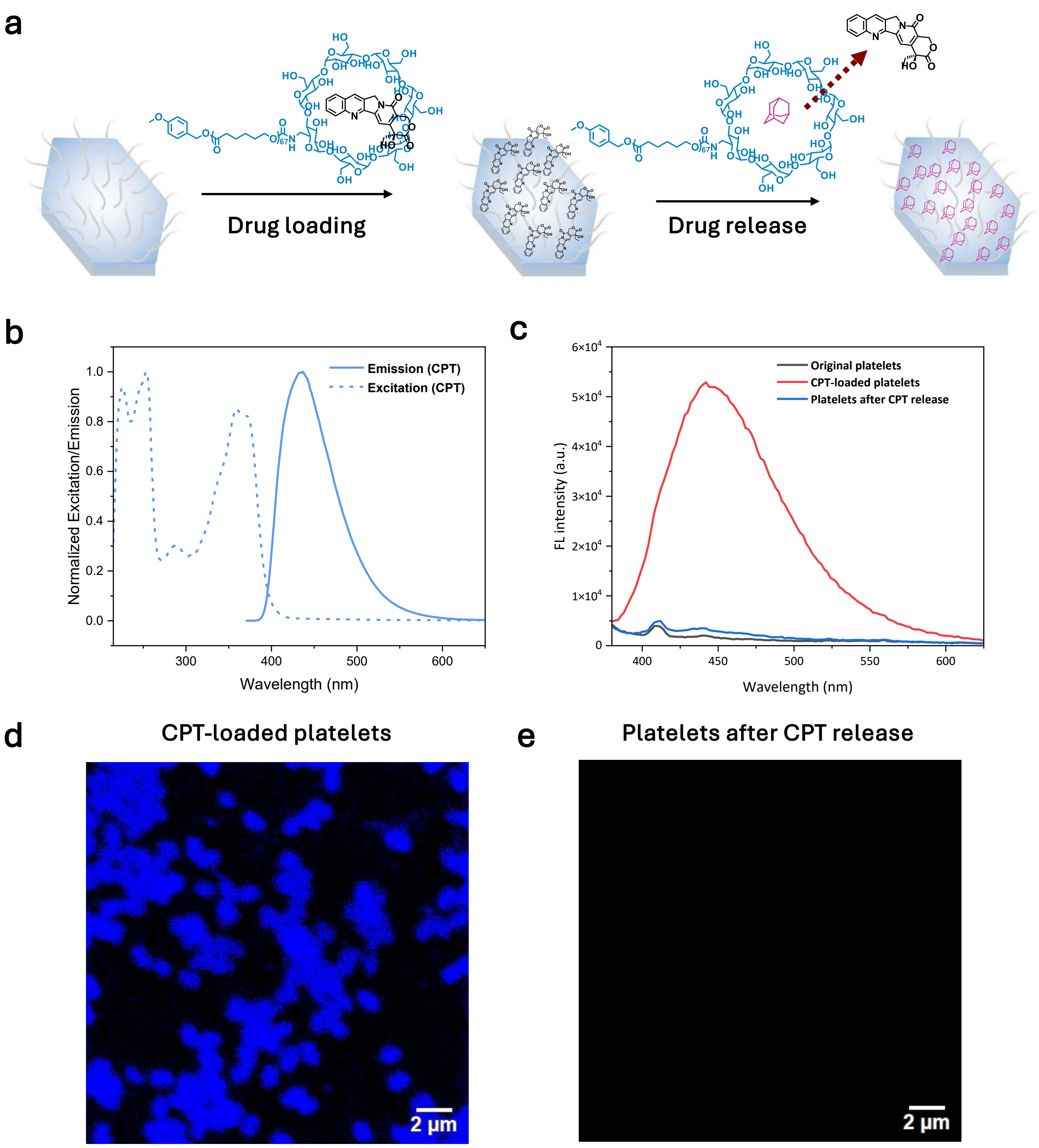

Supplementary Figure S74. (a) Schematic illustration of controllable CPT loading and release on PCL-CD based platelets via host–guest interactions. (b) Normalized excitation (dashed) and emission (solid) spectra of CPT. (c) Fluorescence spectra of original platelets, CPT-loaded platelets, and platelets after CPT release. (d) Confocal image of CPT-loaded PCL-CD based platelets. (e) Confocal image of platelets after CPT release. Samples were imaged at 60× magnification upon excitation using a λ_{ex} = 405 nm laser (blue channel, λ_{em} = 410–510 nm). Scale bar: 2 μm.

CPT can be loaded onto PCL-CD platelets through host–guest inclusion with β-cyclodextrin, leading to a strong fluorescence signal in both fluorescence spectra and confocal imaging

(**Figure S74a–d**). After addition of a competitive guest (adamantane), CPT is displaced from the cyclodextrin cavity, resulting in a significant decrease in fluorescence intensity and nearly complete disappearance of the signal in confocal images (Figure **S74a**, **c**, and **e**). These results confirm reversible binding and release of CPT, demonstrating a controllable supramolecular loading/release process in aqueous media.

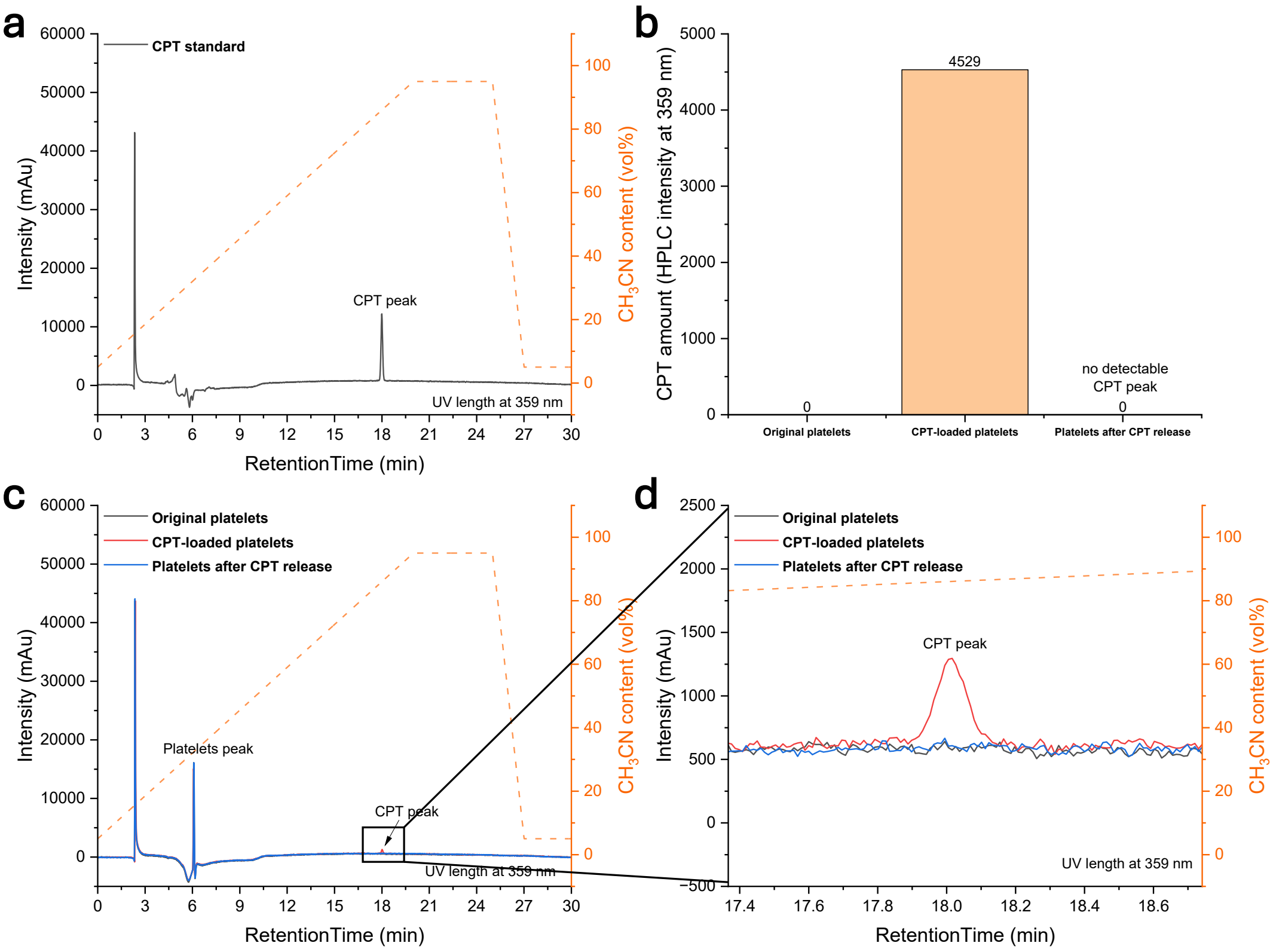

Supplementary Figure S75. HPLC analysis of controllable CPT loading and release on PCL-CD-based platelets via host–guest interactions. (a) HPLC chromatogram of a CPT standard (monitored at 359 nm). (b) Integrated peak area of CPT (from panel c) for original platelets, CPT-loaded platelets, and platelets after CPT release. (c) HPLC chromatograms monitored at 359 nm for original platelets, CPT-loaded platelets, and platelets after CPT release. (d) Enlarged view of the CPT peak region.

The CPT-loaded platelets exhibit a clear characteristic peak at the same retention time as the CPT standard, confirming successful CPT loading (**Figure S75**). In contrast, no detectable CPT peak is observed for the original platelets or after competitive displacement with adamantane, indicating efficient removal of the CPT (**Figure S75**). The analysis of peak areas further supports the reversible and controllable nature of CPT binding and release from the platelet assemblies.

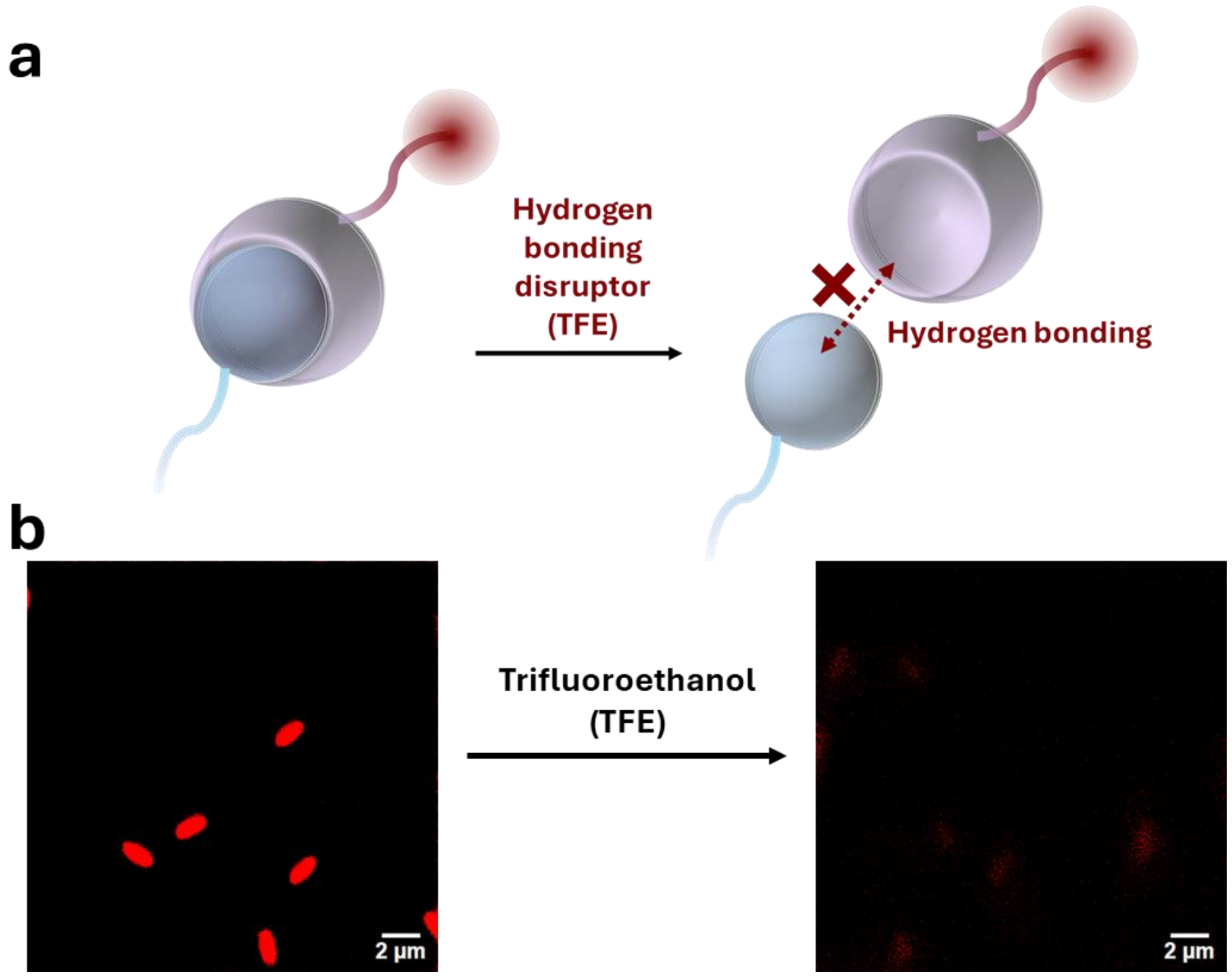

Supplementary Figure S76. (a) Schematic illustration of dynamic interactions in the sextuple hydrogen-bonding system upon addition of the hydrogen-bonding disruptor TFE. (b) Confocal images of PCL-BA based platelets coupled with SPY-HW before and after treatment with TFE. Samples were imaged at 60× magnification upon excitation using a λ_{ex} = 640 nm laser (red channel, λ_{em} = 650–750 nm). Scale bar: 2 μm.

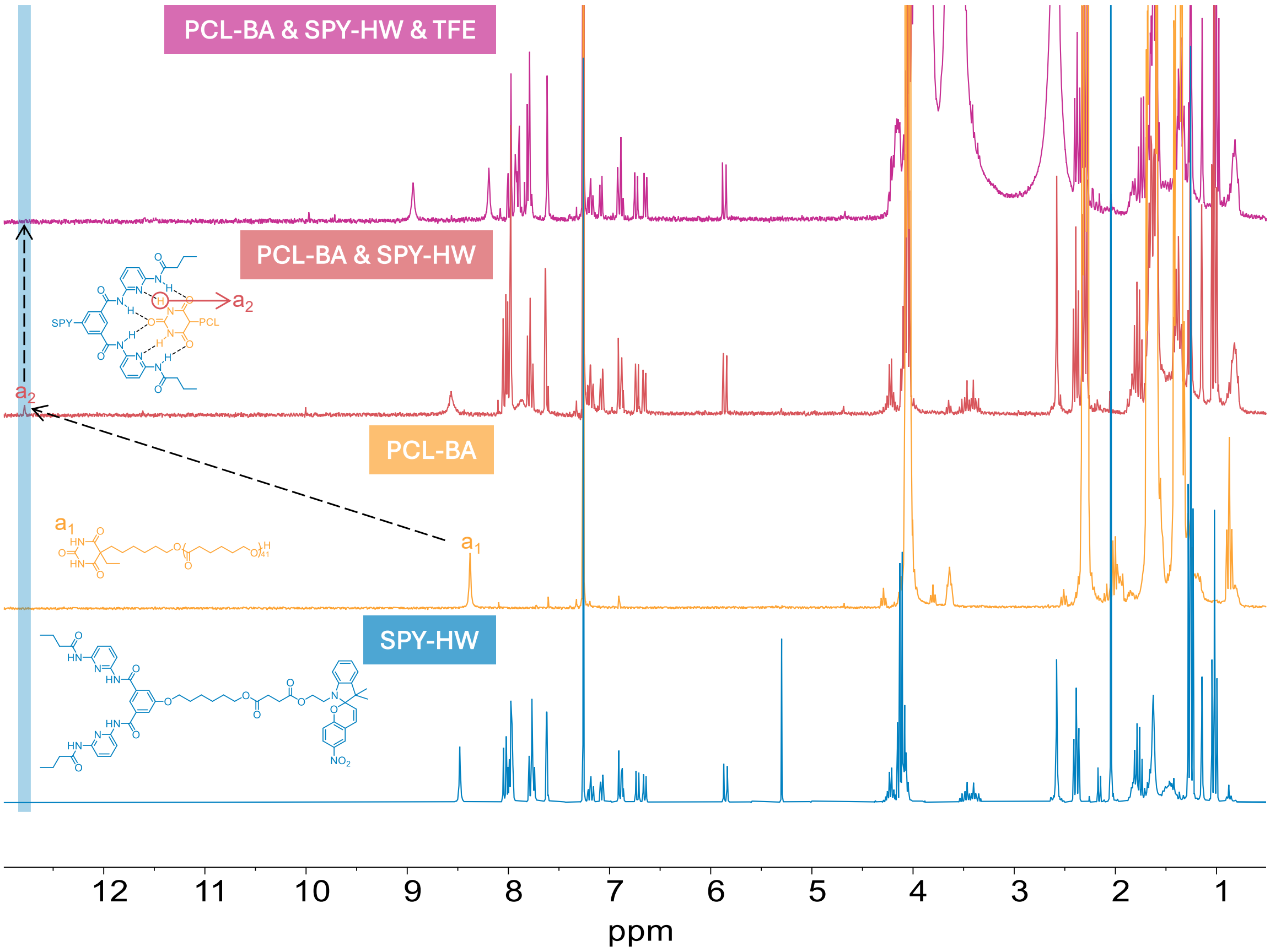

Supplementary Figure S77. ^{1}H NMR spectra ($CDCl_3$, 400 MHz, 298 K) of SPY-HW, PCL-BA, PCL-BA and SPY-HW, and PCL-BA, SPY-HW, and TFE. Upon combining PCL-BA with SPY-HW, the H_{a1} signals shifted downfield to H_{a2}, indicating hydrogen-bonding interactions. Addition of TFE disrupted these interactions, leading to the disappearance of H_{a2}.

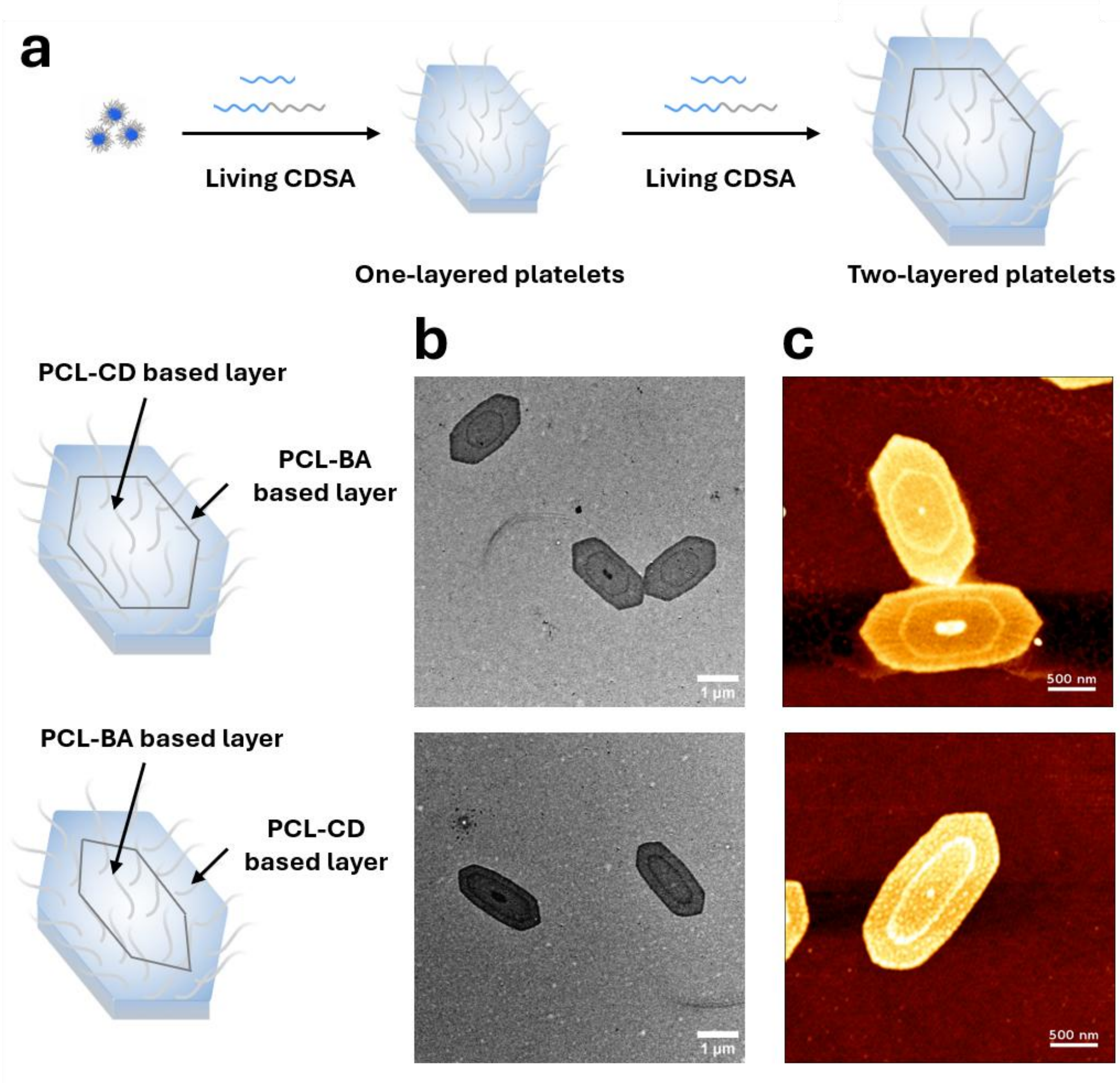

Supplementary Figure S78. (a) Schematic illustration of the preparation of two-layered supramolecularly functionalized platelets via sequential living CDSA. (b) Representative TEM images (scale bar: 1 μm) and (c) AFM images (scale bar: 500 nm) of the resulting two-layered platelets, composed of a PCL-CD-based layer and a PCL-BA-based layer.

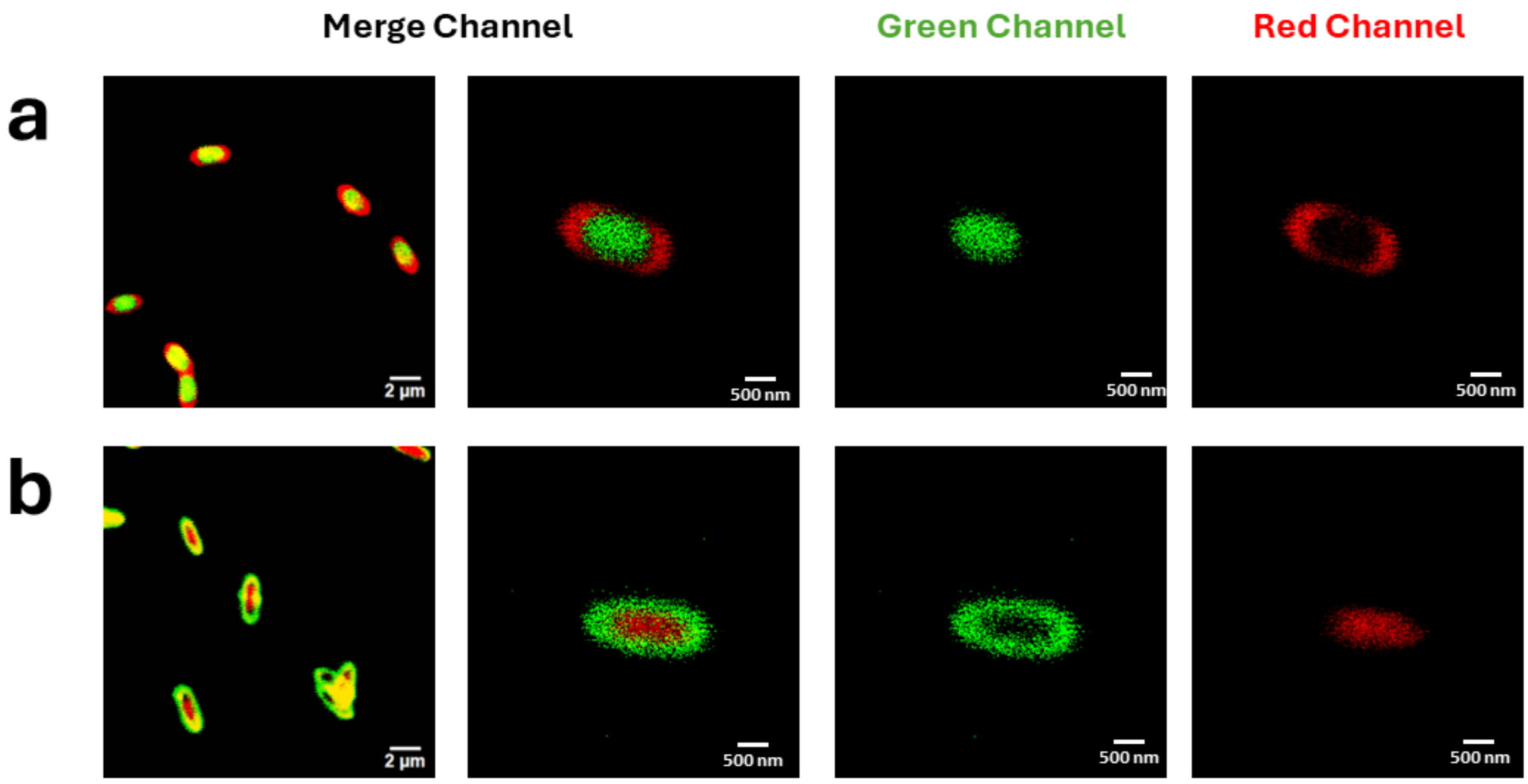

Supplementary Figure S79. Confocal fluorescence images showing region-selective functionalization using host–guest and sextuple hydrogen-bonding interactions. (a) Platelets with host–guest interactions in the inner layer and sextuple hydrogen-bonding in the outer layer. (b) Platelets with sextuple hydrogen-bonding in the inner layer and host–guest interactions in the outer layer. Samples were imaged at 60× magnification upon excitation using a λ_{ex} = 405 nm laser (green channel, λ_{em} = 450–550 nm), and 640 nm laser (red channel, λ_{em} = 650–750 nm). Scale bars: 2 µm (first column) and 500 nm (other columns).

Living CDSA enables spatially controlled placement of orthogonal supramolecular motifs. Upon addition of corresponding dyes, selective localization confirms that the two interaction types operate independently without interference.

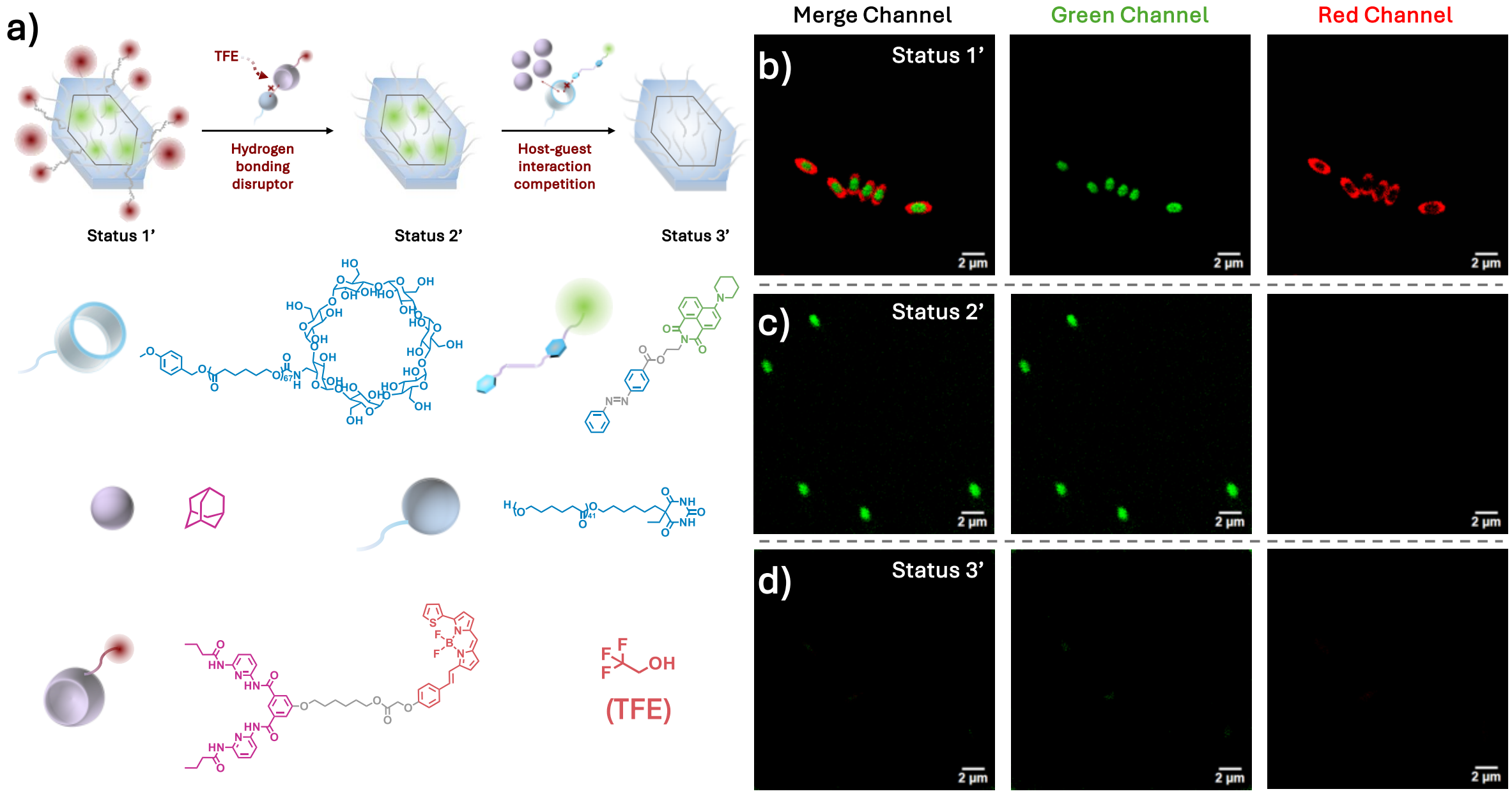

Supplementary Figure S80. Controllable 'wipe-clean' functionality of supramolecular platelets. (**a**) Schematic illustration of sequential fluorescence erasure: initial double-layered platelets functionalized via host–guest (green) and sextuple hydrogen-bonding (red) interactions (**Status 1'**); addition of TFE disrupts hydrogen-bonding, erasing red fluorescence (**Status 2'**); subsequent treatment with Ad displaces NMI-Azo from CD sites, selectively removing green fluorescence and returning platelets to a blank state (**Status 3'**). (**b-d**) Confocal images corresponding to **Status 1'-3'**, demonstrating stepwise, programmable removal of supramolecular signals. Samples were imaged at 60× magnification upon excitation using a λ_{ex} = 405 nm laser (green channel, λ_{em} = 450–550 nm), and 640 nm laser (red channel, λ_{em} = 650–750 nm). Scale bar: 2 μm.

The erasure sequence can also be carried out in the opposite order. Introducing TFE first disrupts the sextuple hydrogen-bonding interaction, selectively removing the HW–BA–bound dye while leaving the host–guest layer intact (**Status 2'**). Subsequent addition of free adamantane then displaces the remaining CD-bound guest, completing the second step of erasure (**Status 3'**). This reversed sequence confirms that the two supramolecular interactions can be independently addressed and that their response order can be programmed at will, demonstrating full bidirectional control and further highlighting the versatility of the "wipe-clean" process.

Reference

(1) Xia, T.; Tong, Z.; Xie, Y.; Arno, M. C.; Lei, S.; Xiao, L.; Rho, J. Y.; Ferguson, C. T. J.; Manners, I.; Dove, A. P.; O'Reilly, R. K. Tuning the Functionality of Self-Assembled 2D Platelets in the Third Dimension. *J. Am. Chem. Soc.* **2023,** *145* (46), 25274-25282.

(2) Xie, Y.; Arno, M. C.; Husband, J. T.; Torrent-Sucarrat, M.; O'Reilly, R. K. Manipulating the fluorescence lifetime at the sub-cellular scale via photo-switchable barcoding. *Nat. Commun.* **2020,** *11* (1), 2460.

(3) Xia, T. L.; Xiao, L. H.; Sun, K. W.; Rho, J. Y.; Xie, Y. J.; Parkinson, S. J.; Sangroniz, L.; Zhang, J.; Lin, J. P.; Müller, A. J.; Gao, L.; Dove, A. P.; O'Reilly, R. K. Control over Aspect Ratio and Polymer Spatial Distribution of 2D Platelets via Living Crystallization-Driven Self-Assembly. *Macromolecules* **2024,** *57* (23), 11210-11220.

(4) Chen, S.; Rocher, M.; Ladavière, C.; Gérard, J.-F.; Lortie, F.; Bernard, J. AB/ABC/ABCD supramolecular block copolymers from Hamilton wedge and barbiturate-functionalized RAFT agents. *Polym. Chem.* **2012,** *3* (11), 3157-3165.

(5) Berl, V.; Schmutz, M.; Krische, M. J.; Khoury, R. G.; Lehn, J.-M. Supramolecular Polymers Generated from Heterocomplementary Monomers Linked through Multiple Hydrogen-Bonding Arrays—Formation, Characterization, and Properties. *Chem. Eur. J.* **2002,** *8* (5), 1227-1244.

(6) Zhuang, J.; Zhou, W.; Li, X.; Li, Y.; Wang, N.; He, X.; Liu, H.; Li, Y.; Jiang, L.; Huang, C.; Cui, S.; Wang, S.; Zhu, D. Multiple hydrogen-bond-induced supramolecular nanostructure from a pincer-like molecule and a [60]fullerene derivative. *Tetrahedron* **2005,** *61* (36), 8686-8693.

(7) Hirao, T.; Kudo, H.; Amimoto, T.; Haino, T. Sequence-controlled supramolecular terpolymerization directed by specific molecular recognitions. *Nat. Commun.* **2017,** *8* (1), 634.

(8) Rajak, A.; Das, A. Crystallization-Driven Controlled Two-Dimensional (2D) Assemblies from Chromophore-Appended Poly(L-lactide)s: Highly Efficient Energy Transfer on a 2D Surface. *Angew. Chem. Int. Ed.* **2022,** *61* (15), e202116572.

(9) Xiao, L. H.; Xia, T. L.; Zhang, J.; Parkinson, S. J.; Rho, J. Y.; Dove, A. P.; O' Reilly, R. K. Direct preparation of two-dimensional platelets from polymers enabled by accelerated seed formation. *Nat. Synth.* **2025,** *4* (7), 808-815.
